# Characterizing the secretome of licensed hiPSC-derived MSCs

**DOI:** 10.1186/s13287-022-03117-2

**Published:** 2022-09-02

**Authors:** Yolande F. M. Ramos, Tobias Tertel, Georgina Shaw, Simon Staubach, Rodrigo Coutinho de Almeida, Eka Suchiman, Thomas B. Kuipers, Hailiang Mei, Frank Barry, Mary Murphy, Bernd Giebel, Ingrid Meulenbelt

**Affiliations:** 1 Department of Biomedical Data Sciences, Section Molecular Epidemiology, Leiden University Medical Center, LUMC Postzone S-05-P, P.O. Box 9600, 2300 RC Leiden, The Netherlands; 2 Institute for Transfusion Medicine, University Hospital Essen, University of Duisburg-Essen, Essen, Germany; 3 National University of Ireland Galway, Galway, Ireland; 4 LUMC, Sequencing Analysis Support Core, Leiden, The Netherlands

**Keywords:** Cell therapy, hiPSCs, Induced mesenchymal stromal cells, Immunomodulation, Extracellular vesicles, Exosomes, RNA sequencing

## Abstract

Although mesenchymal stromal cells (MSCs) from primary tissues have been successfully applied in the clinic, their expansion capabilities are limited and results are variable. MSCs derived from human-induced pluripotent stem cells (hiMSCs) are expected to overcome these limitations and serve as a reproducible and sustainable cell source. We have explored characteristics and therapeutic potential of hiMSCs in comparison to hBMSCs. RNA sequencing confirmed high resemblance, with average Pearson correlation of 0.88 and Jaccard similarity index of 0.99, and similar to hBMSCs the hiMSCs released extracellular vesicles with in vitro immunomodulatory properties. Potency assay with TNFα and IFNγ demonstrated an increase in well-known immunomodulatory genes such as *IDO1*, *CXCL8/IL8*, and *HLA-DRA* which was also highlighted by enhanced secretion in the media. Notably, expression of 125 genes increased more than 1000-fold. These genes were predicted to be regulated by NFΚB signaling, known to play a central role in immune response. Altogether, our data qualify hiMSCs as a promising source for cell therapy and/or cell-based therapeutic products. Additionally, the herewith generated database will add to our understanding of the mode of action of regenerative cell-based therapies and could be used to identify relevant potency markers.

**Supplementary Information:**

The online version contains supplementary material available at 10.1186/s13287-022-03117-2.

## Introduction

The lack of effective treatments for inflammatory diseases as well as major age-related diseases, e.g., chronic Inflammatory Bowel Disease, neurodegenerative disease and osteoarthritis, imposes a huge economic burden on individual patients and health care systems [1, 2]. In that respect, caretakers have high hopes for the application of cell therapy in the clinic using human adult mesenchymal stromal cells (hMSCs) [3]. Upon exposure to signals associated with the in vivo injured environment hMSCs are known to respond with a process named licensing. Cell licensing is characterized by increased secretion of immunomodulatory factors, including growth factors and cytokines, and extracellular vesicles (EVs), having trophic properties and establishing a regenerative environment [4–6].

The application of hMSCs has been explored for many indications with results suggesting promise for clinical efficacy. Nonetheless, results also exposed important restrictions. Among others, the availability and expansion capacity of hMSCs is limited and their therapeutic capacities are donor-dependent. Accordingly, therapeutic products based on primary hMSCs have reduced batch sizes and reveal considerable variation. This restrains the crucial standardization of therapeutic potency of hMSC products [7], and highlights the necessity to optimize production modalities for the generation of therapeutic cell products. For that matter, it is critical to characterize the hMSC-secretome and demonstrate potency and consistency in response to licensing with common licensing factors such as IFNγ and TNFα. The response of hMSC to licensing is currently widely applied to predict potency, hence immunomodulatory efficacy.

To overcome current limitations in cell therapy, the application of hMSCs derived from induced pluripotent stem cells (hiMSCs) is being explored as sustainable, reproducible, and reliable cell source. Such hiMSCs are GMP-compatible for translation into the clinic [8]. Added value of hiMSCs is their ease of access, since collection of natural hMSCs is an invasive procedure for donors. In our laboratory, we established a protocol to robustly and consistently generate hiMSCs, highly comparable to bone marrow-derived hMSCs (hBMSCs) with respect to characteristics such as morphology, surface markers, and lineage commitment [9]. In the current study, we addressed their potency by characterization of the hiMSC-secretome and the immune-suppressive activity of extracellular vesicles released by hiMSCs in the medium (hiMSC-EVs).

## Materials and methods

### Sample description and ethics approval

Ethical approval for the generation of hiPSCs from skin fibroblasts of healthy donors was obtained by the Medical Ethical Committee of the LUMC and is available under number P13.080. Control hiPSC line used in the current study was generated by the LUMC iPSC core facility from male skin fibroblasts (LUMC0004iCTRL10 (004) registered at the Human pluripotent stem cell registry. Cells were characterized according to pluripotent potential and spontaneous differentiation capacity by the iPSC core facility [10]. hiPSCs were maintained under standard conditions and are described in more detail in Additional file 1.

Human bone marrow-derived MSCs (hBMSCs) were derived from bone marrow aspirates of two healthy donors with approval from the NUI Galway Research Ethical and Galway University Hospitals Clinical Research Ethics Committees. Third hBMSC line from a healthy donor was commercially acquired (SCC034; Merck Millipore). Collection of hBMSCs from OA patients undergoing total joint replacement surgery is approved by the Medical Ethical Committee of the LUMC within the ongoing RAAK study [11] and available under numbers P08.239 and P19.013.

### Differentiation of hiPSC towards hiMSCs

Human iMSCs were generated using the Stemcell Technologies Mesenchymal Progenitor Kit following the manufacturers’ instructions with small modifications as described previously [9]. More details are provided in Additional file 1 and Additional file 1: Fig. S1.

### Preparation and characterization of EVs from MSC-conditioned cell culture media

Conditioned media from hiMSCs were harvested 48 h after refreshment. MSC-EVs were prepared from plain medium or conditioned media by polyethylene glycol 6000 precipitation followed by ultracentrifugation, as described previously [12–14]. Obtained MSC-EV preparations were diluted in NaCl-HEPES buffer (Sigma-Aldrich) to represent the yield from the conditioned media of approximately 1.6 × 10^8^ cells in 1 mL and stored on −80 °C until usage. Characterization of MSC-EV preparations according to the MISEV criteria [15] is described in Additional file 1.

### Multi-donor mixed lymphocyte reaction (mdMLR)

The immunomodulatory potential of MSC-EV preparations was compared in a multi-donor mixed lymphocyte reaction assay (mdMLR) as described previously [16]. For more details see Additional file 1.

### Licensing of hMSCs

Three days after seeding hMSCs in culture medium (DMEM high glucose (Gibco) supplemented with 10% fetal calf serum (FCS; Biowest), basic FGF (bFGF; 5 ng/ml; Life Technologies; 30,000 cells in each well of a 6-well plate), cells were licensed for a further three days by exposure to a combination of 50 ng/mL TNFα and 50 ng/mL IFNγ. Subsequently, conditioned media were collected for analyses of secreted factors with immunoassays and cells were lysed for RNA isolation and RNA sequencing (hBMSCs: duplicates of three independent donors; hiMSCs: six independent replicates of one cell line). Replication by RT-qPCR was performed for duplicates of two independent hiMSCs differentiations from another hiPSC line and duplicates of four independent hBMSCs.

### RNA isolation and gene expression analyses

RNA was isolated using the RNAeasy mini kit (Qiagen) according to the manufacturers’ protocol as described previously [17]. Subsequently, total mRNA was outsourced for RNA sequencing by Macrogen using Novaseq 6000 System (Illumina; Additional file 1). Quality control and analyses of generated data was performed with the open-source BioWDL RNAseq pipeline. Details are provided in Additional file 1. Genes were considered differentially expressed (DEGs) upon licensing in comparison to unlicensed controls by a False Discovery Rate (FDR) < 0.05. Replication by RT-qPCR was performed as described [9]. Primer sequences are shown in Additional file 1: Table S1. 

Enrichment for biological pathways and interactions among proteins encoded by identified genes was determined with online tools, respectively, DAVID [18, 19] and STRING [20, 21].

### Secretome protein levels

Concentration of secreted factors in conditioned culture media was determined with multiplex immunoassays (Luminex, BioRad Laboratories) by combining the Bio-Plex Pro Human Cytokine Th1/Th2 Assay, 9-plex (GM-CSF, IL2, IL4, IL5, IL10, IL12/p70, IL13, IFNγ, and TNFα) concurrently with single-plex assays (IL6, IL8/CXCL8, CXCL10, and MCP1/CCL2) according to the manufacturer's protocol. Statistical analysis was done with paired t test in comparison to concentrations for unlicensed control samples.

### Statistics and similarities

RNA sequencing data were corrected for multiple testing to generate a FDR that was considered significant if FDR < 0.05. Similarities between the different cell types were calculated with R statistical language (DESeq2_v.1.30.0 package [22]) based on Pearson correlations and the Jaccard method using normalized and variance-stabilizing transformation (VST) RNA sequencing data. For the mdMLR assay, after confirming normal distribution Shapiro–Wilk, a One-Way ANOVA with the Tukey post hoc test was performed and graphical presentation of MSC-EVs were done with GraphPad version 8.4.3 and mean values ± standard deviations are provided. *P* values < 0.05 were considered statistically significant.

## Results

### hiMSCs are highly comparable to hBMSCs

**Fig. 1:**
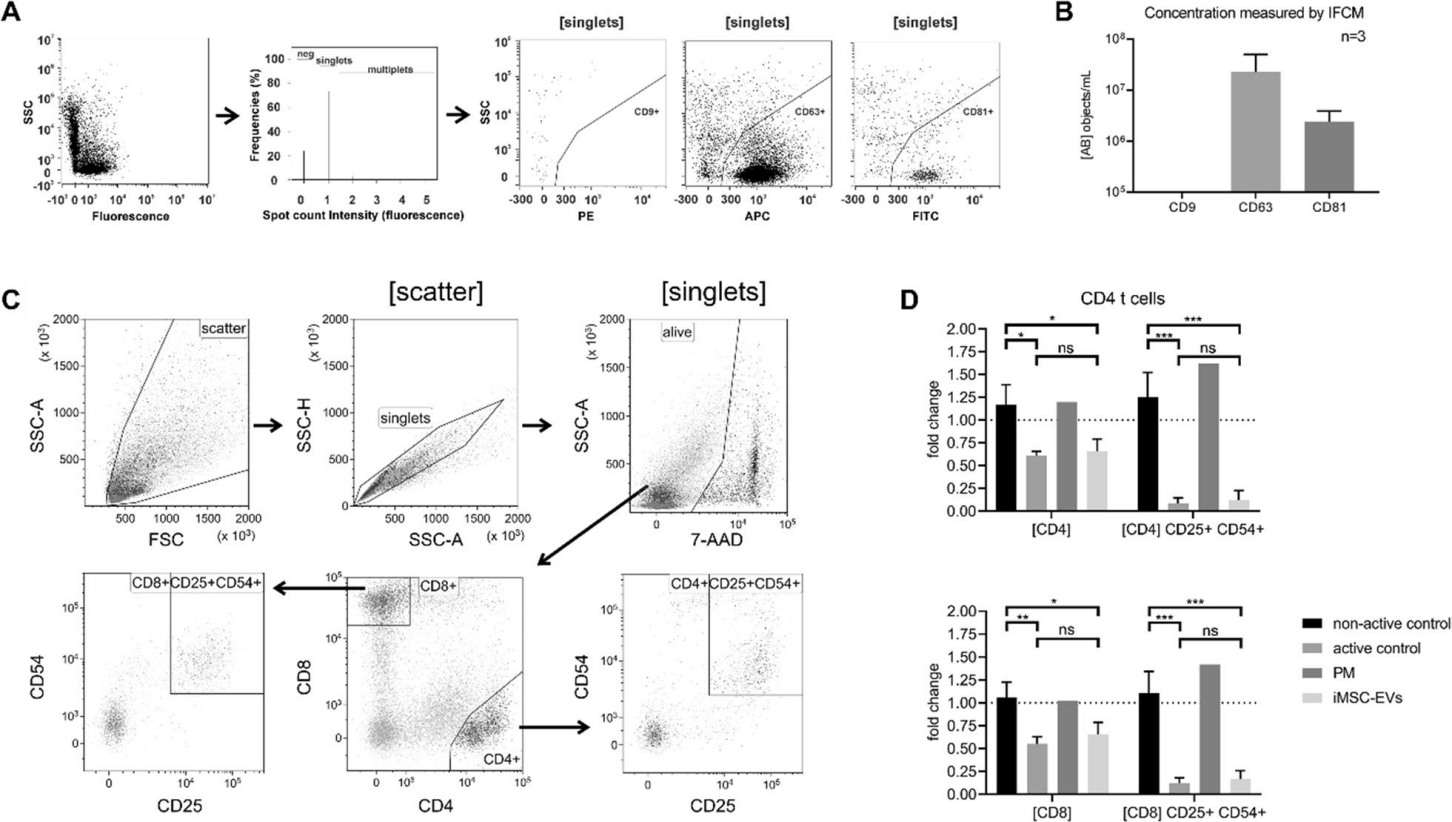
Immunomodulatory capabilities of hiMSC- and hBMSC-EV preparations. (**A**) Representative plots for analysis of CD9, CD63 and CD81 expression on hiMSC-EVs by ImageStreamX Flow Cytometry (IFCM). From all events (1st plot from left), only singlets were selected (2nd plot from left) to plot side scatter (SSC) intensities against the fluorescence intensities of CD9^+^ (PE-labeled), CD63^+^ (APC-labeled) or CD81^+^ (FITC-labeled). (**B**) Average number of CD9^+^, CD63^+^ or CD81^+^ hiMSC-EVs indicated as objects/mL (mean ± SD of three independent EV-preparations). (**C**) Representative plots for analysis of proportion activated lymphocytes by ImageStreamX Flow Cytometry (IFCM). From all events (plot top left), only singlets (plot top middle) and live cells (plot top right) were selected to distinguish CD4^+^ (BV785-labeled) from CD8^+^ (BV650-labeled) cells (plot bottom middle). Subsequently, for both populations fluorescence intensities of CD25^+^ (PE-Cy5.5-labeled) were plotted against CD54^+^ (AF700-labeled; plots bottom right and left, respectively). (**D**) Preparations of hiMSC-EVs (*N* = 3) and respective unconditioned control media (PM) were used in a multi-donor mixed lymphocyte reaction (mdMLR). After 5 days, cells were harvested, labeled with anti-CD4, anti-CD8, anti-CD25, and anti-CD54, and selected as shown. Preparations of hBMSC-EVs with (active control; *N* = 3) or without (non-active control; *N* = 3) immunomodulatory capability were used as controls (graphs show the change compared to untreated controls; *P < 0.05; **P < 0.01; ***P < 0.001)

First, the presence of hiMSC-EVs isolated from collected conditioned media was confirmed with ImageStreamX Flow Cytometry (Fig. 1A, B). Since we recently demonstrated that MSC-EV potency could be more accurately predicted by T cell response as compared to lymphocyte proliferation [23], immunomodulatory capabilities were investigated in a multi donor mixed lymphocyte reaction assay (mdMLR). Following 5 days of culture, either in the presence or absence of EV preparations, the proportions of activated CD4^+^ and CD8^+^ T cells as indicated by the expression of interleukin-2 receptor (CD25) and of intercellular adhesion molecule-1 (CD54; Fig. 1C) showed that addition of control EVs from hBMSCs (active control) significantly reduced CD4^+^ and CD8^+^ T cell activation as compared to the non-active control (~ sixfold reduction; Fig. 1D, CD4/8[CD25^+^ CD54^+^]). Likewise, we observed significantly reduced activation upon addition of hiMSC-EVs (fivefold reduction), while no reduction was observed for the non-active control and the EV-preparations from unconditioned media (PM). Furthermore, there was no significant difference in the proportions of activated CD4^+^ and CD8^+^ T cells between EV preparations from hBMSCs and hiMSCs. This indicated comparable potential therapeutic relevance for hBMSC- and hiMSC-EVs.

### hiMSCs respond similar to licensing as compared to hBMSC

**Fig. 2:**
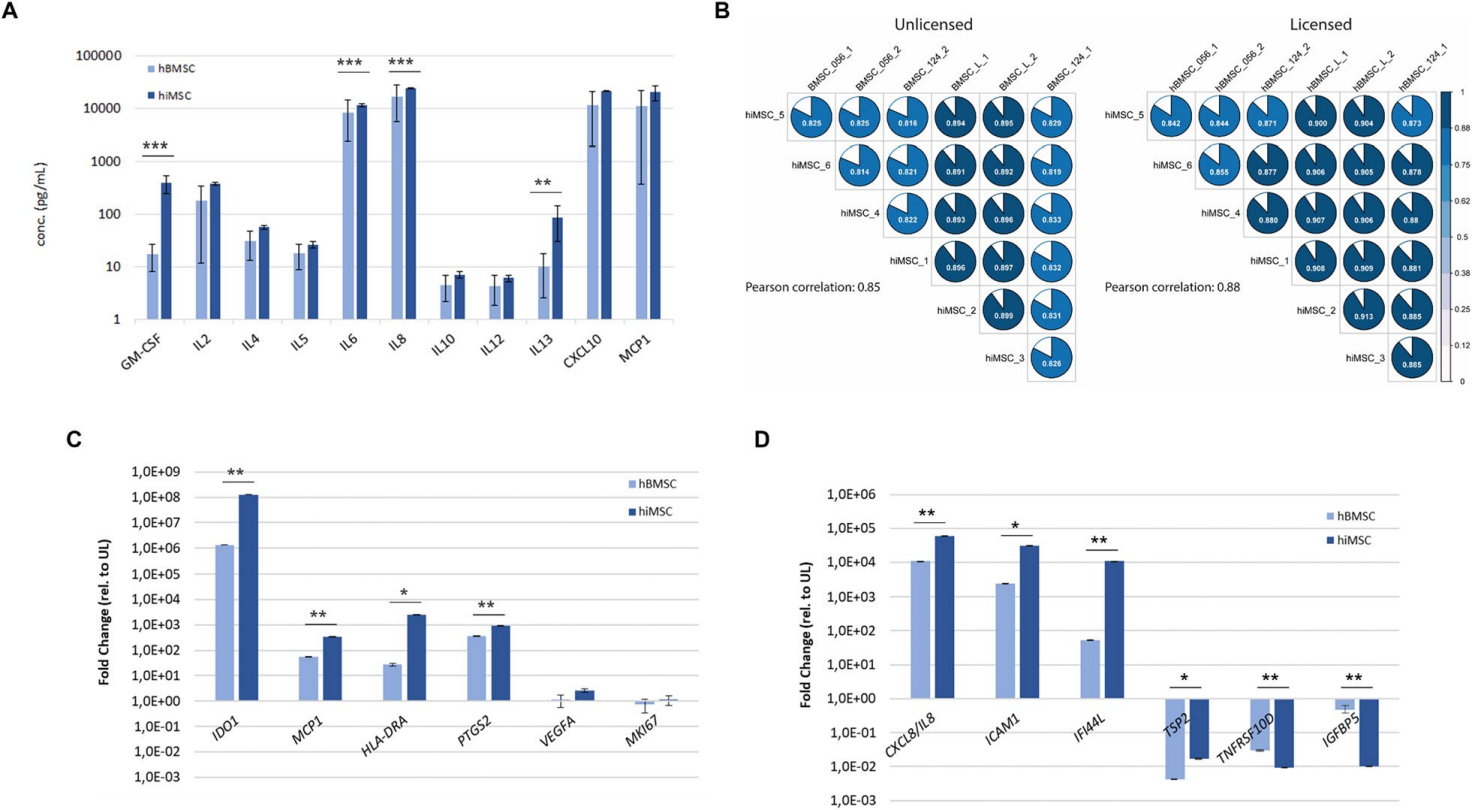
Licensing secretome of hiMSCs and hBMSCs. (**A**) Concentration of well-known immunomodulatory cytokines (pg/mL) in conditioned media upon licensing of hiMSCs or hBMSCs with TNFα and IFNγ. (**B**) Jaccard similarity plots for transcriptome wide Pearson correlations between unlicensed and licensed hMSCs as specified (individual correlations indicated in each pie chart; average correlation across cells indicated left to the plots). (**C**) Fold-changes in expression levels of well-known immunomodulatory genes upon licensing of hiMSCs or hBMSCs. (**D**) Fold-changes in expression levels of FDR significant genes with highest fold up- or downregulation upon licensing of hiMSCs or hBMSCs (significant differences indicated with *P < 0.05; **P < 0.01; ***P < 0.001)

Since the response of hBMSCs to licensing factors TNFα and IFNγ are widely used to predict immunomodulatory potency, we next addressed similarities between hMBSC and hiBMSC in response to TNFα and IFNγ licensing. First, we determined protein concentration of well-known secreted TNFα and IFNγ licensing factors in the culture medium. As illustrated in Fig. 2A (Additional file 1: Fig, S2), the large and significantly increased concentration of secreted factors such as GM-CSF, CXCL8/IL8, and CCL2/MCP1 confirmed response of hiMSCs with immunomodulatory potential. Notably, secretion of GM-CSF, IL6, IL8, and IL13 by the hiMSCs was even higher as compared to that by the hBMSCs.

Next, we performed an in-depth transcriptome wide analysis of the hiMSC secretome as compared to hBMSCs upon combined TNFα and IFNγ licensing. In doing so, we showed that the average Pearson correlation increased from 0.85 to 0.88 while Jaccard similarity index of the overall transcriptome wide expression profiles approached to 1.0 (Fig. 2B and Additional file 1: Fig. S3A).

Results showed in total 6675 genes that significantly changed expression upon licensing with TNFα and IFNγ. Of these genes, 3519 showed increased and 3156 showed decreased expression. Table 1A and B presents the 15 most significant up- and downregulated genes, respectively. Comparison of the hiMSC secretome with the hBMSC secretome showed an overlap of 4780 FDR-significant differentially expressed genes (72%) for the combined TNFα and IFNγ licensing (Additional file 1: Table S2 and Fig. S3B).

**Table 1:** The licensed hiMSC-secretome and enriched biological pathways

A			
	hiMSCs		
Upreglated Genes	*P* value	FDR	FC
*CXCL8/IL8*	0.00	0.00	60,398
*ICAM1*	0.00	0.00	30,931
*IFI44L*	0.00	0.00	10,724
*SAMD9L*	0.00	0.00	7551
*MX1*	0.00	0.00	6487
*GBP1*	0.00	0.00	4186
*IL6*	0.00	0.00	3208
*HLA-DRA*	0.00	0.00	2552
*GCH1*	0.00	0.00	2528
*HLA-B*	0.00	0.00	2257
*APOL6*	0.00	0.00	1689
*CTSS*	0.00	0.00	1011
*TNFAIP3*	0.00	0.00	969
*SERPINB2*	0.00	0.00	934
*C15orf48*	0.00	0.00	793
10^−^			

B			
	hiMSCs		
Downregulated Genes	*P* value	FDR	FC
*TNFRSF10D*	0.00	0.00	-109.1
*THBS2/TSP2*	1.3 × 10^–293^	3.4 × 10^–291^	-57.8
*IGFBP5*	4.2 × 10^–268^	9.5 × 10^–266^	-98.2
*PRPS1*	1.8 × 10^–246^	3.4 × 10^–244^	-30.5
*SFRP1*	2.6 × 10^–237^	4.5 × 10^–235^	-17.8
*LRRC17*	6.8 × 10^–228^	1.1 × 10^–225^	-129.5
*PDE1C*	1.5 × 10^–226^	2.3 × 10^–224^	-31.3
*LAMA4*	2.2 × 10^–195^	2.9 × 10^–193^	-15.6
*DUSP4*	1.5 × 10^–183^	1.9 × 10^–181^	-21.5
*PSD3*	9.8 × 10^–172^	1.1 × 10^–169^	-14.7
*AK5*	5.3 × 10^–157^	5.3 × 10^–155^	-28.7
*SESN3*	6.8 × 10^–136^	6.0 × 10^–134^	-62.8
*GALNT5*	1.6 × 10^–133^	1.4 × 10^–131^	-20.7
*PAMR1*	2.2 × 10^–133^	1.9 × 10^–131^	-53.2
*CPA4*	9.0 × 10^–122^	7.1 × 10^–120^	-20.2

C		
Biological Pathways (upregulated genes)	*P* Value	Fold Enr
GO:0006955 ~ immune response	6.2 × 10^–23^	11.7
GO:0060333 ~ interferon-gamma-mediated signaling pathway	2.4 × 10^–20^	41.3
GO:0006954 ~ inflammatory response	1.2 × 10^–17^	11.1
GO:0071346 ~ cellular response to interferon-gamma	1.5 × 10^–16^	28.2
GO:0030593 ~ neutrophil chemotaxis	3.4 × 10^–16^	32.2
GO:0070098 ~ chemokinx10-mediated signaling pathway	2.2 × 10^–15^	34.6
GO:0051607 ~ defense response to virus	4.9 × 10^–14^	14.2
GO:0060337 ~ type I interferon signaling pathway	8.0 × 10^–14^	32.4
GO:0006935 ~ chemotaxis	9.6 × 10^–14^	21.2
GO:0019882 ~ antigen processing and presentation	2.8 × 10^–12^	39.6
TFBS	PValue	Fold Enr
*NFKAPPAB*	4.4 × 10^–02^	1.3

D		
Biological Pathways (downregulated genes)	*P* Value	Fold Enr
GO:0043406 ~ positive regulation of MAP kinase activity	5.0 × 10^–03^	27.2
GO:0044267 ~ cellular protein metabolic process	1.7 × 10^–02^	14.3
GO:0007507 ~ heart development	3.3 × 10^–02^	10.0
GO:0007155 ~ cell adhesion	3.7 × 10^–02^	5.2
GO:0048565 ~ digestive tract development	3.8 × 10^–02^	49.4
GO:0048863 ~ stem cell differentiation	4.3 × 10^–02^	43.4

TFBS	*P* Value	Fold Enr
*NKX25*	1.45E-05	1.7
*TAL1BETAITF2*	9.37E-05	1.8
*CEBPB*	2.05E-04	1.8
*SOX5*	3.07E-04	1.9
*IRF7*	5.19E-04	1.9
*BRN2*	6.38E-04	1.8
*NKX3A*	7.05E-04	1.9
*POU6F1*	7.51E-04	2.0
*NKX61*	1.00E-03	1.9
*RP58*	1.12E-03	1.7
*LHX3*	1.55E-03	2.0
*NF1*	1.86E-03	2.0
*HOXA3*	2.11E-03	1.9
*HSF2*	2.18E-03	1.9
*SRY*	2.20E-03	1.9
*MEIS1BHOXA9*	2.55E-03	1.7
*CDP*	3.44E-03	1.6
*HNF1*	3.59E-03	1.6
*CART1*	3.67E-03	1.7
*CREBP1*	3.91E-03	1.7
*ISRE*	4.23E-03	1.8
*GATA*	5.33E-03	1.7
*PBX1*	5.86E-03	1.6
*GATA6*	6.08E-03	2.7
*FOXO1*	6.26E-03	1.8
*EVI1*	6.30E-03	1.3
*EN1*	6.32E-03	1.6
*OCT*	7.79E-03	1.7
*LMO2COM*	7.85E-03	1.5
*POU3F2*	9.31E-03	1.5
*AP1*	9.51E-03	1.5
*TBP*	1.03E-02	1.9
*FAC1*	1.07E-02	1.6
*NKX22*	1.39E-02	1.7
*FREAC7*	1.47E-02	1.6
*FOXJ2*	1.51E-02	1.4
*STAT5A*	1.61E-02	1.5
*CDC5*	1.64E-02	1.6
*HTF*	1.85E-02	1.5
*LUN1*	1.85E-02	1.6
*BACH1*	2.03E-02	1.6
*USF*	2.20E-02	1.5
*CEBP*	2.24E-02	1.3
*ER*	2.45E-02	1.5
*MRF2*	2.53E-02	1.5
*ZIC1*	2.66E-02	2.5
*RORA2*	2.75E-02	1.6
*E4BP4*	2.88E-02	1.6
*PAX4*	2.92E-02	1.3
*CHOP*	3.08E-02	1.5
*CEBPA*	3.20E-02	1.9
*GFI1*	3.37E-02	1.6
*TCF11MAFG*	3.41E-02	1.5
*HLF*	3.49E-02	1.6
*MEF2*	3.53E-02	1.3
*OCT1*	3.54E-02	1.2
*AP2REP*	3.65E-02	1.6
*HFH1*	3.69E-02	1.6
*MYB*	4.03E-02	1.5
*CHX10*	4.31E-02	1.6
*HOX13*	4.33E-02	1.5
*NFAT*	4.44E-02	1.6

Top 15 upregulated (A) or downregulated (B) genes. Biological pathways and transcription factor binding sites enriched among the 125 genes with over 1000-fold increase (C) or among the 30 genes with over 50-fold decrease (D). Legend: *FC*: fold change; *Fold Enr*: fold enrichment; *FDR*: False Discovery Rate; *TFBS*: Transcription Factor Binding Sites

Notably, among the 3519 upregulated genes we observed 40 genes with FDR = 0 and 129 genes with over 1000-fold expression level changes upon licensing. Most likely, these genes were turned on or off in response to the licensing. An example is the well-known immunomodulatory gene *IDO1* that showed 1.2 × 10^8^-fold increased expression levels (FDR = 1.2 × 10^–107^). Also, expression levels of *CXCL8/IL8* and *HLA-DRA* increased exceedingly upon licensing with both TNFα and IFNγ (respectively, 6.0 × 10^4^-fold up and 2.6 × 10^3^-fold up, both with FDR = 0). Such changes were found to be highly comparable for hiMSCs and hBMSCs, although the fold changes were not identical (Fig. 2C, D and Additional file 1: Fig. S4). More importantly, the same changes were found for two independent hiMSC lines generated from another hiPSC line (Additional file 1: Fig. S5). Likewise, results for hBMSCs collected from aged OA patients indicated that such cells respond similar to combined TNFα and IFNγ licensing as compared to hBMCs from healthy donors. Together, we demonstrate functionally relevant hiMSC-licensing secretome that has potency hence liability to substitute hBMSCs for therapeutic application.

### Pathway analysis of the hiMSC secretome

**Fig. 3:**
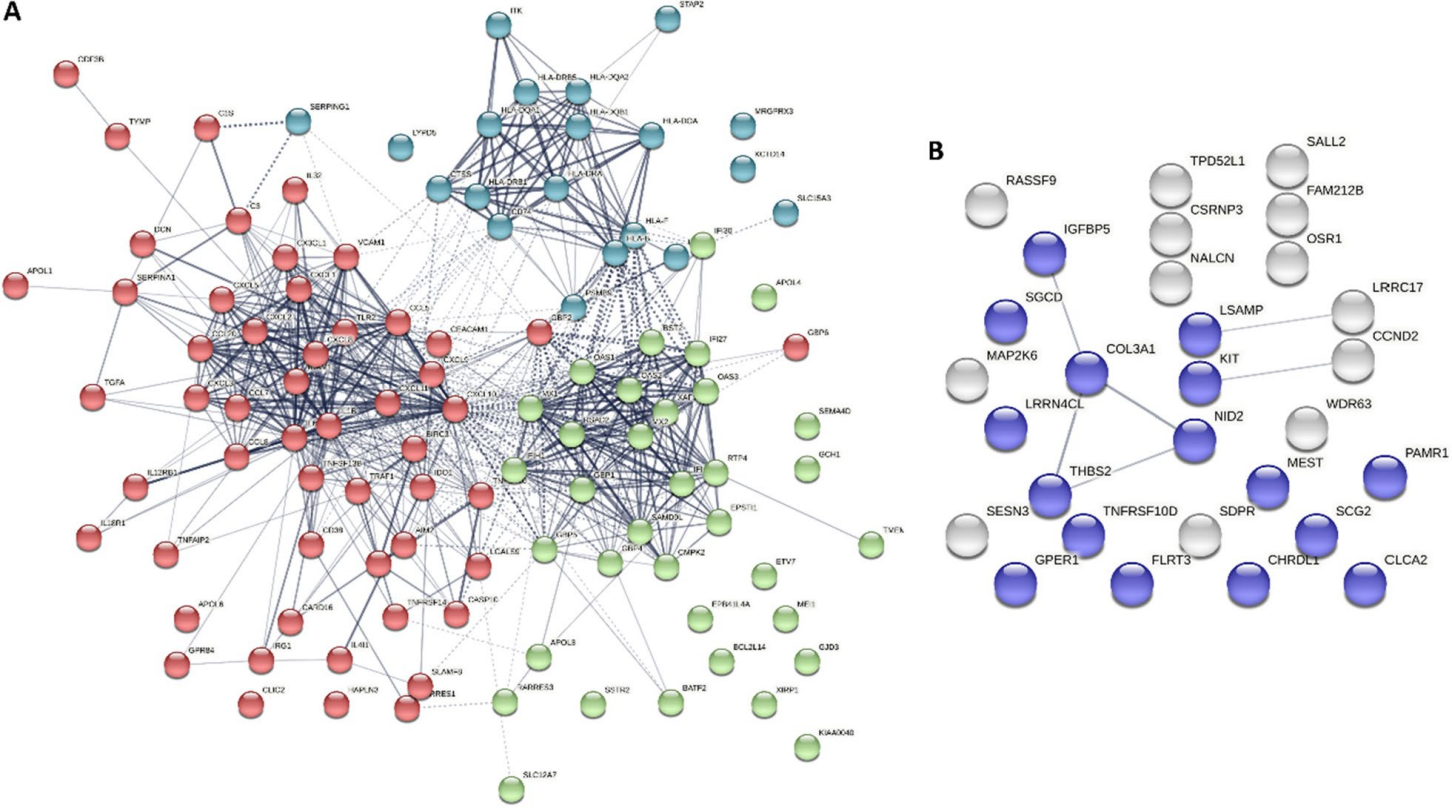
Interaction network for proteins encoded by up- (**A**) and downregulated genes (**B**). Colors indicate different nodes within the network, characterized by connections with HLA-family members exerting MHC class II receptor activity (indicated in blue), CXCL- and CCL-family members exerting chemokine activity (indicated in red), GTP-binding proteins (e.g., MXI and GBP1, indicated in green), or characterized by glycoproteins (indicated in purple)

Pathway analysis of the 125 genes with over 1000-fold increase indicated enrichment of genes involved in immune response (*P* = 6.2 × 10^–23^) and the IFNγ-mediated signaling pathway (*P* = 2.4 × 10^–20^; Table 1C) which is in accordance with the licensing. Additionally, proteins encoded by the 125 extremely upregulated genes showed significant enrichment for protein–protein interactions (*P* < 1.0 × 10^–16^ Fig. 3A). The network revealed three nodes. These nodes were characterized by interactions with HLA-family members exerting MHC class II receptor activity (indicated in blue), CXCL- and CCL-family members exerting chemokine activity (indicated in red), and GTP-binding proteins (e.g., indicated in green; Fig. 3A).

In contrast to the upregulated genes, among 3414 downregulated genes only four genes showed over 1000-fold decrease (*DNAI3*, *OSR1*, *RASSF9*, and *KIT*). A total of 30 genes showed more than 50-fold decrease. Pathway analysis of these 30 genes indicated enrichment for regulation of MAP kinase activity (*P* = 5.0 × 10^–3^) and cellular protein metabolic process (*P* = 3.3 × 10^–2^; Table 1D). There was a significant enrichment for interactions among the proteins encoded by the 30 downregulated genes (*P* = 4.7 × 10^–3^; Fig. 3B). The network was characterized by the presence of multiple glycoproteins (indicated in purple).

Of note was the large difference in enrichment for transcription factor binding sites (TFBSs) in the promoters of identified genes, whereas upregulated genes showed enrichment only for Nuclear Factor kappa-light-chain-enhancer of activated B cells (NFΚB; *P* = 4.4 × 10^–2^; Table 1C), downregulated genes showed enrichment for many different TFBSs (Table 1D). For example, NKX25 (*P* = 1.5 × 10^–5^), CEBPB (*P* = 2.1 × 10^–4^), and SOX5 (*P* = 3.1 × 10^–4^) were amongst the most significant TFBS identified for genes that were downregulated in response to TNFα and IFNγ licensing.

## Discussion & conclusion

In this manuscript, we explored the hiMSC-secretome and -potency. As demonstrated by the gene and protein expression as well as the therapeutically active EVs, our findings for hiMSCs reflect the average response of hBMSCs. Therefore, in contrast to autologous hMSCs that are subject to invasive collection procedure and donor variability, we advocate that hiMSCs may provide a promising “off the shelf,” reproducible and sustainable, cell source for cell therapy or as a tool for the production of clinically relevant EVs.

We found that, at the gene expression level, hiMSCs and hBMCs were highly comparable as denoted by Jaccard similarity index of 0.99 which was even further increased to 1.0 upon licensing with a combination of TNFα and IFNγ. Such uniformization upon licensing of hMSCs was observed before and Pittenger and colleagues [6] suggested that it could be employed to erase donor variability in cell therapy. However, enhanced efficacy of such priming remains to be established. Notably, many genes were extremely upregulated upon licensing (over 1000-fold), while observed decreased expression was typically more modest (10–50 fold). Upregulated genes showed enrichment for functions in immune response and IFNγ-mediated signaling pathways, which is in accordance with the licensing method applied (TNFα and IFNγ).

Given the large numbers of DEGs associated with our licensing conditions, the generated database represents a valuable source for identification of relevant and sensitive potency markers of hiMSCs or hBMSCs. The increased gene expression was in line with increased secretion of immunomodulatory factors and could, as such, be exploited to develop improved assays to indicate hMSCs’ therapeutic potential prior to their use in the clinic [24]. In addition, our data add to understanding the mode of action of regenerative cell-based therapies. For example, upregulated genes were suggested to be regulated by NFΚB signaling. It could be explored whether application of NFΚB-activating or -inhibitory pharmacological compounds could reinforce effects of stem cell therapy.

Taken together, our results indicate that hiMSCs may help to overcome the current limitations of primary hMSCs and products thereof, *e.g.,* their very small batch sizes. Further studies in appropriate preclinical models are warranted to demonstrate therapeutic potential of the hiMSC secretome in addition to the demonstrated immune-suppressive activity of the released hiMSC-EVs in vitro.

## Supplementary Information

**Additional file 1:** Supplementary Materials and Methods, Figures, Tables.

## Data Availability

The data that support the findings of this study are available from the corresponding author upon reasonable request.

## Abbreviations

DEG: Differentially expressed gene
EVs: Extracellular vesicles
FDR: False discovery rate
hBMSC: Human bone marrow-derived mesenchymal stromal cell
hiMSC: HiPSC-derived mesenchymal stromal cell
hiPSC: Human-induced pluripotent stem cell
mdMLR: Multi-donor mixed lymphocyte reaction
MSC: Mesenchymal stromal cell
NFΚB: Nuclear Factor kappa-light-chain-enhancer of activated B cells
TFBS: Transcription factor binding site
