# Supplementary material for: Characterizing the secretome of licensed hiPSC-derived MSCs
Source: Stem Cell Res Ther. 2022 Sep 2;13:434. doi: 10.1186/s13287-022-03117-2 (PMC9438242; doi:10.1186/s13287-022-03117-2)
Supplement: Supplementary file 1 — Additional file 1: Supplementary Materials and Methods, Figures, Tables. [file 13287_2022_3117_MOESM1_ESM.docx]

**SUPPLEMENTARY MATERIAL**

**Supplementary Materials and Methods**

***Maintenance human induced pluripotent stem cells (hiPSCs)***

hiPSCs were maintained under standard conditions (37°C, 5% CO_2_) in TeSR-E8 medium (STEMCELL Technologies) on VitronectinXF-coated plates (STEMCELL Technologies). The medium was refreshed daily and cells were passaged in aggregates using Gentle Cell Dissociation Reagent (STEMCELL Technologies) upon reaching approximately 80% confluency.

***Differentiation of hiPSC towards hiMSCs***

Human iMSCs were generated using the Stemcell Technologies Mesenchymal Progenitor Kit following the manufacturers’ instructions with small modifications as described previously.[1] In short, following three passages using the recommended Mesencult ACF plus medium, cells were further maintaind in DMEM high glucose (Gibco) supplemented with 10% fetal calf serum (FCS; Biowest), basic FGF (bFGF; 5ng/ml; Life Technologies), and antibiotics (100U/ml penicillin, 100μg/ml streptomycin; Gibco) until elongated and with fibroblast-like morphology (**Supplementary Figure S1A**). At passage 5, analyses by Fluorescence-Activated Cell Sorting (FACS) showed that 100% of the cell population expressed the MSC-characteristic cell surface markers CD73 and CD90, and 92% expressed CD105 (**Supplementary Figure S1B**). Trilineage potential of hiMSCs was confirmed (**Supplementary Figure S1C**). All cells were passaged every 4-5 days and showed an average population doubling time of 2±0.5 days.

***RNA isolation and gene expression analyses***

RNA was isolated using the RNAeasy mini kit (Qiagen) according to the manufacturers’ protocol as described previously.[2] Subsequently, total mRNA was further processed for RNA sequencing (500 ng) or for RT-qPCR (200 ng). For RNA sequencing, libraries were prepared (Truseq strand total RNA with Ribo-zero, Illumina) and 40M paired end reads were generated by [Macrogen](https://www.macrogen-europe.com/) using Novaseq 6000 System (Illumina). RNA-Seq reads were processed using the open-source [BIOWDL RNAseq pipeline v5.0.0](https://zenodo.org/record/5109461#.Ya2yLFPMJhE) developed at the LUMC. This pipeline performs FASTQ preprocessing (including quality control, quality trimming, and adapter clipping), RNA-Seq alignment, UMI based deduplication, read quantification, and optionally transcript assembly. FastQC (v0.11.9) was used for checking raw read QC. Adapter clipping was performed using Cutadapt (v2.10) with default settings. RNA-Seq reads’ alignment was performed using STAR (v2.7.5a) on GRCh38 human reference genome. The gene read quantification was performed using HTSeq-count (v0.12.4) with setting “–stranded=yes”. The gene annotation used for quantification was Ensembl version 104.

Read counts were processed and analyzed with R v4.1.0 while removing low expressed genes (genes with a Counts Per Million (CPM) value smaller than 1 in more than 50% of the samples). Following this filtering, the remaining well-expressed genes (~12K) were analyzed for differential expression using the DESeq2 package (v1.32.0). Benjamini-Hochberg multiple testing was applied to calculate False Discovery Rates (FDR). Differentially expressed genes (DEGs) in licensed compared to unlicensed controls were reported with a significance cut-off of FDR<0.05.

Similarities between the hMSCs were determined in a pairwise manner using both the Cosine index method and Pearson correlation. To report the similarities between all hMSCs together, the average of the Cosine indices or of the Pearson correlation coefficients were calculated.

***Preparation and characterization of extracellular vesicles from MSC-conditioned cell culture media***

Conditioned media from hiMSCs were harvested 48hr after refreshment. Extracellular vesicles (EVs) were prepared from unconditioned, plain medium (PM), or conditioned medium by polyethylene glycol 6000 precipitation followed by ultracentrifugation, as described previously.[3-5] Obtained EV preparations were diluted in NaCl-HEPES buffer (Sigma-Aldrich) such that 1 mL of final samples contained the preparation yield of the conditioned media of approximately 1.6x10^8^ cells and stored on -80°C until usage.

EV preparations were characterized according to the MISEV criteria.[6] To that end, average particle concentrations were determined by NTA on a ZetaView PMX-120 platform equipped with the software version 8.03.08.02 (ParticleMetrix, Meerbusch, Germany).[4] Protein concentration was determined by bicinchoninic acid (BCA) assay (Pierce, Rockford, IL, USA) in 96-well plates according to the manufacturer’s recommendations. Presence of EV-specific markers (CD9, CD63, and CD81) were confirmed by ImageStreamX Flow Cytometry (IFCM; **Figure 1A-B**).

***Multi-donor mixed lymphocyte reaction (mdMLR)***

The immunomodulatory potential of EV preparations from conditioned media was determined in a multi-donor mixed lymphocyte reaction assay (MLR) as described previously.[7] Ficoll-prepared peripheral blood mononuclear cells (PBMC) of 12 healthy donors were mixed in equal proportions, aliquoted and stored in the gas-phase of liquid nitrogen until usage. After thawing, 600,000 cells were seeded in 200 µL medium (RPMI 1640 (Thermo Fisher Scientific) supplemented with 10% human AB serum (produced in house) and antibiotics (100 U/mL penicillin and 100 µg/mL streptomycin; Thermo Fisher Scientific)) per well of a 96-well U-bottom shape plate (Corning), either in the presence or absence of EVs. Three independent preparations of hiMSC-EVs and PM were added to the cells. Preparations of BMSC-EVs with and without immunomodulatory capabilities were used as respectively positive and negative controls. After 5 days, cells were harvested, stained with different fluorescent labelled antibodies (CD4-BV785; BioLegend, San Diego, CA, USA; CD8-BV650; BioLegend; CD25-PE-Cy5.5; BD Bioscience; and CD54-AF700; EXBIO) and analysed on a Cytoflex flow cytometer (Software CytExpert 2.3, Beckman-Coulter). Activated and non-activated CD4+ and CD8+ T cells were discriminated by means of their CD25 and CD54 expression, respectively. Typically, 5 µL of EV preparations were applied. The following antibodies were used to further discriminate subpopulations: CD14-PO (EXBIO), CD19-ECD (Beckman Coulter) and CD56-APC (BioLegend). Data was analyzed with the Kaluza software (Version 2.1, Beckman Coulter). For statistical analyses of differences, first we confirmed normal distribution of the data (Shapiro-Wilk for 3 datasets each). Subsequently, a one-way ANOVA was performed with the Tukey posthoc test.

**References**

1. Rodriguez Ruiz A, Dicks A, Tuerlings M, Schepers K, van Pel M, Nelissen R, Freund C, Mummery CL, Orlova V, Guilak F *et al*: **Cartilage from human-induced pluripotent stem cells: comparison with neo-cartilage from chondrocytes and bone marrow mesenchymal stromal cells**. *Cell Tissue Res* 2021.

2. Bomer N, den Hollander W, Ramos YF, Bos SD, van der Breggen R, Lakenberg N, Pepers BA, van Eeden AE, Darvishan A, Tobi EW *et al*: **Underlying molecular mechanisms of DIO2 susceptibility in symptomatic osteoarthritis**. *Ann Rheum Dis* 2015, **74**(8):1571-1579.

3. Kordelas L, Rebmann V, Ludwig AK, Radtke S, Ruesing J, Doeppner TR, Epple M, Horn PA, Beelen DW, Giebel B: **MSC-derived exosomes: a novel tool to treat therapy-refractory graft-versus-host disease**. *Leukemia* 2014, **28**(4):970-973.

4. Ludwig AK, De Miroschedji K, Doeppner TR, Borger V, Ruesing J, Rebmann V, Durst S, Jansen S, Bremer M, Behrmann E *et al*: **Precipitation with polyethylene glycol followed by washing and pelleting by ultracentrifugation enriches extracellular vesicles from tissue culture supernatants in small and large scales**. *J Extracell Vesicles* 2018, **7**(1):1528109.

5. Borger V, Staubach S, Dittrich R, Stambouli O, Giebel B: **Scaled Isolation of Mesenchymal Stem/Stromal Cell-Derived Extracellular Vesicles**. *Curr Protoc Stem Cell Biol* 2020, **55**(1):e128.

6. Thery C, Witwer KW, Aikawa E, Alcaraz MJ, Anderson JD, Andriantsitohaina R, Antoniou A, Arab T, Archer F, Atkin-Smith GK *et al*: **Minimal information for studies of extracellular vesicles 2018 (MISEV2018): a position statement of the International Society for Extracellular Vesicles and update of the MISEV2014 guidelines**. *J Extracell Vesicles* 2018, **7**(1):1535750.

7. Tertel T, Schoppet M, Stambouli O, Al-Jipouri A, James PF, Giebel B: **Imaging flow cytometry challenges the usefulness of classically used extracellular vesicle labeling dyes and qualifies the novel dye Exoria for the labeling of mesenchymal stromal cell-extracellular vesicle preparations**. *Cytotherapy* 2022.

**Supplementary Figures**

**Supplementary Figure S1. Characterization generated hiMSCs.**


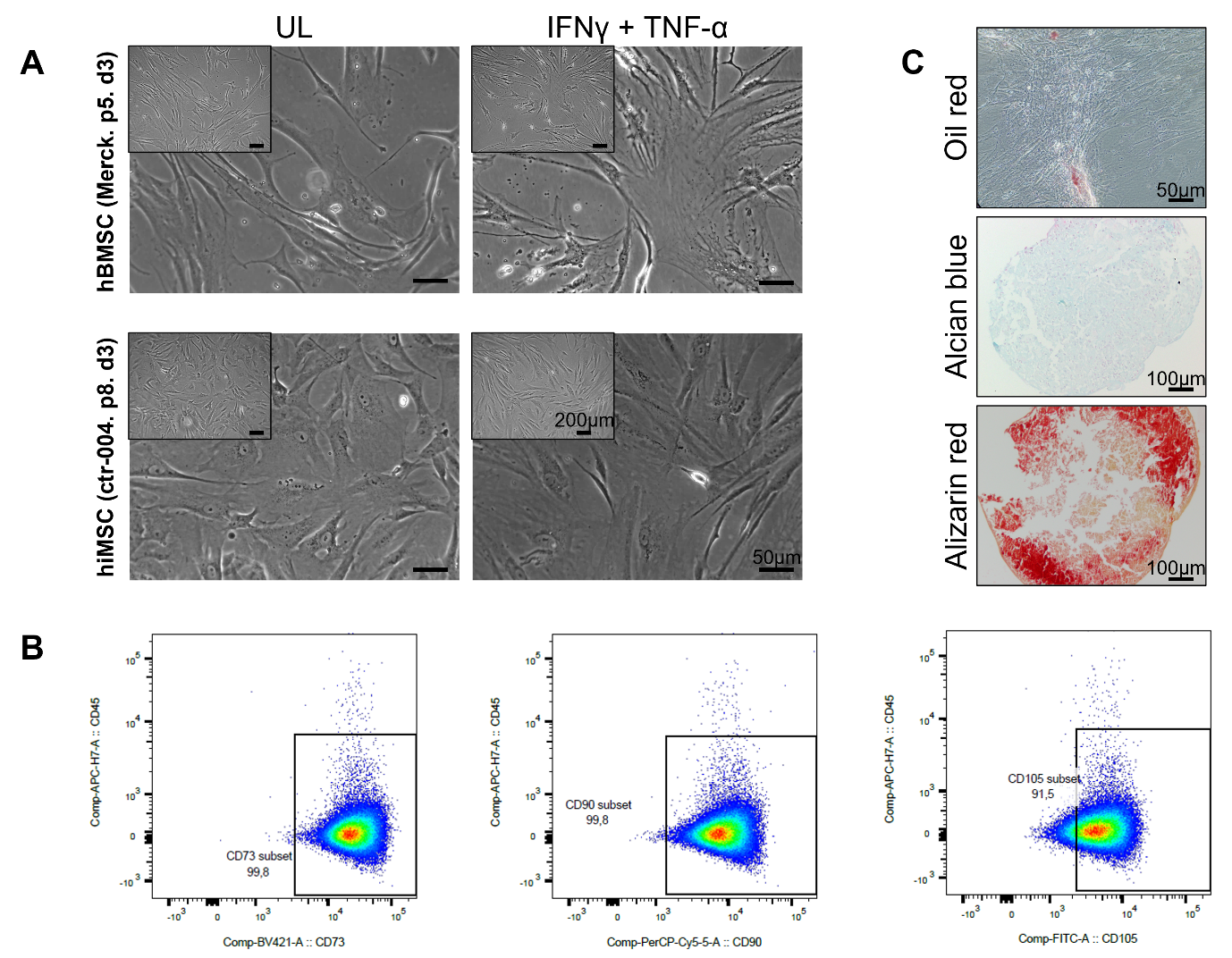


**A)** Representative image of unlicensed (UL) and licensed cells as indicated (scale bar: 50μm and 200μm, inset). **B)** Human iMSCs were analyzed by FACS to determine percentage of cells positive for CD73 (left panel; BV421-labeled), CD90 (middle panel; PerCP-Cy5-labeled) or CD105 (right panel; FITC-labeled), characteristic for hBMSCs. **C)** Multidirectional differentiations (adipogenesis, chondrogenesis and osteogenesis) were performed and visualized (oil red, Alcian blue, Alizarin red staining).

**Supplementary Figure S2. Cytokine concentrations in conditioned media.**


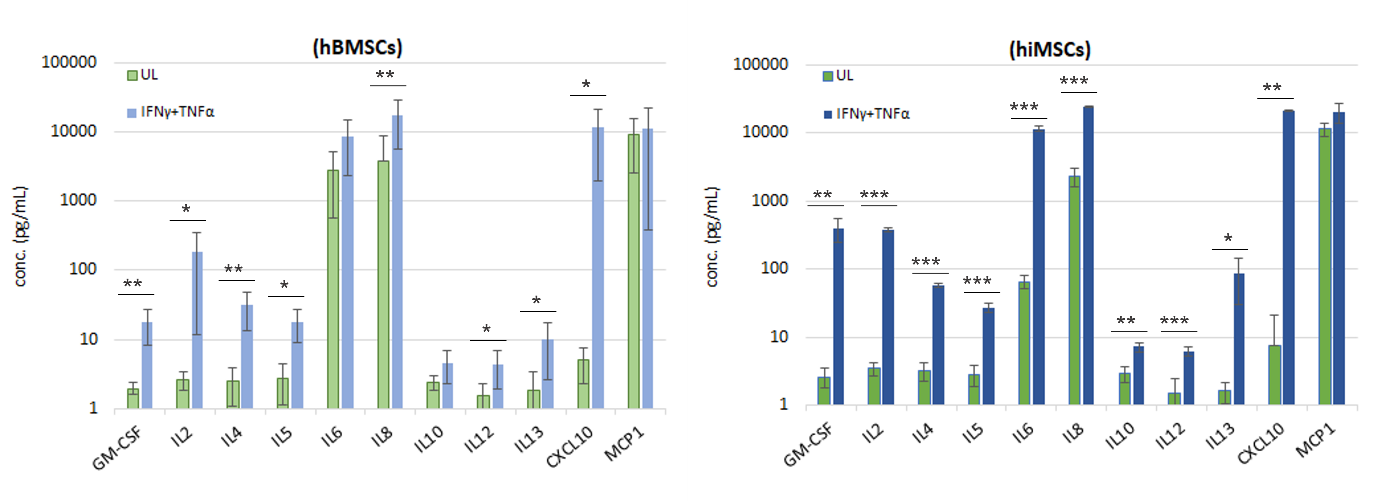


Concentrations of cytokines as indicated (pg/mL), determined with immunoassays in conditioned media from hBMSCs (N=6; **left panel**) or from hiMSCs (N=5; **right panel**). In green: media from unlicensed cells; in blue: media from licensed cells (* *P*<0.05; ** *P*<0.01; *** *P*<0.001).

**Supplementary Figure S3. Comparison of hBMSCs and hiMSCs by RNA sequencing.**


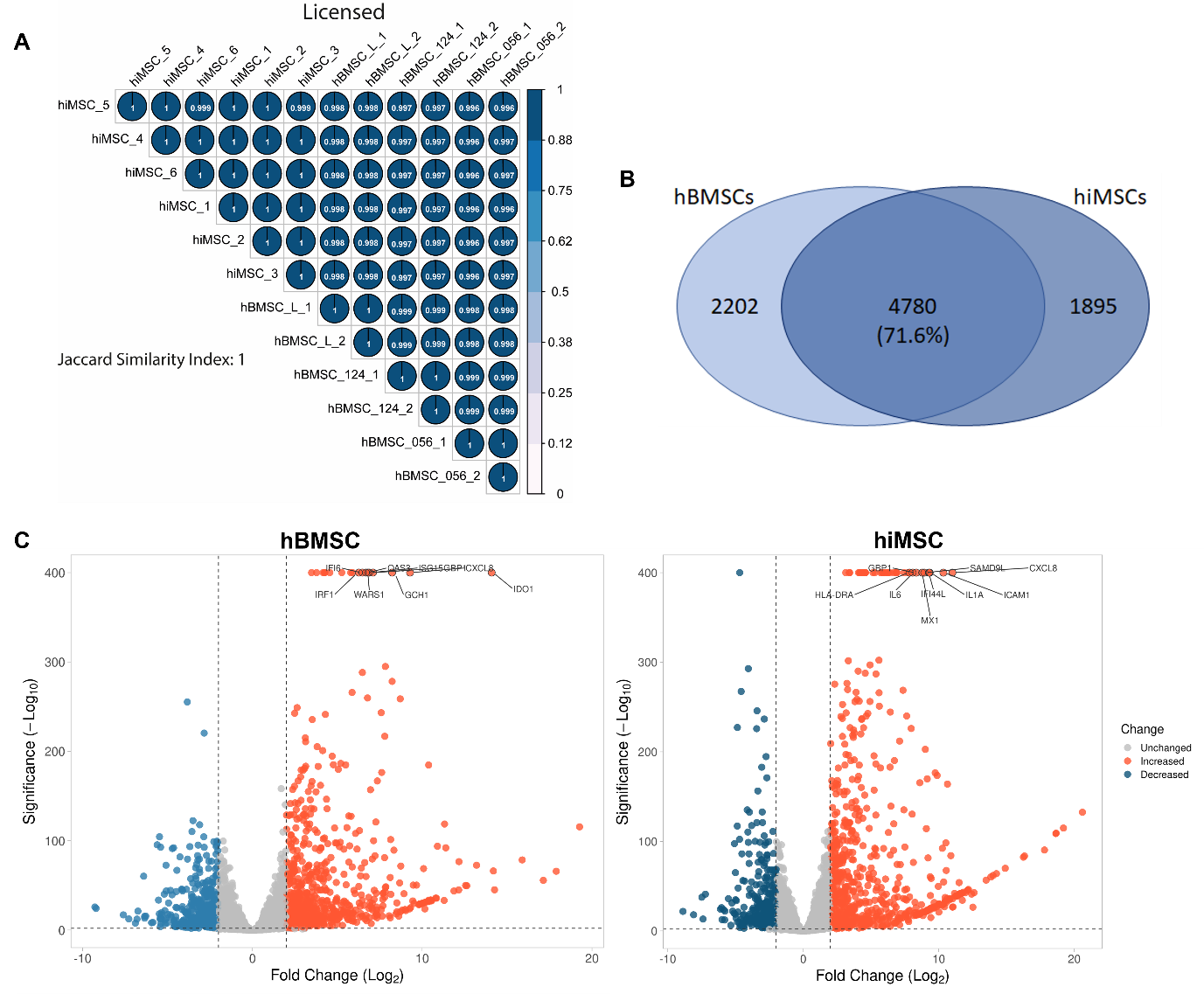


**A)** Jaccard similarities between hMSCs upon licensing with combined TNFα and IFNγ (average Jaccard similarity index indicated left). **B)** Venn diagram of FDR significant genes upon licensing of hiMSCs or hBMSCs. **C)** Volcano plot for gene expression changes following licensing of hBMSCs (left plot) and hiMSCs (right plot). In blue: significant decreased genes; in red: significant increased genes.

**Supplementary Figure S4. Gene expression changes upon licensing of hMSCs with TNFα and IFNγ.**


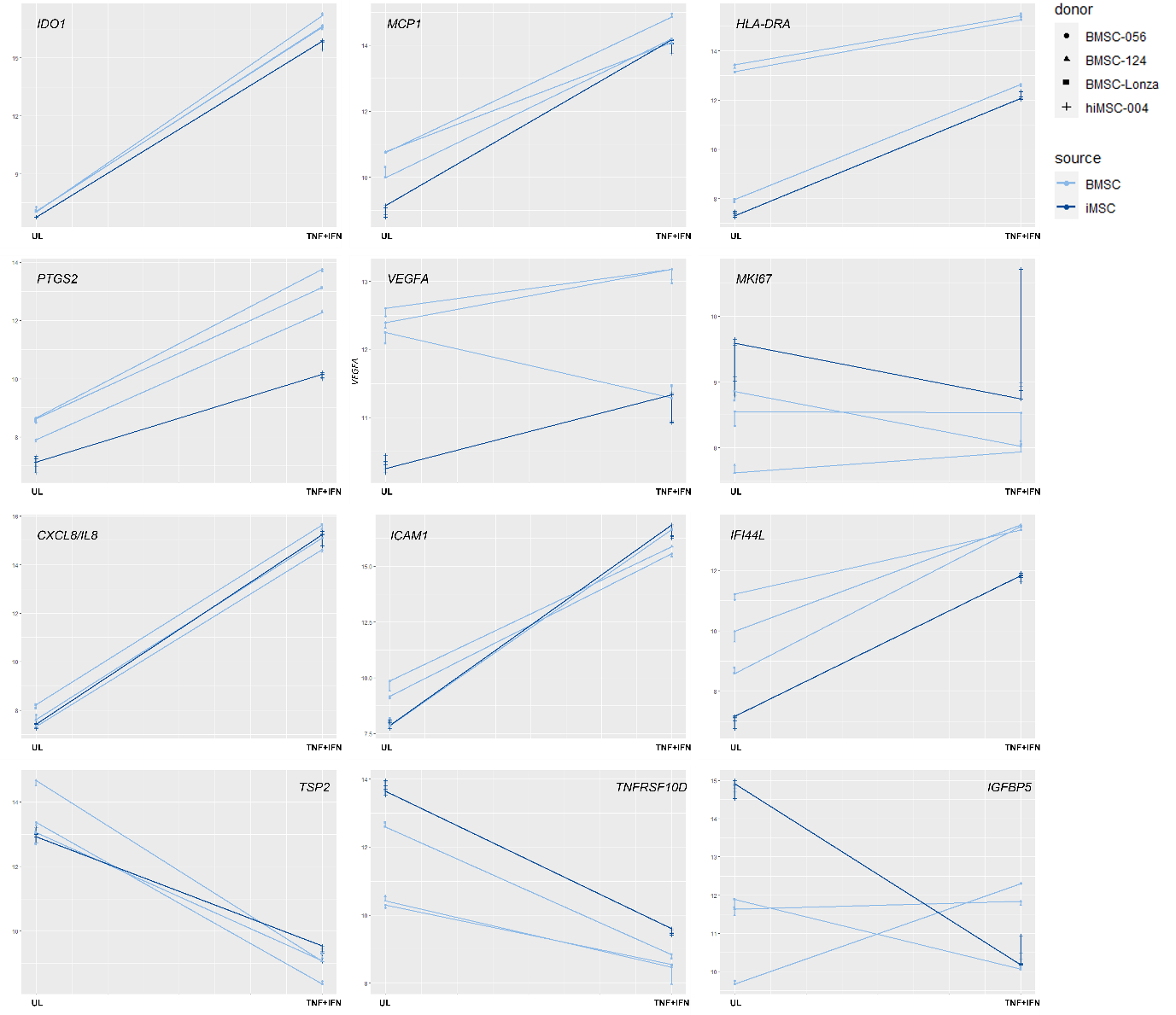


Line plots for hBMSCs (light blue; biological duplicates for three independent donors) and hiMSCs (dark blue; six biological replicates for one cell line). Geometric mean of gene expression per cell line are connected for unlicensed (UL) and licensed cells (TNF+IFN; y-axis: VST-transformed values of RNA sequencing).

**Supplementary Figure S5. Replication of RNA sequencing results by RT-qPCR.**


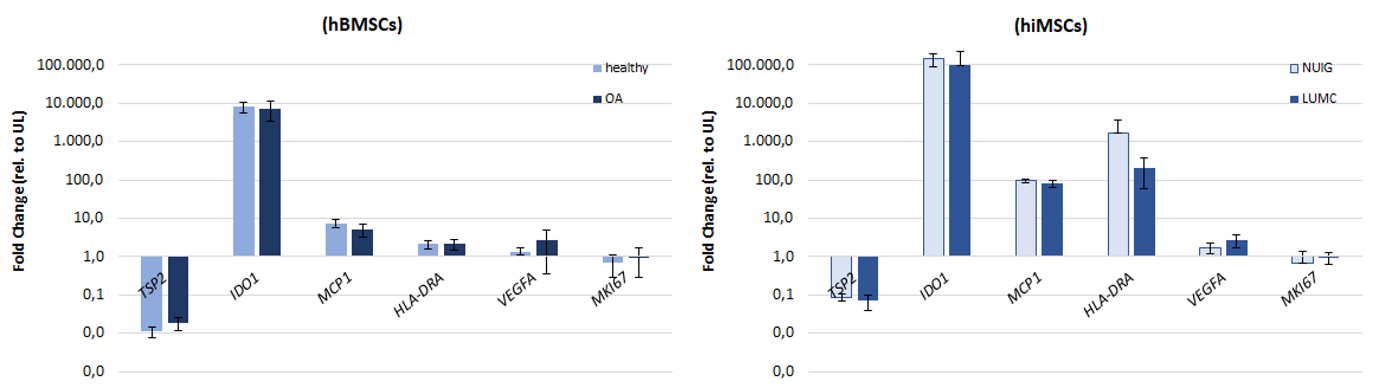


RT-qPCR for differentially expressed genes in response to licensing with TNFα and IFNγ. **Left panel:** hBMSCs from healthy donors (light blue; biological duplicates for N=2 donors) or from OA patients undergoing joint replacement surgery (dark blue; biological duplicates for N=4 donors). **Right panel:** hiMSCs generated at NUIG (light blue; biological duplicates for N=2 differentiations of an independent hiPSC line) or at LUMC (dark blue; 3 independent biological duplicates for N=1 differentiation for hiPSC-004).

**Supplementary Table S1.** Sequence of primers used in RT-qPCR.

|  | **primers** | |
| --- | --- | --- |
| **Gene** | **Fw** | **Rv** |
| *CCL2/MCP1* | 5'-ATCAATGCCCCAGTCACCTG-3' | 5'-TCTCCTTGGCCACAATGGTC-3' |
| *GAPDH* | 5'-TGCCATGTAGACCCCTTGAAG-3' | 5'-ATGGTACATGACAAGGTGCGG-3' |
| *HLA-DRA* | 5'-GTGTCAGAGACAGTCTTCCT-3' | 5'-ACCACGTTCTCTGTAGTCTC-3' |
| *IDO1* | 5'-GGCCAGCTTCGAGAAAGAGT-3' | 5'-TTGCCCCACACATATGCCAT-3' |
| *MKI67* | 5'-ACACATTGTCCTCAGCCTTCT-3' | 5'-GTGCCTGAGATCAAGAAAGACA-3' |
| *SDHA* | 5'-TGGGAACAAGAGGGCATCTG-3' | 5'-GCCTACCACCACTGCATCAA-3' |
| *TSP2* | 5'-AAAGAGCCGGATGTACGTGG-3' | 5'-CTTGCTGGCAACCCTTCTTG-3' |
| *VEGFA* | 5'-TCACCAAGGCCAGCACATAG-3' | 5'-TTGCTCTATCTTTCTTTGGTCTGC-3' |

**Supplementary Table S2.** Differentially Expressed Genes with FDR<10^-5^ in licensed hiMSCs (in grey, overlap with FDR significant genes for hBMSCs).

|  | **hiMSCs** | | |  | **hBMSCs** | | |
| --- | --- | --- | --- | --- | --- | --- | --- |
| **Gene** | **Pvalue** | **FDR** | **FC** |  | **FC** | **Pvalue** | **FDR** |
| *CXCL8/IL8* | 0,00E+00 | 0,00E+00 | 60398,3 |  | 10744,8 | 0,00E+00 | 0,00E+00 |
| *ICAM1* | 0,00E+00 | 0,00E+00 | 30930,9 |  | 2508,5 | 2,54E-48 | 8,97E-47 |
| *IFI44L* | 0,00E+00 | 0,00E+00 | 10723,6 |  | 53,4 | 7,78E-19 | 7,72E-18 |
| *SAMD9L* | 0,00E+00 | 0,00E+00 | 7551,5 |  | 41,3 | 1,92E-155 | 3,94E-153 |
| *MX1* | 0,00E+00 | 0,00E+00 | 6487,1 |  | 1565,1 | 1,38E-167 | 3,33E-165 |
| *GBP1* | 0,00E+00 | 0,00E+00 | 4186,4 |  | 1213,4 | 0,00E+00 | 0,00E+00 |
| *IL6* | 0,00E+00 | 0,00E+00 | 3207,7 |  | 108,5 | 2,52E-195 | 8,36E-193 |
| *HLA-DRA* | 0,00E+00 | 0,00E+00 | 2551,6 |  | 27,3 | 6,94E-08 | 2,78E-07 |
| *GCH1* | 0,00E+00 | 0,00E+00 | 2527,9 |  | 3747,2 | 0,00E+00 | 0,00E+00 |
| *HLA-B* | 0,00E+00 | 0,00E+00 | 2256,7 |  | 57,0 | 3,73E-73 | 2,29E-71 |
| *APOL6* | 0,00E+00 | 0,00E+00 | 1689,4 |  | 26,9 | 5,69E-12 | 3,40E-11 |
| *CTSS* | 0,00E+00 | 0,00E+00 | 1011,3 |  | 1466,9 | 6,45E-42 | 1,87E-40 |
| *TNFAIP3* | 0,00E+00 | 0,00E+00 | 968,9 |  | 218,8 | 3,12E-22 | 3,73E-21 |
| *SERPINB2* | 0,00E+00 | 0,00E+00 | 934,4 |  | 1571,2 | 1,43E-17 | 1,29E-16 |
| *C15orf48* | 0,00E+00 | 0,00E+00 | 793,4 |  | 5841,7 | 1,56E-53 | 6,14E-52 |
| *SOD2* | 0,00E+00 | 0,00E+00 | 597,6 |  | 139,1 | 5,95E-35 | 1,34E-33 |
| *IFI6* | 0,00E+00 | 0,00E+00 | 519,1 |  | 648,6 | 0,00E+00 | 0,00E+00 |
| *TAP1* | 0,00E+00 | 0,00E+00 | 515,0 |  | 325,5 | 0,00E+00 | 0,00E+00 |
| *IRF1* | 0,00E+00 | 0,00E+00 | 470,7 |  | 524,9 | 0,00E+00 | 0,00E+00 |
| *WARS1* | 0,00E+00 | 0,00E+00 | 411,5 |  | 897,6 | 0,00E+00 | 0,00E+00 |
| *TFPI2* | 0,00E+00 | 0,00E+00 | 356,7 |  | 110,4 | 8,50E-13 | 5,40E-12 |
| *CFH* | 0,00E+00 | 0,00E+00 | 351,2 |  | 4,4 | 4,40E-11 | 2,41E-10 |
| *CCL2/MCP1* | 0,00E+00 | 0,00E+00 | 341,1 |  | 54,0 | 1,08E-162 | 2,49E-160 |
| *IFIT3* | 0,00E+00 | 0,00E+00 | 304,8 |  | 647,8 | 5,52E-289 | 3,39E-286 |
| *PARP14* | 0,00E+00 | 0,00E+00 | 196,4 |  | 58,3 | 1,56E-45 | 5,11E-44 |
| *HLA-A* | 0,00E+00 | 0,00E+00 | 172,0 |  | 32,9 | 2,84E-236 | 1,16E-233 |
| *APOL2* | 0,00E+00 | 0,00E+00 | 98,3 |  | 63,4 | 0,00E+00 | 0,00E+00 |
| *IFIT2* | 0,00E+00 | 0,00E+00 | 98,1 |  | 3436,2 | 4,99E-102 | 5,43E-100 |
| *B2M* | 0,00E+00 | 0,00E+00 | 97,1 |  | 18,9 | 1,22E-179 | 3,18E-177 |
| *STAT1* | 0,00E+00 | 0,00E+00 | 86,4 |  | 20,8 | 1,05E-181 | 2,92E-179 |
| *DTX3L* | 0,00E+00 | 0,00E+00 | 79,5 |  | 22,5 | 3,86E-42 | 1,13E-40 |
| *NLRC5* | 0,00E+00 | 0,00E+00 | 76,3 |  | 12,8 | 7,16E-125 | 1,05E-122 |
| *DDX60L* | 0,00E+00 | 0,00E+00 | 67,6 |  | 30,4 | 1,13E-58 | 5,02E-57 |
| *SAMHD1* | 0,00E+00 | 0,00E+00 | 67,2 |  | 22,7 | 1,12E-30 | 2,04E-29 |
| *HLA-C* | 0,00E+00 | 0,00E+00 | 64,0 |  | 22,6 | 2,44E-128 | 3,70E-126 |
| *LAP3* | 0,00E+00 | 0,00E+00 | 59,1 |  | 70,2 | 0,00E+00 | 0,00E+00 |
| *GOLM1* | 0,00E+00 | 0,00E+00 | 30,6 |  | 12,5 | 2,82E-30 | 5,00E-29 |
| *ERAP1* | 0,00E+00 | 0,00E+00 | 29,9 |  | 6,1 | 9,66E-40 | 2,57E-38 |
| *HLA-E* | 0,00E+00 | 0,00E+00 | 22,9 |  | 13,6 | 1,50E-142 | 2,83E-140 |
| *TNFRSF10D* | 0,00E+00 | 0,00E+00 | -109,1 |  | -33,1 | 4,08E-34 | 8,80E-33 |
| *TNFRSF1B* | 5,55E-303 | 1,59E-300 | 266,3 |  | 21,6 | 2,11E-136 | 3,70E-134 |
| *ARNTL2* | 2,46E-302 | 6,89E-300 | 27,9 |  | 7,4 | 8,51E-25 | 1,17E-23 |
| *EREG* | 1,04E-297 | 2,86E-295 | 137,9 |  | 72,2 | 2,40E-17 | 2,12E-16 |
| *THBS2* | 1,27E-293 | 3,41E-291 | -57,8 |  | -239,0 | 3,63E-105 | 4,17E-103 |
| *MAFF* | 8,20E-291 | 2,15E-288 | 57,4 |  | 18,9 | 8,10E-96 | 7,59E-94 |
| *C1QTNF1* | 1,61E-288 | 4,14E-286 | 96,6 |  | 68,2 | 2,29E-25 | 3,28E-24 |
| *CD274* | 1,24E-287 | 3,12E-285 | 213,4 |  | 2023,1 | 4,30E-177 | 1,10E-174 |
| *IL7R* | 3,71E-277 | 9,15E-275 | 25,1 |  | 594,8 | 4,85E-19 | 4,86E-18 |
| *STAT2* | 3,04E-276 | 7,33E-274 | 10,2 |  | 4,9 | 3,07E-19 | 3,14E-18 |
| *ATP2B1* | 3,56E-270 | 8,43E-268 | 26,4 |  | 5,6 | 1,03E-29 | 1,80E-28 |
| *HAPLN3* | 2,05E-269 | 4,76E-267 | 1574,0 |  | 167,5 | 4,98E-94 | 4,49E-92 |
| *IGFBP5* | 4,18E-268 | 9,52E-266 | -98,2 |  | 0,5 | 5,39E-01 | 6,07E-01 |
| *TNIP1* | 3,73E-267 | 8,34E-265 | 49,3 |  | 47,0 | 6,45E-184 | 1,84E-181 |
| *IFI35* | 1,18E-266 | 2,59E-264 | 268,7 |  | 142,2 | 6,10E-98 | 6,09E-96 |
| *BTN3A2* | 2,42E-259 | 5,21E-257 | 52,7 |  | 7,6 | 1,02E-70 | 6,04E-69 |
| *BTN3A1* | 2,80E-257 | 5,93E-255 | 61,9 |  | 30,5 | 3,95E-162 | 8,98E-160 |
| *GSAP* | 6,79E-257 | 1,41E-254 | 137,5 |  | 28,2 | 2,36E-61 | 1,10E-59 |
| *INHBA* | 8,91E-257 | 1,82E-254 | 45,4 |  | -2,1 | 7,77E-01 | 8,18E-01 |
| *RIPK2* | 1,49E-253 | 3,00E-251 | 18,1 |  | 39,3 | 9,59E-113 | 1,20E-110 |
| *IFI44* | 1,18E-251 | 2,33E-249 | 273,7 |  | 10,0 | 9,14E-25 | 1,25E-23 |
| *PRPS1* | 1,75E-246 | 3,40E-244 | -30,5 |  | -2,5 | 2,06E-01 | 2,68E-01 |
| *HLA-DPA1* | 4,24E-245 | 8,13E-243 | 616,7 |  | 15,5 | 2,48E-11 | 1,40E-10 |
| *LIMK2* | 3,60E-244 | 6,78E-242 | 23,6 |  | 28,2 | 2,08E-119 | 2,91E-117 |
| *PARP9* | 1,58E-243 | 2,94E-241 | 116,8 |  | 25,6 | 8,70E-33 | 1,76E-31 |
| *TNFAIP2* | 9,83E-241 | 1,80E-238 | 2059,6 |  | 56,4 | 1,62E-92 | 1,39E-90 |
| *HERC6* | 1,30E-240 | 2,34E-238 | 103,3 |  | 137,3 | 1,51E-32 | 3,04E-31 |
| *ETS1* | 5,21E-240 | 9,25E-238 | 16,1 |  | 6,1 | 2,53E-18 | 2,42E-17 |
| *RALA* | 5,47E-238 | 9,57E-236 | 17,9 |  | 1,1 | 5,33E-06 | 1,69E-05 |
| *ZC3H12A* | 1,17E-237 | 2,01E-235 | 75,1 |  | 80,0 | 1,92E-136 | 3,42E-134 |
| *SFRP1* | 2,63E-237 | 4,47E-235 | -17,8 |  | -7,6 | 6,05E-83 | 4,59E-81 |
| *UBE2L6* | 6,67E-237 | 1,12E-234 | 74,2 |  | 16,6 | 6,09E-51 | 2,25E-49 |
| *LRRC17* | 6,77E-228 | 1,12E-225 | -129,5 |  | -124,3 | 9,16E-18 | 8,40E-17 |
| *NAMPT* | 1,23E-227 | 2,01E-225 | 31,8 |  | 26,8 | 1,48E-114 | 1,89E-112 |
| *DDX60* | 1,33E-227 | 2,14E-225 | 85,4 |  | 25,1 | 5,81E-34 | 1,24E-32 |
| *PDE1C* | 1,49E-226 | 2,33E-224 | -31,3 |  | -9,5 | 1,31E-30 | 2,36E-29 |
| *VEGFC* | 4,07E-225 | 6,30E-223 | 41,0 |  | 10,4 | 4,02E-32 | 7,92E-31 |
| *JUNB* | 9,48E-222 | 1,45E-219 | 43,5 |  | 13,5 | 3,34E-54 | 1,34E-52 |
| *SQOR* | 1,32E-220 | 1,99E-218 | 70,1 |  | 12,2 | 1,34E-135 | 2,29E-133 |
| *NFKBIA* | 4,39E-219 | 6,55E-217 | 38,6 |  | 94,7 | 0,00E+00 | 0,00E+00 |
| *SLC25A37* | 6,65E-218 | 9,78E-216 | 18,0 |  | 0,4 | 1,86E-01 | 2,45E-01 |
| *MLKL* | 8,26E-217 | 1,20E-214 | 40,3 |  | 21,9 | 1,72E-211 | 6,20E-209 |
| *DCN* | 1,11E-212 | 1,59E-210 | 1224,1 |  | 10,5 | 1,06E-129 | 1,70E-127 |
| *SP100* | 7,25E-210 | 1,03E-207 | 7,6 |  | 4,7 | 3,42E-75 | 2,25E-73 |
| *NRK* | 3,51E-209 | 4,92E-207 | 21,4 |  | 1256,7 | 8,89E-101 | 9,58E-99 |
| *CMPK2* | 1,78E-203 | 2,47E-201 | 8181,1 |  | 32198,3 | 2,05E-185 | 6,14E-183 |
| *PMAIP1* | 1,07E-197 | 1,47E-195 | 23,3 |  | 43,0 | 6,66E-49 | 2,38E-47 |
| *SLC2A6* | 6,52E-197 | 8,84E-195 | 28,2 |  | 26,9 | 5,64E-97 | 5,46E-95 |
| *USP18* | 3,07E-196 | 4,11E-194 | 184,9 |  | 125,2 | 5,12E-115 | 6,62E-113 |
| *LAMA4* | 2,17E-195 | 2,88E-193 | -15,6 |  | -3,4 | 5,42E-11 | 2,94E-10 |
| *SERPINB1* | 9,87E-192 | 1,30E-189 | 43,2 |  | 6,7 | 2,19E-15 | 1,66E-14 |
| *RRAD* | 6,15E-191 | 7,98E-189 | 833,9 |  | 610,7 | 3,27E-40 | 8,78E-39 |
| *ACSL1* | 7,65E-191 | 9,82E-189 | 33,6 |  | 4,5 | 9,00E-17 | 7,65E-16 |
| *BTN3A3* | 4,03E-187 | 5,11E-185 | 211,6 |  | 19,1 | 6,26E-130 | 1,01E-127 |
| *PSME1* | 5,61E-186 | 7,05E-184 | 21,7 |  | 19,3 | 2,46E-123 | 3,56E-121 |
| *HAS2* | 7,89E-186 | 9,81E-184 | 62,2 |  | 12,1 | 4,06E-11 | 2,24E-10 |
| *CASP1* | 1,27E-183 | 1,56E-181 | 605,4 |  | 25,6 | 2,27E-09 | 1,06E-08 |
| *DUSP4* | 1,52E-183 | 1,85E-181 | -21,5 |  | -4,3 | 2,95E-10 | 1,50E-09 |
| *IFITM1* | 1,10E-182 | 1,32E-180 | 298,8 |  | 221,4 | 2,66E-24 | 3,53E-23 |
| *LGALS3BP* | 1,25E-182 | 1,50E-180 | 13,3 |  | 8,4 | 5,83E-43 | 1,75E-41 |
| *BIRC3* | 3,33E-177 | 3,94E-175 | 16968,3 |  | 4161,0 | 3,78E-90 | 3,11E-88 |
| *GBP2* | 3,08E-174 | 3,61E-172 | 19542,8 |  | 116,3 | 3,37E-68 | 1,86E-66 |
| *NFKBIZ* | 2,29E-173 | 2,65E-171 | 58,9 |  | 12,4 | 2,12E-30 | 3,79E-29 |
| *PSD3* | 9,79E-172 | 1,12E-169 | -14,7 |  | -8,4 | 6,42E-38 | 1,58E-36 |
| *CD74* | 1,71E-170 | 1,95E-168 | 9395,4 |  | 65,9 | 1,25E-14 | 9,02E-14 |
| *AMPD3* | 5,90E-170 | 6,66E-168 | 37,2 |  | 12,7 | 8,69E-47 | 2,99E-45 |
| *CTSZ* | 3,00E-169 | 3,36E-167 | 55,0 |  | 2,6 | 6,12E-24 | 7,96E-23 |
| *SP110* | 1,05E-168 | 1,17E-166 | 12,5 |  | 23,7 | 2,14E-119 | 2,93E-117 |
| *AKR1B1* | 1,75E-168 | 1,92E-166 | 8,2 |  | 5,8 | 3,40E-27 | 5,34E-26 |
| *APOL3* | 3,31E-166 | 3,60E-164 | 5437,2 |  | 2387,8 | 8,82E-82 | 6,53E-80 |
| *SLFN5* | 2,04E-165 | 2,19E-163 | 11,0 |  | 0,7 | 4,93E-02 | 7,62E-02 |
| *HLA-F* | 1,19E-164 | 1,27E-162 | 41747,8 |  | 242,0 | 1,71E-185 | 5,26E-183 |
| *NRP2* | 2,32E-164 | 2,45E-162 | 13,0 |  | 10,6 | 4,90E-75 | 3,18E-73 |
| *TRIM22* | 4,98E-163 | 5,22E-161 | 11,8 |  | 6,1 | 9,29E-16 | 7,27E-15 |
| *PDCD1LG2* | 5,93E-162 | 6,17E-160 | 8,5 |  | 6,6 | 1,57E-12 | 9,79E-12 |
| *NINJ1* | 7,01E-161 | 7,23E-159 | 72,2 |  | 51,0 | 5,94E-90 | 4,83E-88 |
| *WTAP* | 5,99E-160 | 6,12E-158 | 9,9 |  | 15,8 | 3,50E-84 | 2,72E-82 |
| *PARP12* | 6,13E-158 | 6,22E-156 | 186,6 |  | 116,5 | 8,55E-186 | 2,69E-183 |
| *AK5* | 5,26E-157 | 5,29E-155 | -28,7 |  | -23,4 | 1,44E-118 | 1,94E-116 |
| *TRIM69* | 1,51E-156 | 1,51E-154 | 20,5 |  | 12,6 | 3,28E-83 | 2,50E-81 |
| *PLOD2* | 2,11E-156 | 2,09E-154 | 25,1 |  | 5,7 | 1,90E-33 | 4,00E-32 |
| *ST3GAL1* | 2,85E-156 | 2,80E-154 | 14,7 |  | 12,9 | 5,19E-90 | 4,25E-88 |
| *OPTN* | 1,30E-153 | 1,27E-151 | 11,2 |  | 11,0 | 2,57E-243 | 1,13E-240 |
| *SLC39A8* | 1,15E-152 | 1,11E-150 | 20,3 |  | 109,1 | 1,30E-15 | 1,01E-14 |
| *LAMC2* | 2,33E-152 | 2,23E-150 | 92,9 |  | 4,6 | 1,57E-34 | 3,46E-33 |
| *NMI* | 1,44E-145 | 1,37E-143 | 47,6 |  | 27,3 | 8,26E-56 | 3,41E-54 |
| *GK* | 5,62E-144 | 5,30E-142 | 36,4 |  | 7,0 | 1,22E-30 | 2,21E-29 |
| *MSC* | 5,74E-140 | 5,37E-138 | 645,7 |  | 95,6 | 4,45E-43 | 1,34E-41 |
| *SAT1* | 1,71E-139 | 1,59E-137 | 23,9 |  | 12,0 | 5,49E-38 | 1,36E-36 |
| *STOM* | 1,69E-138 | 1,55E-136 | 8,7 |  | 8,8 | 7,14E-23 | 8,81E-22 |
| *CD82* | 3,20E-138 | 2,92E-136 | 37,0 |  | 41,6 | 7,87E-60 | 3,57E-58 |
| *PPFIA2* | 1,09E-137 | 9,92E-136 | 53,1 |  | 3,1 | 7,40E-05 | 1,97E-04 |
| *TAPBPL* | 1,13E-137 | 1,01E-135 | 108,3 |  | 25,6 | 2,04E-71 | 1,22E-69 |
| *HELZ2* | 3,82E-137 | 3,42E-135 | 50,4 |  | 77,4 | 3,45E-147 | 6,94E-145 |
| *CXCL2* | 6,39E-136 | 5,67E-134 | 1680,0 |  | 305,6 | 4,98E-95 | 4,64E-93 |
| *SESN3* | 6,79E-136 | 5,98E-134 | -62,8 |  | -25,8 | 6,69E-79 | 4,72E-77 |
| *IRAK2* | 1,94E-134 | 1,70E-132 | 64,2 |  | 44,2 | 6,13E-206 | 2,15E-203 |
| *GALNT5* | 1,62E-133 | 1,41E-131 | -20,7 |  | -14,0 | 9,85E-78 | 6,87E-76 |
| *PAMR1* | 2,18E-133 | 1,88E-131 | -53,2 |  | -15,3 | 1,54E-46 | 5,24E-45 |
| *CXCL9* | 3,04E-133 | 2,60E-131 | 889345322,1 |  | 59058251,5 | 1,45E-66 | 7,66E-65 |
| *IFIT5* | 4,76E-133 | 4,04E-131 | 12,1 |  | 8,4 | 2,99E-142 | 5,57E-140 |
| *TRAF1* | 4,10E-131 | 3,46E-129 | 3083,7 |  | 207,0 | 5,57E-21 | 6,25E-20 |
| *CFLAR* | 9,74E-131 | 8,16E-129 | 10,3 |  | 8,4 | 5,72E-130 | 9,36E-128 |
| *SLC31A2* | 2,30E-130 | 1,91E-128 | 45,9 |  | 16,5 | 6,09E-146 | 1,19E-143 |
| *KYNU* | 4,89E-130 | 4,04E-128 | 211,5 |  | 537,6 | 4,93E-31 | 9,14E-30 |
| *ENSG00000215861* | 6,94E-130 | 5,69E-128 | 61,7 |  | 7,0 | 4,72E-18 | 4,42E-17 |
| *BMP2* | 1,35E-126 | 1,10E-124 | 81,0 |  | 186,2 | 1,60E-28 | 2,64E-27 |
| *IFITM3* | 2,49E-124 | 2,02E-122 | 21,6 |  | 17,6 | 2,56E-52 | 9,81E-51 |
| *TAP2* | 4,14E-124 | 3,33E-122 | 58,1 |  | 72,3 | 4,90E-242 | 2,07E-239 |
| *TRIB1* | 2,68E-122 | 2,14E-120 | 21,9 |  | 6,2 | 3,03E-34 | 6,59E-33 |
| *ATP10A* | 6,73E-122 | 5,34E-120 | 28,0 |  | 15,9 | 3,67E-10 | 1,86E-09 |
| *RELB* | 8,94E-122 | 7,05E-120 | 31,1 |  | 39,5 | 4,28E-92 | 3,65E-90 |
| *CPA4* | 9,02E-122 | 7,07E-120 | -20,2 |  | 0,4 | 2,86E-01 | 3,55E-01 |
| *ALPK1* | 1,66E-121 | 1,29E-119 | 29,4 |  | 6,5 | 8,63E-18 | 7,94E-17 |
| *ZNFX1* | 2,21E-121 | 1,70E-119 | 10,5 |  | 21,6 | 8,04E-216 | 2,99E-213 |
| *TMEM62* | 3,70E-121 | 2,83E-119 | 21,6 |  | 20,2 | 3,81E-130 | 6,32E-128 |
| *AKAP12* | 1,46E-120 | 1,11E-118 | 8,8 |  | -3,3 | 1,25E-06 | 4,28E-06 |
| *PDE4DIP* | 1,65E-120 | 1,24E-118 | 8,2 |  | 4,8 | 1,83E-29 | 3,14E-28 |
| *SECTM1* | 2,68E-120 | 2,01E-118 | 796,6 |  | 274,9 | 7,47E-29 | 1,25E-27 |
| *ITGBL1* | 2,40E-118 | 1,79E-116 | -45,2 |  | -18,2 | 3,73E-221 | 1,48E-218 |
| *TRIM21* | 5,41E-118 | 4,01E-116 | 19,7 |  | 29,5 | 1,76E-130 | 2,96E-128 |
| *SOCS3* | 6,87E-118 | 5,06E-116 | 27,7 |  | 4,5 | 2,26E-13 | 1,50E-12 |
| *INKA2* | 7,97E-118 | 5,83E-116 | -130,4 |  | -21,0 | 5,61E-18 | 5,22E-17 |
| *MT2A* | 1,68E-117 | 1,22E-115 | 8,7 |  | 19,8 | 1,50E-13 | 1,00E-12 |
| *MMP3* | 1,30E-116 | 9,43E-115 | 25,3 |  | 15757,7 | 5,02E-51 | 1,87E-49 |
| *CXCL10* | 1,35E-115 | 9,66E-114 | 219705121,5 |  | 27617545,8 | 2,04E-56 | 8,58E-55 |
| *ISG15* | 3,11E-115 | 2,22E-113 | 130,3 |  | 972,7 | 0,00E+00 | 0,00E+00 |
| *IL1B* | 7,04E-115 | 5,00E-113 | 8819,2 |  | 803,1 | 1,09E-60 | 5,02E-59 |
| *CCDC71L* | 1,96E-113 | 1,38E-111 | 7,6 |  | 20,9 | 2,77E-103 | 3,09E-101 |
| *ANTXR2* | 7,95E-113 | 5,58E-111 | 8,5 |  | 3,0 | 1,53E-31 | 2,91E-30 |
| *NCOA7* | 8,22E-113 | 5,73E-111 | 36,9 |  | 9,8 | 6,19E-33 | 1,26E-31 |
| *NFKB1* | 8,12E-112 | 5,63E-110 | 10,7 |  | 5,6 | 1,23E-62 | 5,96E-61 |
| *PRSS23* | 8,32E-112 | 5,74E-110 | -9,4 |  | -9,1 | 3,83E-100 | 4,06E-98 |
| *RHBDF2* | 9,12E-112 | 6,25E-110 | 36,2 |  | 63,9 | 1,94E-55 | 7,94E-54 |
| *NFE2L3* | 1,37E-111 | 9,32E-110 | 6,1 |  | 111,4 | 6,20E-25 | 8,57E-24 |
| *CCND2* | 3,78E-111 | 2,56E-109 | -59,6 |  | -3,0 | 1,72E-01 | 2,30E-01 |
| *IKBKE* | 1,38E-110 | 9,29E-109 | 9,6 |  | 14,5 | 1,42E-36 | 3,36E-35 |
| *CD83* | 1,43E-110 | 9,55E-109 | 537,1 |  | 120,3 | 1,05E-22 | 1,29E-21 |
| *CXCL11* | 8,14E-110 | 5,42E-108 | 127485940,0 |  | 233605209,0 | 2,81E-116 | 3,76E-114 |
| *IDO1* | 1,77E-109 | 1,17E-107 | 124829213,1 |  | 1315333,0 | 0,00E+00 | 0,00E+00 |
| *TDRD7* | 1,87E-109 | 1,23E-107 | 34,3 |  | 22,0 | 4,37E-184 | 1,28E-181 |
| *NFKB2* | 1,18E-107 | 7,76E-106 | 48,3 |  | 29,2 | 1,44E-146 | 2,85E-144 |
| *PPIF* | 1,62E-107 | 1,06E-105 | 7,9 |  | 6,2 | 3,37E-89 | 2,69E-87 |
| *FHL1* | 6,14E-107 | 3,98E-105 | -17,9 |  | -7,9 | 1,31E-14 | 9,44E-14 |
| *PSMB8* | 9,87E-107 | 6,37E-105 | 18,6 |  | 4,9 | 1,49E-56 | 6,29E-55 |
| *BTN2A1* | 8,41E-105 | 5,40E-103 | 10,6 |  | 8,6 | 5,25E-107 | 6,08E-105 |
| *ADAR* | 2,76E-104 | 1,76E-102 | 6,6 |  | 6,5 | 8,45E-77 | 5,67E-75 |
| *PCDH7* | 4,65E-104 | 2,95E-102 | 40,3 |  | 8,3 | 3,69E-32 | 7,29E-31 |
| *FLRT3* | 5,08E-103 | 3,21E-101 | -111,1 |  | -21,8 | 1,12E-20 | 1,23E-19 |
| *SBNO2* | 5,81E-103 | 3,65E-101 | 13,6 |  | 4,8 | 5,94E-46 | 1,97E-44 |
| *SFMBT2* | 1,94E-102 | 1,21E-100 | 53,6 |  | 187,4 | 7,33E-19 | 7,28E-18 |
| *MCL1* | 4,65E-102 | 2,89E-100 | 4,7 |  | 3,5 | 1,34E-54 | 5,42E-53 |
| *CASP4* | 6,07E-102 | 3,76E-100 | 11,0 |  | 7,6 | 6,05E-129 | 9,41E-127 |
| *CSRNP1* | 7,01E-102 | 4,32E-100 | 19,0 |  | 20,1 | 9,46E-27 | 1,45E-25 |
| *PML* | 1,45E-101 | 8,86E-100 | 26,1 |  | 12,5 | 6,23E-39 | 1,61E-37 |
| *AK4* | 2,73E-101 | 1,67E-99 | 33,0 |  | 9,9 | 1,36E-25 | 1,96E-24 |
| *SDC2* | 8,71E-101 | 5,28E-99 | -35,0 |  | -12,8 | 3,52E-23 | 4,42E-22 |
| *MID1* | 1,75E-100 | 1,05E-98 | -12,9 |  | -4,1 | 2,94E-19 | 3,00E-18 |
| *DLG1* | 2,74E-100 | 1,64E-98 | -7,3 |  | -3,4 | 5,67E-09 | 2,54E-08 |
| *TMEM132A* | 2,79E-100 | 1,67E-98 | 12,0 |  | 13,4 | 5,32E-68 | 2,93E-66 |
| *PDP1* | 6,06E-100 | 3,60E-98 | 5,4 |  | 2,2 | 8,71E-14 | 5,93E-13 |
| *MSC-AS1* | 6,29E-100 | 3,72E-98 | 47,7 |  | 2,9 | 3,30E-07 | 1,22E-06 |
| *SCD5* | 1,05E-99 | 6,20E-98 | -24,6 |  | -7,8 | 2,04E-13 | 1,36E-12 |
| *COL3A1* | 1,35E-99 | 7,88E-98 | -57,6 |  | -12,0 | 5,39E-27 | 8,37E-26 |
| *NUB1* | 1,65E-99 | 9,60E-98 | 9,5 |  | 16,1 | 3,24E-172 | 8,11E-170 |
| *IL32* | 4,64E-99 | 2,68E-97 | 37198,5 |  | 7953613,3 | 2,87E-79 | 2,04E-77 |
| *SGCD* | 3,67E-98 | 2,11E-96 | -161,1 |  | -181,2 | 6,46E-41 | 1,78E-39 |
| *IL4R* | 1,01E-97 | 5,77E-96 | 11,6 |  | 5,7 | 1,42E-55 | 5,82E-54 |
| *LGMN* | 1,44E-97 | 8,22E-96 | 14,7 |  | 15,3 | 5,20E-38 | 1,30E-36 |
| *STC2* | 3,15E-97 | 1,79E-95 | 10,3 |  | 1,2 | 3,41E-03 | 6,81E-03 |
| *RCAN1* | 7,36E-97 | 4,15E-95 | -5,0 |  | 1,2 | 2,29E-03 | 4,74E-03 |
| *PLAT* | 1,22E-96 | 6,85E-95 | -17,2 |  | -7,2 | 1,70E-04 | 4,26E-04 |
| *DRAM1* | 3,66E-96 | 2,05E-94 | 12,1 |  | 31,6 | 0,00E+00 | 0,00E+00 |
| *FZD2* | 4,32E-96 | 2,40E-94 | -6,4 |  | -3,6 | 6,42E-08 | 2,59E-07 |
| *ADGRL4* | 4,42E-96 | 2,45E-94 | 8,9 |  | 1,2 | 3,76E-05 | 1,05E-04 |
| *DENND5A* | 5,32E-96 | 2,93E-94 | 6,1 |  | 4,5 | 8,31E-35 | 1,86E-33 |
| *CIITA* | 7,64E-96 | 4,19E-94 | 82,0 |  | 26,0 | 1,24E-72 | 7,56E-71 |
| *ATP13A3* | 1,32E-95 | 7,19E-94 | 9,0 |  | 2,6 | 1,62E-07 | 6,23E-07 |
| *SLC15A3* | 1,84E-94 | 1,00E-92 | 1804,1 |  | 291,2 | 9,06E-77 | 6,04E-75 |
| *TRAFD1* | 2,28E-94 | 1,24E-92 | 7,5 |  | 13,8 | 1,15E-167 | 2,82E-165 |
| *NLGN4Y* | 2,49E-93 | 1,34E-91 | 11,5 |  | 0,1 | 3,58E-01 | 4,31E-01 |
| *CYBA* | 3,24E-93 | 1,74E-91 | 8,8 |  | 3,7 | 1,61E-46 | 5,47E-45 |
| *ACSL5* | 4,44E-93 | 2,37E-91 | 195,9 |  | 29,8 | 9,28E-33 | 1,87E-31 |
| *CCDC80* | 5,21E-93 | 2,77E-91 | -6,7 |  | -11,1 | 1,63E-41 | 4,60E-40 |
| *IFI27* | 5,29E-93 | 2,80E-91 | 28772,9 |  | 6042,9 | 1,79E-259 | 9,16E-257 |
| *SP140L* | 4,26E-92 | 2,24E-90 | 7,9 |  | 2,2 | 2,27E-10 | 1,17E-09 |
| *CYP27B1* | 6,81E-92 | 3,56E-90 | 208,7 |  | 3488,5 | 1,07E-40 | 2,92E-39 |
| *NFKBIE* | 1,76E-91 | 9,14E-90 | 21,2 |  | 63,6 | 7,43E-103 | 8,22E-101 |
| *CCL5* | 4,22E-91 | 2,18E-89 | 54025049,6 |  | 1558700,2 | 9,02E-46 | 2,97E-44 |
| *C1R* | 8,49E-91 | 4,38E-89 | 785,2 |  | 6,2 | 1,27E-03 | 2,75E-03 |
| *GLIPR1* | 7,13E-90 | 3,66E-88 | -6,0 |  | -7,5 | 2,62E-20 | 2,82E-19 |
| *CSF1* | 1,48E-88 | 7,59E-87 | 30,3 |  | 51,3 | 1,31E-59 | 5,86E-58 |
| *CHST2* | 2,21E-88 | 1,12E-86 | 38,5 |  | -2,8 | 3,32E-05 | 9,39E-05 |
| *ZC3H12C* | 2,83E-88 | 1,44E-86 | 10,7 |  | 6,3 | 2,03E-16 | 1,68E-15 |
| *SPP1* | 3,26E-87 | 1,64E-85 | -9,1 |  | 0,2 | 5,53E-01 | 6,19E-01 |
| *ZC3HAV1* | 5,65E-87 | 2,84E-85 | 10,4 |  | 2,9 | 4,62E-42 | 1,35E-40 |
| *NID2* | 8,95E-87 | 4,46E-85 | -54,8 |  | -28,8 | 2,42E-58 | 1,07E-56 |
| *STAT5A* | 3,25E-86 | 1,61E-84 | 186,4 |  | 8,9 | 5,42E-06 | 1,71E-05 |
| *RNF19B* | 3,81E-86 | 1,88E-84 | 18,0 |  | 30,8 | 4,63E-137 | 8,36E-135 |
| *RFLNA* | 3,84E-86 | 1,89E-84 | 25,1 |  | 20,0 | 6,36E-10 | 3,15E-09 |
| *UBASH3B* | 3,89E-86 | 1,91E-84 | -5,6 |  | 0,3 | 3,76E-01 | 4,48E-01 |
| *QPRT* | 4,89E-86 | 2,39E-84 | -25,4 |  | -10,1 | 3,40E-21 | 3,85E-20 |
| *ATP8B1* | 7,71E-86 | 3,75E-84 | -14,3 |  | -5,3 | 7,88E-18 | 7,26E-17 |
| *LRCH2* | 1,54E-85 | 7,44E-84 | 18,5 |  | 19,5 | 4,99E-24 | 6,54E-23 |
| *CD47* | 1,56E-85 | 7,54E-84 | 6,1 |  | 3,5 | 5,31E-43 | 1,60E-41 |
| *SAMD9* | 2,09E-85 | 1,00E-83 | 615,4 |  | 44,2 | 7,90E-110 | 9,42E-108 |
| *KITLG* | 5,48E-85 | 2,62E-83 | -14,6 |  | -10,6 | 1,99E-43 | 6,02E-42 |
| *XRN1* | 7,59E-85 | 3,62E-83 | 8,3 |  | 5,0 | 5,77E-18 | 5,36E-17 |
| *SERPINB7* | 8,33E-85 | 3,96E-83 | 5,8 |  | 64,7 | 1,99E-14 | 1,41E-13 |
| *OAS1* | 9,06E-85 | 4,29E-83 | 53763,4 |  | 1967,3 | 5,64E-244 | 2,57E-241 |
| *ACOD1* | 2,33E-84 | 1,09E-82 | 12309723,0 |  | 1459197,7 | 6,50E-67 | 3,49E-65 |
| *RPS6KA1* | 3,14E-84 | 1,47E-82 | 23,7 |  | 12,2 | 9,51E-17 | 8,06E-16 |
| *TTC3* | 4,61E-84 | 2,15E-82 | -5,5 |  | -25,5 | 8,02E-96 | 7,58E-94 |
| *SLC41A2* | 3,90E-83 | 1,80E-81 | 14,7 |  | 6,9 | 1,00E-31 | 1,94E-30 |
| *GBP4* | 4,55E-83 | 2,10E-81 | 11401771,5 |  | 192106,7 | 2,30E-77 | 1,57E-75 |
| *MET* | 5,67E-83 | 2,60E-81 | 4,7 |  | 2,1 | 1,67E-08 | 7,13E-08 |
| *DSEL* | 6,16E-83 | 2,82E-81 | -5,7 |  | -6,2 | 3,22E-05 | 9,12E-05 |
| *NUAK2* | 2,41E-82 | 1,10E-80 | 17,9 |  | 100,7 | 5,66E-82 | 4,21E-80 |
| *ETV7* | 2,82E-82 | 1,28E-80 | 9982,3 |  | 1306,6 | 2,96E-45 | 9,55E-44 |
| *STAMBPL1* | 4,23E-82 | 1,91E-80 | 13,4 |  | 7,7 | 3,13E-22 | 3,73E-21 |
| *MICB* | 6,19E-82 | 2,79E-80 | 12,0 |  | 20,4 | 2,84E-65 | 1,46E-63 |
| *FAM111A* | 6,29E-82 | 2,82E-80 | 6,8 |  | 1,3 | 7,15E-10 | 3,53E-09 |
| *MYO1B* | 6,60E-82 | 2,95E-80 | 4,9 |  | 0,9 | 3,14E-05 | 8,93E-05 |
| *RNF114* | 1,81E-81 | 8,05E-80 | 5,0 |  | 12,9 | 1,32E-249 | 6,23E-247 |
| *STAC* | 1,86E-81 | 8,25E-80 | -14,6 |  | -43,2 | 5,75E-12 | 3,43E-11 |
| *RN7SL3* | 2,94E-81 | 1,30E-79 | 7,5 |  | 2,2 | 1,31E-12 | 8,19E-12 |
| *CTSO* | 6,47E-81 | 2,85E-79 | 19,3 |  | 2,7 | 1,18E-13 | 7,96E-13 |
| *CA13* | 1,40E-80 | 6,12E-79 | 36,5 |  | 11,3 | 5,87E-16 | 4,69E-15 |
| *TRHDE* | 1,86E-80 | 8,13E-79 | -21,3 |  | -1453,8 | 3,07E-13 | 2,01E-12 |
| *NAV3* | 2,65E-80 | 1,15E-78 | 5,6 |  | 5,4 | 4,54E-52 | 1,73E-50 |
| *CNDP2* | 3,67E-80 | 1,59E-78 | 8,7 |  | 10,0 | 6,21E-90 | 5,02E-88 |
| *HLA-DMA* | 4,37E-80 | 1,89E-78 | 40,1 |  | 5,3 | 1,73E-11 | 9,93E-11 |
| *TAPBP* | 5,23E-80 | 2,26E-78 | 15,7 |  | 13,8 | 2,68E-58 | 1,17E-56 |
| *SLC16A3* | 5,41E-80 | 2,33E-78 | 10,6 |  | 14,4 | 2,90E-25 | 4,13E-24 |
| *BTN2A2* | 9,38E-80 | 4,00E-78 | 12,2 |  | 6,9 | 9,37E-66 | 4,86E-64 |
| *MME* | 1,03E-79 | 4,39E-78 | 5,2 |  | 10,2 | 3,41E-55 | 1,39E-53 |
| *STAT6* | 1,36E-79 | 5,76E-78 | 4,7 |  | 4,0 | 3,64E-37 | 8,84E-36 |
| *ALDH1A3* | 4,54E-79 | 1,92E-77 | 18,8 |  | 0,5 | 7,99E-02 | 1,17E-01 |
| *LITAF* | 2,62E-78 | 1,10E-76 | 10,7 |  | 6,5 | 6,06E-32 | 1,18E-30 |
| *GRIA1* | 2,11E-77 | 8,84E-76 | -27,8 |  | -6,6 | 2,43E-03 | 4,99E-03 |
| *GJD3* | 3,27E-77 | 1,37E-75 | 3376,3 |  | 109,0 | 2,76E-26 | 4,10E-25 |
| *CTSL* | 8,10E-77 | 3,37E-75 | 5,9 |  | 2,4 | 1,43E-28 | 2,37E-27 |
| *GPR68* | 1,53E-76 | 6,33E-75 | 17,9 |  | -2,0 | 9,95E-01 | 9,95E-01 |
| *ADM* | 7,75E-76 | 3,18E-74 | 5,6 |  | 30,8 | 7,04E-38 | 1,73E-36 |
| *CDC42SE2* | 1,06E-75 | 4,32E-74 | 5,9 |  | 7,9 | 3,73E-75 | 2,44E-73 |
| *IFNGR2* | 1,42E-75 | 5,78E-74 | 14,5 |  | 0,3 | 3,04E-03 | 6,14E-03 |
| *SELENOM* | 3,46E-75 | 1,41E-73 | 7,2 |  | 8,0 | 2,51E-58 | 1,10E-56 |
| *EPSTI1* | 4,42E-75 | 1,79E-73 | 1020,8 |  | 97,4 | 8,73E-14 | 5,94E-13 |
| *CXCL1* | 7,09E-75 | 2,86E-73 | 1283,2 |  | 3362,5 | 1,79E-14 | 1,28E-13 |
| *HIVEP2* | 8,45E-75 | 3,40E-73 | 10,9 |  | 3,3 | 1,06E-24 | 1,44E-23 |
| *BTG3* | 1,19E-74 | 4,76E-73 | 12,2 |  | 5,7 | 4,62E-25 | 6,46E-24 |
| *CDCP1* | 3,11E-74 | 1,24E-72 | 9,6 |  | 22,4 | 3,72E-30 | 6,56E-29 |
| *LGALS8* | 3,16E-74 | 1,26E-72 | 4,5 |  | 3,3 | 2,17E-21 | 2,49E-20 |
| *PTCHD4* | 1,03E-73 | 4,07E-72 | -18,2 |  | 1,3 | 6,39E-05 | 1,72E-04 |
| *TMEM255B* | 1,14E-72 | 4,52E-71 | 10,2 |  | 2,6 | 4,26E-04 | 1,00E-03 |
| *LTBP1* | 1,78E-72 | 7,03E-71 | -6,3 |  | -6,6 | 6,85E-31 | 1,26E-29 |
| *CARD6* | 1,18E-71 | 4,65E-70 | 65,8 |  | 1,0 | 6,11E-09 | 2,73E-08 |
| *MOXD1* | 1,44E-71 | 5,64E-70 | -5,2 |  | 0,3 | 5,69E-01 | 6,35E-01 |
| *CCDC50* | 2,61E-71 | 1,02E-69 | 3,7 |  | 2,4 | 1,83E-18 | 1,77E-17 |
| *LIMD2* | 2,85E-71 | 1,11E-69 | 8,5 |  | 9,4 | 3,91E-27 | 6,13E-26 |
| *NF2* | 2,96E-71 | 1,15E-69 | -4,5 |  | -3,3 | 1,40E-06 | 4,78E-06 |
| *LUM* | 4,68E-71 | 1,81E-69 | 150,6 |  | 3,2 | 5,96E-25 | 8,26E-24 |
| *SLAMF8* | 1,21E-70 | 4,66E-69 | 2977943,2 |  | 79994,9 | 6,25E-34 | 1,33E-32 |
| *CALCOCO2* | 2,58E-70 | 9,92E-69 | 6,0 |  | 1,9 | 3,31E-20 | 3,55E-19 |
| *PTPRK* | 3,70E-70 | 1,42E-68 | 4,5 |  | 0,5 | 2,71E-03 | 5,52E-03 |
| *PLSCR1* | 3,76E-70 | 1,44E-68 | 81,7 |  | 43,3 | 2,64E-69 | 1,49E-67 |
| *CLIP2* | 7,55E-70 | 2,87E-68 | 7,1 |  | 2,4 | 3,25E-45 | 1,04E-43 |
| *SLC25A28* | 9,36E-70 | 3,55E-68 | 10,3 |  | 19,8 | 7,81E-98 | 7,73E-96 |
| *ACAT2* | 1,71E-69 | 6,47E-68 | -7,4 |  | -5,6 | 1,19E-25 | 1,73E-24 |
| *OTUD3* | 1,97E-69 | 7,42E-68 | -14,2 |  | -5,3 | 1,69E-03 | 3,57E-03 |
| *TKT* | 9,54E-69 | 3,59E-67 | -4,3 |  | -5,6 | 6,75E-30 | 1,18E-28 |
| *GPC1* | 9,93E-69 | 3,72E-67 | -8,4 |  | -5,5 | 7,34E-18 | 6,78E-17 |
| *NETO2* | 1,01E-68 | 3,76E-67 | -10,0 |  | -11,9 | 2,01E-54 | 8,09E-53 |
| *APOL1* | 2,99E-68 | 1,11E-66 | 38241,5 |  | 260,7 | 2,36E-115 | 3,11E-113 |
| *MLLT6* | 1,61E-67 | 5,95E-66 | 7,5 |  | 1,9 | 7,15E-08 | 2,86E-07 |
| *PIM1* | 5,72E-67 | 2,11E-65 | 25,5 |  | 4,0 | 1,13E-11 | 6,58E-11 |
| *SLC22A4* | 6,02E-67 | 2,22E-65 | 8,6 |  | 6,8 | 8,94E-20 | 9,33E-19 |
| *PIK3R3* | 6,73E-67 | 2,47E-65 | -26,8 |  | 2,1 | 4,54E-05 | 1,25E-04 |
| *NNMT* | 1,59E-66 | 5,81E-65 | 7,0 |  | 0,6 | 2,48E-04 | 6,01E-04 |
| *RNF144B* | 2,01E-66 | 7,32E-65 | 18,3 |  | 131,4 | 2,90E-98 | 2,92E-96 |
| *TRIM14* | 2,20E-66 | 8,00E-65 | 14,5 |  | 1511,6 | 1,82E-26 | 2,74E-25 |
| *PHF11* | 2,30E-66 | 8,32E-65 | 7,5 |  | 4,3 | 1,90E-39 | 5,02E-38 |
| *MSI2* | 4,08E-66 | 1,47E-64 | -7,5 |  | -9,2 | 4,21E-32 | 8,28E-31 |
| *MEST* | 8,13E-66 | 2,93E-64 | -149,4 |  | -16,2 | 1,92E-26 | 2,88E-25 |
| *IRF2* | 9,37E-66 | 3,37E-64 | 7,7 |  | 6,5 | 7,26E-18 | 6,71E-17 |
| *SQSTM1* | 1,06E-65 | 3,79E-64 | 4,9 |  | 3,1 | 9,63E-17 | 8,16E-16 |
| *KLHL5* | 1,34E-65 | 4,78E-64 | 5,0 |  | 0,7 | 1,16E-04 | 2,99E-04 |
| *PARP8* | 1,52E-65 | 5,40E-64 | 12,8 |  | 3,5 | 1,32E-28 | 2,19E-27 |
| *C3orf38* | 1,88E-65 | 6,67E-64 | 6,2 |  | 8,6 | 1,05E-67 | 5,74E-66 |
| *UBA7* | 2,19E-65 | 7,75E-64 | 51,6 |  | 3,5 | 4,58E-12 | 2,76E-11 |
| *INPP4B* | 3,53E-65 | 1,25E-63 | -8,9 |  | -23,5 | 8,39E-69 | 4,68E-67 |
| *CYP1B1* | 3,57E-65 | 1,26E-63 | 177,6 |  | 0,7 | 8,10E-02 | 1,18E-01 |
| *ZNF267* | 3,98E-65 | 1,39E-63 | 18,7 |  | 8,0 | 1,01E-36 | 2,40E-35 |
| *EDN1* | 8,12E-65 | 2,84E-63 | 11,7 |  | 144,2 | 2,68E-12 | 1,64E-11 |
| *XAF1* | 1,06E-64 | 3,68E-63 | 1558217,9 |  | 180,1 | 3,21E-187 | 1,04E-184 |
| *BTN2A3P* | 1,51E-64 | 5,26E-63 | 15,1 |  | 0,5 | 1,95E-01 | 2,56E-01 |
| *UBA6* | 3,07E-64 | 1,06E-62 | 5,6 |  | 3,6 | 7,97E-23 | 9,82E-22 |
| *CXCL6* | 6,97E-64 | 2,41E-62 | 124,7 |  | 645,3 | 6,06E-34 | 1,30E-32 |
| *TRIM38* | 1,95E-63 | 6,71E-62 | 8,1 |  | 6,4 | 1,17E-31 | 2,25E-30 |
| *PDE4B* | 2,36E-63 | 8,10E-62 | 12,0 |  | 9,1 | 1,49E-27 | 2,37E-26 |
| *CYB561* | 2,58E-63 | 8,83E-62 | 19,9 |  | 0,2 | 2,89E-01 | 3,59E-01 |
| *GBP1P1* | 1,29E-62 | 4,40E-61 | 1207899,6 |  | 12617,0 | 2,33E-33 | 4,89E-32 |
| *SKP2* | 2,04E-62 | 6,94E-61 | -8,1 |  | -4,4 | 1,49E-17 | 1,35E-16 |
| *PLCB4* | 2,20E-62 | 7,46E-61 | -14,6 |  | -5,2 | 3,50E-05 | 9,88E-05 |
| *ARHGAP42* | 3,52E-62 | 1,19E-60 | 6,6 |  | 6,2 | 3,16E-27 | 4,96E-26 |
| *JAK2* | 4,01E-62 | 1,35E-60 | 13,2 |  | 7,9 | 4,54E-28 | 7,38E-27 |
| *SUSD6* | 4,48E-62 | 1,51E-60 | 13,6 |  | 8,8 | 9,12E-72 | 5,52E-70 |
| *TGFBR3* | 7,16E-62 | 2,41E-60 | -27,9 |  | -3,2 | 9,99E-12 | 5,85E-11 |
| *STING1* | 7,55E-62 | 2,53E-60 | 16,3 |  | 3,2 | 4,60E-18 | 4,32E-17 |
| *KDM7A* | 9,77E-62 | 3,27E-60 | 28,6 |  | 2,9 | 1,36E-08 | 5,86E-08 |
| *SNX30* | 1,24E-61 | 4,14E-60 | -5,8 |  | -8,6 | 1,23E-30 | 2,22E-29 |
| *NOCT* | 1,43E-61 | 4,76E-60 | 10,7 |  | 10,0 | 5,07E-28 | 8,20E-27 |
| *BNIP3* | 1,87E-61 | 6,20E-60 | 5,7 |  | 17,0 | 1,66E-26 | 2,51E-25 |
| *IL15* | 2,69E-61 | 8,88E-60 | 68,8 |  | 69,4 | 5,31E-75 | 3,43E-73 |
| *ITK* | 3,24E-61 | 1,07E-59 | 11738,7 |  | 2490,3 | 1,21E-43 | 3,69E-42 |
| *RNF213* | 4,80E-61 | 1,58E-59 | 55,8 |  | 24,1 | 2,40E-56 | 1,01E-54 |
| *LMO7* | 5,12E-61 | 1,68E-59 | -6,1 |  | -2,5 | 1,86E-02 | 3,20E-02 |
| *VRK2* | 5,47E-61 | 1,79E-59 | 6,2 |  | 2,0 | 3,15E-13 | 2,06E-12 |
| *PANX1* | 5,57E-61 | 1,82E-59 | 6,0 |  | 10,9 | 1,30E-161 | 2,90E-159 |
| *CCL8* | 5,67E-61 | 1,85E-59 | 1031729,1 |  | 82171,8 | 2,14E-119 | 2,93E-117 |
| *PTN* | 1,07E-60 | 3,46E-59 | 29,7 |  | 1,6 | 4,35E-03 | 8,51E-03 |
| *OAS3* | 1,46E-60 | 4,72E-59 | 1198,3 |  | 797,2 | 0,00E+00 | 0,00E+00 |
| *BAZ1A* | 1,86E-60 | 5,99E-59 | 4,4 |  | 4,8 | 2,90E-52 | 1,11E-50 |
| *PLA2G4C* | 1,87E-60 | 6,02E-59 | 16,0 |  | 20,7 | 9,72E-19 | 9,59E-18 |
| *CNN1* | 1,88E-60 | 6,05E-59 | -14,2 |  | -33,2 | 2,98E-10 | 1,52E-09 |
| *SMYD3* | 4,92E-60 | 1,57E-58 | -16,7 |  | -12,5 | 1,26E-46 | 4,30E-45 |
| *TCIRG1* | 5,05E-60 | 1,61E-58 | 6,0 |  | 3,5 | 1,45E-41 | 4,10E-40 |
| *ARHGEF11* | 5,76E-60 | 1,84E-58 | 5,2 |  | 2,7 | 1,92E-17 | 1,71E-16 |
| *G0S2* | 7,38E-60 | 2,34E-58 | 126,4 |  | 2,4 | 6,59E-03 | 1,24E-02 |
| *HTRA1* | 1,52E-59 | 4,83E-58 | -7,5 |  | -9,1 | 2,57E-62 | 1,24E-60 |
| *C3* | 2,04E-59 | 6,44E-58 | 12581,7 |  | 146,1 | 1,21E-07 | 4,72E-07 |
| *NKX3-1* | 2,80E-59 | 8,83E-58 | 25,6 |  | 12,9 | 1,10E-35 | 2,53E-34 |
| *SLFN11* | 2,89E-59 | 9,08E-58 | 11,8 |  | 2,0 | 5,61E-31 | 1,04E-29 |
| *ENO2* | 4,38E-59 | 1,37E-57 | 10,1 |  | 10,3 | 1,88E-32 | 3,76E-31 |
| *LAPTM4B* | 5,45E-59 | 1,70E-57 | -4,0 |  | -4,0 | 5,82E-15 | 4,31E-14 |
| *GRAMD2B* | 8,17E-59 | 2,55E-57 | 12,4 |  | 0,9 | 7,24E-05 | 1,93E-04 |
| *ISG20* | 1,11E-58 | 3,45E-57 | 433,1 |  | 2514,4 | 1,13E-295 | 7,29E-293 |
| *ATF3* | 1,61E-58 | 4,99E-57 | 137,0 |  | 176,5 | 5,64E-99 | 5,77E-97 |
| *DHCR24* | 1,64E-58 | 5,06E-57 | -10,0 |  | -3,5 | 3,81E-07 | 1,40E-06 |
| *BCL2A1* | 2,26E-58 | 6,97E-57 | 176,8 |  | 77,3 | 3,02E-33 | 6,30E-32 |
| *LYN* | 2,53E-58 | 7,77E-57 | 6,0 |  | 14,5 | 8,72E-43 | 2,61E-41 |
| *SCD* | 3,64E-58 | 1,12E-56 | -8,6 |  | -6,9 | 5,12E-36 | 1,19E-34 |
| *ANKRD28* | 3,90E-58 | 1,19E-56 | -6,2 |  | -5,7 | 2,80E-29 | 4,80E-28 |
| *PRNP* | 4,03E-58 | 1,23E-56 | -6,9 |  | -4,6 | 9,87E-11 | 5,25E-10 |
| *PPP3CC* | 5,18E-58 | 1,58E-56 | 7,1 |  | 5,4 | 1,80E-61 | 8,45E-60 |
| *FADS3* | 6,89E-58 | 2,09E-56 | 7,2 |  | 5,6 | 3,47E-38 | 8,76E-37 |
| *GSDMD* | 8,81E-58 | 2,67E-56 | 13,2 |  | 5,1 | 3,11E-14 | 2,19E-13 |
| *XIRP1* | 1,99E-57 | 6,01E-56 | 727250,1 |  | 32250,4 | 3,76E-34 | 8,14E-33 |
| *CARHSP1* | 2,34E-57 | 7,05E-56 | 10,2 |  | 0,7 | 5,58E-05 | 1,51E-04 |
| *BIRC2* | 2,50E-57 | 7,51E-56 | 7,1 |  | 2,0 | 1,33E-08 | 5,74E-08 |
| *ERO1A* | 3,66E-57 | 1,10E-55 | 5,8 |  | 10,6 | 4,98E-39 | 1,29E-37 |
| *PYGB* | 4,26E-57 | 1,27E-55 | -4,6 |  | -3,4 | 1,70E-06 | 5,74E-06 |
| *LY6E* | 1,10E-56 | 3,28E-55 | 4,3 |  | 22,8 | 1,75E-111 | 2,15E-109 |
| *FBXO6* | 1,86E-56 | 5,49E-55 | 13,1 |  | 44,2 | 2,96E-45 | 9,55E-44 |
| *ZC3H7B* | 6,46E-56 | 1,91E-54 | 3,7 |  | 2,7 | 8,24E-29 | 1,37E-27 |
| *DHX58* | 7,09E-56 | 2,09E-54 | 239,1 |  | 53,3 | 1,31E-58 | 5,79E-57 |
| *SRGN* | 8,92E-56 | 2,62E-54 | 16,3 |  | 6,3 | 8,25E-34 | 1,76E-32 |
| *PRDM1* | 9,10E-56 | 2,67E-54 | 36,6 |  | 34,0 | 5,65E-18 | 5,26E-17 |
| *SLC39A14* | 1,19E-55 | 3,49E-54 | 4,6 |  | 15,6 | 9,19E-29 | 1,53E-27 |
| *EDA2R* | 1,60E-55 | 4,67E-54 | -5,5 |  | -8,6 | 6,05E-29 | 1,02E-27 |
| *RASL10B* | 1,75E-55 | 5,10E-54 | -45,5 |  | -92,9 | 6,93E-16 | 5,50E-15 |
| *C1RL* | 2,17E-55 | 6,30E-54 | 42,4 |  | 1,8 | 7,67E-05 | 2,03E-04 |
| *TMEM140* | 2,23E-55 | 6,44E-54 | 31619,7 |  | 35,1 | 4,24E-52 | 1,62E-50 |
| *KANK1* | 2,82E-55 | 8,15E-54 | 4,1 |  | -4,8 | 1,64E-33 | 3,46E-32 |
| *WNT5A* | 4,48E-55 | 1,29E-53 | 5,4 |  | 10,5 | 2,35E-21 | 2,69E-20 |
| *IRF7* | 7,26E-55 | 2,08E-53 | 39,1 |  | 77,8 | 2,31E-77 | 1,57E-75 |
| *ITGA1* | 8,02E-55 | 2,29E-53 | 9,7 |  | -3,6 | 5,36E-14 | 3,69E-13 |
| *SEC22B* | 8,31E-55 | 2,37E-53 | 3,7 |  | 2,9 | 3,65E-50 | 1,33E-48 |
| *GJA1* | 8,46E-55 | 2,41E-53 | -6,6 |  | -4,6 | 5,96E-38 | 1,48E-36 |
| *PLK3* | 9,57E-55 | 2,72E-53 | 6,2 |  | 4,5 | 4,23E-25 | 5,94E-24 |
| *MRGPRX3* | 9,94E-55 | 2,82E-53 | 99122,4 |  | 48589,4 | 1,97E-36 | 4,64E-35 |
| *TANK* | 9,99E-55 | 2,82E-53 | 8,7 |  | 1,5 | 1,84E-06 | 6,17E-06 |
| *EHD3* | 1,37E-54 | 3,85E-53 | -5,9 |  | -7,7 | 4,58E-27 | 7,14E-26 |
| *GALNT10* | 1,49E-54 | 4,18E-53 | -4,4 |  | -5,3 | 6,73E-14 | 4,60E-13 |
| *LMO4* | 1,56E-54 | 4,39E-53 | 5,6 |  | 15,0 | 2,51E-126 | 3,71E-124 |
| *SEMA3C* | 2,13E-54 | 5,96E-53 | 5,5 |  | 3,1 | 1,94E-45 | 6,29E-44 |
| *HLA-DRB1* | 1,03E-53 | 2,88E-52 | 1077,0 |  | 24,2 | 2,76E-08 | 1,16E-07 |
| *USP25* | 2,83E-53 | 7,89E-52 | 4,0 |  | 2,1 | 3,22E-17 | 2,83E-16 |
| *CYLD* | 4,00E-53 | 1,11E-51 | 3,3 |  | 2,7 | 3,07E-28 | 5,03E-27 |
| *MDK* | 4,51E-53 | 1,25E-51 | 6,4 |  | 38,2 | 6,42E-39 | 1,66E-37 |
| *CRABP2* | 7,82E-53 | 2,17E-51 | -24,7 |  | -8,7 | 8,35E-08 | 3,32E-07 |
| *BTG1* | 9,03E-53 | 2,49E-51 | 5,2 |  | 3,4 | 1,12E-42 | 3,31E-41 |
| *MMP16* | 9,24E-53 | 2,54E-51 | -8,5 |  | -29,7 | 4,61E-61 | 2,14E-59 |
| *FGF7* | 9,65E-53 | 2,65E-51 | 57,8 |  | -2,4 | 4,02E-01 | 4,74E-01 |
| *BACH1* | 1,15E-52 | 3,15E-51 | 3,2 |  | 1,7 | 5,53E-21 | 6,20E-20 |
| *PTPRA* | 1,51E-52 | 4,13E-51 | 4,6 |  | 2,0 | 5,84E-11 | 3,16E-10 |
| *PSEN1* | 2,04E-52 | 5,56E-51 | 4,4 |  | 1,6 | 7,63E-16 | 6,02E-15 |
| *NUDCD1* | 2,28E-52 | 6,19E-51 | 4,9 |  | 7,0 | 1,91E-39 | 5,02E-38 |
| *FLNB* | 2,28E-52 | 6,19E-51 | 5,7 |  | 0,6 | 1,35E-04 | 3,45E-04 |
| *NUCB1* | 3,12E-52 | 8,46E-51 | 4,5 |  | 3,4 | 1,16E-29 | 2,01E-28 |
| *NPAS2* | 4,30E-52 | 1,16E-50 | -14,9 |  | -2,6 | 3,12E-01 | 3,84E-01 |
| *TUB* | 4,56E-52 | 1,23E-50 | -32,6 |  | -17,3 | 1,87E-52 | 7,20E-51 |
| *SHFL* | 9,50E-52 | 2,56E-50 | 6,1 |  | 10,3 | 8,20E-56 | 3,39E-54 |
| *IER3* | 1,06E-51 | 2,83E-50 | 6,7 |  | 5,1 | 1,01E-24 | 1,38E-23 |
| *LAMA3* | 1,80E-51 | 4,82E-50 | 11,9 |  | -4,1 | 1,25E-06 | 4,29E-06 |
| *SDC1* | 1,91E-51 | 5,09E-50 | -6,7 |  | -34,6 | 2,91E-53 | 1,13E-51 |
| *CABLES1* | 2,55E-51 | 6,79E-50 | -9,9 |  | -6,9 | 7,68E-25 | 1,06E-23 |
| *IGFBP3* | 2,69E-51 | 7,16E-50 | 117,7 |  | -2,1 | 8,09E-01 | 8,46E-01 |
| *WSB1* | 2,77E-51 | 7,34E-50 | 5,7 |  | 0,5 | 9,24E-02 | 1,33E-01 |
| *GNPDA1* | 6,77E-51 | 1,79E-49 | -4,4 |  | -3,9 | 4,68E-15 | 3,48E-14 |
| *SRPX* | 8,28E-51 | 2,19E-49 | -4,3 |  | -6,4 | 2,91E-66 | 1,54E-64 |
| *GPD2* | 1,26E-50 | 3,32E-49 | 3,8 |  | 8,1 | 3,09E-62 | 1,48E-60 |
| *SSTR2* | 1,65E-50 | 4,33E-49 | 21290,7 |  | 805,7 | 3,64E-17 | 3,18E-16 |
| *PARP10* | 1,83E-50 | 4,81E-49 | 24,6 |  | 15,4 | 7,48E-42 | 2,16E-40 |
| *ESM1* | 6,93E-50 | 1,81E-48 | 22,6 |  | 25,8 | 9,52E-156 | 1,98E-153 |
| *FZD7* | 8,93E-50 | 2,33E-48 | -5,9 |  | -3,2 | 3,99E-03 | 7,86E-03 |
| *TLR3* | 1,01E-49 | 2,62E-48 | 22,0 |  | 21,5 | 9,15E-28 | 1,47E-26 |
| *GSTK1* | 1,41E-49 | 3,65E-48 | 4,0 |  | 4,3 | 2,97E-25 | 4,21E-24 |
| *PFKFB4* | 1,66E-49 | 4,30E-48 | 8,6 |  | 30,3 | 4,58E-26 | 6,74E-25 |
| *HSDL2* | 2,08E-49 | 5,37E-48 | -5,7 |  | -4,5 | 4,51E-20 | 4,78E-19 |
| *TRIM56* | 2,27E-49 | 5,86E-48 | 7,0 |  | 1,9 | 5,80E-07 | 2,07E-06 |
| *CEBPB* | 2,57E-49 | 6,60E-48 | 8,4 |  | 5,4 | 2,67E-32 | 5,31E-31 |
| *CD40* | 2,93E-49 | 7,52E-48 | 312,0 |  | 948,9 | 4,31E-25 | 6,05E-24 |
| *TNFAIP6* | 3,42E-49 | 8,75E-48 | 229,9 |  | 156,4 | 1,68E-180 | 4,59E-178 |
| *IFI16* | 4,67E-49 | 1,19E-47 | 5,6 |  | 1,9 | 1,19E-04 | 3,05E-04 |
| *SYTL2* | 7,19E-49 | 1,84E-47 | -8,2 |  | -2,2 | 3,04E-01 | 3,75E-01 |
| *CHST11* | 1,11E-48 | 2,81E-47 | 7,8 |  | 6,1 | 1,45E-23 | 1,85E-22 |
| *METRNL* | 1,25E-48 | 3,16E-47 | 9,1 |  | 8,8 | 5,33E-97 | 5,19E-95 |
| *SPARC* | 2,01E-48 | 5,07E-47 | -5,6 |  | -5,8 | 1,27E-12 | 7,97E-12 |
| *SYNGR1* | 2,58E-48 | 6,50E-47 | -18,8 |  | -4,9 | 3,39E-03 | 6,76E-03 |
| *STC1* | 2,65E-48 | 6,67E-47 | 5,6 |  | 51,8 | 4,28E-11 | 2,35E-10 |
| *NRIP1* | 3,16E-48 | 7,95E-47 | 4,4 |  | 1,9 | 6,97E-06 | 2,18E-05 |
| *TSPAN5* | 3,22E-48 | 8,08E-47 | -4,4 |  | -3,4 | 5,26E-09 | 2,37E-08 |
| *EFEMP1* | 4,02E-48 | 1,01E-46 | -16,6 |  | -4,4 | 4,04E-12 | 2,44E-11 |
| *DSE* | 4,46E-48 | 1,11E-46 | 3,6 |  | 0,0 | 9,68E-01 | 9,75E-01 |
| *SCARB2* | 4,48E-48 | 1,12E-46 | 3,3 |  | 5,0 | 1,53E-110 | 1,84E-108 |
| *PTGS2* | 9,42E-48 | 2,34E-46 | 954,4 |  | 361,8 | 0,00E+00 | 0,00E+00 |
| *ERAP2* | 9,98E-48 | 2,48E-46 | 63,8 |  | 1,8 | 2,98E-07 | 1,11E-06 |
| *LSAMP* | 1,06E-47 | 2,63E-46 | -52,4 |  | -850,1 | 5,84E-22 | 6,85E-21 |
| *IFIH1* | 1,69E-47 | 4,19E-46 | 2067,7 |  | 523,0 | 1,47E-49 | 5,30E-48 |
| *CD38* | 5,23E-47 | 1,29E-45 | 197081,4 |  | 88270,5 | 8,21E-93 | 7,15E-91 |
| *CRTAP* | 7,25E-47 | 1,79E-45 | -4,1 |  | -6,8 | 1,06E-34 | 2,35E-33 |
| *TMEM171* | 7,61E-47 | 1,87E-45 | 6,4 |  | 13,2 | 9,29E-37 | 2,23E-35 |
| *CADM1* | 7,98E-47 | 1,96E-45 | 8,6 |  | 20,8 | 1,91E-02 | 3,28E-02 |
| *ELOVL7* | 9,41E-47 | 2,30E-45 | 903,6 |  | 142995,1 | 1,91E-44 | 5,95E-43 |
| *ENDOD1* | 9,70E-47 | 2,37E-45 | -3,4 |  | 0,2 | 2,81E-01 | 3,51E-01 |
| *B4GALT5* | 1,10E-46 | 2,67E-45 | 3,6 |  | 4,5 | 1,42E-85 | 1,12E-83 |
| *KIAA0040* | 1,19E-46 | 2,89E-45 | 1802,9 |  | 744,5 | 2,72E-76 | 1,80E-74 |
| *APOLD1* | 1,63E-46 | 3,96E-45 | 29,7 |  | 1,8 | 3,09E-04 | 7,43E-04 |
| *RIPK1* | 1,94E-46 | 4,69E-45 | 5,1 |  | 3,5 | 2,37E-20 | 2,56E-19 |
| *AIM2* | 3,56E-46 | 8,62E-45 | 185238,5 |  | 25705,4 | 1,92E-24 | 2,56E-23 |
| *RPS6KA3* | 3,63E-46 | 8,75E-45 | 4,8 |  | 0,2 | 2,83E-01 | 3,53E-01 |
| *PSME2* | 3,68E-46 | 8,87E-45 | 6,2 |  | 16,8 | 4,96E-54 | 1,97E-52 |
| *HLA-H* | 4,49E-46 | 1,08E-44 | 213,3 |  | 32,6 | 3,33E-43 | 1,01E-41 |
| *ENSG00000285744* | 4,50E-46 | 1,08E-44 | 187078,0 |  | 13,7 | 1,28E-03 | 2,75E-03 |
| *LGR4* | 4,78E-46 | 1,15E-44 | -5,2 |  | -3,0 | 1,10E-04 | 2,84E-04 |
| *ENSG00000251136* | 4,83E-46 | 1,15E-44 | 7,0 |  | 4,9 | 2,64E-12 | 1,62E-11 |
| *ITGA2* | 5,36E-46 | 1,28E-44 | 9,3 |  | 3,8 | 8,12E-04 | 1,81E-03 |
| *TBC1D9* | 6,61E-46 | 1,57E-44 | 3,3 |  | 9,3 | 9,64E-30 | 1,68E-28 |
| *CYP4V2* | 6,97E-46 | 1,66E-44 | 40,8 |  | 1,6 | 7,44E-05 | 1,98E-04 |
| *GNG11* | 7,20E-46 | 1,71E-44 | 5,8 |  | 3,5 | 3,22E-08 | 1,34E-07 |
| *TIFA* | 1,08E-45 | 2,56E-44 | 21,1 |  | 6,3 | 1,76E-25 | 2,54E-24 |
| *CTNNAL1* | 1,36E-45 | 3,20E-44 | -6,7 |  | -5,6 | 1,19E-13 | 8,06E-13 |
| *ARL4C* | 1,63E-45 | 3,85E-44 | -9,8 |  | -4,4 | 2,58E-17 | 2,28E-16 |
| *ACVRL1* | 1,72E-45 | 4,03E-44 | 20,2 |  | 17,7 | 1,72E-77 | 1,18E-75 |
| *C19orf12* | 1,90E-45 | 4,46E-44 | 4,5 |  | 4,4 | 9,19E-69 | 5,10E-67 |
| *STYK1* | 2,12E-45 | 4,96E-44 | 14,2 |  | 13,6 | 4,42E-23 | 5,52E-22 |
| *UACA* | 3,73E-45 | 8,72E-44 | -8,3 |  | -13,2 | 2,22E-46 | 7,45E-45 |
| *PTGES* | 3,77E-45 | 8,79E-44 | 20,9 |  | 43,9 | 1,26E-73 | 7,95E-72 |
| *ITPR2* | 6,47E-45 | 1,51E-43 | 7,9 |  | -2,1 | 7,53E-01 | 7,98E-01 |
| *RELA* | 6,62E-45 | 1,54E-43 | 5,8 |  | 10,4 | 1,85E-73 | 1,15E-71 |
| *PPP1R15A* | 7,21E-45 | 1,67E-43 | 3,7 |  | 2,3 | 1,20E-18 | 1,17E-17 |
| *CASP7* | 7,25E-45 | 1,68E-43 | 5,6 |  | 7,3 | 5,19E-63 | 2,56E-61 |
| *CD248* | 9,10E-45 | 2,10E-43 | -16,3 |  | -5,1 | 3,27E-06 | 1,06E-05 |
| *ALPK2* | 1,01E-44 | 2,33E-43 | 5,5 |  | -4,2 | 5,04E-06 | 1,60E-05 |
| *MYO10* | 1,11E-44 | 2,56E-43 | 5,1 |  | 0,5 | 1,31E-03 | 2,82E-03 |
| *ADGRE5* | 1,55E-44 | 3,57E-43 | 15,7 |  | 33,0 | 2,58E-65 | 1,33E-63 |
| *OASL* | 1,97E-44 | 4,52E-43 | 40,0 |  | 3739,5 | 5,06E-279 | 2,96E-276 |
| *PTGFRN* | 2,15E-44 | 4,91E-43 | -5,2 |  | -9,5 | 5,20E-26 | 7,63E-25 |
| *CDC42EP4* | 2,20E-44 | 5,02E-43 | 6,0 |  | 5,2 | 8,82E-70 | 5,06E-68 |
| *ANKRD1* | 2,63E-44 | 6,00E-43 | 19,5 |  | 64,8 | 5,39E-08 | 2,19E-07 |
| *DNAJA1* | 3,17E-44 | 7,21E-43 | 3,5 |  | 4,5 | 5,13E-159 | 1,12E-156 |
| *FAM168B* | 3,86E-44 | 8,76E-43 | -3,0 |  | -11,5 | 1,26E-58 | 5,59E-57 |
| *CAPN2* | 4,34E-44 | 9,82E-43 | -3,1 |  | -5,7 | 7,96E-18 | 7,34E-17 |
| *CITED2* | 5,10E-44 | 1,15E-42 | -3,6 |  | 1,2 | 2,21E-03 | 4,58E-03 |
| *IL4I1* | 6,04E-44 | 1,36E-42 | 136292,3 |  | 2216,1 | 5,47E-42 | 1,60E-40 |
| *H6PD* | 6,32E-44 | 1,42E-42 | 6,1 |  | 0,9 | 1,47E-06 | 4,99E-06 |
| *EPB41L3* | 6,48E-44 | 1,46E-42 | 2,5 |  | 3,7 | 2,19E-04 | 5,36E-04 |
| *TNFAIP8* | 7,70E-44 | 1,73E-42 | 16,1 |  | 5,1 | 2,83E-19 | 2,91E-18 |
| *MOB3C* | 7,77E-44 | 1,74E-42 | 10,7 |  | 5,6 | 6,88E-20 | 7,22E-19 |
| *CLDN11* | 8,20E-44 | 1,83E-42 | -7,9 |  | -6,7 | 5,38E-33 | 1,10E-31 |
| *RNF182* | 1,06E-43 | 2,36E-42 | -4,7 |  | 0,1 | 8,76E-01 | 9,02E-01 |
| *UBD* | 1,78E-43 | 3,95E-42 | 300090,3 |  | 188460,8 | 1,17E-45 | 3,84E-44 |
| *CCNDBP1* | 2,20E-43 | 4,89E-42 | 5,3 |  | 1,8 | 2,88E-25 | 4,10E-24 |
| *OR2I1P* | 2,34E-43 | 5,19E-42 | 156368,9 |  | 50735,4 | 2,18E-36 | 5,13E-35 |
| *EHF* | 2,59E-43 | 5,73E-42 | 328,6 |  | 12995,7 | 4,85E-51 | 1,81E-49 |
| *MT1E* | 2,95E-43 | 6,52E-42 | 5,8 |  | 32,2 | 6,77E-49 | 2,42E-47 |
| *ACBD5* | 4,10E-43 | 9,03E-42 | 5,1 |  | 3,0 | 6,54E-35 | 1,47E-33 |
| *GBP3* | 4,21E-43 | 9,27E-42 | 97,7 |  | 42,9 | 0,00E+00 | 0,00E+00 |
| *LGALS17A* | 4,48E-43 | 9,85E-42 | 122738,4 |  | 282791,0 | 1,22E-50 | 4,48E-49 |
| *ANKRD37* | 5,09E-43 | 1,12E-41 | 70,2 |  | 30,1 | 3,12E-12 | 1,90E-11 |
| *MSMO1* | 5,71E-43 | 1,25E-41 | -4,5 |  | 0,2 | 5,17E-01 | 5,86E-01 |
| *SERPINF1* | 5,88E-43 | 1,29E-41 | -3,7 |  | -5,6 | 5,89E-28 | 9,49E-27 |
| *CDC42SE1* | 7,01E-43 | 1,53E-41 | 3,3 |  | 1,6 | 3,45E-18 | 3,26E-17 |
| *DNAJC1* | 7,40E-43 | 1,61E-41 | 4,5 |  | 3,1 | 3,45E-41 | 9,66E-40 |
| *MFAP5* | 1,05E-42 | 2,27E-41 | -12,8 |  | -13,6 | 6,47E-04 | 1,47E-03 |
| *OAS2* | 1,33E-42 | 2,89E-41 | 70333,3 |  | 190,9 | 0,00E+00 | 0,00E+00 |
| *HLA-DQA1* | 1,51E-42 | 3,27E-41 | 118199,6 |  | 761,7 | 4,62E-13 | 2,98E-12 |
| *RSAD2* | 1,81E-42 | 3,90E-41 | 107146,9 |  | 54082,0 | 1,51E-94 | 1,39E-92 |
| *APOL4* | 1,87E-42 | 4,02E-41 | 111440,3 |  | 459,3 | 1,61E-09 | 7,67E-09 |
| *FAM172A* | 2,38E-42 | 5,11E-41 | -10,0 |  | -8,7 | 1,04E-16 | 8,79E-16 |
| *FGF2* | 3,18E-42 | 6,82E-41 | 8,0 |  | 2,6 | 1,89E-11 | 1,08E-10 |
| *NME4* | 3,41E-42 | 7,29E-41 | -4,6 |  | -4,6 | 3,70E-11 | 2,04E-10 |
| *PREPL* | 3,81E-42 | 8,14E-41 | -3,5 |  | -3,9 | 1,43E-09 | 6,82E-09 |
| *TNIP2* | 4,19E-42 | 8,93E-41 | 4,2 |  | 2,6 | 8,36E-30 | 1,46E-28 |
| *EIF4B* | 4,43E-42 | 9,43E-41 | -3,2 |  | -9,3 | 7,91E-63 | 3,89E-61 |
| *NR2F1* | 6,43E-42 | 1,37E-40 | -12,1 |  | 0,8 | 1,26E-02 | 2,24E-02 |
| *SLC37A1* | 7,37E-42 | 1,56E-40 | 459,3 |  | 64,0 | 4,74E-58 | 2,06E-56 |
| *CXCL3* | 7,59E-42 | 1,61E-40 | 98944,0 |  | 612,3 | 3,50E-70 | 2,04E-68 |
| *VCAM1* | 7,81E-42 | 1,65E-40 | 249845,0 |  | 3,5 | 1,62E-04 | 4,08E-04 |
| *TM4SF1* | 7,94E-42 | 1,67E-40 | 3,7 |  | -2,1 | 6,78E-01 | 7,32E-01 |
| *KIT* | 8,67E-42 | 1,83E-40 | -1375,3 |  | -37,8 | 8,95E-03 | 1,64E-02 |
| *UGCG* | 8,82E-42 | 1,85E-40 | 2,9 |  | 4,7 | 6,59E-16 | 5,23E-15 |
| *INSIG2* | 1,11E-41 | 2,32E-40 | 12,9 |  | 10,9 | 7,82E-68 | 4,29E-66 |
| *KCTD11* | 1,19E-41 | 2,49E-40 | 9,3 |  | 2,2 | 2,17E-18 | 2,09E-17 |
| *ITPR3* | 1,24E-41 | 2,59E-40 | 21,7 |  | 1,7 | 3,38E-06 | 1,09E-05 |
| *LPXN* | 1,72E-41 | 3,58E-40 | 10,4 |  | 6,1 | 6,66E-19 | 6,63E-18 |
| *BDNF* | 2,04E-41 | 4,23E-40 | -4,3 |  | 2,5 | 1,76E-05 | 5,19E-05 |
| *MITD1* | 2,59E-41 | 5,38E-40 | 7,7 |  | 1,5 | 1,10E-09 | 5,33E-09 |
| *RIOK3* | 3,46E-41 | 7,18E-40 | 4,4 |  | 3,3 | 3,27E-28 | 5,34E-27 |
| *RND3* | 4,19E-41 | 8,66E-40 | 3,3 |  | 4,1 | 2,74E-33 | 5,74E-32 |
| *LIF* | 7,16E-41 | 1,48E-39 | 6,9 |  | 9,0 | 1,46E-06 | 4,96E-06 |
| *DOCK4* | 7,39E-41 | 1,53E-39 | 3,7 |  | 2,8 | 1,24E-60 | 5,66E-59 |
| *OXSR1* | 7,84E-41 | 1,61E-39 | 2,5 |  | 2,3 | 2,12E-21 | 2,43E-20 |
| *OTUD4* | 8,54E-41 | 1,76E-39 | 7,1 |  | 3,7 | 5,86E-18 | 5,45E-17 |
| *CCNG1* | 9,31E-41 | 1,91E-39 | -3,2 |  | -5,7 | 4,75E-14 | 3,29E-13 |
| *MOV10* | 9,94E-41 | 2,03E-39 | 5,1 |  | 11,9 | 3,91E-115 | 5,11E-113 |
| *ASS1* | 1,87E-40 | 3,82E-39 | 20,0 |  | 2,1 | 4,70E-18 | 4,41E-17 |
| *GPER1* | 1,95E-40 | 3,97E-39 | -54,3 |  | -5,0 | 7,18E-07 | 2,54E-06 |
| *FXYD5* | 2,48E-40 | 5,05E-39 | 3,8 |  | 3,7 | 1,08E-38 | 2,75E-37 |
| *SLC2A12* | 2,93E-40 | 5,95E-39 | -37,3 |  | -7,0 | 3,30E-08 | 1,37E-07 |
| *SLC11A2* | 5,33E-40 | 1,08E-38 | 3,4 |  | 0,9 | 1,46E-07 | 5,62E-07 |
| *IL18R1* | 5,82E-40 | 1,18E-38 | 81939,2 |  | 87,0 | 1,16E-59 | 5,22E-58 |
| *SLC8A1* | 5,87E-40 | 1,19E-38 | -4,5 |  | -2,2 | 2,95E-01 | 3,65E-01 |
| *SLC4A7* | 9,44E-40 | 1,90E-38 | -4,0 |  | -5,5 | 2,59E-47 | 8,99E-46 |
| *TBX3* | 9,72E-40 | 1,95E-38 | 4,6 |  | 3,8 | 3,43E-21 | 3,88E-20 |
| *LGALS9* | 1,15E-39 | 2,30E-38 | 79389,6 |  | 1040,6 | 9,66E-28 | 1,55E-26 |
| *GPR84* | 1,33E-39 | 2,66E-38 | 76684,7 |  | 7522,6 | 2,10E-41 | 5,89E-40 |
| *CTDSPL* | 2,25E-39 | 4,49E-38 | -8,0 |  | -5,2 | 4,52E-22 | 5,34E-21 |
| *IMPDH2* | 2,45E-39 | 4,87E-38 | -4,4 |  | -10,7 | 1,34E-99 | 1,38E-97 |
| *KRT18* | 2,75E-39 | 5,46E-38 | -5,7 |  | -2,9 | 3,02E-01 | 3,73E-01 |
| *TRIM25* | 4,42E-39 | 8,76E-38 | 6,7 |  | 2,4 | 1,99E-10 | 1,03E-09 |
| *POLR1E* | 4,63E-39 | 9,17E-38 | -6,8 |  | -8,4 | 4,38E-26 | 6,45E-25 |
| *MTHFD2* | 4,93E-39 | 9,76E-38 | 4,1 |  | 2,7 | 2,10E-10 | 1,09E-09 |
| *ARHGAP27* | 5,06E-39 | 1,00E-37 | 25,7 |  | 13,2 | 4,27E-23 | 5,34E-22 |
| *DIAPH2* | 5,18E-39 | 1,02E-37 | 4,4 |  | 1,8 | 9,99E-10 | 4,86E-09 |
| *PCLO* | 5,29E-39 | 1,04E-37 | 46,5 |  | 1164,3 | 2,77E-80 | 2,02E-78 |
| *NT5DC2* | 5,38E-39 | 1,06E-37 | -5,8 |  | -12,8 | 4,78E-25 | 6,69E-24 |
| *SULF2* | 5,41E-39 | 1,06E-37 | -29,6 |  | -18,6 | 3,00E-13 | 1,96E-12 |
| *ATP10D* | 6,27E-39 | 1,23E-37 | -4,0 |  | -3,3 | 3,83E-08 | 1,57E-07 |
| *MORC3* | 6,30E-39 | 1,23E-37 | 3,6 |  | 2,3 | 4,60E-17 | 3,98E-16 |
| *GRAMD1A* | 6,75E-39 | 1,32E-37 | 9,4 |  | 3,6 | 1,88E-20 | 2,05E-19 |
| *GRIPAP1* | 7,69E-39 | 1,50E-37 | 4,0 |  | 3,0 | 8,17E-29 | 1,37E-27 |
| *WNT5B* | 7,76E-39 | 1,51E-37 | -3,5 |  | 0,5 | 7,32E-02 | 1,08E-01 |
| *CASP10* | 9,73E-39 | 1,89E-37 | 1072,3 |  | 27,0 | 1,77E-73 | 1,11E-71 |
| *STAP2* | 9,94E-39 | 1,93E-37 | 2559,4 |  | 35,2 | 3,14E-08 | 1,31E-07 |
| *WDR45* | 9,95E-39 | 1,93E-37 | 9,0 |  | 1,5 | 7,15E-05 | 1,91E-04 |
| *NBN* | 1,82E-38 | 3,52E-37 | 3,9 |  | 4,3 | 3,06E-57 | 1,30E-55 |
| *VAMP5* | 1,99E-38 | 3,83E-37 | 19,2 |  | 12,5 | 1,37E-32 | 2,76E-31 |
| *OSR1* | 2,12E-38 | 4,08E-37 | -1788,2 |  | -6,5 | 4,12E-06 | 1,32E-05 |
| *NAB1* | 3,85E-38 | 7,40E-37 | 5,3 |  | 1,5 | 3,25E-10 | 1,65E-09 |
| *CAMK2D* | 4,23E-38 | 8,13E-37 | -2,9 |  | 0,3 | 2,17E-02 | 3,68E-02 |
| *C1S* | 4,37E-38 | 8,37E-37 | 35545,1 |  | 16,3 | 4,41E-06 | 1,41E-05 |
| *POGLUT2* | 4,68E-38 | 8,96E-37 | -7,8 |  | -4,5 | 1,04E-04 | 2,69E-04 |
| *LRCH1* | 5,61E-38 | 1,07E-36 | 7,3 |  | 4,8 | 1,17E-24 | 1,60E-23 |
| *DNPEP* | 6,39E-38 | 1,22E-36 | 3,9 |  | 3,5 | 2,63E-30 | 4,67E-29 |
| *COLEC12* | 6,41E-38 | 1,22E-36 | -6,0 |  | -34,2 | 3,43E-123 | 4,90E-121 |
| *MTSS1* | 6,55E-38 | 1,25E-36 | 19,7 |  | -3,1 | 2,90E-02 | 4,75E-02 |
| *COL27A1* | 1,15E-37 | 2,19E-36 | 7,5 |  | -2,6 | 2,47E-02 | 4,13E-02 |
| *MRAS* | 1,27E-37 | 2,42E-36 | 4,6 |  | 0,7 | 1,37E-05 | 4,10E-05 |
| *FAM171B* | 1,38E-37 | 2,61E-36 | -6,6 |  | -23,3 | 3,02E-65 | 1,55E-63 |
| *LACTB* | 1,56E-37 | 2,95E-36 | 5,2 |  | 1,8 | 3,45E-17 | 3,01E-16 |
| *PVR* | 1,89E-37 | 3,57E-36 | 6,1 |  | 3,4 | 1,34E-09 | 6,42E-09 |
| *CDK17* | 1,90E-37 | 3,58E-36 | 3,5 |  | 1,4 | 1,91E-09 | 9,04E-09 |
| *PODXL* | 2,07E-37 | 3,89E-36 | 12,4 |  | 4,9 | 4,10E-19 | 4,14E-18 |
| *SNHG29* | 2,10E-37 | 3,94E-36 | -3,7 |  | -9,9 | 2,55E-69 | 1,44E-67 |
| *USP15* | 2,26E-37 | 4,23E-36 | 3,1 |  | 2,3 | 4,35E-16 | 3,50E-15 |
| *KCNK3* | 3,14E-37 | 5,87E-36 | -13,7 |  | -10,6 | 1,15E-02 | 2,07E-02 |
| *CCL7* | 4,54E-37 | 8,48E-36 | 1623,6 |  | 547,0 | 4,72E-112 | 5,85E-110 |
| *IKZF2* | 4,85E-37 | 9,05E-36 | 6,9 |  | 2,2 | 5,71E-07 | 2,04E-06 |
| *FMNL3* | 4,97E-37 | 9,25E-36 | 3,0 |  | 1,2 | 2,55E-11 | 1,43E-10 |
| *SLC16A6* | 5,84E-37 | 1,09E-35 | 27,0 |  | -2,9 | 1,78E-01 | 2,37E-01 |
| *BAG2* | 5,87E-37 | 1,09E-35 | -6,4 |  | -3,7 | 7,60E-12 | 4,49E-11 |
| *THSD1* | 7,15E-37 | 1,32E-35 | 10,6 |  | 1,6 | 3,92E-07 | 1,43E-06 |
| *PRKD3* | 7,76E-37 | 1,44E-35 | 3,5 |  | 0,5 | 9,93E-04 | 2,18E-03 |
| *CLSTN2* | 8,06E-37 | 1,49E-35 | -34,4 |  | -27,8 | 8,91E-11 | 4,76E-10 |
| *SLC12A7* | 9,50E-37 | 1,75E-35 | 50888,2 |  | 38,2 | 1,14E-24 | 1,56E-23 |
| *S1PR3* | 9,63E-37 | 1,77E-35 | 9,6 |  | 2,6 | 7,69E-05 | 2,04E-04 |
| *LINC01539* | 1,02E-36 | 1,87E-35 | 61613,6 |  | 4944,6 | 3,44E-31 | 6,45E-30 |
| *PPA1* | 1,03E-36 | 1,89E-35 | 3,8 |  | 3,9 | 2,25E-17 | 2,00E-16 |
| *RFLNB* | 1,13E-36 | 2,07E-35 | -5,9 |  | -11,0 | 3,07E-32 | 6,09E-31 |
| *SNX18* | 1,18E-36 | 2,15E-35 | -3,9 |  | -4,0 | 4,70E-28 | 7,63E-27 |
| *PLAAT4* | 1,27E-36 | 2,31E-35 | 40439,5 |  | 102,1 | 6,32E-16 | 5,03E-15 |
| *FAT3* | 1,34E-36 | 2,44E-35 | -5,7 |  | -7,2 | 1,09E-05 | 3,31E-05 |
| *CYB5A* | 1,83E-36 | 3,32E-35 | 4,0 |  | 4,4 | 5,40E-46 | 1,79E-44 |
| *MKNK2* | 1,83E-36 | 3,33E-35 | 3,2 |  | 4,2 | 1,41E-46 | 4,81E-45 |
| *PRKAG2* | 2,00E-36 | 3,63E-35 | -4,1 |  | 1,3 | 1,87E-03 | 3,93E-03 |
| *ENSG00000253838* | 2,27E-36 | 4,10E-35 | 49154,3 |  | 32034,3 | 1,39E-32 | 2,80E-31 |
| *ZNF106* | 3,02E-36 | 5,45E-35 | -3,1 |  | -7,9 | 3,25E-23 | 4,09E-22 |
| *PSMB9* | 4,44E-36 | 8,00E-35 | 1852,1 |  | 74,3 | 2,45E-67 | 1,33E-65 |
| *PLA2G12A* | 4,74E-36 | 8,54E-35 | -9,8 |  | -14,1 | 1,06E-38 | 2,71E-37 |
| *CRISPLD1* | 7,04E-36 | 1,27E-34 | 12,2 |  | -2,3 | 7,16E-01 | 7,65E-01 |
| *SERPINB8* | 7,21E-36 | 1,29E-34 | 5,1 |  | 4,5 | 5,90E-37 | 1,42E-35 |
| *IL18BP* | 7,32E-36 | 1,31E-34 | 292,9 |  | 332,9 | 4,24E-128 | 6,35E-126 |
| *ROBO1* | 9,73E-36 | 1,74E-34 | 4,1 |  | 0,7 | 3,46E-06 | 1,12E-05 |
| *MAN1A1* | 1,22E-35 | 2,19E-34 | 4,1 |  | 0,1 | 7,93E-01 | 8,32E-01 |
| *PFN2* | 1,35E-35 | 2,42E-34 | -2,3 |  | 0,3 | 1,45E-01 | 1,98E-01 |
| *PARP4* | 1,40E-35 | 2,50E-34 | 2,6 |  | 0,6 | 3,82E-04 | 9,04E-04 |
| *CLCA2* | 1,59E-35 | 2,82E-34 | -148,1 |  | -217,3 | 8,82E-13 | 5,59E-12 |
| *HTATIP2* | 1,97E-35 | 3,49E-34 | 4,4 |  | 2,7 | 4,28E-35 | 9,68E-34 |
| *TENM3* | 2,04E-35 | 3,61E-34 | -5,0 |  | -13,8 | 2,40E-21 | 2,74E-20 |
| *RALGPS2* | 2,20E-35 | 3,88E-34 | 4,2 |  | 3,7 | 1,58E-17 | 1,42E-16 |
| *CEACAM1* | 3,31E-35 | 5,85E-34 | 47743,3 |  | 1502,0 | 2,07E-23 | 2,62E-22 |
| *OLFML3* | 3,36E-35 | 5,93E-34 | -5,8 |  | -3,1 | 1,52E-10 | 7,98E-10 |
| *KLF13* | 3,41E-35 | 6,00E-34 | 3,7 |  | 0,1 | 6,69E-01 | 7,24E-01 |
| *ENSG00000273132* | 4,92E-35 | 8,64E-34 | 195,8 |  | 1479,8 | 3,48E-19 | 3,53E-18 |
| *COL4A2* | 5,33E-35 | 9,34E-34 | 5,9 |  | 0,5 | 6,57E-03 | 1,24E-02 |
| *MALT1* | 5,62E-35 | 9,84E-34 | 4,6 |  | 0,9 | 2,62E-03 | 5,35E-03 |
| *BST2* | 5,65E-35 | 9,89E-34 | 125085,3 |  | 1048,1 | 1,04E-157 | 2,20E-155 |
| *EPS8* | 7,37E-35 | 1,29E-33 | -4,0 |  | -3,2 | 4,49E-04 | 1,05E-03 |
| *ZEB2* | 7,89E-35 | 1,37E-33 | 2,9 |  | 0,9 | 2,13E-10 | 1,10E-09 |
| *RHOBTB3* | 9,54E-35 | 1,66E-33 | 2,3 |  | -2,8 | 3,63E-04 | 8,62E-04 |
| *BPGM* | 9,97E-35 | 1,73E-33 | 26,1 |  | 1,1 | 5,15E-05 | 1,41E-04 |
| *CYB5R2* | 1,24E-34 | 2,15E-33 | 10,4 |  | 34,2 | 4,05E-32 | 7,97E-31 |
| *FNIP2* | 1,25E-34 | 2,16E-33 | 5,1 |  | 1,1 | 7,50E-06 | 2,33E-05 |
| *PLP2* | 1,90E-34 | 3,28E-33 | 3,3 |  | 2,1 | 1,95E-24 | 2,60E-23 |
| *FTH1* | 2,42E-34 | 4,18E-33 | 4,8 |  | 1,9 | 2,55E-15 | 1,93E-14 |
| *PM20D2* | 2,54E-34 | 4,37E-33 | -5,0 |  | -2,4 | 4,35E-01 | 5,07E-01 |
| *MCTP2* | 2,76E-34 | 4,74E-33 | 7,0 |  | -3,5 | 1,01E-02 | 1,83E-02 |
| *MIR34AHG* | 3,01E-34 | 5,16E-33 | 4,8 |  | 0,3 | 3,82E-01 | 4,54E-01 |
| *FAM177A1* | 3,07E-34 | 5,27E-33 | 5,4 |  | 4,7 | 4,51E-50 | 1,63E-48 |
| *WASF3* | 3,10E-34 | 5,30E-33 | -4,9 |  | -5,1 | 5,15E-18 | 4,80E-17 |
| *NT5DC3* | 3,23E-34 | 5,53E-33 | -8,7 |  | -3,9 | 1,30E-05 | 3,91E-05 |
| *NUDT4* | 3,60E-34 | 6,15E-33 | -3,4 |  | -5,0 | 2,70E-19 | 2,77E-18 |
| *SOCS1* | 3,65E-34 | 6,22E-33 | 18,2 |  | 76,0 | 5,19E-65 | 2,65E-63 |
| *SYNGR2* | 3,65E-34 | 6,22E-33 | 6,1 |  | 11,2 | 1,10E-50 | 4,05E-49 |
| *LYPD6* | 4,51E-34 | 7,67E-33 | 98,0 |  | 7,1 | 3,20E-18 | 3,04E-17 |
| *F3* | 4,85E-34 | 8,22E-33 | 5,1 |  | 19,1 | 6,85E-09 | 3,05E-08 |
| *EGFR* | 5,29E-34 | 8,96E-33 | 5,5 |  | 0,7 | 8,76E-06 | 2,70E-05 |
| *LDB2* | 5,33E-34 | 9,02E-33 | -26,8 |  | -7,6 | 2,11E-24 | 2,81E-23 |
| *AIFM2* | 5,84E-34 | 9,87E-33 | 10,4 |  | 6,1 | 2,17E-26 | 3,24E-25 |
| *HLA-DRB5* | 7,39E-34 | 1,25E-32 | 34912,2 |  | 26,6 | 1,88E-09 | 8,92E-09 |
| *S100A11* | 7,98E-34 | 1,35E-32 | 3,0 |  | 1,9 | 3,62E-16 | 2,93E-15 |
| *TET1* | 8,77E-34 | 1,48E-32 | -10,8 |  | -14,8 | 9,88E-16 | 7,70E-15 |
| *TNFRSF21* | 9,72E-34 | 1,63E-32 | -5,5 |  | 0,1 | 8,64E-01 | 8,92E-01 |
| *SGIP1* | 1,24E-33 | 2,07E-32 | -8,0 |  | -3,7 | 1,42E-03 | 3,05E-03 |
| *PFKFB3* | 1,26E-33 | 2,10E-32 | 4,5 |  | 11,3 | 4,36E-14 | 3,03E-13 |
| *HLA-DOA* | 1,41E-33 | 2,36E-32 | 35057,8 |  | 13,0 | 2,02E-04 | 4,98E-04 |
| *FRMD5* | 1,42E-33 | 2,37E-32 | -11,0 |  | -2,7 | 1,02E-04 | 2,65E-04 |
| *LOXL1* | 1,72E-33 | 2,86E-32 | -3,0 |  | -5,0 | 3,22E-08 | 1,34E-07 |
| *AR* | 2,46E-33 | 4,10E-32 | -13,8 |  | -31,7 | 1,10E-04 | 2,84E-04 |
| *JAM2* | 2,53E-33 | 4,20E-32 | 23,2 |  | 5,5 | 9,03E-09 | 3,97E-08 |
| *NEDD4L* | 2,60E-33 | 4,31E-32 | 4,0 |  | 2,3 | 1,56E-09 | 7,43E-09 |
| *PSMB10* | 2,75E-33 | 4,56E-32 | 63,6 |  | 55,6 | 6,25E-93 | 5,48E-91 |
| *SYNE1* | 3,13E-33 | 5,19E-32 | 5,0 |  | 0,5 | 5,60E-03 | 1,07E-02 |
| *TSPAN13* | 3,77E-33 | 6,23E-32 | -5,1 |  | -4,9 | 2,84E-02 | 4,67E-02 |
| *FAR2* | 4,07E-33 | 6,72E-32 | 7,3 |  | 2,5 | 1,65E-10 | 8,61E-10 |
| *RUNX3* | 4,80E-33 | 7,91E-32 | 14,6 |  | 4,0 | 6,59E-07 | 2,34E-06 |
| *CTHRC1* | 4,84E-33 | 7,96E-32 | 22,5 |  | -3,3 | 1,98E-15 | 1,51E-14 |
| *NDUFAF8* | 5,11E-33 | 8,40E-32 | -5,4 |  | -10,6 | 2,46E-29 | 4,22E-28 |
| *CMBL* | 5,33E-33 | 8,75E-32 | -14,7 |  | -27,2 | 3,93E-32 | 7,76E-31 |
| *RBCK1* | 6,19E-33 | 1,01E-31 | 3,7 |  | 3,8 | 1,25E-31 | 2,40E-30 |
| *PTPN2* | 6,75E-33 | 1,11E-31 | 3,9 |  | 3,9 | 2,28E-23 | 2,87E-22 |
| *MCUB* | 7,71E-33 | 1,26E-31 | 5,6 |  | 5,5 | 4,55E-20 | 4,82E-19 |
| *ALDH4A1* | 9,08E-33 | 1,48E-31 | -5,9 |  | -7,0 | 1,21E-17 | 1,10E-16 |
| *HNMT* | 9,31E-33 | 1,52E-31 | 131,2 |  | 1,3 | 5,64E-10 | 2,81E-09 |
| *UNC119B* | 1,11E-32 | 1,81E-31 | -4,8 |  | -13,2 | 9,95E-43 | 2,97E-41 |
| *MGLL* | 1,12E-32 | 1,83E-31 | 4,6 |  | 9,5 | 1,32E-23 | 1,69E-22 |
| *BATF2* | 1,20E-32 | 1,95E-31 | 3806,6 |  | 6233,2 | 2,45E-72 | 1,49E-70 |
| *KLF10* | 1,38E-32 | 2,23E-31 | 4,2 |  | 1,4 | 2,86E-08 | 1,19E-07 |
| *SC5D* | 1,46E-32 | 2,37E-31 | -3,6 |  | -2,5 | 1,14E-02 | 2,05E-02 |
| *PTTG1IP* | 1,51E-32 | 2,44E-31 | 4,6 |  | 1,9 | 2,03E-20 | 2,20E-19 |
| *TXNIP* | 1,53E-32 | 2,47E-31 | 3,2 |  | 3,0 | 3,32E-19 | 3,37E-18 |
| *NT5E* | 1,59E-32 | 2,56E-31 | 2,3 |  | 2,0 | 3,15E-20 | 3,38E-19 |
| *TMEM165* | 1,60E-32 | 2,58E-31 | 3,5 |  | 2,9 | 7,83E-15 | 5,72E-14 |
| *PEG10* | 1,73E-32 | 2,78E-31 | -9,4 |  | -10,0 | 2,16E-06 | 7,18E-06 |
| *P2RX4* | 1,90E-32 | 3,04E-31 | 12,2 |  | 7,5 | 7,58E-56 | 3,14E-54 |
| *LINC02605* | 1,95E-32 | 3,12E-31 | 37843,6 |  | 13,5 | 7,23E-05 | 1,93E-04 |
| *PFKP* | 2,04E-32 | 3,26E-31 | 3,6 |  | 9,3 | 5,68E-09 | 2,55E-08 |
| *CCL20* | 2,05E-32 | 3,28E-31 | 15684,3 |  | 3340,8 | 1,51E-59 | 6,72E-58 |
| *TGIF1* | 2,11E-32 | 3,36E-31 | 3,9 |  | 4,9 | 4,74E-32 | 9,29E-31 |
| *SLC9B2* | 2,62E-32 | 4,18E-31 | 4,7 |  | 3,3 | 1,53E-32 | 3,08E-31 |
| *SMO* | 3,01E-32 | 4,79E-31 | -14,3 |  | -18,5 | 8,91E-16 | 7,00E-15 |
| *PTGES3P1* | 3,03E-32 | 4,82E-31 | 358,3 |  | 59,6 | 7,59E-31 | 1,39E-29 |
| *PIM3* | 3,10E-32 | 4,92E-31 | 4,8 |  | 9,8 | 5,02E-64 | 2,54E-62 |
| *SAMD4A* | 3,28E-32 | 5,20E-31 | 3,0 |  | 1,2 | 6,48E-08 | 2,61E-07 |
| *ATP6V1B2* | 3,29E-32 | 5,21E-31 | 2,9 |  | 1,9 | 1,46E-35 | 3,37E-34 |
| *PGK1* | 3,41E-32 | 5,39E-31 | 3,2 |  | 7,0 | 5,52E-13 | 3,54E-12 |
| *PACSIN3* | 3,41E-32 | 5,39E-31 | -6,0 |  | -9,2 | 8,37E-08 | 3,33E-07 |
| *NLRP10* | 3,56E-32 | 5,61E-31 | -7,2 |  | 2,6 | 4,02E-04 | 9,49E-04 |
| *MAGED1* | 4,24E-32 | 6,67E-31 | -3,3 |  | -3,9 | 3,15E-12 | 1,91E-11 |
| *ADGRL2* | 4,54E-32 | 7,15E-31 | -3,0 |  | -2,1 | 3,22E-01 | 3,93E-01 |
| *COL4A5* | 5,33E-32 | 8,37E-31 | -8,8 |  | -22,2 | 1,95E-12 | 1,20E-11 |
| *PHACTR1* | 5,37E-32 | 8,43E-31 | 16,0 |  | 33,9 | 1,76E-12 | 1,09E-11 |
| *RNF141* | 5,54E-32 | 8,69E-31 | -4,2 |  | -5,6 | 6,32E-16 | 5,03E-15 |
| *BNC2* | 6,00E-32 | 9,40E-31 | -7,3 |  | -4,1 | 3,69E-20 | 3,94E-19 |
| *SALL2* | 6,17E-32 | 9,64E-31 | -193,3 |  | -7,1 | 2,18E-05 | 6,35E-05 |
| *MSRB1* | 6,20E-32 | 9,68E-31 | 10,4 |  | 1,3 | 2,60E-07 | 9,74E-07 |
| *PLXNC1* | 6,60E-32 | 1,03E-30 | 69,9 |  | 2,0 | 2,16E-03 | 4,50E-03 |
| *NTNG1* | 7,08E-32 | 1,10E-30 | -4,0 |  | -3,6 | 3,17E-05 | 9,00E-05 |
| *TP53BP2* | 7,40E-32 | 1,15E-30 | 3,2 |  | 3,4 | 1,26E-26 | 1,91E-25 |
| *EPS8L1* | 7,75E-32 | 1,20E-30 | 98,5 |  | 154,4 | 4,48E-13 | 2,90E-12 |
| *APBA2* | 9,00E-32 | 1,40E-30 | 3,5 |  | 11,0 | 1,51E-08 | 6,48E-08 |
| *MAP7D3* | 9,25E-32 | 1,43E-30 | -3,3 |  | -2,3 | 5,48E-03 | 1,05E-02 |
| *NECTIN2* | 9,54E-32 | 1,48E-30 | 3,2 |  | 14,1 | 1,03E-42 | 3,04E-41 |
| *KRT19* | 1,15E-31 | 1,77E-30 | -5,8 |  | 1,6 | 4,79E-03 | 9,28E-03 |
| *TMCO3* | 1,20E-31 | 1,85E-30 | -2,6 |  | -4,5 | 2,23E-28 | 3,67E-27 |
| *LACC1* | 1,33E-31 | 2,05E-30 | 6,9 |  | -2,5 | 6,20E-02 | 9,36E-02 |
| *STAT3* | 1,54E-31 | 2,36E-30 | 3,5 |  | 1,5 | 3,39E-09 | 1,56E-08 |
| *POU2F2* | 1,55E-31 | 2,38E-30 | 18,5 |  | 82,5 | 1,88E-29 | 3,23E-28 |
| *CACHD1* | 1,86E-31 | 2,85E-30 | 7,1 |  | 2,9 | 1,26E-05 | 3,79E-05 |
| *ID2* | 1,89E-31 | 2,89E-30 | -7,8 |  | 3,8 | 1,15E-15 | 8,94E-15 |
| *PDIA3* | 1,95E-31 | 2,98E-30 | 2,3 |  | 3,7 | 7,23E-16 | 5,72E-15 |
| *VWA5A* | 2,08E-31 | 3,18E-30 | -21,6 |  | -4,1 | 1,48E-06 | 5,02E-06 |
| *IFIT1* | 2,39E-31 | 3,65E-30 | 8,0 |  | 433,4 | 7,33E-109 | 8,65E-107 |
| *F2R* | 2,44E-31 | 3,71E-30 | -2,9 |  | -9,8 | 5,12E-20 | 5,39E-19 |
| *TCAF2* | 2,54E-31 | 3,87E-30 | 8,9 |  | 25,0 | 4,37E-26 | 6,45E-25 |
| *HS3ST3B1* | 2,71E-31 | 4,11E-30 | 4,2 |  | 5,7 | 1,96E-40 | 5,32E-39 |
| *B3GNT2* | 3,28E-31 | 4,98E-30 | 6,8 |  | 5,5 | 2,05E-82 | 1,53E-80 |
| *TMEM119* | 3,29E-31 | 4,98E-30 | -17,3 |  | -24,6 | 5,37E-51 | 1,99E-49 |
| *GCA* | 3,39E-31 | 5,13E-30 | 51,3 |  | 142,6 | 3,77E-18 | 3,55E-17 |
| *HDGF* | 3,62E-31 | 5,47E-30 | 2,9 |  | 1,8 | 9,92E-21 | 1,09E-19 |
| *SNED1* | 3,67E-31 | 5,54E-30 | -15,1 |  | -29,4 | 1,86E-70 | 1,09E-68 |
| *PRKAR2A* | 3,90E-31 | 5,89E-30 | -2,7 |  | -4,8 | 2,03E-17 | 1,81E-16 |
| *PARD3* | 4,56E-31 | 6,87E-30 | 2,9 |  | 2,0 | 1,17E-09 | 5,64E-09 |
| *ENSG00000283265* | 4,75E-31 | 7,15E-30 | 22319,4 |  | 939,9 | 2,26E-37 | 5,52E-36 |
| *PALM2AKAP2* | 5,11E-31 | 7,68E-30 | 2,7 |  | 3,7 | 6,02E-44 | 1,84E-42 |
| *TINF2* | 5,52E-31 | 8,28E-30 | 4,3 |  | 1,4 | 5,78E-09 | 2,59E-08 |
| *APOBEC3G* | 6,02E-31 | 9,03E-30 | 378,4 |  | 14,5 | 3,55E-35 | 8,07E-34 |
| *S1PR2* | 6,87E-31 | 1,03E-29 | 4,1 |  | 2,6 | 9,06E-16 | 7,09E-15 |
| *DPYSL4* | 7,21E-31 | 1,08E-29 | 12,5 |  | 26,0 | 1,62E-29 | 2,79E-28 |
| *GTPBP1* | 8,06E-31 | 1,20E-29 | 4,2 |  | 10,6 | 3,39E-66 | 1,78E-64 |
| *HK1* | 8,55E-31 | 1,28E-29 | 3,5 |  | 1,9 | 1,07E-08 | 4,66E-08 |
| *BANCR* | 8,98E-31 | 1,34E-29 | 22585,3 |  | 18127,1 | 4,39E-29 | 7,43E-28 |
| *MX2* | 9,12E-31 | 1,36E-29 | 30521,2 |  | 393,0 | 4,16E-45 | 1,33E-43 |
| *GDF5* | 1,11E-30 | 1,64E-29 | -29,5 |  | -39,1 | 4,25E-58 | 1,85E-56 |
| *SNRPD3* | 1,13E-30 | 1,67E-29 | -4,2 |  | -5,8 | 5,36E-13 | 3,44E-12 |
| *GYPC* | 1,50E-30 | 2,22E-29 | 3,6 |  | 4,1 | 3,92E-23 | 4,92E-22 |
| *CALHM6* | 1,53E-30 | 2,26E-29 | 457,9 |  | 13651,1 | 1,07E-30 | 1,95E-29 |
| *ZNF697* | 1,54E-30 | 2,28E-29 | 3,5 |  | 0,7 | 1,16E-03 | 2,52E-03 |
| *BCL2L14* | 1,57E-30 | 2,32E-29 | 31067,5 |  | 31844,0 | 8,98E-33 | 1,82E-31 |
| *SERPINE1* | 1,65E-30 | 2,43E-29 | 2,6 |  | 9,4 | 1,83E-04 | 4,56E-04 |
| *GALNT11* | 1,89E-30 | 2,79E-29 | -3,8 |  | -5,3 | 7,97E-35 | 1,79E-33 |
| *EFL1* | 1,97E-30 | 2,89E-29 | 4,3 |  | 2,2 | 3,86E-13 | 2,51E-12 |
| *IRF1-AS1* | 2,48E-30 | 3,64E-29 | 60,0 |  | 14,0 | 8,04E-35 | 1,81E-33 |
| *EML4* | 2,80E-30 | 4,11E-29 | 2,9 |  | 0,0 | 7,56E-01 | 8,00E-01 |
| *LRP10* | 2,94E-30 | 4,31E-29 | 3,4 |  | 2,5 | 6,42E-23 | 7,95E-22 |
| *IGF2BP3* | 3,07E-30 | 4,49E-29 | -2,5 |  | -4,6 | 3,93E-09 | 1,79E-08 |
| *IL3RA* | 3,33E-30 | 4,86E-29 | 17,1 |  | 344,6 | 4,04E-11 | 2,23E-10 |
| *SERPINA1* | 4,07E-30 | 5,93E-29 | 6127,0 |  | 17,3 | 7,54E-15 | 5,52E-14 |
| *MT1M* | 4,46E-30 | 6,50E-29 | 822,0 |  | 1292,6 | 3,86E-35 | 8,76E-34 |
| *F2RL2* | 5,72E-30 | 8,32E-29 | -4,6 |  | -25,3 | 6,40E-10 | 3,17E-09 |
| *NPC1* | 6,81E-30 | 9,90E-29 | 4,2 |  | 0,2 | 1,63E-01 | 2,19E-01 |
| *UHMK1* | 8,69E-30 | 1,26E-28 | 2,8 |  | 2,5 | 9,60E-11 | 5,11E-10 |
| *SMURF1* | 9,37E-30 | 1,36E-28 | 3,3 |  | 3,3 | 4,58E-31 | 8,52E-30 |
| *GLT8D2* | 9,92E-30 | 1,44E-28 | -4,5 |  | -26,3 | 4,40E-84 | 3,40E-82 |
| *TTC39C* | 1,01E-29 | 1,46E-28 | 3,9 |  | -2,8 | 2,11E-05 | 6,15E-05 |
| *EI24* | 1,10E-29 | 1,59E-28 | -2,9 |  | -2,9 | 1,39E-05 | 4,15E-05 |
| *METRN* | 1,18E-29 | 1,70E-28 | -5,1 |  | -12,3 | 7,71E-29 | 1,29E-27 |
| *GPNMB* | 1,19E-29 | 1,71E-28 | -22,4 |  | -10,7 | 1,55E-23 | 1,97E-22 |
| *CDKN1B* | 1,23E-29 | 1,77E-28 | 4,4 |  | 1,1 | 3,37E-12 | 2,05E-11 |
| *VDR* | 1,23E-29 | 1,77E-28 | 7,1 |  | 1,3 | 2,64E-08 | 1,11E-07 |
| *QPCT* | 1,31E-29 | 1,88E-28 | 4,4 |  | 0,3 | 2,33E-01 | 2,98E-01 |
| *TNFRSF14* | 1,49E-29 | 2,14E-28 | 21128,5 |  | 9,2 | 2,98E-26 | 4,44E-25 |
| *DOCK10* | 1,53E-29 | 2,19E-28 | 4,4 |  | 1,0 | 2,55E-10 | 1,31E-09 |
| *SESN1* | 1,84E-29 | 2,64E-28 | -5,1 |  | -4,3 | 5,50E-07 | 1,97E-06 |
| *CXCL5* | 1,94E-29 | 2,77E-28 | 27656,3 |  | 23868,3 | 1,50E-69 | 8,52E-68 |
| *MIB1* | 2,60E-29 | 3,71E-28 | -3,4 |  | -5,4 | 3,38E-18 | 3,20E-17 |
| *PLAUR* | 2,87E-29 | 4,09E-28 | 3,1 |  | 2,5 | 2,01E-06 | 6,72E-06 |
| *OGFR* | 2,99E-29 | 4,26E-28 | 3,6 |  | 6,9 | 1,12E-61 | 5,28E-60 |
| *SPPL2A* | 3,04E-29 | 4,33E-28 | 3,2 |  | 2,2 | 2,86E-19 | 2,93E-18 |
| *INPP5A* | 3,18E-29 | 4,51E-28 | -3,6 |  | -2,6 | 3,77E-02 | 5,99E-02 |
| *RRAS2* | 3,44E-29 | 4,88E-28 | -2,8 |  | 0,8 | 1,52E-03 | 3,25E-03 |
| *DNAJB4* | 4,55E-29 | 6,45E-28 | -6,0 |  | -2,7 | 2,55E-02 | 4,24E-02 |
| *ECPAS* | 5,17E-29 | 7,32E-28 | 2,5 |  | 0,2 | 1,62E-01 | 2,18E-01 |
| *ZNF792* | 7,51E-29 | 1,06E-27 | 10,0 |  | 0,4 | 3,40E-01 | 4,12E-01 |
| *ADI1* | 7,75E-29 | 1,10E-27 | -3,3 |  | -3,5 | 2,95E-15 | 2,22E-14 |
| *FAM8A1* | 8,21E-29 | 1,16E-27 | 3,2 |  | 6,1 | 4,72E-24 | 6,20E-23 |
| *KARS1* | 8,41E-29 | 1,19E-27 | 2,8 |  | 2,1 | 2,46E-26 | 3,67E-25 |
| *COPRS* | 8,77E-29 | 1,24E-27 | -3,9 |  | -4,1 | 9,23E-17 | 7,84E-16 |
| *SMAD7* | 9,66E-29 | 1,36E-27 | 5,3 |  | 0,7 | 1,47E-04 | 3,71E-04 |
| *ZMAT3* | 9,68E-29 | 1,36E-27 | -4,7 |  | -10,1 | 8,24E-35 | 1,85E-33 |
| *GFPT2* | 1,12E-28 | 1,57E-27 | 5,7 |  | 0,1 | 5,37E-01 | 6,05E-01 |
| *NEK10* | 1,13E-28 | 1,57E-27 | -20,4 |  | -21,9 | 5,94E-27 | 9,17E-26 |
| *RFX5* | 1,15E-28 | 1,60E-27 | 3,8 |  | 9,6 | 4,27E-158 | 9,19E-156 |
| *ARL4D* | 1,15E-28 | 1,60E-27 | -5,2 |  | -2,6 | 6,45E-02 | 9,70E-02 |
| *ZNF470* | 1,19E-28 | 1,65E-27 | 7,2 |  | 4,2 | 1,15E-22 | 1,41E-21 |
| *CCDC82* | 1,19E-28 | 1,65E-27 | 7,1 |  | 0,3 | 2,67E-01 | 3,35E-01 |
| *HMGCR* | 1,20E-28 | 1,66E-27 | -3,1 |  | -2,2 | 3,16E-01 | 3,87E-01 |
| *BTBD3* | 1,20E-28 | 1,67E-27 | -3,9 |  | -3,7 | 4,55E-07 | 1,65E-06 |
| *SLC6A8* | 1,22E-28 | 1,69E-27 | -5,6 |  | -2,9 | 6,99E-03 | 1,31E-02 |
| *COL1A2* | 1,70E-28 | 2,36E-27 | -3,8 |  | -4,2 | 1,71E-14 | 1,22E-13 |
| *NEMP1* | 1,72E-28 | 2,38E-27 | 4,1 |  | 16,7 | 6,17E-180 | 1,65E-177 |
| *FUT8* | 1,73E-28 | 2,39E-27 | -2,5 |  | -3,8 | 1,20E-10 | 6,34E-10 |
| *RAP2B* | 1,82E-28 | 2,52E-27 | -2,9 |  | -6,6 | 1,30E-35 | 3,00E-34 |
| *AMOT* | 1,99E-28 | 2,75E-27 | -22,8 |  | -8,5 | 3,00E-12 | 1,83E-11 |
| *STEAP2* | 2,43E-28 | 3,35E-27 | 5,3 |  | 2,1 | 1,85E-10 | 9,62E-10 |
| *PCED1A* | 2,60E-28 | 3,58E-27 | 5,0 |  | 0,4 | 1,91E-01 | 2,52E-01 |
| *ARHGAP31* | 2,63E-28 | 3,62E-27 | 5,1 |  | -2,0 | 9,33E-01 | 9,48E-01 |
| *NIPA1* | 2,78E-28 | 3,82E-27 | 6,6 |  | 3,0 | 3,70E-13 | 2,41E-12 |
| *VPS26B* | 2,81E-28 | 3,85E-27 | -2,8 |  | -3,2 | 7,72E-08 | 3,08E-07 |
| *SEC24A* | 2,87E-28 | 3,93E-27 | 4,8 |  | 3,8 | 4,06E-33 | 8,36E-32 |
| *P4HB* | 3,22E-28 | 4,41E-27 | 2,2 |  | 1,3 | 4,99E-12 | 3,00E-11 |
| *EIF2AK4* | 3,84E-28 | 5,26E-27 | -2,3 |  | -3,0 | 4,81E-11 | 2,62E-10 |
| *ENPP1* | 4,14E-28 | 5,66E-27 | -2,9 |  | -5,8 | 7,89E-24 | 1,02E-22 |
| *CCNY* | 5,21E-28 | 7,11E-27 | -2,8 |  | -3,0 | 4,18E-05 | 1,16E-04 |
| *CTNNBIP1* | 5,24E-28 | 7,14E-27 | -6,1 |  | -4,8 | 1,07E-11 | 6,25E-11 |
| *BLZF1* | 5,61E-28 | 7,64E-27 | 3,5 |  | 5,7 | 7,31E-58 | 3,15E-56 |
| *ARL6IP5* | 6,81E-28 | 9,27E-27 | 3,1 |  | 0,1 | 2,36E-01 | 3,01E-01 |
| *RBMS1* | 6,86E-28 | 9,32E-27 | 2,7 |  | 0,6 | 7,58E-04 | 1,70E-03 |
| *TMEM170A* | 7,44E-28 | 1,01E-26 | 5,7 |  | 4,8 | 2,32E-33 | 4,88E-32 |
| *S1PR1* | 8,05E-28 | 1,09E-26 | -28,8 |  | -160,4 | 6,11E-14 | 4,20E-13 |
| *TRIM24* | 8,33E-28 | 1,13E-26 | 5,1 |  | 0,6 | 3,43E-03 | 6,84E-03 |
| *BEND6* | 8,34E-28 | 1,13E-26 | -7,4 |  | -28,8 | 4,59E-27 | 7,16E-26 |
| *PPP1R13L* | 9,43E-28 | 1,27E-26 | 31,8 |  | 6,9 | 8,15E-10 | 4,00E-09 |
| *SGCB* | 9,76E-28 | 1,32E-26 | 4,4 |  | 3,0 | 1,04E-26 | 1,58E-25 |
| *TIPARP* | 1,02E-27 | 1,38E-26 | 2,9 |  | 7,9 | 8,51E-21 | 9,43E-20 |
| *SGK1* | 1,04E-27 | 1,40E-26 | -3,2 |  | -2,5 | 7,42E-02 | 1,10E-01 |
| *ZDHHC23* | 1,07E-27 | 1,44E-26 | 19,7 |  | 13,0 | 4,65E-30 | 8,16E-29 |
| *SPECC1* | 1,18E-27 | 1,58E-26 | 4,3 |  | -2,5 | 1,70E-02 | 2,95E-02 |
| *KCNJ15* | 1,22E-27 | 1,64E-26 | 43,5 |  | 0,9 | 1,23E-02 | 2,18E-02 |
| *ENSG00000267607* | 1,31E-27 | 1,76E-26 | 16125,8 |  | 976,6 | 7,35E-15 | 5,38E-14 |
| *MLLT11* | 1,32E-27 | 1,77E-26 | 2,5 |  | 1,3 | 4,73E-07 | 1,71E-06 |
| *RHOJ* | 1,37E-27 | 1,83E-26 | -11,6 |  | -35,6 | 6,47E-16 | 5,14E-15 |
| *CEBPD* | 1,39E-27 | 1,86E-26 | 9,2 |  | -2,0 | 9,53E-01 | 9,63E-01 |
| *RBPJ* | 1,53E-27 | 2,04E-26 | 2,9 |  | 0,4 | 2,59E-02 | 4,30E-02 |
| *SERPING1* | 1,53E-27 | 2,04E-26 | 62943,7 |  | 53,6 | 3,67E-09 | 1,68E-08 |
| *TSPAN9* | 1,68E-27 | 2,23E-26 | -8,1 |  | -4,2 | 6,26E-11 | 3,38E-10 |
| *ECE1* | 1,82E-27 | 2,41E-26 | 4,1 |  | 4,7 | 3,94E-19 | 3,98E-18 |
| *SNX9* | 2,31E-27 | 3,06E-26 | 2,5 |  | -3,0 | 4,41E-03 | 8,61E-03 |
| *SRPX2* | 2,63E-27 | 3,47E-26 | -2,9 |  | -2,9 | 9,64E-07 | 3,36E-06 |
| *SLC43A3* | 3,02E-27 | 3,98E-26 | 7,1 |  | 2,6 | 1,34E-15 | 1,03E-14 |
| *PRPF3* | 3,02E-27 | 3,98E-26 | 2,9 |  | 3,1 | 2,03E-34 | 4,43E-33 |
| *GBP5* | 3,07E-27 | 4,04E-26 | 273399,5 |  | 540391,9 | 2,48E-73 | 1,54E-71 |
| *MAP3K8* | 3,16E-27 | 4,15E-26 | 38,2 |  | 4,8 | 1,36E-08 | 5,86E-08 |
| *CD55* | 3,21E-27 | 4,22E-26 | 4,8 |  | 9,0 | 5,23E-31 | 9,69E-30 |
| *PHTF2* | 3,26E-27 | 4,27E-26 | -4,2 |  | -4,4 | 1,14E-10 | 6,05E-10 |
| *CSRNP3* | 3,32E-27 | 4,35E-26 | -79,9 |  | -211,3 | 2,68E-20 | 2,88E-19 |
| *TMEM41B* | 3,41E-27 | 4,46E-26 | 4,8 |  | 2,0 | 2,43E-17 | 2,15E-16 |
| *MXI1* | 3,42E-27 | 4,47E-26 | 4,9 |  | 2,8 | 3,16E-31 | 5,96E-30 |
| *EPHA4* | 3,73E-27 | 4,86E-26 | -9,8 |  | 2,1 | 2,21E-05 | 6,43E-05 |
| *DHCR7* | 4,63E-27 | 6,03E-26 | -5,5 |  | -4,4 | 7,35E-12 | 4,34E-11 |
| *IDH2* | 4,70E-27 | 6,12E-26 | -3,6 |  | -3,1 | 1,83E-05 | 5,37E-05 |
| *TMTC2* | 5,07E-27 | 6,59E-26 | 44,1 |  | 2,1 | 2,65E-05 | 7,62E-05 |
| *DGKA* | 5,27E-27 | 6,84E-26 | -4,1 |  | -3,5 | 5,50E-10 | 2,74E-09 |
| *STX12* | 5,45E-27 | 7,06E-26 | 3,1 |  | 2,1 | 2,78E-27 | 4,38E-26 |
| *CTNNBL1* | 5,47E-27 | 7,08E-26 | 4,1 |  | 11,3 | 4,04E-143 | 7,75E-141 |
| *SPHK1* | 6,19E-27 | 8,01E-26 | 2,9 |  | -2,3 | 1,34E-01 | 1,85E-01 |
| *ADPGK* | 6,71E-27 | 8,67E-26 | 2,6 |  | 4,1 | 9,57E-23 | 1,18E-21 |
| *EIF2AK2* | 9,21E-27 | 1,19E-25 | 4,1 |  | 1,4 | 1,03E-07 | 4,05E-07 |
| *OGFRL1* | 9,63E-27 | 1,24E-25 | 3,4 |  | -2,3 | 3,76E-01 | 4,48E-01 |
| *DYRK4* | 1,05E-26 | 1,35E-25 | 6,2 |  | 4,5 | 1,79E-27 | 2,84E-26 |
| *SH3BP2* | 1,19E-26 | 1,53E-25 | 2,9 |  | 2,3 | 1,64E-17 | 1,48E-16 |
| *SPEG* | 1,27E-26 | 1,62E-25 | -7,0 |  | -4,8 | 9,05E-21 | 1,00E-19 |
| *ABCD3* | 1,32E-26 | 1,69E-25 | -2,6 |  | -3,5 | 2,02E-12 | 1,25E-11 |
| *SLC23A2* | 1,53E-26 | 1,95E-25 | -4,9 |  | -14,8 | 3,13E-22 | 3,73E-21 |
| *CLIC2* | 1,53E-26 | 1,95E-25 | 4100,7 |  | 21,5 | 9,81E-18 | 8,99E-17 |
| *UBE2E3* | 1,64E-26 | 2,09E-25 | -4,1 |  | -4,4 | 6,96E-15 | 5,11E-14 |
| *TYRO3* | 1,79E-26 | 2,28E-25 | -5,2 |  | -6,9 | 4,06E-16 | 3,27E-15 |
| *PPP1R18* | 1,87E-26 | 2,37E-25 | 2,6 |  | 1,7 | 1,78E-11 | 1,02E-10 |
| *SOCS5* | 2,10E-26 | 2,67E-25 | -3,1 |  | -4,3 | 1,74E-24 | 2,33E-23 |
| *PNPLA8* | 2,30E-26 | 2,91E-25 | 3,4 |  | 1,1 | 2,27E-06 | 7,52E-06 |
| *RTP4* | 2,77E-26 | 3,50E-25 | 15431,4 |  | 278,2 | 2,04E-12 | 1,26E-11 |
| *GAMT* | 3,03E-26 | 3,82E-25 | -9,8 |  | -6,4 | 5,26E-12 | 3,15E-11 |
| *CDV3* | 3,26E-26 | 4,11E-25 | 2,2 |  | 0,2 | 8,78E-02 | 1,27E-01 |
| *BTC* | 3,36E-26 | 4,23E-25 | 214,8 |  | 373,7 | 7,13E-20 | 7,46E-19 |
| *ALS2* | 3,42E-26 | 4,31E-25 | -3,5 |  | -3,2 | 1,47E-10 | 7,70E-10 |
| *YEATS2* | 3,51E-26 | 4,41E-25 | 2,6 |  | 4,4 | 3,96E-56 | 1,65E-54 |
| *PARD6G* | 3,77E-26 | 4,74E-25 | -8,8 |  | -103,8 | 2,90E-11 | 1,62E-10 |
| *CPNE3* | 3,78E-26 | 4,75E-25 | -2,0 |  | -2,9 | 7,87E-09 | 3,47E-08 |
| *BZW2* | 4,03E-26 | 5,06E-25 | -2,8 |  | -6,7 | 1,15E-20 | 1,26E-19 |
| *MLPH* | 4,29E-26 | 5,37E-25 | -11,9 |  | -7,1 | 4,12E-14 | 2,87E-13 |
| *CHI3L2* | 4,64E-26 | 5,81E-25 | 26,1 |  | 0,6 | 2,11E-02 | 3,59E-02 |
| *VPS26C* | 5,30E-26 | 6,62E-25 | 2,7 |  | 1,1 | 1,43E-20 | 1,57E-19 |
| *GPAM* | 5,37E-26 | 6,71E-25 | -5,3 |  | -14,2 | 5,17E-41 | 1,44E-39 |
| *CALCOCO1* | 5,46E-26 | 6,81E-25 | 3,3 |  | 2,5 | 1,61E-10 | 8,44E-10 |
| *PIEZO1* | 6,66E-26 | 8,30E-25 | 3,4 |  | 4,3 | 1,44E-14 | 1,03E-13 |
| *BICC1* | 7,20E-26 | 8,96E-25 | -2,8 |  | -4,4 | 4,54E-37 | 1,10E-35 |
| *SEMA4D* | 8,01E-26 | 9,96E-25 | 11836,5 |  | 6094,5 | 2,45E-21 | 2,79E-20 |
| *AGPAT3* | 9,05E-26 | 1,12E-24 | 3,2 |  | 2,1 | 2,91E-18 | 2,77E-17 |
| *MYL12A* | 9,91E-26 | 1,23E-24 | 2,8 |  | 2,2 | 1,29E-18 | 1,26E-17 |
| *DFFA* | 1,06E-25 | 1,31E-24 | -2,9 |  | -3,9 | 2,96E-19 | 3,03E-18 |
| *TMEM97* | 1,11E-25 | 1,37E-24 | -9,7 |  | -33,3 | 1,23E-13 | 8,29E-13 |
| *SPSB1* | 1,14E-25 | 1,41E-24 | 5,8 |  | 2,1 | 2,13E-09 | 1,00E-08 |
| *LONRF1* | 1,51E-25 | 1,86E-24 | 6,7 |  | 1,1 | 5,15E-04 | 1,19E-03 |
| *ARHGEF40* | 1,55E-25 | 1,91E-24 | 2,6 |  | 0,6 | 2,35E-03 | 4,85E-03 |
| *EBPL* | 1,58E-25 | 1,95E-24 | -5,2 |  | -6,8 | 7,56E-16 | 5,97E-15 |
| *LTBP2* | 1,64E-25 | 2,02E-24 | -5,3 |  | -5,3 | 1,63E-22 | 1,98E-21 |
| *GXYLT2* | 1,69E-25 | 2,08E-24 | -8,7 |  | -33,6 | 1,18E-10 | 6,24E-10 |
| *TMBIM1* | 1,70E-25 | 2,09E-24 | 2,6 |  | 3,0 | 1,72E-31 | 3,26E-30 |
| *SMAGP* | 1,77E-25 | 2,17E-24 | -3,6 |  | -2,9 | 4,10E-05 | 1,15E-04 |
| *PDE4DIPP2* | 1,79E-25 | 2,20E-24 | 6,3 |  | 1,4 | 2,87E-04 | 6,93E-04 |
| *SYDE1* | 1,79E-25 | 2,20E-24 | 2,4 |  | 1,8 | 7,23E-11 | 3,88E-10 |
| *TENM4* | 1,88E-25 | 2,30E-24 | -9,9 |  | -36,3 | 5,12E-111 | 6,22E-109 |
| *IVNS1ABP* | 2,32E-25 | 2,84E-24 | 2,8 |  | 1,9 | 2,19E-20 | 2,37E-19 |
| *EIF3F* | 2,54E-25 | 3,10E-24 | -2,5 |  | -4,0 | 1,85E-22 | 2,23E-21 |
| *SNX6* | 2,69E-25 | 3,28E-24 | 2,3 |  | 2,6 | 3,52E-23 | 4,42E-22 |
| *RNF24* | 2,83E-25 | 3,44E-24 | 6,4 |  | 1,5 | 2,35E-20 | 2,54E-19 |
| *HLA-L* | 2,95E-25 | 3,58E-24 | 31,1 |  | 22,5 | 7,18E-36 | 1,67E-34 |
| *ITGA10* | 3,22E-25 | 3,91E-24 | -14,5 |  | -89,9 | 7,48E-44 | 2,29E-42 |
| *TMEM120B* | 3,62E-25 | 4,39E-24 | -5,2 |  | -5,3 | 2,58E-07 | 9,66E-07 |
| *HLA-DQA2* | 3,76E-25 | 4,56E-24 | 11418,9 |  | 57,8 | 8,70E-67 | 4,63E-65 |
| *SLC30A7* | 3,86E-25 | 4,67E-24 | 3,1 |  | 1,0 | 2,59E-06 | 8,51E-06 |
| *CLDN1* | 4,34E-25 | 5,25E-24 | 6,7 |  | 3149,8 | 9,77E-17 | 8,27E-16 |
| *MYBL1* | 4,37E-25 | 5,27E-24 | -5,3 |  | 0,8 | 7,78E-02 | 1,14E-01 |
| *LMNA* | 4,48E-25 | 5,40E-24 | -2,7 |  | -3,2 | 1,07E-06 | 3,72E-06 |
| *FHL2* | 5,50E-25 | 6,63E-24 | -2,7 |  | -4,2 | 1,00E-27 | 1,60E-26 |
| *SNHG14* | 5,74E-25 | 6,91E-24 | -3,4 |  | -6,7 | 8,39E-18 | 7,72E-17 |
| *CMTR1* | 5,76E-25 | 6,92E-24 | 2,8 |  | 6,1 | 2,92E-69 | 1,64E-67 |
| *SNTB1* | 5,97E-25 | 7,16E-24 | -7,4 |  | -5,7 | 9,47E-08 | 3,74E-07 |
| *CDYL2* | 6,09E-25 | 7,31E-24 | 3,9 |  | 3,0 | 1,79E-16 | 1,49E-15 |
| *PCYT2* | 7,07E-25 | 8,48E-24 | -3,0 |  | -3,4 | 6,07E-12 | 3,61E-11 |
| *RCN2* | 7,39E-25 | 8,84E-24 | -2,8 |  | -3,6 | 1,93E-11 | 1,10E-10 |
| *CPPED1* | 8,61E-25 | 1,03E-23 | 2,9 |  | 2,8 | 2,48E-22 | 2,96E-21 |
| *TESK2* | 9,67E-25 | 1,15E-23 | 44,0 |  | 18,1 | 1,47E-04 | 3,72E-04 |
| *ATXN7* | 1,00E-24 | 1,19E-23 | 3,4 |  | 2,7 | 2,34E-26 | 3,50E-25 |
| *SNHG16* | 1,16E-24 | 1,38E-23 | -2,3 |  | -4,0 | 2,84E-10 | 1,45E-09 |
| *MORC4* | 1,24E-24 | 1,47E-23 | -3,5 |  | -5,9 | 3,29E-21 | 3,72E-20 |
| *DDR2* | 1,33E-24 | 1,58E-23 | -2,4 |  | -7,8 | 1,47E-48 | 5,22E-47 |
| *SH3D21* | 1,50E-24 | 1,78E-23 | 10,4 |  | 12,8 | 1,85E-24 | 2,47E-23 |
| *MAP3K5* | 1,56E-24 | 1,85E-23 | 4,2 |  | 0,9 | 3,33E-05 | 9,41E-05 |
| *ALAS1* | 1,81E-24 | 2,14E-23 | 3,5 |  | 1,8 | 1,25E-24 | 1,69E-23 |
| *PEBP1* | 1,86E-24 | 2,19E-23 | -2,3 |  | -3,3 | 1,71E-22 | 2,07E-21 |
| *PRSS12* | 1,97E-24 | 2,33E-23 | -7,9 |  | -21,1 | 2,35E-32 | 4,67E-31 |
| *ADCY4* | 2,32E-24 | 2,74E-23 | 159,1 |  | 6,6 | 1,76E-08 | 7,51E-08 |
| *PFKM* | 2,39E-24 | 2,82E-23 | -2,8 |  | -4,4 | 4,77E-20 | 5,04E-19 |
| *LYPD5* | 2,46E-24 | 2,90E-23 | 11612,3 |  | 71,1 | 1,84E-23 | 2,34E-22 |
| *GALC* | 2,61E-24 | 3,07E-23 | 3,1 |  | -2,8 | 4,14E-07 | 1,51E-06 |
| *PDCD10* | 2,61E-24 | 3,07E-23 | 2,8 |  | 2,0 | 8,30E-21 | 9,21E-20 |
| *RAB27B* | 2,70E-24 | 3,17E-23 | -3,5 |  | 2,5 | 3,00E-12 | 1,83E-11 |
| *FUT10* | 3,14E-24 | 3,68E-23 | -3,8 |  | -4,4 | 3,50E-08 | 1,45E-07 |
| *CYBRD1* | 4,16E-24 | 4,87E-23 | -2,6 |  | -6,7 | 2,17E-39 | 5,69E-38 |
| *DENND4A* | 4,88E-24 | 5,71E-23 | 3,2 |  | 0,2 | 4,54E-01 | 5,26E-01 |
| *MN1* | 4,88E-24 | 5,71E-23 | -43,0 |  | 8,8 | 3,20E-07 | 1,19E-06 |
| *PLEKHO1* | 4,96E-24 | 5,80E-23 | 3,3 |  | 0,8 | 4,67E-06 | 1,49E-05 |
| *GAP43* | 5,05E-24 | 5,89E-23 | -38,3 |  | -538,7 | 2,31E-16 | 1,90E-15 |
| *CCDC170* | 5,45E-24 | 6,35E-23 | 13,9 |  | 1,4 | 4,07E-03 | 8,00E-03 |
| *RAB8B* | 6,16E-24 | 7,17E-23 | 3,2 |  | 3,6 | 2,18E-21 | 2,49E-20 |
| *GRHPR* | 6,54E-24 | 7,62E-23 | -2,7 |  | -3,1 | 1,13E-08 | 4,92E-08 |
| *HYAL3* | 6,72E-24 | 7,81E-23 | 7,9 |  | 9,0 | 7,04E-16 | 5,58E-15 |
| *IDH1* | 7,20E-24 | 8,36E-23 | -2,7 |  | -3,6 | 8,85E-13 | 5,61E-12 |
| *DYNLT1* | 7,72E-24 | 8,95E-23 | 2,7 |  | 4,9 | 2,38E-18 | 2,29E-17 |
| *ITGB5* | 7,94E-24 | 9,20E-23 | -3,5 |  | -5,3 | 1,02E-18 | 1,01E-17 |
| *ILK* | 8,06E-24 | 9,33E-23 | 2,6 |  | 1,7 | 1,77E-13 | 1,18E-12 |
| *OSBPL3* | 8,51E-24 | 9,85E-23 | -2,4 |  | -4,6 | 3,61E-06 | 1,17E-05 |
| *GSTA4* | 8,62E-24 | 9,96E-23 | -12,2 |  | -22,3 | 1,23E-24 | 1,66E-23 |
| *CLN8* | 9,03E-24 | 1,04E-22 | -4,2 |  | -3,4 | 4,76E-05 | 1,31E-04 |
| *KCTD3* | 9,08E-24 | 1,05E-22 | 3,2 |  | -2,0 | 8,27E-01 | 8,61E-01 |
| *YPEL2* | 9,24E-24 | 1,07E-22 | 4,7 |  | 1,4 | 4,38E-05 | 1,21E-04 |
| *CARD16* | 1,04E-23 | 1,20E-22 | 2609,2 |  | 17,3 | 1,07E-10 | 5,69E-10 |
| *TNFAIP1* | 1,05E-23 | 1,20E-22 | 2,9 |  | 1,3 | 3,44E-17 | 3,01E-16 |
| *NAAA* | 1,25E-23 | 1,43E-22 | 4,4 |  | 0,4 | 2,65E-02 | 4,39E-02 |
| *EPB41* | 1,49E-23 | 1,72E-22 | 4,4 |  | 1,5 | 2,89E-05 | 8,27E-05 |
| *TRIP10* | 1,58E-23 | 1,81E-22 | 3,9 |  | 1,2 | 5,06E-08 | 2,06E-07 |
| *SH3RF2* | 1,75E-23 | 2,00E-22 | -12,2 |  | -7,0 | 2,22E-05 | 6,45E-05 |
| *HGF* | 1,75E-23 | 2,00E-22 | 114,1 |  | -2,6 | 7,08E-06 | 2,21E-05 |
| *GPAT3* | 1,75E-23 | 2,00E-22 | -7,0 |  | -6,2 | 1,14E-08 | 4,96E-08 |
| *NUMB* | 2,05E-23 | 2,34E-22 | 3,3 |  | 1,9 | 1,07E-30 | 1,95E-29 |
| *SEPHS1* | 2,37E-23 | 2,70E-22 | -3,3 |  | -4,6 | 3,58E-31 | 6,72E-30 |
| *MIR155HG* | 2,57E-23 | 2,93E-22 | 12,1 |  | 68,7 | 8,95E-104 | 1,01E-101 |
| *AHR* | 2,59E-23 | 2,94E-22 | 4,4 |  | 0,8 | 3,22E-05 | 9,12E-05 |
| *YWHAQ* | 2,66E-23 | 3,03E-22 | -2,1 |  | -2,3 | 1,79E-01 | 2,37E-01 |
| *MYH10* | 2,73E-23 | 3,10E-22 | -4,3 |  | -14,5 | 4,25E-56 | 1,77E-54 |
| *NECAP2* | 2,85E-23 | 3,24E-22 | 2,4 |  | 4,2 | 3,80E-31 | 7,11E-30 |
| *MCTP1* | 2,87E-23 | 3,26E-22 | 3,4 |  | 2,0 | 3,48E-05 | 9,82E-05 |
| *BCL6* | 2,88E-23 | 3,27E-22 | 4,3 |  | 2,7 | 1,79E-15 | 1,37E-14 |
| *AOX1* | 2,95E-23 | 3,34E-22 | -2,9 |  | -2,0 | 8,68E-01 | 8,95E-01 |
| *RELL1* | 3,30E-23 | 3,73E-22 | 5,3 |  | -2,3 | 1,71E-01 | 2,29E-01 |
| *SUSD1* | 3,31E-23 | 3,74E-22 | 3,3 |  | 2,9 | 5,00E-25 | 6,97E-24 |
| *CCBE1* | 3,36E-23 | 3,79E-22 | 2,9 |  | 3,2 | 2,48E-24 | 3,29E-23 |
| *RETREG3* | 3,44E-23 | 3,88E-22 | 2,8 |  | 2,2 | 9,71E-25 | 1,33E-23 |
| *CNTNAP1* | 3,72E-23 | 4,19E-22 | 4,8 |  | 1,3 | 3,22E-05 | 9,12E-05 |
| *PRKCE* | 3,74E-23 | 4,21E-22 | -15,8 |  | -2,1 | 5,86E-01 | 6,50E-01 |
| *GDF11* | 3,89E-23 | 4,37E-22 | -4,6 |  | -9,0 | 4,40E-38 | 1,10E-36 |
| *N4BP1* | 4,23E-23 | 4,75E-22 | 2,8 |  | 6,5 | 1,50E-113 | 1,90E-111 |
| *COPZ2* | 4,29E-23 | 4,81E-22 | -4,8 |  | -4,6 | 1,62E-14 | 1,16E-13 |
| *HLA-DQB1* | 4,39E-23 | 4,92E-22 | 8641,7 |  | 283,0 | 2,84E-20 | 3,05E-19 |
| *TYMP* | 4,44E-23 | 4,97E-22 | 2441,2 |  | 878,1 | 1,81E-260 | 9,65E-258 |
| *ENY2* | 4,52E-23 | 5,06E-22 | 2,9 |  | 1,4 | 4,80E-09 | 2,18E-08 |
| *SMIM3* | 4,95E-23 | 5,53E-22 | 3,4 |  | 0,9 | 1,18E-02 | 2,11E-02 |
| *AMOTL1* | 5,03E-23 | 5,62E-22 | -3,2 |  | -3,0 | 2,31E-09 | 1,08E-08 |
| *A4GALT* | 5,47E-23 | 6,11E-22 | 17,5 |  | 7,6 | 2,13E-30 | 3,80E-29 |
| *DENND3* | 5,70E-23 | 6,35E-22 | 7,0 |  | 10,7 | 1,70E-35 | 3,91E-34 |
| *BAK1* | 5,77E-23 | 6,43E-22 | 4,0 |  | 5,2 | 1,90E-45 | 6,20E-44 |
| *RPS3A* | 6,04E-23 | 6,72E-22 | -3,4 |  | -8,6 | 3,93E-55 | 1,60E-53 |
| *GCAT* | 6,63E-23 | 7,37E-22 | -6,2 |  | -19,0 | 7,14E-26 | 1,04E-24 |
| *EPOP* | 7,78E-23 | 8,65E-22 | 299,7 |  | 14,7 | 1,43E-14 | 1,03E-13 |
| *TRIP6* | 7,87E-23 | 8,74E-22 | -3,0 |  | -2,2 | 1,97E-01 | 2,58E-01 |
| *H2AZ2* | 9,29E-23 | 1,03E-21 | -2,7 |  | -5,8 | 3,44E-67 | 1,86E-65 |
| *IL12RB1* | 9,65E-23 | 1,07E-21 | 9273,3 |  | 21863,2 | 9,63E-31 | 1,76E-29 |
| *SMARCA5* | 9,66E-23 | 1,07E-21 | 2,3 |  | 1,1 | 2,09E-06 | 6,95E-06 |
| *S100A3* | 1,04E-22 | 1,14E-21 | 7,9 |  | 2,1 | 2,93E-04 | 7,07E-04 |
| *BEX3* | 1,09E-22 | 1,20E-21 | -3,0 |  | -2,8 | 6,69E-04 | 1,51E-03 |
| *GSDME* | 1,17E-22 | 1,29E-21 | 2,9 |  | 1,3 | 6,04E-16 | 4,82E-15 |
| *PTGIR* | 1,18E-22 | 1,30E-21 | 36,9 |  | 60,1 | 4,65E-34 | 1,00E-32 |
| *EHD4* | 1,23E-22 | 1,35E-21 | 2,8 |  | 5,5 | 2,42E-18 | 2,32E-17 |
| *DNAI3* | 1,51E-22 | 1,65E-21 | -7208,2 |  | -24,9 | 1,07E-21 | 1,24E-20 |
| *RCN3* | 1,52E-22 | 1,66E-21 | 4,0 |  | 0,5 | 1,03E-03 | 2,25E-03 |
| *AGTRAP* | 1,55E-22 | 1,70E-21 | 3,5 |  | 5,8 | 2,48E-16 | 2,04E-15 |
| *HERPUD2* | 1,56E-22 | 1,71E-21 | 2,8 |  | 1,5 | 1,34E-12 | 8,34E-12 |
| *NBEA* | 1,62E-22 | 1,77E-21 | -9,0 |  | -3,4 | 1,94E-02 | 3,32E-02 |
| *EOGT* | 1,64E-22 | 1,79E-21 | 3,1 |  | -2,1 | 7,71E-01 | 8,13E-01 |
| *TLK2* | 1,66E-22 | 1,81E-21 | 2,8 |  | 1,2 | 4,21E-09 | 1,92E-08 |
| *ATG7* | 1,77E-22 | 1,92E-21 | 2,5 |  | 7,2 | 3,93E-51 | 1,47E-49 |
| *ENSG00000232618* | 1,83E-22 | 2,00E-21 | 8624,7 |  | 110,5 | 6,55E-06 | 2,05E-05 |
| *EHBP1L1* | 1,92E-22 | 2,09E-21 | 4,4 |  | 0,4 | 2,10E-03 | 4,39E-03 |
| *TUFT1* | 2,01E-22 | 2,19E-21 | -4,1 |  | 0,0 | 9,53E-01 | 9,63E-01 |
| *TLN2* | 2,07E-22 | 2,25E-21 | -3,6 |  | -8,8 | 2,72E-25 | 3,87E-24 |
| *RB1* | 2,07E-22 | 2,25E-21 | 2,7 |  | 1,5 | 2,40E-13 | 1,59E-12 |
| *FNDC3B* | 2,35E-22 | 2,54E-21 | 3,3 |  | 0,9 | 6,28E-05 | 1,69E-04 |
| *BCAR3* | 2,42E-22 | 2,62E-21 | 2,2 |  | 2,6 | 5,24E-19 | 5,25E-18 |
| *FAS* | 2,50E-22 | 2,70E-21 | 5,4 |  | 7,0 | 9,85E-58 | 4,21E-56 |
| *GBP6* | 3,01E-22 | 3,25E-21 | 8385,8 |  | 8426,6 | 1,45E-22 | 1,76E-21 |
| *MECOM* | 3,04E-22 | 3,28E-21 | -6,0 |  | -2,4 | 1,78E-01 | 2,36E-01 |
| *PTPN13* | 3,08E-22 | 3,33E-21 | -2,7 |  | -14,2 | 8,97E-70 | 5,13E-68 |
| *GPR155* | 3,44E-22 | 3,70E-21 | -5,5 |  | -3,2 | 1,27E-03 | 2,74E-03 |
| *PSMA5* | 3,50E-22 | 3,76E-21 | 2,7 |  | 2,2 | 2,71E-28 | 4,44E-27 |
| *ODF3B* | 3,70E-22 | 3,98E-21 | 7000,3 |  | 144,0 | 1,37E-36 | 3,26E-35 |
| *NOTCH2* | 3,82E-22 | 4,11E-21 | 3,1 |  | 3,9 | 9,79E-62 | 4,62E-60 |
| *STX11* | 3,82E-22 | 4,11E-21 | 870,1 |  | 581,6 | 1,87E-61 | 8,75E-60 |
| *ADGRA2* | 3,84E-22 | 4,12E-21 | -6,2 |  | -6,3 | 2,35E-17 | 2,08E-16 |
| *CGAS* | 3,91E-22 | 4,19E-21 | 30,8 |  | 8,0 | 7,18E-53 | 2,78E-51 |
| *C6orf62* | 4,01E-22 | 4,29E-21 | 2,8 |  | 1,7 | 3,05E-16 | 2,49E-15 |
| *GATD1* | 4,25E-22 | 4,55E-21 | -2,5 |  | -3,1 | 2,09E-08 | 8,86E-08 |
| *EBF3* | 4,64E-22 | 4,96E-21 | -7,6 |  | -6,8 | 1,07E-18 | 1,05E-17 |
| *WDR11* | 4,73E-22 | 5,05E-21 | 2,4 |  | 0,5 | 4,48E-04 | 1,05E-03 |
| *MYLK* | 4,96E-22 | 5,29E-21 | -2,1 |  | -2,1 | 2,76E-01 | 3,45E-01 |
| *TAGLN2* | 5,00E-22 | 5,33E-21 | 2,5 |  | 1,9 | 3,01E-09 | 1,39E-08 |
| *CCSER2* | 5,08E-22 | 5,41E-21 | 3,3 |  | 1,4 | 5,42E-09 | 2,44E-08 |
| *NR3C1* | 5,08E-22 | 5,41E-21 | 2,6 |  | 0,7 | 3,19E-05 | 9,06E-05 |
| *NEXN* | 5,17E-22 | 5,49E-21 | -5,8 |  | -3,5 | 7,27E-05 | 1,94E-04 |
| *CPEB4* | 5,52E-22 | 5,87E-21 | 5,0 |  | 0,8 | 1,98E-03 | 4,14E-03 |
| *DUSP1* | 5,57E-22 | 5,91E-21 | 3,2 |  | 3,3 | 6,34E-08 | 2,55E-07 |
| *POLD2* | 5,58E-22 | 5,91E-21 | -3,1 |  | -3,0 | 2,24E-03 | 4,63E-03 |
| *RPL7* | 5,72E-22 | 6,06E-21 | -2,9 |  | -9,5 | 3,39E-94 | 3,11E-92 |
| *KDSR* | 6,24E-22 | 6,61E-21 | 2,8 |  | 0,6 | 3,34E-04 | 7,98E-04 |
| *FADS2* | 6,32E-22 | 6,68E-21 | -2,4 |  | -2,4 | 5,99E-02 | 9,08E-02 |
| *ITM2B* | 6,85E-22 | 7,24E-21 | 2,5 |  | 4,8 | 1,83E-64 | 9,27E-63 |
| *IL6ST* | 6,89E-22 | 7,28E-21 | 3,1 |  | 0,1 | 4,93E-01 | 5,64E-01 |
| *CBX6* | 6,92E-22 | 7,30E-21 | -3,0 |  | -4,1 | 4,37E-20 | 4,65E-19 |
| *DENND1A* | 6,95E-22 | 7,32E-21 | 4,1 |  | 11,9 | 4,25E-100 | 4,46E-98 |
| *MAP2K6* | 7,33E-22 | 7,72E-21 | -124,4 |  | -570,5 | 4,66E-14 | 3,23E-13 |
| *FAM210B* | 7,84E-22 | 8,24E-21 | -2,6 |  | -3,5 | 1,86E-06 | 6,23E-06 |
| *SCAI* | 7,87E-22 | 8,27E-21 | -6,3 |  | -8,3 | 7,29E-31 | 1,34E-29 |
| *FDFT1* | 7,98E-22 | 8,37E-21 | -2,7 |  | -2,3 | 3,09E-02 | 5,02E-02 |
| *SPATA18* | 8,07E-22 | 8,46E-21 | -4,0 |  | -9,0 | 3,90E-24 | 5,12E-23 |
| *NUDT3* | 8,17E-22 | 8,56E-21 | -4,6 |  | -12,5 | 3,05E-29 | 5,21E-28 |
| *CLDN12* | 8,25E-22 | 8,63E-21 | 3,1 |  | 1,9 | 8,30E-19 | 8,21E-18 |
| *TRAF2* | 8,52E-22 | 8,90E-21 | 5,0 |  | 9,2 | 4,56E-33 | 9,35E-32 |
| *NUCKS1* | 8,68E-22 | 9,06E-21 | -2,3 |  | -3,2 | 1,06E-07 | 4,16E-07 |
| *SLC15A4* | 8,87E-22 | 9,25E-21 | 3,6 |  | 3,5 | 1,36E-24 | 1,83E-23 |
| *SNAI2* | 9,25E-22 | 9,64E-21 | -2,5 |  | -23,8 | 3,33E-80 | 2,40E-78 |
| *PRR16* | 1,00E-21 | 1,05E-20 | -9,7 |  | 0,7 | 1,17E-01 | 1,64E-01 |
| *TNFRSF10A* | 1,10E-21 | 1,14E-20 | 4,7 |  | 4,2 | 6,04E-25 | 8,37E-24 |
| *ADAMTS9* | 1,22E-21 | 1,26E-20 | 258,8 |  | 1,3 | 5,65E-03 | 1,08E-02 |
| *LHFPL6* | 1,26E-21 | 1,31E-20 | 4,4 |  | 2,7 | 5,21E-34 | 1,12E-32 |
| *ANKLE2* | 1,27E-21 | 1,32E-20 | 2,5 |  | 0,6 | 1,06E-07 | 4,14E-07 |
| *MACROH2A2* | 1,31E-21 | 1,36E-20 | 6,7 |  | 10,0 | 1,06E-73 | 6,71E-72 |
| *BRIP1* | 1,32E-21 | 1,37E-20 | 3,6 |  | 21,5 | 5,62E-70 | 3,26E-68 |
| *PAPSS2* | 1,43E-21 | 1,48E-20 | 2,8 |  | -4,5 | 2,22E-03 | 4,60E-03 |
| *CREG1* | 1,43E-21 | 1,48E-20 | 5,1 |  | 2,8 | 2,40E-19 | 2,47E-18 |
| *PLCD3* | 1,52E-21 | 1,57E-20 | -5,2 |  | -6,9 | 2,28E-19 | 2,35E-18 |
| *MEF2C* | 1,54E-21 | 1,59E-20 | -5,0 |  | -2,8 | 2,54E-02 | 4,23E-02 |
| *EPB41L4A* | 1,60E-21 | 1,65E-20 | 6194,1 |  | 642,1 | 2,09E-16 | 1,72E-15 |
| *PLEK2* | 1,60E-21 | 1,65E-20 | 8,1 |  | 63,4 | 2,73E-08 | 1,14E-07 |
| *B4GALT2* | 1,70E-21 | 1,74E-20 | -2,6 |  | -3,0 | 5,90E-04 | 1,35E-03 |
| *FSTL1* | 1,71E-21 | 1,75E-20 | 3,1 |  | 0,5 | 8,28E-05 | 2,18E-04 |
| *FAM168A* | 1,79E-21 | 1,83E-20 | -3,4 |  | -3,8 | 9,68E-21 | 1,07E-19 |
| *LUZP1* | 2,03E-21 | 2,08E-20 | 2,8 |  | 0,7 | 8,95E-10 | 4,38E-09 |
| *SLC39A10* | 2,06E-21 | 2,11E-20 | -3,4 |  | -15,3 | 8,78E-42 | 2,53E-40 |
| *ENSG00000286190* | 2,24E-21 | 2,30E-20 | -26,4 |  | -42,4 | 1,26E-08 | 5,45E-08 |
| *HIVEP1* | 2,56E-21 | 2,62E-20 | 3,9 |  | 1,0 | 9,15E-07 | 3,20E-06 |
| *RAB40B* | 2,67E-21 | 2,73E-20 | -12,8 |  | -24,7 | 1,50E-11 | 8,67E-11 |
| *GSN* | 2,73E-21 | 2,79E-20 | -2,4 |  | -4,3 | 4,08E-23 | 5,11E-22 |
| *FOSL2* | 2,79E-21 | 2,84E-20 | 2,7 |  | 1,9 | 4,29E-10 | 2,16E-09 |
| *ATOH8* | 2,96E-21 | 3,02E-20 | 6,0 |  | 7,4 | 2,74E-24 | 3,62E-23 |
| *SCP2* | 3,05E-21 | 3,11E-20 | -2,7 |  | -3,5 | 1,39E-23 | 1,77E-22 |
| *CARMN* | 3,05E-21 | 3,11E-20 | -18,5 |  | -62,8 | 5,89E-27 | 9,12E-26 |
| *DDX58* | 3,13E-21 | 3,18E-20 | 31,8 |  | 17,8 | 1,04E-12 | 6,51E-12 |
| *GNG12* | 3,14E-21 | 3,18E-20 | -2,3 |  | -3,6 | 2,94E-06 | 9,59E-06 |
| *DANCR* | 3,16E-21 | 3,21E-20 | -10,4 |  | -18,6 | 7,91E-17 | 6,73E-16 |
| *ARMCX2* | 3,89E-21 | 3,94E-20 | -2,3 |  | -3,1 | 6,80E-10 | 3,36E-09 |
| *LIMCH1* | 3,97E-21 | 4,01E-20 | 16,0 |  | 3,2 | 2,58E-09 | 1,20E-08 |
| *KLF12* | 4,21E-21 | 4,25E-20 | -4,3 |  | -4,8 | 1,27E-17 | 1,16E-16 |
| *PTK7* | 4,23E-21 | 4,28E-20 | -2,5 |  | -4,0 | 3,88E-14 | 2,71E-13 |
| *TUBB6* | 4,25E-21 | 4,29E-20 | 2,2 |  | 1,6 | 9,58E-07 | 3,34E-06 |
| *RNF19A* | 4,53E-21 | 4,57E-20 | 2,9 |  | 2,1 | 2,33E-16 | 1,92E-15 |
| *HACD2* | 5,05E-21 | 5,09E-20 | -3,6 |  | -3,4 | 4,72E-08 | 1,93E-07 |
| *IARS1* | 5,08E-21 | 5,11E-20 | -2,2 |  | -3,5 | 8,06E-09 | 3,55E-08 |
| *LAMB3* | 5,23E-21 | 5,26E-20 | 19,3 |  | 0,4 | 5,92E-02 | 8,99E-02 |
| *ADORA2B* | 5,27E-21 | 5,30E-20 | -8,4 |  | 1,4 | 4,51E-02 | 7,03E-02 |
| *RPL13A* | 5,42E-21 | 5,44E-20 | -3,1 |  | -6,0 | 1,90E-46 | 6,42E-45 |
| *CHP1* | 5,44E-21 | 5,46E-20 | -2,2 |  | -4,3 | 4,59E-21 | 5,16E-20 |
| *RILP* | 5,49E-21 | 5,50E-20 | 7,5 |  | 2,2 | 1,08E-04 | 2,80E-04 |
| *AHCY* | 5,75E-21 | 5,76E-20 | -2,6 |  | -4,1 | 4,48E-16 | 3,60E-15 |
| *BCL10* | 5,93E-21 | 5,93E-20 | 2,6 |  | 2,6 | 5,61E-26 | 8,21E-25 |
| *ATF5* | 6,14E-21 | 6,14E-20 | 3,4 |  | 36,3 | 3,29E-136 | 5,69E-134 |
| *MAPKAPK3* | 6,27E-21 | 6,26E-20 | -4,4 |  | -2,7 | 2,19E-03 | 4,55E-03 |
| *TRIM2* | 6,71E-21 | 6,70E-20 | -5,3 |  | -19,9 | 2,22E-44 | 6,90E-43 |
| *PIK3IP1* | 6,75E-21 | 6,73E-20 | 4,7 |  | 0,4 | 1,63E-01 | 2,19E-01 |
| *FAP* | 6,77E-21 | 6,75E-20 | 2,4 |  | -9,0 | 6,51E-15 | 4,79E-14 |
| *GPRASP2* | 6,96E-21 | 6,93E-20 | -3,9 |  | -4,6 | 4,56E-11 | 2,50E-10 |
| *HIP1R* | 6,97E-21 | 6,94E-20 | -4,7 |  | -3,3 | 1,02E-02 | 1,85E-02 |
| *CPE* | 7,59E-21 | 7,54E-20 | -4,2 |  | -8,8 | 1,98E-05 | 5,78E-05 |
| *LINC02015* | 7,68E-21 | 7,62E-20 | 168,3 |  | 216,3 | 3,14E-91 | 2,62E-89 |
| *TEC* | 7,77E-21 | 7,71E-20 | 37,0 |  | 10,6 | 2,34E-08 | 9,88E-08 |
| *MEGF6* | 7,95E-21 | 7,88E-20 | 552,1 |  | 0,3 | 2,00E-01 | 2,61E-01 |
| *ATXN7L3B* | 8,06E-21 | 7,98E-20 | -2,2 |  | -2,6 | 1,39E-04 | 3,53E-04 |
| *UPP1* | 8,31E-21 | 8,23E-20 | 5,7 |  | 2,8 | 2,30E-11 | 1,30E-10 |
| *IBTK* | 9,44E-21 | 9,34E-20 | 2,5 |  | -2,6 | 7,17E-03 | 1,34E-02 |
| *BEND5* | 9,49E-21 | 9,38E-20 | 119,8 |  | 73,8 | 5,29E-25 | 7,36E-24 |
| *ERN1* | 9,97E-21 | 9,84E-20 | 6,9 |  | 2,8 | 9,15E-14 | 6,22E-13 |
| *PCK2* | 1,07E-20 | 1,05E-19 | 5,1 |  | 1,3 | 3,11E-06 | 1,01E-05 |
| *JAK1* | 1,13E-20 | 1,11E-19 | 2,1 |  | -2,1 | 4,87E-01 | 5,58E-01 |
| *CXorf38* | 1,20E-20 | 1,17E-19 | 3,2 |  | 3,4 | 1,26E-11 | 7,34E-11 |
| *SH2D5* | 1,23E-20 | 1,21E-19 | -5,4 |  | 2,1 | 2,85E-02 | 4,68E-02 |
| *EPM2AIP1* | 1,27E-20 | 1,24E-19 | -2,9 |  | -4,0 | 1,14E-19 | 1,19E-18 |
| *VMP1* | 1,32E-20 | 1,29E-19 | 2,5 |  | -2,6 | 2,21E-02 | 3,73E-02 |
| *FLI1* | 1,47E-20 | 1,44E-19 | 3,2 |  | 4,4 | 4,42E-26 | 6,51E-25 |
| *NIPSNAP1* | 1,51E-20 | 1,48E-19 | -3,1 |  | -2,6 | 6,85E-03 | 1,29E-02 |
| *TRIM47* | 1,73E-20 | 1,69E-19 | 34,5 |  | 3,6 | 5,12E-22 | 6,04E-21 |
| *NAGK* | 1,87E-20 | 1,82E-19 | 2,9 |  | 2,1 | 1,82E-26 | 2,75E-25 |
| *TRPM4* | 1,89E-20 | 1,84E-19 | 3,3 |  | 2,6 | 1,46E-14 | 1,05E-13 |
| *MDGA1* | 1,91E-20 | 1,86E-19 | 5,4 |  | 0,2 | 6,21E-01 | 6,81E-01 |
| *TRAM2* | 1,99E-20 | 1,93E-19 | -2,6 |  | -2,9 | 3,35E-11 | 1,86E-10 |
| *CCDC68* | 2,26E-20 | 2,19E-19 | 5,0 |  | 1,3 | 9,38E-04 | 2,07E-03 |
| *ABHD12* | 2,30E-20 | 2,22E-19 | 2,2 |  | 1,5 | 1,58E-06 | 5,35E-06 |
| *SPOCD1* | 2,46E-20 | 2,38E-19 | 2,1 |  | 0,3 | 1,03E-02 | 1,87E-02 |
| *POMP* | 2,56E-20 | 2,47E-19 | 2,4 |  | 2,5 | 5,29E-13 | 3,40E-12 |
| *TMEM192* | 2,59E-20 | 2,51E-19 | -2,8 |  | -5,7 | 1,69E-14 | 1,21E-13 |
| *RAP1B* | 2,77E-20 | 2,67E-19 | 2,5 |  | 0,4 | 9,54E-03 | 1,74E-02 |
| *PGM2* | 2,81E-20 | 2,71E-19 | 2,9 |  | 0,9 | 3,68E-05 | 1,04E-04 |
| *PLPP4* | 2,89E-20 | 2,78E-19 | -8,0 |  | -10,5 | 1,10E-08 | 4,78E-08 |
| *LINC01137* | 2,97E-20 | 2,86E-19 | 12,8 |  | 20,1 | 3,10E-39 | 8,11E-38 |
| *HNRNPF* | 3,22E-20 | 3,10E-19 | 2,3 |  | 0,3 | 1,26E-01 | 1,74E-01 |
| *ENSG00000258666* | 3,63E-20 | 3,49E-19 | 693,7 |  | 8026,9 | 9,12E-24 | 1,18E-22 |
| *SYNE3* | 3,66E-20 | 3,51E-19 | -4,8 |  | -7,0 | 4,27E-45 | 1,36E-43 |
| *TMEM117* | 3,68E-20 | 3,53E-19 | -5,6 |  | -21,1 | 1,30E-30 | 2,35E-29 |
| *FERMT2* | 4,44E-20 | 4,25E-19 | 2,6 |  | 0,6 | 1,97E-04 | 4,88E-04 |
| *OPHN1* | 4,60E-20 | 4,41E-19 | -2,7 |  | -3,1 | 2,89E-04 | 6,97E-04 |
| *DDX10* | 4,72E-20 | 4,51E-19 | -3,4 |  | -2,7 | 2,79E-03 | 5,66E-03 |
| *RPLP0P2* | 4,73E-20 | 4,53E-19 | 24,2 |  | 1,0 | 1,33E-03 | 2,87E-03 |
| *UBFD1* | 4,77E-20 | 4,56E-19 | -2,1 |  | -2,4 | 1,88E-02 | 3,23E-02 |
| *ENSG00000271581* | 5,33E-20 | 5,09E-19 | 4989,1 |  | 34,4 | 1,51E-14 | 1,08E-13 |
| *PRR5L* | 5,59E-20 | 5,33E-19 | -4,9 |  | -3,1 | 3,05E-03 | 6,15E-03 |
| *ABRACL* | 5,65E-20 | 5,39E-19 | 2,9 |  | 1,1 | 4,01E-09 | 1,83E-08 |
| *ZNF710* | 5,84E-20 | 5,56E-19 | 3,7 |  | 5,7 | 1,98E-31 | 3,75E-30 |
| *FZD1* | 6,06E-20 | 5,76E-19 | 3,1 |  | 0,6 | 3,00E-04 | 7,22E-04 |
| *HEPH* | 6,11E-20 | 5,81E-19 | -4,0 |  | -3,8 | 2,33E-16 | 1,92E-15 |
| *UBE2Z* | 6,22E-20 | 5,91E-19 | 2,0 |  | 2,4 | 6,41E-21 | 7,17E-20 |
| *GUCD1* | 6,61E-20 | 6,27E-19 | 2,3 |  | 1,4 | 3,34E-16 | 2,71E-15 |
| *C20orf27* | 7,09E-20 | 6,72E-19 | -4,9 |  | -4,1 | 3,69E-07 | 1,36E-06 |
| *CAV1* | 7,36E-20 | 6,97E-19 | -1,9 |  | -2,2 | 6,41E-01 | 6,99E-01 |
| *VPS13A* | 7,46E-20 | 7,06E-19 | 4,2 |  | -3,9 | 5,33E-05 | 1,45E-04 |
| *ANPEP* | 7,70E-20 | 7,28E-19 | 7,7 |  | 7,0 | 1,42E-14 | 1,02E-13 |
| *LRP6* | 7,71E-20 | 7,29E-19 | -3,3 |  | -5,8 | 1,35E-17 | 1,23E-16 |
| *LURAP1L* | 7,85E-20 | 7,41E-19 | -6,9 |  | 3,3 | 3,58E-28 | 5,85E-27 |
| *WDR12* | 7,87E-20 | 7,43E-19 | -2,8 |  | -5,2 | 1,48E-27 | 2,36E-26 |
| *TSKU* | 8,42E-20 | 7,94E-19 | 3,5 |  | 4,0 | 1,80E-17 | 1,61E-16 |
| *ASPHD2* | 9,00E-20 | 8,48E-19 | 4,6 |  | 4,6 | 1,39E-11 | 8,05E-11 |
| *PRICKLE1* | 9,03E-20 | 8,50E-19 | -10,3 |  | -8,8 | 2,55E-40 | 6,89E-39 |
| *SPAG9* | 9,83E-20 | 9,25E-19 | 2,2 |  | -2,2 | 1,69E-01 | 2,26E-01 |
| *BHLHE40* | 1,04E-19 | 9,80E-19 | 7,3 |  | 0,8 | 2,39E-02 | 4,00E-02 |
| *AGBL5* | 1,06E-19 | 9,99E-19 | -3,9 |  | -4,8 | 5,52E-16 | 4,41E-15 |
| *GYS1* | 1,10E-19 | 1,04E-18 | 2,7 |  | 11,7 | 4,02E-10 | 2,03E-09 |
| *RHOD* | 1,14E-19 | 1,07E-18 | -7,2 |  | -6,3 | 2,93E-10 | 1,49E-09 |
| *TBKBP1* | 1,18E-19 | 1,10E-18 | 3,4 |  | 1,8 | 1,14E-06 | 3,94E-06 |
| *EGF* | 1,19E-19 | 1,11E-18 | -14,6 |  | -3,6 | 5,34E-02 | 8,19E-02 |
| *SETX* | 1,28E-19 | 1,20E-18 | 2,4 |  | 0,2 | 3,93E-01 | 4,65E-01 |
| *DGLUCY* | 1,32E-19 | 1,23E-18 | 3,1 |  | 0,6 | 2,61E-06 | 8,58E-06 |
| *ACAT1* | 1,34E-19 | 1,25E-18 | -3,0 |  | -4,8 | 5,92E-15 | 4,38E-14 |
| *PGPEP1* | 1,36E-19 | 1,27E-18 | -3,3 |  | -4,3 | 3,16E-15 | 2,37E-14 |
| *PHLDB2* | 1,37E-19 | 1,28E-18 | -2,5 |  | -2,3 | 6,99E-02 | 1,04E-01 |
| *CYTH3* | 1,41E-19 | 1,32E-18 | -2,6 |  | -4,7 | 4,47E-12 | 2,69E-11 |
| *RPL6* | 1,43E-19 | 1,33E-18 | -2,8 |  | -7,7 | 5,11E-74 | 3,25E-72 |
| *NTPCR* | 1,44E-19 | 1,34E-18 | -3,2 |  | -2,9 | 7,17E-07 | 2,54E-06 |
| *ARHGAP24* | 1,45E-19 | 1,35E-18 | 2,9 |  | -2,8 | 4,68E-03 | 9,08E-03 |
| *PFKFB2* | 1,52E-19 | 1,41E-18 | -6,7 |  | -18,5 | 1,00E-42 | 2,99E-41 |
| *ADAM12* | 1,57E-19 | 1,45E-18 | -8,1 |  | -5,1 | 5,65E-13 | 3,63E-12 |
| *FDXR* | 1,59E-19 | 1,47E-18 | -6,5 |  | -3,8 | 5,99E-06 | 1,89E-05 |
| *SERPINB9* | 1,62E-19 | 1,50E-18 | 13,0 |  | 4,8 | 6,71E-04 | 1,51E-03 |
| *SYNGR3* | 1,77E-19 | 1,64E-18 | 35,3 |  | 69,7 | 4,37E-12 | 2,63E-11 |
| *LRPPRC* | 1,77E-19 | 1,64E-18 | -2,1 |  | -3,7 | 5,77E-13 | 3,70E-12 |
| *IRAK1* | 1,84E-19 | 1,70E-18 | 3,3 |  | 0,2 | 5,08E-02 | 7,83E-02 |
| *IRF3* | 1,86E-19 | 1,72E-18 | 3,1 |  | 2,8 | 1,53E-22 | 1,86E-21 |
| *PCBP4* | 1,98E-19 | 1,83E-18 | -3,3 |  | -2,6 | 1,21E-02 | 2,16E-02 |
| *KCTD9* | 1,99E-19 | 1,83E-18 | 3,0 |  | 1,7 | 2,17E-09 | 1,02E-08 |
| *EBI3* | 2,46E-19 | 2,26E-18 | 228,3 |  | 19190,5 | 3,45E-30 | 6,09E-29 |
| *RPLP0* | 2,55E-19 | 2,35E-18 | -2,9 |  | -6,8 | 3,32E-33 | 6,88E-32 |
| *JADE1* | 2,73E-19 | 2,51E-18 | -4,5 |  | -7,5 | 7,20E-27 | 1,11E-25 |
| *TMEM50A* | 2,82E-19 | 2,59E-18 | 2,0 |  | 1,7 | 1,37E-31 | 2,63E-30 |
| *PYCR1* | 2,85E-19 | 2,62E-18 | -2,8 |  | -11,1 | 1,98E-25 | 2,85E-24 |
| *FTL* | 2,88E-19 | 2,64E-18 | -2,5 |  | -4,5 | 4,15E-29 | 7,03E-28 |
| *RPL32* | 2,91E-19 | 2,66E-18 | -2,7 |  | -5,2 | 1,63E-27 | 2,60E-26 |
| *TPD52L1* | 2,91E-19 | 2,66E-18 | -52,9 |  | -113,1 | 5,82E-92 | 4,93E-90 |
| *ZNF521* | 2,94E-19 | 2,69E-18 | -5,4 |  | -53,1 | 6,21E-06 | 1,95E-05 |
| *TMEM63A* | 2,95E-19 | 2,70E-18 | 4,5 |  | 2,9 | 1,89E-11 | 1,08E-10 |
| *GALNT7* | 3,01E-19 | 2,75E-18 | -3,2 |  | -4,9 | 9,66E-11 | 5,14E-10 |
| *FBXL3* | 3,08E-19 | 2,81E-18 | 2,7 |  | 0,9 | 1,56E-04 | 3,94E-04 |
| *SNHG8* | 3,25E-19 | 2,97E-18 | -3,5 |  | -8,1 | 8,16E-55 | 3,31E-53 |
| *ZFYVE28* | 3,38E-19 | 3,08E-18 | 24,6 |  | 2,6 | 2,99E-14 | 2,11E-13 |
| *FGF1* | 3,40E-19 | 3,10E-18 | -3,6 |  | -4,5 | 3,25E-03 | 6,52E-03 |
| *PCYOX1* | 3,46E-19 | 3,15E-18 | -2,6 |  | -7,9 | 1,70E-31 | 3,23E-30 |
| *SOX9* | 3,64E-19 | 3,32E-18 | -7,0 |  | -3,3 | 4,14E-05 | 1,16E-04 |
| *ETS2* | 3,75E-19 | 3,41E-18 | 4,2 |  | 4,0 | 5,36E-07 | 1,93E-06 |
| *HAGH* | 3,84E-19 | 3,48E-18 | 2,8 |  | 2,5 | 1,60E-15 | 1,23E-14 |
| *KCTD14* | 3,97E-19 | 3,60E-18 | 1813,3 |  | 1432,5 | 1,18E-121 | 1,67E-119 |
| *MARCKS* | 4,09E-19 | 3,71E-18 | 2,1 |  | 2,4 | 1,98E-10 | 1,03E-09 |
| *ANK2* | 4,12E-19 | 3,74E-18 | 2,6 |  | 0,5 | 1,67E-04 | 4,20E-04 |
| *NQO1* | 4,20E-19 | 3,80E-18 | -3,0 |  | -2,6 | 1,24E-05 | 3,73E-05 |
| *MAML2* | 4,27E-19 | 3,87E-18 | 4,3 |  | 2,6 | 1,11E-14 | 8,02E-14 |
| *GTPBP2* | 4,29E-19 | 3,88E-18 | 2,6 |  | 4,8 | 7,66E-45 | 2,43E-43 |
| *GCLM* | 4,31E-19 | 3,89E-18 | 3,0 |  | 0,8 | 1,17E-07 | 4,56E-07 |
| *PPARA* | 4,49E-19 | 4,05E-18 | 3,7 |  | 0,1 | 6,49E-01 | 7,06E-01 |
| *CPT1A* | 4,79E-19 | 4,32E-18 | 3,7 |  | 0,1 | 2,77E-01 | 3,47E-01 |
| *EID1* | 4,83E-19 | 4,35E-18 | -2,3 |  | -2,4 | 2,16E-02 | 3,66E-02 |
| *SHISA5* | 5,11E-19 | 4,60E-18 | -2,2 |  | 0,1 | 6,13E-01 | 6,74E-01 |
| *PC* | 5,38E-19 | 4,84E-18 | -8,0 |  | -3,3 | 2,71E-04 | 6,56E-04 |
| *PCNX2* | 5,78E-19 | 5,20E-18 | -3,6 |  | -5,5 | 1,09E-16 | 9,21E-16 |
| *TRIM8* | 6,33E-19 | 5,68E-18 | 2,5 |  | 1,8 | 1,82E-29 | 3,14E-28 |
| *NXPE3* | 6,36E-19 | 5,70E-18 | -2,6 |  | -2,7 | 2,89E-06 | 9,45E-06 |
| *GAS5* | 6,51E-19 | 5,84E-18 | -2,7 |  | -6,1 | 6,63E-42 | 1,92E-40 |
| *ENSG00000272941* | 6,75E-19 | 6,05E-18 | 5001,0 |  | 37,5 | 3,36E-14 | 2,35E-13 |
| *KBTBD2* | 6,85E-19 | 6,14E-18 | 3,0 |  | 1,2 | 3,77E-09 | 1,72E-08 |
| *HEXD* | 6,98E-19 | 6,25E-18 | 6,9 |  | 3,6 | 4,46E-11 | 2,44E-10 |
| *WDR55* | 7,04E-19 | 6,29E-18 | 2,4 |  | 0,7 | 7,00E-06 | 2,19E-05 |
| *BASP1* | 7,21E-19 | 6,44E-18 | 2,0 |  | 0,1 | 6,59E-01 | 7,15E-01 |
| *HLA-J* | 7,31E-19 | 6,52E-18 | 4735,2 |  | 2151,7 | 3,96E-35 | 8,97E-34 |
| *SNU13* | 8,15E-19 | 7,27E-18 | -2,3 |  | -2,2 | 1,69E-01 | 2,26E-01 |
| *PICALM* | 8,18E-19 | 7,29E-18 | 2,3 |  | 0,3 | 8,84E-02 | 1,28E-01 |
| *TMEM87A* | 8,57E-19 | 7,63E-18 | 2,3 |  | 1,1 | 3,36E-10 | 1,71E-09 |
| *SLC25A22* | 8,71E-19 | 7,75E-18 | 3,5 |  | 6,2 | 4,89E-16 | 3,91E-15 |
| *ATP1B3* | 9,55E-19 | 8,48E-18 | -2,0 |  | -2,6 | 9,55E-06 | 2,93E-05 |
| *AGTR1* | 1,05E-18 | 9,31E-18 | 6,3 |  | 0,6 | 2,03E-01 | 2,64E-01 |
| *RPL15* | 1,17E-18 | 1,04E-17 | -2,1 |  | -4,3 | 6,92E-51 | 2,55E-49 |
| *NDUFV3* | 1,24E-18 | 1,10E-17 | -3,1 |  | -3,1 | 5,30E-05 | 1,44E-04 |
| *RECQL* | 1,33E-18 | 1,17E-17 | 2,6 |  | -2,2 | 1,66E-01 | 2,23E-01 |
| *MMP14* | 1,47E-18 | 1,30E-17 | 2,6 |  | -2,8 | 1,29E-12 | 8,06E-12 |
| *RPL3* | 1,48E-18 | 1,31E-17 | -2,4 |  | -7,6 | 6,90E-64 | 3,47E-62 |
| *SLC20A1* | 1,58E-18 | 1,39E-17 | -2,7 |  | -5,1 | 2,90E-11 | 1,62E-10 |
| *TNS3* | 1,63E-18 | 1,44E-17 | 2,7 |  | 69,0 | 0,00E+00 | 0,00E+00 |
| *SH3BP5* | 1,67E-18 | 1,47E-17 | -3,1 |  | -3,6 | 7,24E-12 | 4,29E-11 |
| *TMEM87B* | 1,73E-18 | 1,52E-17 | 2,5 |  | 1,6 | 1,90E-08 | 8,08E-08 |
| *ENSG00000254287* | 1,80E-18 | 1,58E-17 | 4250,3 |  | 10513,5 | 1,68E-24 | 2,26E-23 |
| *SCG2* | 1,86E-18 | 1,64E-17 | -86,7 |  | -20,2 | 3,94E-19 | 3,98E-18 |
| *IP6K2* | 1,88E-18 | 1,66E-17 | -2,5 |  | -2,1 | 5,75E-01 | 6,40E-01 |
| *PDCD4* | 2,02E-18 | 1,78E-17 | -2,8 |  | -3,1 | 5,20E-06 | 1,65E-05 |
| *AP5B1* | 2,04E-18 | 1,79E-17 | 4,1 |  | 9,8 | 8,05E-42 | 2,32E-40 |
| *IL17RD* | 2,10E-18 | 1,85E-17 | -10,0 |  | -16,4 | 2,36E-17 | 2,09E-16 |
| *UBE2E1* | 2,11E-18 | 1,85E-17 | 2,3 |  | 2,2 | 1,66E-24 | 2,23E-23 |
| *HSPB8* | 2,13E-18 | 1,86E-17 | -7,6 |  | 0,0 | 9,85E-01 | 9,88E-01 |
| *ALDH3A2* | 2,16E-18 | 1,89E-17 | -2,8 |  | -3,7 | 4,14E-10 | 2,09E-09 |
| *TNC* | 2,17E-18 | 1,90E-17 | 3,6 |  | -2,6 | 6,45E-05 | 1,73E-04 |
| *ENSG00000266976* | 2,30E-18 | 2,01E-17 | 3983,2 |  | 26891,8 | 5,25E-32 | 1,03E-30 |
| *B3GALNT2* | 2,36E-18 | 2,06E-17 | -2,9 |  | -5,5 | 9,17E-20 | 9,57E-19 |
| *DTNA* | 2,51E-18 | 2,19E-17 | -6,0 |  | -4,2 | 5,87E-04 | 1,34E-03 |
| *NEU3* | 2,60E-18 | 2,26E-17 | -5,1 |  | -4,0 | 8,62E-06 | 2,66E-05 |
| *USP31* | 2,60E-18 | 2,27E-17 | 3,9 |  | 2,0 | 9,24E-13 | 5,85E-12 |
| *LAYN* | 2,74E-18 | 2,39E-17 | -2,5 |  | 5,9 | 1,38E-41 | 3,93E-40 |
| *XBP1* | 3,17E-18 | 2,75E-17 | 2,7 |  | 1,7 | 3,09E-07 | 1,15E-06 |
| *STK10* | 3,21E-18 | 2,79E-17 | 2,4 |  | 5,6 | 8,26E-45 | 2,61E-43 |
| *TRHDE-AS1* | 3,33E-18 | 2,89E-17 | -30,4 |  | -120,5 | 2,38E-15 | 1,80E-14 |
| *NCEH1* | 3,43E-18 | 2,98E-17 | 3,4 |  | 10,4 | 1,04E-16 | 8,82E-16 |
| *NIT2* | 3,48E-18 | 3,02E-17 | 3,2 |  | 0,0 | 8,77E-01 | 9,03E-01 |
| *HDLBP* | 3,58E-18 | 3,10E-17 | -2,0 |  | -5,0 | 8,54E-36 | 1,98E-34 |
| *ALDH2* | 3,63E-18 | 3,14E-17 | -5,3 |  | -3,5 | 8,28E-04 | 1,84E-03 |
| *ITGA11* | 3,85E-18 | 3,33E-17 | -4,3 |  | -3,3 | 3,02E-05 | 8,62E-05 |
| *RPL27A* | 3,86E-18 | 3,34E-17 | -2,8 |  | -5,3 | 4,31E-33 | 8,86E-32 |
| *SCAND2P* | 3,98E-18 | 3,44E-17 | 5,0 |  | 2,2 | 4,52E-10 | 2,27E-09 |
| *KDM3A* | 4,06E-18 | 3,51E-17 | 2,8 |  | 1,0 | 3,41E-08 | 1,41E-07 |
| *IER5L* | 4,07E-18 | 3,51E-17 | -5,7 |  | -3,5 | 1,01E-05 | 3,10E-05 |
| *RARRES1* | 4,10E-18 | 3,54E-17 | 1916,1 |  | 6,9 | 5,99E-11 | 3,24E-10 |
| *MSL3* | 4,19E-18 | 3,61E-17 | 3,1 |  | 2,9 | 5,88E-31 | 1,08E-29 |
| *CASTOR2* | 4,26E-18 | 3,67E-17 | -5,7 |  | -18,6 | 2,77E-18 | 2,65E-17 |
| *CENPV* | 4,30E-18 | 3,70E-17 | -7,8 |  | -15,1 | 1,30E-11 | 7,57E-11 |
| *TMEM106A* | 4,33E-18 | 3,72E-17 | 5,0 |  | 3,9 | 1,86E-13 | 1,24E-12 |
| *SEPHS2* | 4,65E-18 | 3,99E-17 | 4,9 |  | -2,1 | 7,02E-01 | 7,53E-01 |
| *SWAP70* | 4,79E-18 | 4,11E-17 | 2,4 |  | -2,1 | 5,14E-01 | 5,84E-01 |
| *CD58* | 4,86E-18 | 4,17E-17 | 6,3 |  | 2,6 | 5,49E-07 | 1,97E-06 |
| *EML1* | 4,92E-18 | 4,22E-17 | -2,9 |  | -5,4 | 3,42E-17 | 2,99E-16 |
| *C1RL-AS1* | 5,10E-18 | 4,37E-17 | 79,7 |  | 0,7 | 2,23E-01 | 2,87E-01 |
| *TJP1* | 5,32E-18 | 4,55E-17 | 2,1 |  | 0,7 | 8,78E-06 | 2,71E-05 |
| *TMEM43* | 5,64E-18 | 4,83E-17 | 2,2 |  | 0,5 | 3,51E-07 | 1,29E-06 |
| *STAT4* | 5,81E-18 | 4,97E-17 | 7,1 |  | 13,8 | 1,19E-11 | 6,95E-11 |
| *ARMH4* | 5,87E-18 | 5,01E-17 | -4,5 |  | -18,6 | 1,19E-25 | 1,72E-24 |
| *TGM2* | 5,97E-18 | 5,09E-17 | 2,9 |  | 27,1 | 3,59E-15 | 2,68E-14 |
| *LTA4H* | 6,18E-18 | 5,27E-17 | -2,5 |  | -3,6 | 4,09E-15 | 3,05E-14 |
| *LARP6* | 6,22E-18 | 5,30E-17 | -2,3 |  | -2,9 | 4,29E-06 | 1,37E-05 |
| *VEGFA* | 6,32E-18 | 5,38E-17 | 2,7 |  | 0,2 | 4,68E-01 | 5,39E-01 |
| *ENSG00000262879* | 6,95E-18 | 5,91E-17 | -3,7 |  | -7,4 | 1,98E-28 | 3,27E-27 |
| *CX3CL1* | 6,97E-18 | 5,92E-17 | 6900,7 |  | 1021,2 | 2,03E-31 | 3,84E-30 |
| *RALB* | 7,06E-18 | 6,00E-17 | 2,4 |  | 1,7 | 3,00E-14 | 2,11E-13 |
| *FARSB* | 7,13E-18 | 6,06E-17 | -2,4 |  | -3,6 | 3,19E-07 | 1,18E-06 |
| *MAN2B1* | 7,48E-18 | 6,35E-17 | 2,6 |  | 0,6 | 3,79E-02 | 6,02E-02 |
| *DUSP6* | 7,48E-18 | 6,35E-17 | 3,5 |  | 8,1 | 7,72E-21 | 8,58E-20 |
| *LYRM7* | 7,51E-18 | 6,36E-17 | -3,4 |  | -6,0 | 1,82E-10 | 9,48E-10 |
| *RAB27A* | 7,89E-18 | 6,68E-17 | 7,2 |  | 1,0 | 2,87E-05 | 8,19E-05 |
| *OSBPL1A* | 7,93E-18 | 6,71E-17 | -2,6 |  | -3,5 | 1,30E-19 | 1,35E-18 |
| *QKI* | 8,18E-18 | 6,92E-17 | 2,9 |  | 1,4 | 1,22E-06 | 4,19E-06 |
| *NPM3* | 8,34E-18 | 7,05E-17 | -5,2 |  | -11,6 | 6,18E-04 | 1,40E-03 |
| *ERG28* | 8,35E-18 | 7,05E-17 | -3,6 |  | -2,7 | 1,94E-02 | 3,33E-02 |
| *MXD4* | 8,40E-18 | 7,09E-17 | -2,6 |  | -2,9 | 9,27E-05 | 2,42E-04 |
| *TMEM35A* | 8,48E-18 | 7,15E-17 | -19,2 |  | -55,8 | 8,36E-22 | 9,78E-21 |
| *ATP11B* | 8,60E-18 | 7,24E-17 | 2,5 |  | 0,1 | 5,29E-01 | 5,98E-01 |
| *GNL3L* | 8,86E-18 | 7,45E-17 | -2,4 |  | -4,2 | 5,46E-12 | 3,26E-11 |
| *IFI30* | 8,99E-18 | 7,55E-17 | 3478,8 |  | 49,9 | 3,39E-16 | 2,75E-15 |
| *EVI5L* | 9,32E-18 | 7,82E-17 | -2,9 |  | -2,2 | 2,57E-01 | 3,25E-01 |
| *ARNT2* | 9,36E-18 | 7,86E-17 | -7,0 |  | -14,2 | 1,72E-41 | 4,84E-40 |
| *TECR* | 9,69E-18 | 8,13E-17 | -3,6 |  | 1,0 | 4,95E-05 | 1,36E-04 |
| *C1GALT1C1* | 9,87E-18 | 8,27E-17 | 3,4 |  | 4,0 | 1,13E-26 | 1,71E-25 |
| *CASP8* | 1,04E-17 | 8,74E-17 | 2,8 |  | 2,7 | 1,74E-24 | 2,33E-23 |
| *TRAF3IP2* | 1,05E-17 | 8,82E-17 | 2,9 |  | 5,1 | 8,40E-54 | 3,32E-52 |
| *EHD1* | 1,12E-17 | 9,33E-17 | 2,5 |  | 0,5 | 1,18E-02 | 2,11E-02 |
| *ZNF731P* | 1,12E-17 | 9,36E-17 | 4,6 |  | 0,0 | 9,21E-01 | 9,39E-01 |
| *RUNX2* | 1,14E-17 | 9,49E-17 | -25,4 |  | -5,3 | 1,38E-14 | 9,97E-14 |
| *CUL4B* | 1,17E-17 | 9,73E-17 | -2,2 |  | -2,8 | 1,98E-03 | 4,14E-03 |
| *RNF149* | 1,23E-17 | 1,03E-16 | 2,4 |  | 3,0 | 1,77E-20 | 1,93E-19 |
| *HSPA12A* | 1,23E-17 | 1,03E-16 | -5,0 |  | -11,9 | 3,37E-25 | 4,77E-24 |
| *ABCA3* | 1,24E-17 | 1,04E-16 | 103,9 |  | 2,2 | 1,05E-02 | 1,90E-02 |
| *ARMCX3* | 1,30E-17 | 1,08E-16 | -2,4 |  | -4,7 | 4,66E-15 | 3,46E-14 |
| *FAM216A* | 1,30E-17 | 1,08E-16 | -6,3 |  | -3,4 | 4,49E-04 | 1,05E-03 |
| *DGKG* | 1,31E-17 | 1,09E-16 | -4,9 |  | 8,7 | 1,06E-24 | 1,45E-23 |
| *SMYD2* | 1,41E-17 | 1,17E-16 | 2,7 |  | 1,3 | 4,71E-11 | 2,57E-10 |
| *EPHX1* | 1,41E-17 | 1,17E-16 | -8,7 |  | -12,2 | 3,95E-19 | 3,99E-18 |
| *RELT* | 1,41E-17 | 1,17E-16 | 4,3 |  | 6,1 | 2,05E-07 | 7,74E-07 |
| *KLHL9* | 1,43E-17 | 1,19E-16 | -2,2 |  | -3,0 | 3,05E-08 | 1,27E-07 |
| *RPS6* | 1,44E-17 | 1,19E-16 | -3,0 |  | -9,2 | 4,77E-96 | 4,54E-94 |
| *LRATD1* | 1,46E-17 | 1,21E-16 | 4,7 |  | 1,2 | 4,15E-02 | 6,53E-02 |
| *CD2AP* | 1,54E-17 | 1,27E-16 | 4,0 |  | 3,7 | 5,47E-23 | 6,80E-22 |
| *RPS3* | 1,60E-17 | 1,32E-16 | -2,7 |  | -5,7 | 9,44E-42 | 2,71E-40 |
| *DCLRE1C* | 1,70E-17 | 1,40E-16 | 2,8 |  | -2,3 | 2,84E-01 | 3,54E-01 |
| *KHNYN* | 1,70E-17 | 1,40E-16 | 2,1 |  | 0,3 | 8,47E-02 | 1,23E-01 |
| *PPP2R5C* | 1,90E-17 | 1,57E-16 | -2,1 |  | -3,3 | 6,58E-16 | 5,22E-15 |
| *RAB31* | 1,92E-17 | 1,58E-16 | 2,2 |  | 0,4 | 8,48E-02 | 1,23E-01 |
| *EXOC2* | 1,97E-17 | 1,62E-16 | 2,4 |  | 1,4 | 6,40E-23 | 7,94E-22 |
| *C7orf50* | 1,98E-17 | 1,62E-16 | -2,4 |  | -2,1 | 6,98E-01 | 7,50E-01 |
| *CD99L2* | 2,01E-17 | 1,65E-16 | -2,1 |  | -2,6 | 9,41E-04 | 2,08E-03 |
| *FAM78B* | 2,04E-17 | 1,67E-16 | 577,5 |  | 418,1 | 1,58E-41 | 4,47E-40 |
| *SOD3* | 2,11E-17 | 1,73E-16 | 221,2 |  | -2,5 | 2,34E-01 | 2,99E-01 |
| *SNRNP25* | 2,14E-17 | 1,75E-16 | -3,3 |  | -6,7 | 7,60E-20 | 7,95E-19 |
| *ACKR4* | 2,27E-17 | 1,86E-16 | 123,5 |  | 14,6 | 8,22E-14 | 5,60E-13 |
| *AVPI1* | 2,27E-17 | 1,86E-16 | -6,7 |  | 1,0 | 8,01E-05 | 2,12E-04 |
| *ARID5B* | 2,30E-17 | 1,88E-16 | 2,7 |  | 2,6 | 1,48E-16 | 1,24E-15 |
| *ADAMTS4* | 2,43E-17 | 1,98E-16 | 221,0 |  | 69,7 | 3,88E-48 | 1,37E-46 |
| *SORBS2* | 2,47E-17 | 2,01E-16 | -6,4 |  | -25,9 | 2,48E-52 | 9,54E-51 |
| *MRPL41* | 2,53E-17 | 2,06E-16 | -2,4 |  | -2,5 | 1,33E-02 | 2,35E-02 |
| *HMGN4* | 2,55E-17 | 2,08E-16 | 2,3 |  | 0,3 | 6,70E-02 | 1,00E-01 |
| *SLC22A17* | 2,63E-17 | 2,14E-16 | -5,1 |  | -4,8 | 3,10E-10 | 1,58E-09 |
| *EIF4A3* | 2,75E-17 | 2,24E-16 | 2,6 |  | 1,2 | 5,77E-11 | 3,12E-10 |
| *IL31RA* | 3,06E-17 | 2,49E-16 | 3,1 |  | 355,8 | 2,07E-71 | 1,24E-69 |
| *BTG2* | 3,16E-17 | 2,57E-16 | -7,5 |  | -3,1 | 2,00E-02 | 3,42E-02 |
| *MYADM* | 3,22E-17 | 2,62E-16 | 3,5 |  | 1,2 | 4,47E-03 | 8,72E-03 |
| *CTSC* | 3,23E-17 | 2,62E-16 | 3,2 |  | 5,3 | 1,53E-11 | 8,83E-11 |
| *APPL2* | 3,25E-17 | 2,64E-16 | -2,7 |  | -10,0 | 4,21E-37 | 1,02E-35 |
| *VGLL3* | 3,31E-17 | 2,69E-16 | -4,6 |  | -3,2 | 1,48E-04 | 3,76E-04 |
| *RHOB* | 3,67E-17 | 2,97E-16 | -2,2 |  | 0,6 | 1,93E-02 | 3,30E-02 |
| *AK3* | 3,80E-17 | 3,07E-16 | 2,8 |  | 1,0 | 2,16E-03 | 4,50E-03 |
| *ELF1* | 3,80E-17 | 3,07E-16 | 2,8 |  | 2,1 | 2,57E-11 | 1,44E-10 |
| *GADD45B* | 4,19E-17 | 3,38E-16 | 4,4 |  | 0,2 | 1,99E-01 | 2,60E-01 |
| *LRP4* | 4,24E-17 | 3,42E-16 | -9,5 |  | -85,1 | 7,55E-05 | 2,00E-04 |
| *LINC01963* | 4,27E-17 | 3,45E-16 | -5,9 |  | -44,8 | 4,51E-20 | 4,78E-19 |
| *ST3GAL5* | 4,48E-17 | 3,61E-16 | -3,6 |  | -5,5 | 1,51E-26 | 2,28E-25 |
| *KIF20A* | 4,62E-17 | 3,72E-16 | -5,2 |  | -3,1 | 7,30E-02 | 1,08E-01 |
| *SLC9A8* | 4,76E-17 | 3,83E-16 | 4,4 |  | 3,7 | 1,80E-16 | 1,49E-15 |
| *GPD1L* | 4,77E-17 | 3,84E-16 | -3,0 |  | -12,5 | 2,69E-18 | 2,58E-17 |
| *MTMR14* | 4,81E-17 | 3,87E-16 | 2,5 |  | 0,9 | 1,04E-05 | 3,17E-05 |
| *RPL14* | 4,92E-17 | 3,96E-16 | -2,6 |  | -4,6 | 7,11E-41 | 1,96E-39 |
| *FAHD2A* | 5,62E-17 | 4,51E-16 | -2,9 |  | -2,5 | 5,76E-02 | 8,76E-02 |
| *DOCK9* | 6,19E-17 | 4,96E-16 | 3,2 |  | 1,9 | 2,02E-07 | 7,66E-07 |
| *IL12A* | 6,20E-17 | 4,97E-16 | 9,9 |  | 22,4 | 2,69E-17 | 2,37E-16 |
| *STARD8* | 6,47E-17 | 5,18E-16 | 3,5 |  | 0,9 | 5,42E-03 | 1,04E-02 |
| *ITSN1* | 6,50E-17 | 5,20E-16 | -2,4 |  | -2,5 | 1,21E-03 | 2,62E-03 |
| *DESI1* | 6,82E-17 | 5,45E-16 | 2,0 |  | 1,9 | 8,78E-12 | 5,16E-11 |
| *MINDY2* | 6,90E-17 | 5,51E-16 | 3,1 |  | -2,0 | 9,13E-01 | 9,33E-01 |
| *DAAM2* | 7,03E-17 | 5,61E-16 | -3,1 |  | -3,6 | 6,89E-15 | 5,07E-14 |
| *MTURN* | 7,08E-17 | 5,65E-16 | -4,3 |  | -8,7 | 4,33E-41 | 1,21E-39 |
| *BRD2* | 7,14E-17 | 5,70E-16 | 2,0 |  | 1,6 | 2,70E-21 | 3,08E-20 |
| *PANK1* | 8,28E-17 | 6,60E-16 | -9,4 |  | -7,0 | 5,31E-09 | 2,39E-08 |
| *ARSA* | 8,50E-17 | 6,77E-16 | 5,3 |  | 2,4 | 4,38E-11 | 2,40E-10 |
| *ITPRIPL2* | 8,57E-17 | 6,82E-16 | 2,7 |  | -3,2 | 1,48E-11 | 8,55E-11 |
| *ABHD10* | 8,67E-17 | 6,89E-16 | -3,1 |  | -4,4 | 3,12E-07 | 1,16E-06 |
| *ENSG00000236453* | 8,79E-17 | 6,99E-16 | 21,4 |  | 13,0 | 1,35E-04 | 3,45E-04 |
| *KCTD7* | 9,38E-17 | 7,45E-16 | -4,3 |  | -10,3 | 3,72E-14 | 2,59E-13 |
| *RASA2* | 9,61E-17 | 7,63E-16 | 2,9 |  | 2,7 | 2,26E-11 | 1,28E-10 |
| *ENSG00000279348* | 9,71E-17 | 7,70E-16 | -7,8 |  | -241,8 | 7,01E-19 | 6,98E-18 |
| *VPS54* | 1,03E-16 | 8,14E-16 | 2,1 |  | 0,8 | 2,09E-03 | 4,35E-03 |
| *CELF2* | 1,06E-16 | 8,38E-16 | -4,0 |  | -5,1 | 1,61E-02 | 2,80E-02 |
| *AFMID* | 1,07E-16 | 8,44E-16 | -3,7 |  | -3,5 | 3,90E-08 | 1,60E-07 |
| *SEC11C* | 1,09E-16 | 8,58E-16 | 3,1 |  | 1,9 | 2,81E-13 | 1,84E-12 |
| *GALNT3* | 1,09E-16 | 8,65E-16 | 7,9 |  | 3,8 | 2,97E-08 | 1,24E-07 |
| *DCP1A* | 1,15E-16 | 9,06E-16 | 2,9 |  | 3,4 | 9,82E-24 | 1,27E-22 |
| *SUGCT* | 1,15E-16 | 9,10E-16 | -8,0 |  | -19,4 | 2,59E-14 | 1,83E-13 |
| *PSTPIP2* | 1,21E-16 | 9,57E-16 | 3,6 |  | 8,9 | 1,59E-17 | 1,44E-16 |
| *SUN2* | 1,22E-16 | 9,63E-16 | 3,4 |  | 0,9 | 3,65E-08 | 1,51E-07 |
| *SMG1* | 1,24E-16 | 9,76E-16 | 3,2 |  | 0,6 | 9,63E-03 | 1,76E-02 |
| *DDX20* | 1,24E-16 | 9,76E-16 | -2,4 |  | 0,1 | 3,82E-01 | 4,55E-01 |
| *VPS26A* | 1,28E-16 | 1,01E-15 | 2,6 |  | 0,1 | 5,17E-01 | 5,86E-01 |
| *SMCO4* | 1,33E-16 | 1,05E-15 | 11,3 |  | 1,9 | 1,30E-06 | 4,44E-06 |
| *B3GALNT1* | 1,36E-16 | 1,07E-15 | 3,9 |  | -2,9 | 1,70E-01 | 2,27E-01 |
| *RPL5* | 1,39E-16 | 1,09E-15 | -2,6 |  | -7,2 | 2,44E-75 | 1,61E-73 |
| *OAF* | 1,49E-16 | 1,17E-15 | 2,2 |  | 2,0 | 2,06E-05 | 6,02E-05 |
| *MTERF2* | 1,67E-16 | 1,31E-15 | -6,3 |  | -7,4 | 3,63E-08 | 1,50E-07 |
| *RPL23* | 1,77E-16 | 1,39E-15 | -2,5 |  | -5,9 | 1,68E-53 | 6,61E-52 |
| *EPB41L1* | 1,82E-16 | 1,42E-15 | -4,0 |  | -6,1 | 3,96E-13 | 2,57E-12 |
| *COL16A1* | 1,84E-16 | 1,44E-15 | 3,7 |  | -4,9 | 4,46E-31 | 8,32E-30 |
| *HEXB* | 1,89E-16 | 1,47E-15 | -2,3 |  | -2,8 | 1,71E-04 | 4,29E-04 |
| *ME1* | 1,94E-16 | 1,52E-15 | -2,8 |  | -4,7 | 9,61E-25 | 1,32E-23 |
| *RPS2* | 1,97E-16 | 1,53E-15 | -2,8 |  | -6,1 | 1,19E-43 | 3,64E-42 |
| *NR2F2* | 1,97E-16 | 1,54E-15 | -7,0 |  | 0,1 | 6,82E-01 | 7,36E-01 |
| *SMAD1* | 1,98E-16 | 1,54E-15 | 4,2 |  | 0,4 | 1,86E-01 | 2,45E-01 |
| *ZDHHC7* | 2,03E-16 | 1,58E-15 | -1,9 |  | -2,4 | 1,62E-03 | 3,45E-03 |
| *FCHO2* | 2,04E-16 | 1,59E-15 | 3,1 |  | 2,1 | 2,05E-14 | 1,45E-13 |
| *RNF207* | 2,06E-16 | 1,60E-15 | 8,8 |  | 7,2 | 1,22E-21 | 1,41E-20 |
| *ERLIN2* | 2,10E-16 | 1,63E-15 | -2,1 |  | -3,2 | 1,08E-04 | 2,81E-04 |
| *MRGPRF* | 2,16E-16 | 1,68E-15 | -4,8 |  | -3,4 | 1,02E-06 | 3,55E-06 |
| *DPP3* | 2,26E-16 | 1,75E-15 | 2,2 |  | 1,8 | 1,51E-09 | 7,22E-09 |
| *UBXN4* | 2,35E-16 | 1,82E-15 | 2,3 |  | 0,8 | 4,70E-06 | 1,50E-05 |
| *DNAJC10* | 2,46E-16 | 1,91E-15 | 2,2 |  | -2,9 | 2,03E-07 | 7,69E-07 |
| *TCEAL7* | 2,52E-16 | 1,95E-15 | -3,9 |  | -2,9 | 5,64E-03 | 1,08E-02 |
| *CARMIL1* | 2,59E-16 | 2,00E-15 | -2,8 |  | 0,4 | 5,07E-02 | 7,82E-02 |
| *SELENOS* | 2,65E-16 | 2,05E-15 | 2,6 |  | 1,0 | 7,09E-05 | 1,89E-04 |
| *PLSCR4* | 2,66E-16 | 2,05E-15 | 7,7 |  | 1,1 | 4,36E-03 | 8,52E-03 |
| *XPOT* | 2,86E-16 | 2,21E-15 | -1,8 |  | -3,6 | 5,11E-11 | 2,78E-10 |
| *NAP1L3* | 2,87E-16 | 2,22E-15 | -9,1 |  | -16,4 | 1,15E-28 | 1,91E-27 |
| *B4GAT1* | 2,94E-16 | 2,27E-15 | -3,1 |  | -2,3 | 1,15E-01 | 1,61E-01 |
| *ELMO2* | 2,97E-16 | 2,29E-15 | 2,3 |  | 8,8 | 3,87E-107 | 4,52E-105 |
| *PPT1* | 3,10E-16 | 2,39E-15 | -2,6 |  | -2,9 | 8,96E-05 | 2,35E-04 |
| *BBC3* | 3,12E-16 | 2,40E-15 | 4,9 |  | 3,2 | 3,93E-11 | 2,17E-10 |
| *ERP44* | 3,36E-16 | 2,58E-15 | 2,5 |  | 2,7 | 9,35E-24 | 1,21E-22 |
| *EIF3E* | 3,46E-16 | 2,65E-15 | -2,4 |  | -5,0 | 2,47E-23 | 3,11E-22 |
| *S100A10* | 3,60E-16 | 2,76E-15 | 2,6 |  | -2,4 | 4,37E-03 | 8,54E-03 |
| *BDKRB2* | 3,64E-16 | 2,79E-15 | 42,0 |  | 2,8 | 7,78E-11 | 4,17E-10 |
| *EMP2* | 3,74E-16 | 2,86E-15 | 4,3 |  | -2,7 | 1,25E-04 | 3,21E-04 |
| *ADPRS* | 3,78E-16 | 2,89E-15 | 3,0 |  | 5,7 | 1,57E-28 | 2,60E-27 |
| *TSPAN10* | 3,95E-16 | 3,02E-15 | -25,7 |  | -9,0 | 3,53E-11 | 1,96E-10 |
| *RBM47* | 3,97E-16 | 3,03E-15 | 11,3 |  | 13,3 | 1,88E-21 | 2,17E-20 |
| *NFE2L2* | 4,02E-16 | 3,07E-15 | 3,2 |  | -2,4 | 1,27E-01 | 1,76E-01 |
| *TYK2* | 4,26E-16 | 3,25E-15 | 2,7 |  | 1,0 | 1,89E-06 | 6,33E-06 |
| *ST13* | 4,44E-16 | 3,39E-15 | -1,9 |  | -3,4 | 2,17E-18 | 2,10E-17 |
| *C9orf72* | 4,65E-16 | 3,55E-15 | 5,8 |  | 0,7 | 7,15E-02 | 1,06E-01 |
| *PTPN12* | 4,98E-16 | 3,79E-15 | 10,6 |  | 4,3 | 2,22E-22 | 2,67E-21 |
| *SLC4A8* | 5,20E-16 | 3,96E-15 | -4,1 |  | -3,8 | 9,73E-05 | 2,54E-04 |
| *SLC25A23* | 5,23E-16 | 3,98E-15 | -3,3 |  | -2,8 | 1,56E-02 | 2,73E-02 |
| *TP53INP1* | 5,28E-16 | 4,01E-15 | -3,4 |  | -6,3 | 2,65E-09 | 1,23E-08 |
| *CCDC93* | 5,46E-16 | 4,15E-15 | 2,2 |  | 0,2 | 2,32E-01 | 2,97E-01 |
| *RAP1GAP2* | 5,51E-16 | 4,18E-15 | 3,3 |  | 1,3 | 1,20E-02 | 2,14E-02 |
| *EEF2* | 5,51E-16 | 4,18E-15 | -2,1 |  | -7,9 | 1,18E-62 | 5,74E-61 |
| *HRH1* | 5,58E-16 | 4,23E-15 | 3,4 |  | 1,5 | 1,98E-11 | 1,13E-10 |
| *L3MBTL4* | 5,85E-16 | 4,43E-15 | 342,0 |  | 6887,3 | 4,28E-23 | 5,35E-22 |
| *VWA1* | 6,47E-16 | 4,90E-15 | 8,5 |  | 0,3 | 1,13E-01 | 1,59E-01 |
| *RPS24* | 6,63E-16 | 5,02E-15 | -2,3 |  | -9,0 | 1,64E-77 | 1,13E-75 |
| *RGS3* | 6,65E-16 | 5,03E-15 | -2,6 |  | -5,3 | 4,30E-17 | 3,73E-16 |
| *SHC3* | 6,65E-16 | 5,03E-15 | -2,7 |  | -3,1 | 1,27E-02 | 2,26E-02 |
| *CC2D1B* | 6,81E-16 | 5,14E-15 | 2,1 |  | 1,2 | 6,93E-12 | 4,11E-11 |
| *GAN* | 7,01E-16 | 5,29E-15 | -2,7 |  | -4,8 | 1,49E-13 | 1,00E-12 |
| *TIAM1* | 7,40E-16 | 5,58E-15 | -5,8 |  | -15,0 | 2,16E-17 | 1,92E-16 |
| *PAICS* | 7,49E-16 | 5,64E-15 | -2,0 |  | -2,2 | 5,02E-01 | 5,72E-01 |
| *SCYL3* | 7,59E-16 | 5,72E-15 | 3,1 |  | 2,5 | 2,32E-18 | 2,23E-17 |
| *GPATCH11* | 7,80E-16 | 5,87E-15 | -2,8 |  | -11,5 | 5,12E-14 | 3,54E-13 |
| *RPL7A* | 7,99E-16 | 6,01E-15 | -2,6 |  | -6,3 | 3,50E-77 | 2,36E-75 |
| *RHOQ* | 8,03E-16 | 6,04E-15 | -2,3 |  | -3,8 | 2,43E-09 | 1,14E-08 |
| *AGO1* | 8,27E-16 | 6,21E-15 | -3,0 |  | -4,2 | 5,13E-17 | 4,42E-16 |
| *FAM83G* | 8,67E-16 | 6,51E-15 | 2,8 |  | 4,5 | 2,61E-13 | 1,72E-12 |
| *CREB3* | 8,87E-16 | 6,66E-15 | 2,4 |  | 1,8 | 4,19E-09 | 1,91E-08 |
| *GNB4* | 8,93E-16 | 6,70E-15 | 2,2 |  | 1,4 | 4,14E-14 | 2,88E-13 |
| *HCP5* | 9,06E-16 | 6,79E-15 | 1155,9 |  | 44,1 | 4,46E-66 | 2,33E-64 |
| *DCTN1* | 9,19E-16 | 6,88E-15 | -2,0 |  | -3,1 | 1,13E-07 | 4,43E-07 |
| *EDF1* | 9,29E-16 | 6,95E-15 | 2,3 |  | 1,7 | 2,09E-16 | 1,72E-15 |
| *MTSS2* | 9,74E-16 | 7,29E-15 | -4,5 |  | -4,6 | 1,81E-09 | 8,61E-09 |
| *EARS2* | 9,89E-16 | 7,39E-15 | -2,8 |  | -3,4 | 2,39E-05 | 6,92E-05 |
| *MCMBP* | 1,01E-15 | 7,56E-15 | 2,1 |  | 1,1 | 6,85E-11 | 3,68E-10 |
| *CINP* | 1,04E-15 | 7,75E-15 | -2,8 |  | -2,6 | 2,24E-02 | 3,77E-02 |
| *TNFSF10* | 1,04E-15 | 7,79E-15 | 12257,4 |  | 2204,4 | 5,78E-42 | 1,68E-40 |
| *RITA1* | 1,07E-15 | 7,96E-15 | -3,2 |  | -2,5 | 2,96E-02 | 4,83E-02 |
| *ADAM19* | 1,09E-15 | 8,09E-15 | 2,5 |  | -2,0 | 8,80E-01 | 9,05E-01 |
| *SERTAD1* | 1,15E-15 | 8,57E-15 | 3,5 |  | 3,1 | 7,01E-19 | 6,98E-18 |
| *KAT2B* | 1,19E-15 | 8,83E-15 | -5,6 |  | -2,2 | 1,78E-01 | 2,37E-01 |
| *IL15RA* | 1,22E-15 | 9,07E-15 | 546,7 |  | 2428,0 | 1,56E-217 | 6,00E-215 |
| *PCGF5* | 1,22E-15 | 9,07E-15 | 3,9 |  | 1,3 | 4,84E-06 | 1,54E-05 |
| *PTBP3* | 1,24E-15 | 9,21E-15 | 2,8 |  | 0,9 | 4,76E-04 | 1,11E-03 |
| *ZHX3* | 1,25E-15 | 9,24E-15 | -2,2 |  | -5,1 | 3,57E-24 | 4,70E-23 |
| *HDX* | 1,25E-15 | 9,29E-15 | 3,5 |  | 2,0 | 1,19E-08 | 5,18E-08 |
| *BIRC5* | 1,27E-15 | 9,42E-15 | -3,9 |  | -4,1 | 3,95E-03 | 7,79E-03 |
| *AKIP1* | 1,30E-15 | 9,63E-15 | 2,7 |  | 1,4 | 1,36E-13 | 9,15E-13 |
| *EPS8L2* | 1,30E-15 | 9,66E-15 | 5,4 |  | 3,3 | 2,29E-13 | 1,52E-12 |
| *FLYWCH2* | 1,34E-15 | 9,93E-15 | -3,3 |  | -4,4 | 4,64E-15 | 3,45E-14 |
| *BAIAP2* | 1,42E-15 | 1,05E-14 | -5,5 |  | -2,3 | 1,67E-01 | 2,24E-01 |
| *CDK2AP1* | 1,43E-15 | 1,06E-14 | -1,8 |  | -2,4 | 4,27E-02 | 6,71E-02 |
| *EMP3* | 1,44E-15 | 1,07E-14 | 2,2 |  | 1,3 | 3,31E-11 | 1,84E-10 |
| *mei-01* | 1,46E-15 | 1,08E-14 | 1077,9 |  | 326,5 | 3,50E-24 | 4,62E-23 |
| *PRKCA* | 1,48E-15 | 1,09E-14 | -3,8 |  | -14,6 | 4,78E-29 | 8,09E-28 |
| *CAP1* | 1,54E-15 | 1,14E-14 | 2,1 |  | 0,4 | 8,89E-03 | 1,63E-02 |
| *IRF2BP2* | 1,56E-15 | 1,15E-14 | -2,8 |  | -2,4 | 6,30E-03 | 1,19E-02 |
| *FAM219A* | 1,56E-15 | 1,15E-14 | 2,8 |  | 5,2 | 1,60E-20 | 1,74E-19 |
| *EYA3* | 1,65E-15 | 1,21E-14 | 2,4 |  | 0,2 | 2,76E-01 | 3,45E-01 |
| *SLC12A8* | 1,79E-15 | 1,31E-14 | 4,6 |  | -5,1 | 6,20E-06 | 1,95E-05 |
| *RAB30* | 1,79E-15 | 1,32E-14 | -3,0 |  | -3,3 | 4,58E-04 | 1,07E-03 |
| *SOX5* | 1,91E-15 | 1,41E-14 | 5,3 |  | 1,2 | 2,90E-01 | 3,59E-01 |
| *SH3D19* | 1,93E-15 | 1,42E-14 | -2,6 |  | -4,3 | 5,62E-41 | 1,56E-39 |
| *FAM120AOS* | 2,04E-15 | 1,50E-14 | 2,7 |  | 0,9 | 2,07E-10 | 1,07E-09 |
| *KCTD20* | 2,28E-15 | 1,67E-14 | -2,1 |  | -4,2 | 5,45E-09 | 2,45E-08 |
| *BCAR1* | 2,29E-15 | 1,67E-14 | 2,2 |  | 12,4 | 3,06E-27 | 4,81E-26 |
| *ZDHHC2* | 2,29E-15 | 1,67E-14 | 3,0 |  | 1,4 | 2,36E-06 | 7,79E-06 |
| *RPL4* | 2,31E-15 | 1,69E-14 | -2,3 |  | -6,7 | 2,21E-78 | 1,55E-76 |
| *ZNF766* | 2,32E-15 | 1,70E-14 | -3,1 |  | -3,1 | 9,73E-05 | 2,54E-04 |
| *STEAP1* | 2,35E-15 | 1,72E-14 | 3,8 |  | 1,6 | 1,54E-07 | 5,92E-07 |
| *PPM1D* | 2,39E-15 | 1,74E-14 | -4,1 |  | -3,4 | 1,70E-03 | 3,59E-03 |
| *FUT4* | 2,44E-15 | 1,78E-14 | 8,5 |  | 2,0 | 1,57E-08 | 6,75E-08 |
| *ABCD4* | 2,50E-15 | 1,82E-14 | 3,5 |  | 0,7 | 4,23E-04 | 9,94E-04 |
| *LZIC* | 2,51E-15 | 1,83E-14 | -2,5 |  | -3,1 | 3,70E-08 | 1,52E-07 |
| *ADGRA3* | 2,54E-15 | 1,85E-14 | -3,1 |  | -3,6 | 3,68E-07 | 1,35E-06 |
| *SLC26A2* | 2,56E-15 | 1,86E-14 | -2,6 |  | -10,9 | 1,15E-36 | 2,74E-35 |
| *CDKN1A* | 2,57E-15 | 1,87E-14 | -2,6 |  | -2,4 | 2,54E-03 | 5,20E-03 |
| *CTIF* | 2,60E-15 | 1,89E-14 | -2,4 |  | -2,6 | 1,68E-05 | 4,96E-05 |
| *ADAM8* | 2,60E-15 | 1,89E-14 | 23,8 |  | 135,1 | 1,40E-33 | 2,96E-32 |
| *AFAP1L1* | 2,61E-15 | 1,89E-14 | 2,9 |  | 7,1 | 1,62E-07 | 6,22E-07 |
| *PDLIM2* | 2,73E-15 | 1,99E-14 | -3,8 |  | -3,5 | 1,05E-10 | 5,58E-10 |
| *TGFBR2* | 2,77E-15 | 2,01E-14 | -2,3 |  | -2,7 | 8,08E-03 | 1,50E-02 |
| *BFSP1* | 2,81E-15 | 2,04E-14 | -5,4 |  | -15,3 | 4,98E-04 | 1,15E-03 |
| *CTSB* | 2,81E-15 | 2,04E-14 | 2,5 |  | 0,4 | 7,06E-04 | 1,59E-03 |
| *WDR47* | 2,83E-15 | 2,05E-14 | 2,7 |  | 1,2 | 2,88E-07 | 1,07E-06 |
| *ARRDC1* | 2,94E-15 | 2,13E-14 | 5,2 |  | 5,1 | 2,48E-16 | 2,04E-15 |
| *PXK* | 2,97E-15 | 2,15E-14 | -3,2 |  | -4,6 | 1,68E-34 | 3,68E-33 |
| *RORA* | 2,98E-15 | 2,16E-14 | 3,8 |  | 2,9 | 7,08E-21 | 7,89E-20 |
| *PPA2* | 2,98E-15 | 2,16E-14 | -2,7 |  | -4,4 | 4,62E-12 | 2,78E-11 |
| *GSTO1* | 3,14E-15 | 2,26E-14 | 2,3 |  | 1,2 | 6,12E-05 | 1,65E-04 |
| *PDE4D* | 3,28E-15 | 2,37E-14 | 5,3 |  | 28,8 | 7,19E-07 | 2,55E-06 |
| *ENAH* | 3,32E-15 | 2,39E-14 | -2,6 |  | -4,2 | 1,02E-09 | 4,97E-09 |
| *OSMR* | 3,37E-15 | 2,42E-14 | 2,9 |  | 1,4 | 6,42E-21 | 7,17E-20 |
| *PTPRS* | 3,47E-15 | 2,50E-14 | -3,7 |  | -3,9 | 1,34E-16 | 1,12E-15 |
| *RPS5* | 3,53E-15 | 2,54E-14 | -2,2 |  | -3,9 | 3,93E-18 | 3,70E-17 |
| *GRINA* | 3,70E-15 | 2,66E-14 | 2,7 |  | 1,4 | 3,65E-16 | 2,95E-15 |
| *BVES* | 3,73E-15 | 2,68E-14 | 2,7 |  | 1,3 | 8,32E-06 | 2,58E-05 |
| *CHRNA1* | 3,77E-15 | 2,70E-14 | 6,0 |  | 1166,8 | 2,86E-23 | 3,61E-22 |
| *RPL11* | 3,82E-15 | 2,74E-14 | -2,4 |  | -5,4 | 1,03E-47 | 3,60E-46 |
| *HILPDA* | 3,85E-15 | 2,76E-14 | 5,2 |  | 4,6 | 1,88E-05 | 5,50E-05 |
| *PLGRKT* | 3,88E-15 | 2,78E-14 | 4,4 |  | 1,4 | 1,68E-04 | 4,22E-04 |
| *PCDH18* | 3,94E-15 | 2,82E-14 | -2,8 |  | -3,1 | 2,68E-11 | 1,50E-10 |
| *UBR2* | 3,95E-15 | 2,82E-14 | 2,0 |  | 1,3 | 2,86E-10 | 1,46E-09 |
| *MFSD14B* | 4,09E-15 | 2,92E-14 | 2,3 |  | 1,7 | 2,26E-30 | 4,02E-29 |
| *HSD3B7* | 4,14E-15 | 2,96E-14 | 5,4 |  | 2,1 | 1,02E-15 | 7,92E-15 |
| *NISCH* | 4,15E-15 | 2,96E-14 | -2,4 |  | -2,8 | 8,38E-06 | 2,59E-05 |
| *SPHK2* | 4,21E-15 | 3,01E-14 | -3,6 |  | -2,4 | 3,89E-02 | 6,17E-02 |
| *DOP1B* | 4,23E-15 | 3,02E-14 | -3,8 |  | -2,2 | 2,37E-01 | 3,02E-01 |
| *RIN2* | 4,39E-15 | 3,13E-14 | -2,3 |  | -2,5 | 1,75E-03 | 3,70E-03 |
| *MRPL30* | 4,41E-15 | 3,14E-14 | -2,2 |  | -2,8 | 2,45E-04 | 5,95E-04 |
| *KIFAP3* | 4,42E-15 | 3,15E-14 | 2,4 |  | 1,1 | 5,72E-12 | 3,42E-11 |
| *SLC7A14* | 4,52E-15 | 3,21E-14 | -10,6 |  | -102,4 | 1,43E-10 | 7,50E-10 |
| *SERINC5* | 4,75E-15 | 3,38E-14 | -4,4 |  | -19,8 | 2,27E-53 | 8,85E-52 |
| *NAMPTP1* | 5,28E-15 | 3,75E-14 | 51,0 |  | 75,8 | 4,47E-21 | 5,04E-20 |
| *COL4A1* | 5,54E-15 | 3,93E-14 | 6,5 |  | 0,3 | 1,84E-01 | 2,44E-01 |
| *TCTN3* | 5,65E-15 | 4,01E-14 | -2,2 |  | -2,5 | 7,05E-04 | 1,59E-03 |
| *WTIP* | 5,86E-15 | 4,16E-14 | -3,1 |  | -4,5 | 9,09E-13 | 5,75E-12 |
| *TMUB2* | 6,31E-15 | 4,47E-14 | 2,5 |  | 1,4 | 1,29E-09 | 6,23E-09 |
| *ARHGEF9* | 6,34E-15 | 4,49E-14 | -4,6 |  | -7,1 | 2,79E-21 | 3,17E-20 |
| *C14orf132* | 6,37E-15 | 4,51E-14 | -2,8 |  | -6,8 | 1,64E-34 | 3,60E-33 |
| *TMEM14A* | 6,38E-15 | 4,52E-14 | -2,9 |  | -3,4 | 1,62E-13 | 1,08E-12 |
| *APOBEC3F* | 6,39E-15 | 4,52E-14 | 7,7 |  | 2,5 | 2,57E-06 | 8,44E-06 |
| *NDEL1* | 6,48E-15 | 4,58E-14 | 2,1 |  | 0,2 | 7,40E-02 | 1,09E-01 |
| *GOLIM4* | 6,52E-15 | 4,60E-14 | -2,3 |  | -4,0 | 6,06E-18 | 5,62E-17 |
| *NPM1* | 6,60E-15 | 4,66E-14 | -2,3 |  | -6,0 | 4,07E-57 | 1,73E-55 |
| *TRAF3* | 6,75E-15 | 4,76E-14 | 2,1 |  | 0,5 | 3,03E-02 | 4,94E-02 |
| *RIN1* | 6,98E-15 | 4,92E-14 | -4,2 |  | -3,9 | 9,59E-09 | 4,20E-08 |
| *STXBP4* | 7,00E-15 | 4,93E-14 | -3,7 |  | -13,0 | 2,18E-17 | 1,93E-16 |
| *UBE2D3* | 7,16E-15 | 5,04E-14 | 1,7 |  | 1,6 | 1,89E-14 | 1,35E-13 |
| *MID2* | 7,69E-15 | 5,41E-14 | 5,2 |  | 2,5 | 7,47E-17 | 6,38E-16 |
| *PSMA6* | 7,75E-15 | 5,45E-14 | 5,1 |  | 3,0 | 3,41E-07 | 1,26E-06 |
| *ZNF24* | 7,90E-15 | 5,55E-14 | 2,3 |  | 0,6 | 4,67E-04 | 1,09E-03 |
| *CTDSP2* | 8,46E-15 | 5,94E-14 | 2,4 |  | 0,4 | 2,58E-02 | 4,28E-02 |
| *ASXL1* | 8,88E-15 | 6,23E-14 | 2,7 |  | 0,5 | 1,12E-03 | 2,45E-03 |
| *TRADD* | 8,92E-15 | 6,25E-14 | 3,0 |  | 4,2 | 1,17E-29 | 2,02E-28 |
| *PRKCD* | 9,01E-15 | 6,31E-14 | 7,9 |  | 17,1 | 1,58E-19 | 1,63E-18 |
| *MFSD12* | 9,15E-15 | 6,41E-14 | 2,1 |  | 1,4 | 5,35E-11 | 2,90E-10 |
| *USP46* | 9,57E-15 | 6,69E-14 | -2,3 |  | -4,5 | 4,27E-11 | 2,35E-10 |
| *ERI3* | 9,84E-15 | 6,88E-14 | -2,6 |  | -2,7 | 8,25E-04 | 1,84E-03 |
| *FAM91A1* | 9,88E-15 | 6,90E-14 | 2,3 |  | 0,2 | 3,11E-01 | 3,83E-01 |
| *ZNF274* | 1,02E-14 | 7,13E-14 | 2,9 |  | 1,7 | 5,19E-12 | 3,11E-11 |
| *BOC* | 1,05E-14 | 7,32E-14 | 6,7 |  | 2,1 | 4,44E-09 | 2,02E-08 |
| *ACP2* | 1,05E-14 | 7,32E-14 | 2,3 |  | 10,1 | 7,32E-26 | 1,07E-24 |
| *SAMD14* | 1,06E-14 | 7,38E-14 | 3,7 |  | 0,8 | 2,95E-02 | 4,82E-02 |
| *MAPK11* | 1,09E-14 | 7,60E-14 | 3,1 |  | 1,9 | 2,78E-08 | 1,16E-07 |
| *NEK6* | 1,10E-14 | 7,67E-14 | -2,4 |  | -3,7 | 3,22E-21 | 3,65E-20 |
| *SEC22B4P* | 1,10E-14 | 7,67E-14 | 6,1 |  | 4,3 | 1,83E-09 | 8,67E-09 |
| *DNMBP* | 1,14E-14 | 7,93E-14 | -3,3 |  | -2,4 | 1,89E-01 | 2,49E-01 |
| *ZNF276* | 1,15E-14 | 7,96E-14 | 3,2 |  | 0,9 | 2,98E-04 | 7,17E-04 |
| *ARF3* | 1,17E-14 | 8,09E-14 | 1,9 |  | 1,2 | 1,06E-09 | 5,14E-09 |
| *BMP1* | 1,19E-14 | 8,24E-14 | 2,7 |  | -2,5 | 6,97E-04 | 1,57E-03 |
| *ZNF850* | 1,20E-14 | 8,28E-14 | -10,8 |  | -19,0 | 3,87E-07 | 1,42E-06 |
| *RAP2C* | 1,20E-14 | 8,31E-14 | 2,6 |  | 3,3 | 1,03E-14 | 7,51E-14 |
| *FKBP7* | 1,20E-14 | 8,34E-14 | -2,9 |  | -6,3 | 4,11E-26 | 6,09E-25 |
| *FSTL3* | 1,28E-14 | 8,85E-14 | 17,1 |  | 4,6 | 4,12E-04 | 9,69E-04 |
| *ATP5MC1* | 1,28E-14 | 8,86E-14 | -3,2 |  | -4,1 | 1,04E-09 | 5,04E-09 |
| *BTBD11* | 1,34E-14 | 9,25E-14 | -3,5 |  | 3,5 | 9,00E-11 | 4,81E-10 |
| *RMC1* | 1,34E-14 | 9,25E-14 | 2,7 |  | 2,2 | 3,18E-12 | 1,93E-11 |
| *SCN2A* | 1,34E-14 | 9,27E-14 | -4,3 |  | -10,6 | 1,42E-14 | 1,02E-13 |
| *ANKRD12* | 1,37E-14 | 9,47E-14 | 3,2 |  | 0,1 | 6,26E-01 | 6,85E-01 |
| *C2* | 1,40E-14 | 9,67E-14 | 77,7 |  | 42,5 | 2,72E-05 | 7,81E-05 |
| *ARID5A* | 1,41E-14 | 9,74E-14 | 4,9 |  | 23,8 | 9,59E-43 | 2,87E-41 |
| *RPS23* | 1,42E-14 | 9,76E-14 | -2,5 |  | -6,6 | 1,57E-56 | 6,61E-55 |
| *RHOG* | 1,42E-14 | 9,80E-14 | 3,0 |  | 2,4 | 2,63E-15 | 1,98E-14 |
| *MAP3K11* | 1,42E-14 | 9,80E-14 | 2,1 |  | 1,7 | 3,11E-14 | 2,19E-13 |
| *CDK4* | 1,44E-14 | 9,90E-14 | -2,4 |  | -4,0 | 1,48E-30 | 2,67E-29 |
| *MIR4435-2HG* | 1,45E-14 | 9,97E-14 | 1,9 |  | -2,9 | 1,92E-07 | 7,29E-07 |
| *MTFR1* | 1,50E-14 | 1,03E-13 | -2,7 |  | -3,7 | 1,81E-07 | 6,92E-07 |
| *MTCH2* | 1,56E-14 | 1,07E-13 | -2,0 |  | -2,7 | 1,51E-04 | 3,82E-04 |
| *TCEAL3* | 1,57E-14 | 1,07E-13 | -3,6 |  | -3,1 | 7,35E-05 | 1,95E-04 |
| *RPL22* | 1,58E-14 | 1,08E-13 | -2,7 |  | -6,9 | 2,21E-25 | 3,17E-24 |
| *CEP250* | 1,58E-14 | 1,08E-13 | -2,5 |  | -2,6 | 2,84E-03 | 5,76E-03 |
| *OLFML2A* | 1,62E-14 | 1,11E-13 | -20,8 |  | -114,8 | 3,07E-40 | 8,27E-39 |
| *HLTF* | 1,65E-14 | 1,13E-13 | -2,7 |  | -3,9 | 1,91E-12 | 1,18E-11 |
| *TAF9B* | 1,73E-14 | 1,18E-13 | -6,5 |  | -91,0 | 3,57E-26 | 5,29E-25 |
| *FGFR1OP2* | 1,85E-14 | 1,26E-13 | 2,9 |  | -2,2 | 2,97E-01 | 3,67E-01 |
| *TEP1* | 1,91E-14 | 1,30E-13 | 2,7 |  | 0,5 | 2,40E-03 | 4,94E-03 |
| *FAH* | 1,92E-14 | 1,31E-13 | -2,8 |  | -4,3 | 3,12E-15 | 2,34E-14 |
| *ZFYVE9* | 1,93E-14 | 1,31E-13 | -2,2 |  | -2,6 | 4,62E-04 | 1,08E-03 |
| *SNAPC1* | 1,94E-14 | 1,32E-13 | 3,2 |  | 1,0 | 4,36E-05 | 1,21E-04 |
| *RAB2B* | 1,95E-14 | 1,33E-13 | 2,4 |  | 2,1 | 3,49E-16 | 2,82E-15 |
| *SLFN12* | 1,96E-14 | 1,33E-13 | 2,3 |  | 3,9 | 3,92E-25 | 5,52E-24 |
| *HECA* | 2,05E-14 | 1,39E-13 | 2,7 |  | -3,1 | 1,19E-01 | 1,66E-01 |
| *PARP3* | 2,07E-14 | 1,40E-13 | 3,8 |  | 2,8 | 2,74E-29 | 4,69E-28 |
| *FNBP1L* | 2,11E-14 | 1,43E-13 | -2,5 |  | -6,3 | 4,81E-18 | 4,50E-17 |
| *H4C5* | 2,32E-14 | 1,57E-13 | -3,9 |  | -11,2 | 3,51E-40 | 9,40E-39 |
| *RANGAP1* | 2,37E-14 | 1,61E-13 | 1,9 |  | 10,3 | 2,31E-102 | 2,53E-100 |
| *MRPL42* | 2,54E-14 | 1,72E-13 | -2,4 |  | -2,7 | 6,94E-03 | 1,30E-02 |
| *SLC4A4* | 2,59E-14 | 1,75E-13 | -2,8 |  | 2,2 | 3,53E-06 | 1,14E-05 |
| *TACC2* | 2,63E-14 | 1,78E-13 | -6,7 |  | -3,3 | 5,85E-03 | 1,12E-02 |
| *CAB39* | 2,68E-14 | 1,81E-13 | 2,8 |  | 0,2 | 1,49E-01 | 2,03E-01 |
| *EIF3L* | 2,68E-14 | 1,81E-13 | -2,6 |  | -7,7 | 9,28E-58 | 3,99E-56 |
| *CMTM4* | 2,77E-14 | 1,87E-13 | -5,2 |  | -62,2 | 1,60E-44 | 5,03E-43 |
| *DHRSX* | 2,79E-14 | 1,88E-13 | 2,3 |  | 1,6 | 5,28E-10 | 2,64E-09 |
| *PPP1R15B* | 2,80E-14 | 1,89E-13 | 2,6 |  | 0,5 | 1,58E-03 | 3,36E-03 |
| *SH3BP4* | 2,85E-14 | 1,92E-13 | -2,4 |  | 0,0 | 7,73E-01 | 8,16E-01 |
| *C21orf91* | 2,90E-14 | 1,95E-13 | 4,4 |  | 0,8 | 3,95E-03 | 7,79E-03 |
| *GPSM3* | 2,91E-14 | 1,96E-13 | 5,3 |  | 56,6 | 4,25E-26 | 6,28E-25 |
| *SACS* | 3,00E-14 | 2,02E-13 | -2,5 |  | -9,9 | 4,20E-17 | 3,65E-16 |
| *IFI27L1* | 3,06E-14 | 2,06E-13 | 4,4 |  | 4,1 | 1,10E-26 | 1,68E-25 |
| *IFT52* | 3,11E-14 | 2,09E-13 | -2,3 |  | -3,2 | 1,83E-10 | 9,55E-10 |
| *NCK2* | 3,26E-14 | 2,19E-13 | 2,4 |  | 1,3 | 3,58E-05 | 1,01E-04 |
| *MPV17* | 3,26E-14 | 2,19E-13 | -2,4 |  | -3,4 | 7,06E-09 | 3,13E-08 |
| *IFNGR1* | 3,31E-14 | 2,22E-13 | 2,9 |  | 0,8 | 5,27E-05 | 1,44E-04 |
| *RPL10A* | 3,31E-14 | 2,22E-13 | -2,4 |  | -8,7 | 1,66E-89 | 1,33E-87 |
| *LMCD1* | 3,53E-14 | 2,36E-13 | -9,9 |  | -6,9 | 1,07E-20 | 1,18E-19 |
| *ADGRB2* | 3,61E-14 | 2,41E-13 | -4,3 |  | -3,5 | 7,69E-04 | 1,72E-03 |
| *HLA-DPB1* | 3,87E-14 | 2,59E-13 | 4,4 |  | 5,5 | 5,38E-24 | 7,04E-23 |
| *GUK1* | 4,03E-14 | 2,69E-13 | 2,3 |  | 0,9 | 1,24E-04 | 3,17E-04 |
| *RPL8* | 4,03E-14 | 2,69E-13 | -2,2 |  | -4,4 | 6,16E-29 | 1,04E-27 |
| *SLC30A4* | 4,05E-14 | 2,70E-13 | -2,7 |  | -2,3 | 6,60E-02 | 9,90E-02 |
| *ST6GAL1* | 4,10E-14 | 2,74E-13 | 4,6 |  | 0,0 | 9,62E-01 | 9,71E-01 |
| *PTPN11* | 4,19E-14 | 2,79E-13 | -1,9 |  | -5,4 | 2,01E-47 | 6,99E-46 |
| *ELL2* | 4,19E-14 | 2,79E-13 | 2,1 |  | 2,2 | 6,38E-26 | 9,32E-25 |
| *IRAK3* | 4,22E-14 | 2,81E-13 | 342,0 |  | 6,3 | 5,55E-09 | 2,49E-08 |
| *THEMIS2* | 4,39E-14 | 2,92E-13 | 12,1 |  | 19,1 | 1,45E-31 | 2,77E-30 |
| *TMEM102* | 4,44E-14 | 2,95E-13 | 21,4 |  | 7,4 | 3,95E-09 | 1,80E-08 |
| *SLIT3* | 4,59E-14 | 3,05E-13 | -7,5 |  | -7,4 | 5,08E-22 | 5,99E-21 |
| *POLR1F* | 4,76E-14 | 3,16E-13 | -2,3 |  | -2,1 | 6,33E-01 | 6,92E-01 |
| *DCTN5* | 4,92E-14 | 3,27E-13 | -2,1 |  | -2,5 | 5,82E-04 | 1,33E-03 |
| *TLE5* | 4,99E-14 | 3,31E-13 | 1,7 |  | 1,2 | 1,47E-10 | 7,70E-10 |
| *CAT* | 4,99E-14 | 3,31E-13 | -2,9 |  | -5,0 | 1,28E-23 | 1,64E-22 |
| *AFAP1* | 5,07E-14 | 3,36E-13 | -2,2 |  | 0,7 | 6,60E-03 | 1,25E-02 |
| *RIPK3* | 5,13E-14 | 3,40E-13 | 1224,6 |  | 15,7 | 1,91E-21 | 2,19E-20 |
| *RASSF9* | 5,33E-14 | 3,53E-13 | -1638,8 |  | -96,9 | 8,35E-05 | 2,20E-04 |
| *RABEP1* | 5,40E-14 | 3,57E-13 | -1,9 |  | -3,6 | 1,21E-12 | 7,61E-12 |
| *CD151* | 5,81E-14 | 3,84E-13 | -1,8 |  | -2,2 | 1,45E-01 | 1,98E-01 |
| *ANKRD10* | 5,86E-14 | 3,87E-13 | 2,6 |  | -3,9 | 2,93E-02 | 4,79E-02 |
| *GNB1* | 5,93E-14 | 3,92E-13 | 1,9 |  | 0,8 | 6,17E-08 | 2,49E-07 |
| *RPGR* | 5,94E-14 | 3,92E-13 | 4,7 |  | 0,7 | 4,24E-02 | 6,67E-02 |
| *RPL37A* | 6,00E-14 | 3,96E-13 | -2,4 |  | -5,0 | 1,88E-30 | 3,36E-29 |
| *ZSWIM4* | 6,36E-14 | 4,19E-13 | 7,8 |  | 5,0 | 8,70E-15 | 6,35E-14 |
| *HSPA4* | 6,51E-14 | 4,29E-13 | -1,6 |  | -2,4 | 3,45E-04 | 8,23E-04 |
| *TBC1D4* | 6,55E-14 | 4,31E-13 | -2,5 |  | -4,1 | 3,73E-17 | 3,25E-16 |
| *SLC50A1* | 6,65E-14 | 4,37E-13 | 2,8 |  | 2,1 | 5,29E-10 | 2,65E-09 |
| *MMAA* | 6,71E-14 | 4,41E-13 | 3,6 |  | 2,0 | 2,53E-11 | 1,42E-10 |
| *AP5Z1* | 7,01E-14 | 4,61E-13 | 2,4 |  | 0,7 | 5,41E-04 | 1,24E-03 |
| *KCNG1* | 7,10E-14 | 4,66E-13 | 2,8 |  | 2,5 | 4,36E-02 | 6,83E-02 |
| *COL13A1* | 7,26E-14 | 4,76E-13 | -2,7 |  | -2,4 | 3,92E-01 | 4,64E-01 |
| *RPL9* | 7,33E-14 | 4,81E-13 | -2,5 |  | -5,1 | 4,11E-25 | 5,79E-24 |
| *BCKDHB* | 7,36E-14 | 4,82E-13 | -5,6 |  | -4,6 | 4,67E-08 | 1,91E-07 |
| *FAXDC2* | 7,47E-14 | 4,89E-13 | -19,6 |  | -10,3 | 1,20E-13 | 8,08E-13 |
| *RGS4* | 7,63E-14 | 5,00E-13 | -13,0 |  | -63,3 | 4,91E-46 | 1,63E-44 |
| *FGD6* | 7,66E-14 | 5,02E-13 | 4,2 |  | 0,5 | 3,41E-02 | 5,49E-02 |
| *XPNPEP1* | 7,79E-14 | 5,10E-13 | 2,1 |  | 1,6 | 8,56E-10 | 4,20E-09 |
| *TMEM164* | 7,86E-14 | 5,14E-13 | -3,0 |  | -2,3 | 2,55E-01 | 3,22E-01 |
| *NFE2L1* | 8,00E-14 | 5,23E-13 | 2,2 |  | 2,4 | 2,12E-25 | 3,04E-24 |
| *SPOCK1* | 8,01E-14 | 5,23E-13 | -2,8 |  | -2,9 | 4,36E-04 | 1,02E-03 |
| *ENSG00000285106* | 8,22E-14 | 5,37E-13 | 6,4 |  | -2,4 | 1,64E-01 | 2,20E-01 |
| *DNAJB1* | 8,36E-14 | 5,45E-13 | -2,1 |  | 0,0 | 9,20E-01 | 9,39E-01 |
| *RPL29* | 8,44E-14 | 5,51E-13 | -2,3 |  | -4,3 | 1,55E-23 | 1,97E-22 |
| *LCTL* | 8,77E-14 | 5,72E-13 | -4,4 |  | 6,0 | 2,05E-07 | 7,75E-07 |
| *RAB3B* | 8,95E-14 | 5,83E-13 | -2,4 |  | -5,5 | 8,65E-18 | 7,94E-17 |
| *IDI1* | 9,25E-14 | 6,02E-13 | -2,4 |  | -2,1 | 6,85E-01 | 7,39E-01 |
| *MYH15* | 9,33E-14 | 6,07E-13 | -12,6 |  | -18,0 | 6,59E-09 | 2,94E-08 |
| *IGSF8* | 9,79E-14 | 6,37E-13 | 4,0 |  | 6,9 | 4,55E-38 | 1,14E-36 |
| *TUFM* | 1,01E-13 | 6,56E-13 | -2,0 |  | -2,8 | 1,15E-07 | 4,49E-07 |
| *GRPEL1* | 1,03E-13 | 6,69E-13 | 2,6 |  | 2,4 | 5,79E-15 | 4,28E-14 |
| *TOR4A* | 1,04E-13 | 6,74E-13 | 3,8 |  | 5,3 | 1,19E-31 | 2,28E-30 |
| *TSC22D4* | 1,04E-13 | 6,77E-13 | 2,6 |  | 1,2 | 2,07E-08 | 8,78E-08 |
| *TSPAN6* | 1,08E-13 | 7,01E-13 | -2,8 |  | -2,9 | 1,34E-05 | 4,01E-05 |
| *SLC4A3* | 1,11E-13 | 7,20E-13 | -4,2 |  | -13,3 | 6,33E-10 | 3,14E-09 |
| *CDC42EP3* | 1,12E-13 | 7,25E-13 | -2,1 |  | -3,3 | 2,03E-05 | 5,94E-05 |
| *WASHC2A* | 1,18E-13 | 7,63E-13 | 1,9 |  | 2,2 | 7,90E-21 | 8,78E-20 |
| *RPL34* | 1,23E-13 | 7,93E-13 | -2,4 |  | -4,8 | 2,05E-35 | 4,70E-34 |
| *DIMT1* | 1,24E-13 | 8,00E-13 | -2,4 |  | -3,7 | 3,33E-09 | 1,53E-08 |
| *HDAC6* | 1,25E-13 | 8,06E-13 | 2,4 |  | 0,5 | 2,17E-02 | 3,67E-02 |
| *PARP11* | 1,26E-13 | 8,15E-13 | 7,8 |  | 1,6 | 5,87E-07 | 2,10E-06 |
| *ULBP2* | 1,26E-13 | 8,16E-13 | 3,8 |  | 1,2 | 2,00E-03 | 4,19E-03 |
| *DIPK2A* | 1,27E-13 | 8,20E-13 | 3,7 |  | 4,2 | 9,47E-10 | 4,63E-09 |
| *LMO2* | 1,28E-13 | 8,22E-13 | 126,5 |  | 5,4 | 4,93E-08 | 2,01E-07 |
| *IPO7* | 1,30E-13 | 8,36E-13 | 1,8 |  | -2,4 | 7,53E-02 | 1,11E-01 |
| *TMEM204* | 1,32E-13 | 8,51E-13 | 7,6 |  | 0,1 | 6,42E-01 | 7,00E-01 |
| *LINC01138* | 1,35E-13 | 8,65E-13 | 22,0 |  | 5,9 | 4,28E-05 | 1,19E-04 |
| *FILIP1L* | 1,35E-13 | 8,67E-13 | 2,1 |  | 30,3 | 1,45E-63 | 7,28E-62 |
| *CNPPD1* | 1,38E-13 | 8,85E-13 | 2,8 |  | 1,3 | 2,31E-08 | 9,77E-08 |
| *WWC1* | 1,41E-13 | 9,09E-13 | 2,6 |  | 42,0 | 8,76E-66 | 4,56E-64 |
| *CYP27A1* | 1,45E-13 | 9,32E-13 | 9,0 |  | 0,5 | 3,12E-01 | 3,83E-01 |
| *PBX1* | 1,54E-13 | 9,90E-13 | -16,1 |  | -5,8 | 1,42E-15 | 1,09E-14 |
| *ARHGEF2* | 1,56E-13 | 9,98E-13 | 2,3 |  | 1,9 | 1,05E-19 | 1,09E-18 |
| *MRPL3* | 1,57E-13 | 1,01E-12 | -2,2 |  | -4,3 | 3,51E-12 | 2,13E-11 |
| *LRRC8E* | 1,59E-13 | 1,02E-12 | -4,7 |  | -2,4 | 6,12E-02 | 9,25E-02 |
| *NUP50-DT* | 1,60E-13 | 1,03E-12 | 3,0 |  | 0,7 | 1,02E-02 | 1,85E-02 |
| *SLC8A1-AS1* | 1,65E-13 | 1,05E-12 | 15,2 |  | 126,7 | 1,33E-20 | 1,45E-19 |
| *ANAPC13* | 1,66E-13 | 1,06E-12 | -2,0 |  | -2,1 | 4,52E-01 | 5,23E-01 |
| *CREBRF* | 1,67E-13 | 1,07E-12 | 2,7 |  | 2,5 | 3,33E-05 | 9,40E-05 |
| *SKIL* | 1,69E-13 | 1,08E-12 | 3,3 |  | -2,1 | 4,39E-01 | 5,11E-01 |
| *ATP5PB* | 1,71E-13 | 1,09E-12 | -2,1 |  | -3,0 | 7,74E-09 | 3,42E-08 |
| *JAK3* | 1,75E-13 | 1,12E-12 | 276,9 |  | 52,8 | 8,01E-46 | 2,65E-44 |
| *SLC35G2* | 1,75E-13 | 1,12E-12 | 2,9 |  | 2,5 | 2,98E-11 | 1,67E-10 |
| *RPS14* | 1,78E-13 | 1,13E-12 | -2,2 |  | -3,7 | 6,37E-14 | 4,37E-13 |
| *FAM131B* | 1,80E-13 | 1,15E-12 | -4,2 |  | -17,2 | 1,29E-11 | 7,49E-11 |
| *ARHGAP35* | 1,81E-13 | 1,15E-12 | -2,3 |  | -3,1 | 9,12E-08 | 3,61E-07 |
| *PNP* | 1,93E-13 | 1,23E-12 | 2,6 |  | 0,6 | 3,94E-02 | 6,24E-02 |
| *FGD4* | 1,96E-13 | 1,25E-12 | -30,4 |  | -19,0 | 2,70E-27 | 4,27E-26 |
| *SYNJ2BP* | 2,04E-13 | 1,29E-12 | -2,4 |  | -5,4 | 4,83E-14 | 3,34E-13 |
| *FRG1BP* | 2,08E-13 | 1,32E-12 | 7,4 |  | 0,2 | 5,41E-01 | 6,08E-01 |
| *ZNF542P* | 2,11E-13 | 1,34E-12 | 2,9 |  | -2,1 | 5,02E-01 | 5,72E-01 |
| *GSR* | 2,12E-13 | 1,35E-12 | -2,7 |  | 0,1 | 5,02E-01 | 5,73E-01 |
| *KIFBP* | 2,14E-13 | 1,36E-12 | 2,1 |  | 0,8 | 7,28E-08 | 2,91E-07 |
| *DBT* | 2,16E-13 | 1,37E-12 | -2,7 |  | -3,0 | 4,89E-04 | 1,13E-03 |
| *PPP2R2A* | 2,18E-13 | 1,38E-12 | 2,9 |  | 4,9 | 9,54E-42 | 2,73E-40 |
| *BATF3* | 2,23E-13 | 1,41E-12 | 77,7 |  | 47,1 | 1,13E-17 | 1,03E-16 |
| *FKBP14* | 2,62E-13 | 1,66E-12 | -2,1 |  | -6,8 | 3,67E-34 | 7,97E-33 |
| *TGFA* | 2,63E-13 | 1,66E-12 | 2295,1 |  | 37,8 | 1,33E-31 | 2,55E-30 |
| *FAM171A2* | 2,64E-13 | 1,67E-12 | -7,5 |  | -59,2 | 6,73E-12 | 3,99E-11 |
| *JMJD6* | 2,71E-13 | 1,71E-12 | 2,5 |  | 1,5 | 9,88E-08 | 3,88E-07 |
| *NPTXR* | 2,73E-13 | 1,72E-12 | -9,7 |  | -64,0 | 4,20E-10 | 2,12E-09 |
| *WWTR1* | 2,86E-13 | 1,81E-12 | -3,2 |  | -3,2 | 4,57E-04 | 1,07E-03 |
| *CSGALNACT2* | 2,89E-13 | 1,82E-12 | 3,7 |  | -2,3 | 2,02E-01 | 2,64E-01 |
| *SLC1A4* | 2,92E-13 | 1,84E-12 | 7,0 |  | 1,1 | 1,04E-03 | 2,28E-03 |
| *CEBPZOS* | 3,19E-13 | 2,01E-12 | -2,5 |  | -3,2 | 1,26E-05 | 3,78E-05 |
| *PPFIA4* | 3,27E-13 | 2,06E-12 | 6,9 |  | 2,2 | 3,76E-09 | 1,72E-08 |
| *PRKG1* | 3,30E-13 | 2,08E-12 | -2,9 |  | -6,6 | 1,18E-15 | 9,13E-15 |
| *NSMCE4A* | 3,32E-13 | 2,09E-12 | 2,9 |  | 1,2 | 4,57E-05 | 1,26E-04 |
| *TSC22D2* | 3,36E-13 | 2,11E-12 | -3,0 |  | -2,2 | 2,97E-01 | 3,68E-01 |
| *LRIF1* | 3,38E-13 | 2,12E-12 | 2,5 |  | 2,1 | 7,48E-17 | 6,38E-16 |
| *FGFRL1* | 3,40E-13 | 2,14E-12 | -2,4 |  | -3,5 | 8,29E-06 | 2,57E-05 |
| *PSENEN* | 3,47E-13 | 2,18E-12 | 3,4 |  | 0,7 | 2,92E-02 | 4,77E-02 |
| *MCFD2* | 3,54E-13 | 2,22E-12 | -1,8 |  | -2,7 | 2,83E-02 | 4,66E-02 |
| *SLC25A6* | 3,62E-13 | 2,27E-12 | -2,2 |  | -5,6 | 2,38E-50 | 8,67E-49 |
| *MDH1* | 3,66E-13 | 2,29E-12 | -1,9 |  | -2,1 | 3,20E-01 | 3,91E-01 |
| *ETV6* | 3,70E-13 | 2,32E-12 | 3,7 |  | 1,9 | 6,95E-12 | 4,12E-11 |
| *MYO18A* | 3,86E-13 | 2,42E-12 | -2,9 |  | -2,0 | 8,49E-01 | 8,79E-01 |
| *BBX* | 3,87E-13 | 2,42E-12 | 2,2 |  | 0,9 | 2,16E-06 | 7,17E-06 |
| *PGAP2* | 3,91E-13 | 2,45E-12 | -4,6 |  | -3,4 | 1,64E-04 | 4,13E-04 |
| *DBN1* | 3,96E-13 | 2,47E-12 | -1,8 |  | -2,3 | 7,38E-03 | 1,38E-02 |
| *HMG20B* | 4,01E-13 | 2,50E-12 | -2,2 |  | -3,2 | 9,48E-07 | 3,31E-06 |
| *CYB5R4* | 4,08E-13 | 2,54E-12 | 2,4 |  | 0,5 | 1,21E-01 | 1,69E-01 |
| *TECPR1* | 4,11E-13 | 2,56E-12 | 2,4 |  | 0,6 | 7,45E-04 | 1,67E-03 |
| *CTU2* | 4,34E-13 | 2,70E-12 | -4,1 |  | -4,3 | 3,82E-04 | 9,04E-04 |
| *ELL* | 4,44E-13 | 2,76E-12 | 3,2 |  | 3,8 | 3,26E-25 | 4,62E-24 |
| *ADPRH* | 4,46E-13 | 2,78E-12 | 14,4 |  | 3,0 | 3,14E-29 | 5,35E-28 |
| *MAP2K1* | 4,53E-13 | 2,82E-12 | 2,3 |  | 3,7 | 1,38E-33 | 2,94E-32 |
| *EYA1* | 4,60E-13 | 2,86E-12 | -6,9 |  | -3,2 | 8,27E-06 | 2,56E-05 |
| *SLC35C1* | 4,61E-13 | 2,86E-12 | 3,0 |  | 1,2 | 7,35E-10 | 3,63E-09 |
| *ESD* | 4,71E-13 | 2,92E-12 | -2,0 |  | -2,3 | 2,01E-02 | 3,43E-02 |
| *FLOT2* | 4,86E-13 | 3,02E-12 | 2,5 |  | 1,4 | 2,27E-11 | 1,28E-10 |
| *SGTB* | 4,87E-13 | 3,02E-12 | 2,3 |  | 0,2 | 2,57E-01 | 3,24E-01 |
| *ABHD4* | 4,87E-13 | 3,02E-12 | -2,9 |  | -3,2 | 3,26E-07 | 1,20E-06 |
| *UBR1* | 5,13E-13 | 3,18E-12 | 2,0 |  | 0,7 | 6,76E-05 | 1,81E-04 |
| *ZNF79* | 5,14E-13 | 3,18E-12 | -3,6 |  | -3,0 | 1,82E-02 | 3,14E-02 |
| *ENO1* | 5,32E-13 | 3,29E-12 | 2,0 |  | 3,5 | 1,18E-10 | 6,21E-10 |
| *AMZ2P1* | 5,48E-13 | 3,39E-12 | -8,0 |  | -3,8 | 4,58E-04 | 1,07E-03 |
| *CRYBG1* | 5,52E-13 | 3,41E-12 | 2,5 |  | 0,4 | 3,08E-02 | 5,01E-02 |
| *MAP3K7CL* | 5,89E-13 | 3,64E-12 | -6,0 |  | -4,3 | 1,56E-02 | 2,72E-02 |
| *P3H2* | 5,99E-13 | 3,70E-12 | 3,0 |  | 5,3 | 1,13E-48 | 4,03E-47 |
| *BAZ2A* | 6,02E-13 | 3,71E-12 | 3,9 |  | 3,2 | 5,42E-26 | 7,94E-25 |
| *RHOBTB1* | 6,08E-13 | 3,74E-12 | -3,4 |  | -15,9 | 2,42E-40 | 6,56E-39 |
| *JUP* | 6,10E-13 | 3,76E-12 | 3,1 |  | -2,6 | 1,55E-01 | 2,10E-01 |
| *APOE* | 6,13E-13 | 3,77E-12 | -9,4 |  | -2,7 | 4,30E-01 | 5,02E-01 |
| *RPL35A* | 6,13E-13 | 3,77E-12 | -2,6 |  | -4,4 | 1,78E-25 | 2,56E-24 |
| *ETV5* | 6,22E-13 | 3,83E-12 | -2,2 |  | 0,0 | 8,30E-01 | 8,63E-01 |
| *SEMA6D* | 6,29E-13 | 3,87E-12 | -45,3 |  | -28,3 | 4,19E-15 | 3,12E-14 |
| *PHC2* | 6,34E-13 | 3,89E-12 | 1,8 |  | 0,6 | 1,36E-04 | 3,47E-04 |
| *KLF6* | 6,57E-13 | 4,03E-12 | 5,0 |  | 6,7 | 3,83E-40 | 1,02E-38 |
| *ZNF655* | 6,62E-13 | 4,06E-12 | 2,3 |  | 0,3 | 2,72E-01 | 3,41E-01 |
| *COL6A1* | 6,66E-13 | 4,09E-12 | -2,4 |  | -4,5 | 7,47E-19 | 7,41E-18 |
| *TBC1D12* | 7,04E-13 | 4,32E-12 | 2,7 |  | 0,4 | 1,20E-01 | 1,67E-01 |
| *RPL24* | 7,09E-13 | 4,34E-12 | -2,3 |  | -5,2 | 8,31E-70 | 4,79E-68 |
| *CLDND1* | 7,29E-13 | 4,46E-12 | 2,4 |  | 0,9 | 8,42E-07 | 2,96E-06 |
| *HSPD1* | 7,36E-13 | 4,51E-12 | -1,9 |  | -3,2 | 3,27E-09 | 1,50E-08 |
| *CEP350* | 7,45E-13 | 4,56E-12 | 2,2 |  | 2,7 | 5,54E-20 | 5,84E-19 |
| *LINC02762* | 7,62E-13 | 4,66E-12 | -7,0 |  | -10,3 | 5,06E-06 | 1,61E-05 |
| *EMX2OS* | 7,64E-13 | 4,67E-12 | -6,2 |  | -24,5 | 2,90E-31 | 5,47E-30 |
| *ABCC3* | 8,01E-13 | 4,89E-12 | 19,5 |  | 3,1 | 1,68E-11 | 9,66E-11 |
| *PPARG* | 8,03E-13 | 4,90E-12 | -44,3 |  | -8,8 | 2,10E-08 | 8,90E-08 |
| *ELOA* | 8,05E-13 | 4,92E-12 | 2,0 |  | 1,7 | 6,68E-08 | 2,68E-07 |
| *RAB20* | 8,20E-13 | 5,00E-12 | 10,8 |  | 21,2 | 6,20E-60 | 2,82E-58 |
| *ARRDC2* | 8,22E-13 | 5,01E-12 | 5,0 |  | 8,8 | 6,86E-94 | 6,15E-92 |
| *TSPAN14* | 8,96E-13 | 5,46E-12 | -2,3 |  | -2,0 | 8,74E-01 | 9,01E-01 |
| *ALDH6A1* | 9,14E-13 | 5,57E-12 | -4,0 |  | -3,2 | 2,11E-06 | 7,03E-06 |
| *DRP2* | 9,17E-13 | 5,58E-12 | -3,5 |  | -10,6 | 8,96E-16 | 7,03E-15 |
| *GALNS* | 9,20E-13 | 5,60E-12 | 2,7 |  | 1,6 | 4,27E-12 | 2,58E-11 |
| *SLC25A3* | 9,36E-13 | 5,69E-12 | -1,8 |  | -3,2 | 1,73E-26 | 2,61E-25 |
| *TMEM64* | 9,44E-13 | 5,74E-12 | -2,3 |  | -3,2 | 3,14E-05 | 8,93E-05 |
| *ADAMTSL1* | 9,64E-13 | 5,85E-12 | -2,2 |  | -2,4 | 1,23E-02 | 2,20E-02 |
| *PNPLA3* | 9,66E-13 | 5,87E-12 | -3,8 |  | -5,0 | 6,10E-06 | 1,92E-05 |
| *FHOD1* | 9,67E-13 | 5,87E-12 | -3,5 |  | -6,6 | 3,60E-34 | 7,83E-33 |
| *ANLN* | 1,05E-12 | 6,36E-12 | -2,8 |  | -3,1 | 2,90E-02 | 4,75E-02 |
| *KLHL36* | 1,08E-12 | 6,52E-12 | -2,2 |  | -4,7 | 2,81E-18 | 2,69E-17 |
| *OLFML2B* | 1,08E-12 | 6,56E-12 | -7,2 |  | 0,0 | 9,85E-01 | 9,88E-01 |
| *GLRX5* | 1,09E-12 | 6,58E-12 | -2,6 |  | -2,4 | 1,09E-02 | 1,96E-02 |
| *FRMD8* | 1,09E-12 | 6,58E-12 | 2,1 |  | 1,3 | 1,48E-12 | 9,23E-12 |
| *MYL12B* | 1,09E-12 | 6,58E-12 | 1,9 |  | 1,3 | 1,91E-15 | 1,46E-14 |
| *FHAD1* | 1,10E-12 | 6,66E-12 | 336,1 |  | 0,7 | 1,63E-01 | 2,19E-01 |
| *ARSK* | 1,12E-12 | 6,74E-12 | -3,0 |  | -5,0 | 8,79E-10 | 4,31E-09 |
| *RNF4* | 1,13E-12 | 6,82E-12 | 2,0 |  | 1,5 | 1,91E-18 | 1,85E-17 |
| *ABCC4* | 1,21E-12 | 7,32E-12 | 2,1 |  | -2,0 | 9,03E-01 | 9,25E-01 |
| *COG5* | 1,25E-12 | 7,56E-12 | 1,8 |  | 0,7 | 4,91E-05 | 1,35E-04 |
| *TPM2* | 1,29E-12 | 7,78E-12 | -2,1 |  | -7,8 | 7,95E-48 | 2,79E-46 |
| *GPR108* | 1,30E-12 | 7,80E-12 | 2,3 |  | 1,0 | 2,44E-09 | 1,14E-08 |
| *STK24* | 1,30E-12 | 7,82E-12 | 2,0 |  | 1,0 | 2,25E-16 | 1,86E-15 |
| *CDK18* | 1,31E-12 | 7,89E-12 | 164,7 |  | 574,2 | 1,62E-15 | 1,24E-14 |
| *ATP5MC3* | 1,32E-12 | 7,92E-12 | -2,1 |  | -2,3 | 7,51E-02 | 1,11E-01 |
| *UNC93B1* | 1,32E-12 | 7,92E-12 | 3,2 |  | 32,4 | 4,95E-167 | 1,17E-164 |
| *BPNT2* | 1,34E-12 | 8,05E-12 | 1,9 |  | 0,4 | 1,02E-01 | 1,45E-01 |
| *LMF2* | 1,35E-12 | 8,08E-12 | 2,5 |  | 2,1 | 8,86E-12 | 5,21E-11 |
| *PSMA4* | 1,35E-12 | 8,11E-12 | 2,0 |  | 2,9 | 6,58E-36 | 1,53E-34 |
| *ACTR3* | 1,35E-12 | 8,12E-12 | 1,8 |  | 0,5 | 9,65E-04 | 2,12E-03 |
| *AP1S2* | 1,38E-12 | 8,28E-12 | 2,5 |  | -2,1 | 7,53E-01 | 7,98E-01 |
| *DUSP22* | 1,40E-12 | 8,38E-12 | 3,1 |  | 0,7 | 1,88E-04 | 4,66E-04 |
| *CHRDL1* | 1,41E-12 | 8,47E-12 | -66,3 |  | -2,4 | 2,32E-01 | 2,97E-01 |
| *NARS2* | 1,42E-12 | 8,47E-12 | -3,5 |  | -4,0 | 5,98E-05 | 1,62E-04 |
| *COPS3* | 1,45E-12 | 8,67E-12 | -2,1 |  | -2,5 | 1,01E-03 | 2,21E-03 |
| *CUEDC1* | 1,45E-12 | 8,67E-12 | -2,7 |  | -2,1 | 5,53E-01 | 6,19E-01 |
| *ZMYM1* | 1,47E-12 | 8,79E-12 | -2,7 |  | -3,5 | 2,82E-05 | 8,07E-05 |
| *MACIR* | 1,48E-12 | 8,82E-12 | 3,1 |  | 0,8 | 3,05E-04 | 7,33E-04 |
| *NUDT16L1* | 1,49E-12 | 8,90E-12 | -2,9 |  | -2,6 | 6,71E-02 | 1,01E-01 |
| *DNM1* | 1,54E-12 | 9,16E-12 | -14,2 |  | -24,5 | 5,03E-03 | 9,72E-03 |
| *EXTL3* | 1,54E-12 | 9,19E-12 | -2,0 |  | -2,6 | 2,66E-03 | 5,43E-03 |
| *PAK1* | 1,55E-12 | 9,23E-12 | -2,0 |  | -2,2 | 3,24E-01 | 3,95E-01 |
| *FAM13A* | 1,55E-12 | 9,24E-12 | -2,8 |  | 0,7 | 3,23E-03 | 6,49E-03 |
| *ZNF438* | 1,57E-12 | 9,35E-12 | 7,2 |  | 2,7 | 2,78E-07 | 1,04E-06 |
| *SLC8B1* | 1,58E-12 | 9,42E-12 | 4,3 |  | 0,7 | 8,67E-02 | 1,26E-01 |
| *TRO* | 1,62E-12 | 9,66E-12 | -2,9 |  | -5,0 | 2,57E-12 | 1,58E-11 |
| *LINC01605* | 1,64E-12 | 9,77E-12 | -49,9 |  | -2,7 | 2,69E-01 | 3,37E-01 |
| *GHR* | 1,65E-12 | 9,83E-12 | -30,8 |  | -48,1 | 1,20E-21 | 1,39E-20 |
| *HACD3* | 1,76E-12 | 1,04E-11 | -1,8 |  | 0,1 | 3,80E-01 | 4,53E-01 |
| *ATF4* | 1,77E-12 | 1,05E-11 | 2,0 |  | 1,1 | 2,48E-13 | 1,64E-12 |
| *WDR48* | 1,77E-12 | 1,05E-11 | 2,1 |  | 1,0 | 3,26E-09 | 1,50E-08 |
| *RCAN2* | 1,83E-12 | 1,09E-11 | -4,3 |  | -5,8 | 1,27E-04 | 3,25E-04 |
| *ABCE1* | 1,90E-12 | 1,13E-11 | -1,9 |  | -4,3 | 5,04E-17 | 4,35E-16 |
| *RARB* | 1,93E-12 | 1,14E-11 | 4,5 |  | 6,9 | 1,25E-23 | 1,61E-22 |
| *TNFRSF11B* | 1,94E-12 | 1,15E-11 | -4,1 |  | 0,5 | 3,20E-03 | 6,42E-03 |
| *NRG1* | 2,04E-12 | 1,21E-11 | 1,8 |  | 0,0 | 9,23E-01 | 9,41E-01 |
| *TTBK2* | 2,04E-12 | 1,21E-11 | 2,3 |  | 0,5 | 1,30E-01 | 1,80E-01 |
| *TBC1D22B* | 2,04E-12 | 1,21E-11 | 2,7 |  | 4,1 | 4,98E-34 | 1,07E-32 |
| *RACK1* | 2,06E-12 | 1,22E-11 | -2,0 |  | -4,7 | 2,67E-42 | 7,87E-41 |
| *PTPN4* | 2,06E-12 | 1,22E-11 | -3,6 |  | -5,0 | 1,94E-20 | 2,11E-19 |
| *MAP3K14* | 2,09E-12 | 1,24E-11 | -5,0 |  | -4,1 | 4,22E-04 | 9,93E-04 |
| *DYNC2H1* | 2,10E-12 | 1,24E-11 | -3,3 |  | -16,0 | 1,97E-32 | 3,94E-31 |
| *TRPA1* | 2,12E-12 | 1,25E-11 | 171,3 |  | 303778,0 | 2,24E-50 | 8,18E-49 |
| *SPRY4* | 2,15E-12 | 1,27E-11 | 3,3 |  | 2,4 | 6,70E-17 | 5,74E-16 |
| *MYO1C* | 2,17E-12 | 1,28E-11 | 2,0 |  | 0,7 | 2,35E-04 | 5,73E-04 |
| *USP10* | 2,19E-12 | 1,29E-11 | 1,9 |  | 1,7 | 1,61E-17 | 1,45E-16 |
| *PDGFRB* | 2,19E-12 | 1,29E-11 | -2,8 |  | -4,3 | 4,93E-11 | 2,68E-10 |
| *FBXO17* | 2,21E-12 | 1,30E-11 | -5,0 |  | -6,4 | 1,02E-14 | 7,37E-14 |
| *USF1* | 2,30E-12 | 1,35E-11 | 2,4 |  | 2,8 | 7,11E-18 | 6,58E-17 |
| *DIS3* | 2,33E-12 | 1,37E-11 | 1,8 |  | -2,3 | 4,07E-02 | 6,43E-02 |
| *TSC22D1* | 2,38E-12 | 1,40E-11 | -1,8 |  | -2,0 | 9,35E-01 | 9,50E-01 |
| *THBS3* | 2,40E-12 | 1,41E-11 | -2,5 |  | -5,9 | 9,46E-27 | 1,45E-25 |
| *PLCE1* | 2,41E-12 | 1,42E-11 | -9,7 |  | -11,0 | 2,17E-20 | 2,35E-19 |
| *DMTF1* | 2,45E-12 | 1,44E-11 | 2,3 |  | 0,0 | 8,83E-01 | 9,07E-01 |
| *COX7C* | 2,64E-12 | 1,55E-11 | -2,1 |  | -3,0 | 5,94E-13 | 3,80E-12 |
| *MCM6* | 2,68E-12 | 1,57E-11 | -3,3 |  | -6,3 | 5,82E-09 | 2,61E-08 |
| *BEST1* | 2,81E-12 | 1,64E-11 | 7,7 |  | 4,8 | 1,34E-07 | 5,21E-07 |
| *TAF8* | 2,81E-12 | 1,64E-11 | 2,3 |  | 2,4 | 1,87E-13 | 1,24E-12 |
| *OSTF1* | 2,81E-12 | 1,64E-11 | 2,2 |  | 0,4 | 3,68E-02 | 5,87E-02 |
| *EPS15* | 2,84E-12 | 1,66E-11 | 1,9 |  | 1,4 | 6,87E-28 | 1,11E-26 |
| *AIDA* | 2,94E-12 | 1,72E-11 | 2,9 |  | 1,1 | 7,17E-08 | 2,87E-07 |
| *GPBP1L1* | 3,02E-12 | 1,76E-11 | 2,0 |  | 1,3 | 1,08E-16 | 9,08E-16 |
| *ZBTB1* | 3,02E-12 | 1,77E-11 | 2,0 |  | 0,1 | 4,51E-01 | 5,22E-01 |
| *TOMM20* | 3,05E-12 | 1,78E-11 | -1,7 |  | -3,2 | 8,70E-08 | 3,45E-07 |
| *MARCHF3* | 3,09E-12 | 1,80E-11 | 7,7 |  | 42,3 | 1,22E-10 | 6,41E-10 |
| *ITPKC* | 3,13E-12 | 1,82E-11 | 2,9 |  | 0,7 | 6,86E-04 | 1,55E-03 |
| *H2AZ1* | 3,23E-12 | 1,88E-11 | -2,2 |  | -2,4 | 9,56E-02 | 1,37E-01 |
| *FEM1C* | 3,33E-12 | 1,94E-11 | 2,8 |  | 1,3 | 1,64E-09 | 7,79E-09 |
| *ZBTB18* | 3,35E-12 | 1,95E-11 | -3,2 |  | 0,0 | 9,57E-01 | 9,67E-01 |
| *DCPS* | 3,47E-12 | 2,02E-11 | -3,1 |  | -2,6 | 5,01E-02 | 7,74E-02 |
| *RPS18* | 3,51E-12 | 2,04E-11 | -2,4 |  | -5,7 | 1,33E-40 | 3,63E-39 |
| *SNX7* | 3,61E-12 | 2,10E-11 | 2,2 |  | -2,1 | 5,07E-01 | 5,77E-01 |
| *ARHGAP20* | 3,62E-12 | 2,10E-11 | 21,1 |  | 0,9 | 1,64E-02 | 2,85E-02 |
| *XPR1* | 3,65E-12 | 2,12E-11 | 1,8 |  | 1,2 | 1,41E-11 | 8,18E-11 |
| *RPS27L* | 3,70E-12 | 2,15E-11 | -2,0 |  | -3,3 | 5,42E-10 | 2,70E-09 |
| *PCDH17* | 3,80E-12 | 2,20E-11 | 10,5 |  | 719,1 | 1,68E-28 | 2,76E-27 |
| *SLC25A43* | 3,90E-12 | 2,26E-11 | 3,0 |  | 0,3 | 5,74E-02 | 8,73E-02 |
| *SYBU* | 3,94E-12 | 2,29E-11 | 22,3 |  | 11,1 | 2,88E-02 | 4,72E-02 |
| *CKAP4* | 3,95E-12 | 2,29E-11 | 1,7 |  | 3,9 | 9,47E-15 | 6,90E-14 |
| *NADK2* | 3,96E-12 | 2,29E-11 | -2,8 |  | -5,1 | 7,78E-11 | 4,17E-10 |
| *CYTOR* | 3,97E-12 | 2,30E-11 | 2,4 |  | 0,2 | 2,95E-01 | 3,65E-01 |
| *ASAP3* | 4,01E-12 | 2,32E-11 | 2,6 |  | 0,1 | 6,59E-01 | 7,15E-01 |
| *KIF2A* | 4,04E-12 | 2,33E-11 | 2,1 |  | 0,1 | 6,20E-01 | 6,80E-01 |
| *DCAKD* | 4,08E-12 | 2,36E-11 | -2,4 |  | -2,2 | 8,66E-02 | 1,26E-01 |
| *WHAMMP3* | 4,10E-12 | 2,37E-11 | 10,0 |  | 2,3 | 1,54E-05 | 4,56E-05 |
| *DAB2IP* | 4,10E-12 | 2,37E-11 | 2,7 |  | 5,2 | 5,88E-21 | 6,58E-20 |
| *CD109* | 4,11E-12 | 2,38E-11 | -2,5 |  | -10,8 | 4,24E-54 | 1,69E-52 |
| *EML3* | 4,17E-12 | 2,40E-11 | 2,9 |  | -2,2 | 4,10E-01 | 4,82E-01 |
| *TAB2* | 4,19E-12 | 2,42E-11 | 2,8 |  | 1,3 | 1,31E-14 | 9,43E-14 |
| *PITHD1* | 4,19E-12 | 2,42E-11 | -2,5 |  | -3,4 | 2,86E-09 | 1,33E-08 |
| *H1-0* | 4,29E-12 | 2,47E-11 | 3,7 |  | 13,2 | 6,99E-81 | 5,14E-79 |
| *DAB2* | 4,34E-12 | 2,50E-11 | -1,8 |  | -2,4 | 2,03E-03 | 4,24E-03 |
| *BCKDK* | 4,40E-12 | 2,53E-11 | 2,1 |  | 2,0 | 3,70E-06 | 1,19E-05 |
| *RPS2P5* | 4,46E-12 | 2,57E-11 | -2,5 |  | -6,8 | 1,70E-16 | 1,41E-15 |
| *SCUBE3* | 4,56E-12 | 2,62E-11 | -6,1 |  | -5,3 | 6,19E-03 | 1,18E-02 |
| *VLDLR* | 4,86E-12 | 2,79E-11 | 4,3 |  | 13,6 | 1,84E-11 | 1,06E-10 |
| *SCML1* | 5,00E-12 | 2,87E-11 | 5,7 |  | 4,4 | 1,25E-12 | 7,84E-12 |
| *FABP5* | 5,06E-12 | 2,90E-11 | -3,6 |  | -6,8 | 1,02E-09 | 4,94E-09 |
| *SERPINE2* | 5,06E-12 | 2,90E-11 | -4,7 |  | -3,5 | 4,37E-02 | 6,85E-02 |
| *ADAMTSL4* | 5,08E-12 | 2,91E-11 | 10,8 |  | 0,4 | 2,87E-01 | 3,57E-01 |
| *RNF13* | 5,12E-12 | 2,93E-11 | 2,5 |  | 2,4 | 1,09E-15 | 8,48E-15 |
| *WSB2* | 5,19E-12 | 2,97E-11 | -1,8 |  | -2,0 | 9,38E-01 | 9,52E-01 |
| *SSBP2* | 5,27E-12 | 3,02E-11 | -4,1 |  | -2,7 | 4,40E-05 | 1,22E-04 |
| *RAC1* | 5,33E-12 | 3,05E-11 | 1,8 |  | 0,8 | 3,84E-13 | 2,50E-12 |
| *CARS1* | 5,34E-12 | 3,05E-11 | 2,3 |  | 1,2 | 2,03E-14 | 1,44E-13 |
| *L3MBTL2-AS1* | 5,65E-12 | 3,23E-11 | 13,6 |  | 13,2 | 3,42E-15 | 2,56E-14 |
| *YBX1* | 5,93E-12 | 3,39E-11 | -1,9 |  | -3,1 | 5,70E-07 | 2,04E-06 |
| *DYSF* | 6,06E-12 | 3,46E-11 | -5,4 |  | 3,7 | 5,94E-02 | 9,01E-02 |
| *OIP5-AS1* | 6,29E-12 | 3,59E-11 | -2,0 |  | -6,6 | 2,68E-18 | 2,57E-17 |
| *CHN1* | 6,33E-12 | 3,61E-11 | -2,2 |  | 1,9 | 7,61E-10 | 3,75E-09 |
| *UXS1* | 6,50E-12 | 3,70E-11 | 2,0 |  | 1,0 | 3,74E-08 | 1,54E-07 |
| *CYB5R3* | 6,56E-12 | 3,74E-11 | 2,0 |  | 0,9 | 2,35E-06 | 7,76E-06 |
| *CD99* | 6,64E-12 | 3,78E-11 | 1,7 |  | -2,0 | 7,99E-01 | 8,37E-01 |
| *ETFRF1* | 6,85E-12 | 3,89E-11 | -3,4 |  | -3,0 | 4,58E-03 | 8,90E-03 |
| *RPSA* | 6,91E-12 | 3,93E-11 | -2,4 |  | -5,4 | 1,45E-23 | 1,85E-22 |
| *WDR3* | 6,94E-12 | 3,95E-11 | -2,3 |  | -3,1 | 2,41E-08 | 1,01E-07 |
| *PELI1* | 7,04E-12 | 4,00E-11 | 3,8 |  | 6,6 | 3,38E-26 | 5,02E-25 |
| *PHB* | 7,06E-12 | 4,01E-11 | -1,9 |  | -2,2 | 2,00E-01 | 2,61E-01 |
| *THRA* | 7,06E-12 | 4,01E-11 | -3,1 |  | -2,5 | 5,29E-03 | 1,02E-02 |
| *VEZT* | 7,21E-12 | 4,09E-11 | 2,0 |  | 0,6 | 6,83E-04 | 1,54E-03 |
| *SELENOK* | 7,30E-12 | 4,14E-11 | 2,5 |  | 1,3 | 3,11E-10 | 1,58E-09 |
| *RRAS* | 7,31E-12 | 4,14E-11 | 2,1 |  | 1,7 | 1,21E-06 | 4,17E-06 |
| *TES* | 7,37E-12 | 4,17E-11 | 2,5 |  | 0,5 | 5,65E-04 | 1,30E-03 |
| *TMEM219* | 7,47E-12 | 4,23E-11 | 2,0 |  | 1,5 | 1,71E-11 | 9,82E-11 |
| *NEGR1* | 7,56E-12 | 4,28E-11 | -4,5 |  | -5,9 | 1,06E-20 | 1,17E-19 |
| *TYW5* | 7,72E-12 | 4,36E-11 | 2,5 |  | 1,0 | 4,24E-03 | 8,31E-03 |
| *SIPA1* | 7,98E-12 | 4,51E-11 | 2,2 |  | 3,1 | 4,42E-32 | 8,68E-31 |
| *DUSP5* | 8,27E-12 | 4,67E-11 | 2,1 |  | 12,5 | 3,04E-20 | 3,27E-19 |
| *STOML1* | 8,54E-12 | 4,82E-11 | 3,3 |  | 2,3 | 7,38E-16 | 5,83E-15 |
| *P4HA2* | 8,72E-12 | 4,93E-11 | 4,7 |  | 3,2 | 2,21E-03 | 4,57E-03 |
| *COL6A3* | 8,76E-12 | 4,95E-11 | -4,7 |  | -46,8 | 3,83E-80 | 2,75E-78 |
| *TXNDC11* | 8,78E-12 | 4,95E-11 | 2,4 |  | 1,9 | 1,82E-10 | 9,48E-10 |
| *CNOT8* | 9,05E-12 | 5,10E-11 | 2,0 |  | 2,2 | 6,75E-15 | 4,97E-14 |
| *ZDHHC9* | 9,24E-12 | 5,21E-11 | 2,7 |  | 2,1 | 2,52E-08 | 1,06E-07 |
| *STK17B* | 9,24E-12 | 5,21E-11 | -2,1 |  | -3,8 | 4,56E-15 | 3,39E-14 |
| *TFDP2* | 9,27E-12 | 5,22E-11 | -2,5 |  | -2,8 | 2,19E-03 | 4,55E-03 |
| *PNMA2* | 9,39E-12 | 5,28E-11 | 16,2 |  | 0,5 | 1,05E-02 | 1,89E-02 |
| *SNAPC5* | 9,45E-12 | 5,32E-11 | -4,6 |  | -3,0 | 3,71E-05 | 1,04E-04 |
| *CEP78* | 9,76E-12 | 5,49E-11 | -2,6 |  | -6,5 | 1,36E-19 | 1,41E-18 |
| *EPHB4* | 9,97E-12 | 5,60E-11 | -2,6 |  | -2,8 | 2,24E-04 | 5,49E-04 |
| *TMTC3* | 1,09E-11 | 6,12E-11 | -2,5 |  | -3,0 | 6,66E-05 | 1,79E-04 |
| *SPTBN5* | 1,13E-11 | 6,34E-11 | 6,5 |  | 9,9 | 4,14E-04 | 9,75E-04 |
| *MAST4* | 1,14E-11 | 6,42E-11 | 3,3 |  | 0,8 | 7,14E-03 | 1,34E-02 |
| *UEVLD* | 1,15E-11 | 6,43E-11 | 2,1 |  | 0,5 | 2,36E-03 | 4,86E-03 |
| *RUVBL1* | 1,16E-11 | 6,49E-11 | -2,2 |  | -2,8 | 1,66E-05 | 4,91E-05 |
| *CARD10* | 1,16E-11 | 6,49E-11 | -5,9 |  | -4,9 | 1,74E-04 | 4,35E-04 |
| *BTD* | 1,17E-11 | 6,55E-11 | -3,4 |  | -2,5 | 1,53E-02 | 2,67E-02 |
| *PLEKHO2* | 1,17E-11 | 6,57E-11 | 2,7 |  | 2,6 | 2,84E-32 | 5,63E-31 |
| *MRPL36* | 1,19E-11 | 6,66E-11 | -2,9 |  | -3,4 | 1,76E-06 | 5,92E-06 |
| *NAV2-AS6* | 1,20E-11 | 6,73E-11 | 37,5 |  | 439,8 | 3,01E-23 | 3,78E-22 |
| *MON1A* | 1,21E-11 | 6,74E-11 | -3,3 |  | -2,4 | 3,33E-02 | 5,38E-02 |
| *AMD1* | 1,22E-11 | 6,82E-11 | -1,9 |  | -2,4 | 7,17E-02 | 1,06E-01 |
| *SMARCD3* | 1,26E-11 | 7,03E-11 | -2,8 |  | -3,3 | 5,07E-04 | 1,17E-03 |
| *GLRB* | 1,26E-11 | 7,06E-11 | -5,4 |  | -5,6 | 2,08E-10 | 1,08E-09 |
| *CHST15* | 1,27E-11 | 7,07E-11 | 28,7 |  | 7,2 | 1,91E-14 | 1,36E-13 |
| *HS3ST3A1* | 1,28E-11 | 7,15E-11 | 3,4 |  | 3,4 | 1,42E-07 | 5,51E-07 |
| *SLC16A4* | 1,31E-11 | 7,29E-11 | 2,2 |  | 6,4 | 2,07E-129 | 3,26E-127 |
| *RMDN3* | 1,31E-11 | 7,29E-11 | 2,4 |  | 2,0 | 1,00E-16 | 8,48E-16 |
| *MAX* | 1,33E-11 | 7,43E-11 | 2,0 |  | 1,5 | 2,11E-22 | 2,54E-21 |
| *NOL9* | 1,36E-11 | 7,54E-11 | -2,1 |  | -3,3 | 1,85E-06 | 6,20E-06 |
| *LRRN4CL* | 1,38E-11 | 7,67E-11 | -89,0 |  | -52,3 | 9,42E-21 | 1,04E-19 |
| *C6orf47* | 1,38E-11 | 7,67E-11 | 2,3 |  | 1,2 | 1,22E-07 | 4,73E-07 |
| *ARMC9* | 1,38E-11 | 7,67E-11 | -2,1 |  | -14,6 | 8,93E-23 | 1,10E-21 |
| *TCP11L1* | 1,45E-11 | 8,03E-11 | 2,2 |  | 0,6 | 5,01E-03 | 9,68E-03 |
| *TP53INP2* | 1,56E-11 | 8,65E-11 | 6,9 |  | 0,5 | 7,65E-02 | 1,13E-01 |
| *PDGFC* | 1,57E-11 | 8,69E-11 | -1,9 |  | -3,9 | 1,37E-26 | 2,07E-25 |
| *PEX2* | 1,57E-11 | 8,69E-11 | -2,3 |  | -3,3 | 6,98E-16 | 5,54E-15 |
| *SUN1* | 1,59E-11 | 8,82E-11 | -2,3 |  | -2,8 | 1,15E-04 | 2,95E-04 |
| *MBNL2* | 1,67E-11 | 9,25E-11 | -2,2 |  | -6,6 | 2,14E-22 | 2,58E-21 |
| *TTLL12* | 1,67E-11 | 9,27E-11 | -2,6 |  | -2,2 | 4,87E-01 | 5,58E-01 |
| *SERBP1* | 1,68E-11 | 9,28E-11 | -1,7 |  | -3,8 | 6,07E-22 | 7,11E-21 |
| *MYCBP2* | 1,68E-11 | 9,29E-11 | 2,2 |  | -3,0 | 1,94E-03 | 4,07E-03 |
| *GINS4* | 1,68E-11 | 9,29E-11 | -4,1 |  | -4,1 | 1,31E-02 | 2,32E-02 |
| *RUSC2* | 1,69E-11 | 9,35E-11 | 2,0 |  | 1,3 | 8,29E-12 | 4,89E-11 |
| *EPB41L2* | 1,69E-11 | 9,36E-11 | 2,5 |  | 0,4 | 7,46E-03 | 1,39E-02 |
| *ELF4* | 1,72E-11 | 9,48E-11 | 3,2 |  | 4,9 | 2,53E-25 | 3,62E-24 |
| *SLC25A51* | 1,72E-11 | 9,50E-11 | -2,8 |  | -4,4 | 3,19E-19 | 3,25E-18 |
| *GSTP1* | 1,73E-11 | 9,53E-11 | -2,0 |  | -3,0 | 3,64E-05 | 1,02E-04 |
| *IFT172* | 1,73E-11 | 9,55E-11 | -2,9 |  | -11,9 | 3,81E-36 | 8,90E-35 |
| *WASHC2C* | 1,74E-11 | 9,58E-11 | 1,8 |  | 0,8 | 3,15E-04 | 7,57E-04 |
| *ZFAND2A* | 1,76E-11 | 9,69E-11 | 3,5 |  | 4,2 | 1,04E-23 | 1,33E-22 |
| *ZNF101* | 1,78E-11 | 9,81E-11 | 3,1 |  | 0,2 | 2,36E-01 | 3,01E-01 |
| *TMEM59* | 1,80E-11 | 9,92E-11 | 2,1 |  | 2,5 | 4,02E-27 | 6,29E-26 |
| *LOX* | 1,81E-11 | 9,97E-11 | 5,3 |  | 2,3 | 3,38E-03 | 6,75E-03 |
| *WDR43* | 1,81E-11 | 9,97E-11 | -1,8 |  | -2,5 | 3,94E-03 | 7,78E-03 |
| *NET1* | 1,87E-11 | 1,03E-10 | -2,1 |  | -5,0 | 6,56E-08 | 2,64E-07 |
| *KCNC4* | 1,88E-11 | 1,04E-10 | -5,0 |  | -2,6 | 1,98E-01 | 2,59E-01 |
| *NTN1* | 1,90E-11 | 1,04E-10 | 783,5 |  | 170,1 | 8,06E-15 | 5,89E-14 |
| *RPSAP52* | 1,90E-11 | 1,05E-10 | 11,1 |  | 5,8 | 4,74E-13 | 3,06E-12 |
| *LRP3* | 1,93E-11 | 1,06E-10 | -3,4 |  | -4,4 | 7,93E-14 | 5,41E-13 |
| *NDFIP2* | 1,94E-11 | 1,06E-10 | -2,1 |  | -2,1 | 6,61E-01 | 7,17E-01 |
| *ME2* | 1,96E-11 | 1,08E-10 | 1,9 |  | 1,0 | 7,53E-07 | 2,66E-06 |
| *FZD5* | 2,03E-11 | 1,11E-10 | 122,1 |  | 0,8 | 4,24E-01 | 4,96E-01 |
| *DIAPH3* | 2,05E-11 | 1,12E-10 | -2,8 |  | -2,7 | 1,56E-01 | 2,11E-01 |
| *RNF169* | 2,07E-11 | 1,13E-10 | 2,0 |  | 1,0 | 1,98E-11 | 1,13E-10 |
| *WDR77* | 2,09E-11 | 1,14E-10 | -2,6 |  | -3,6 | 3,45E-05 | 9,73E-05 |
| *CALM1* | 2,12E-11 | 1,16E-10 | 1,7 |  | 0,1 | 4,03E-01 | 4,75E-01 |
| *ANKRD29* | 2,13E-11 | 1,17E-10 | -7,5 |  | -8,0 | 4,18E-31 | 7,80E-30 |
| *TMA7* | 2,14E-11 | 1,17E-10 | -2,0 |  | -4,5 | 1,62E-16 | 1,35E-15 |
| *BOK* | 2,15E-11 | 1,17E-10 | 2,5 |  | 1,2 | 3,73E-08 | 1,54E-07 |
| *SERP1* | 2,22E-11 | 1,21E-10 | -1,7 |  | -2,9 | 4,22E-05 | 1,17E-04 |
| *FRY* | 2,28E-11 | 1,25E-10 | -21,5 |  | -8,4 | 2,58E-11 | 1,45E-10 |
| *RIOX2* | 2,29E-11 | 1,25E-10 | -2,0 |  | -3,8 | 2,72E-13 | 1,78E-12 |
| *NXN* | 2,32E-11 | 1,27E-10 | 2,2 |  | -2,1 | 7,43E-01 | 7,89E-01 |
| *ITSN2* | 2,38E-11 | 1,30E-10 | 2,5 |  | 0,3 | 1,99E-01 | 2,60E-01 |
| *ENSG00000239665* | 2,40E-11 | 1,31E-10 | -7,4 |  | -24,3 | 4,67E-19 | 4,69E-18 |
| *CDR2L* | 2,45E-11 | 1,33E-10 | 2,4 |  | 0,3 | 8,08E-02 | 1,18E-01 |
| *METTL9* | 2,48E-11 | 1,35E-10 | -1,7 |  | -2,2 | 1,97E-01 | 2,58E-01 |
| *PSAT1* | 2,52E-11 | 1,37E-10 | -2,5 |  | 0,6 | 1,84E-01 | 2,43E-01 |
| *RPL18* | 2,56E-11 | 1,39E-10 | -1,9 |  | -3,7 | 1,87E-18 | 1,81E-17 |
| *PCYOX1L* | 2,58E-11 | 1,40E-10 | -5,2 |  | -4,3 | 4,60E-06 | 1,47E-05 |
| *PAIP2* | 2,60E-11 | 1,41E-10 | 2,4 |  | 2,2 | 2,06E-16 | 1,70E-15 |
| *ZNF428* | 2,62E-11 | 1,42E-10 | -2,3 |  | -3,1 | 1,47E-05 | 4,37E-05 |
| *NAXE* | 2,62E-11 | 1,42E-10 | -2,4 |  | -2,3 | 6,66E-02 | 9,98E-02 |
| *PLEKHN1* | 2,64E-11 | 1,43E-10 | 22,8 |  | 31,0 | 6,08E-24 | 7,93E-23 |
| *CNN3* | 2,65E-11 | 1,44E-10 | -1,8 |  | 1,0 | 4,33E-08 | 1,77E-07 |
| *NDRG3* | 2,65E-11 | 1,44E-10 | -2,0 |  | -2,8 | 2,24E-06 | 7,42E-06 |
| *EPHB2* | 2,67E-11 | 1,45E-10 | -2,7 |  | 4,4 | 2,54E-11 | 1,43E-10 |
| *PKN1* | 2,71E-11 | 1,47E-10 | 1,9 |  | 0,7 | 1,15E-05 | 3,49E-05 |
| *ANK3* | 2,79E-11 | 1,51E-10 | 3,8 |  | -7,5 | 1,15E-04 | 2,95E-04 |
| *PPP2R5B* | 2,84E-11 | 1,54E-10 | 2,3 |  | 1,7 | 3,08E-08 | 1,28E-07 |
| *TFPI* | 2,88E-11 | 1,56E-10 | 2,8 |  | 0,0 | 9,78E-01 | 9,83E-01 |
| *AGO4* | 2,89E-11 | 1,56E-10 | 2,4 |  | 0,4 | 3,17E-02 | 5,13E-02 |
| *SMCR8* | 2,94E-11 | 1,59E-10 | 2,1 |  | 1,6 | 2,60E-10 | 1,34E-09 |
| *RABEPK* | 2,94E-11 | 1,59E-10 | -2,7 |  | -4,2 | 1,07E-06 | 3,71E-06 |
| *DECR1* | 2,96E-11 | 1,60E-10 | 1,8 |  | 1,4 | 1,48E-21 | 1,71E-20 |
| *DDX23* | 3,10E-11 | 1,67E-10 | 2,2 |  | 1,5 | 2,33E-22 | 2,79E-21 |
| *CHD3* | 3,12E-11 | 1,68E-10 | -2,1 |  | -5,9 | 5,70E-42 | 1,66E-40 |
| *RAB4A* | 3,14E-11 | 1,69E-10 | -2,1 |  | -2,9 | 1,24E-10 | 6,51E-10 |
| *ZNF217* | 3,20E-11 | 1,72E-10 | 2,4 |  | 1,0 | 6,20E-13 | 3,96E-12 |
| *SIRT7* | 3,36E-11 | 1,81E-10 | 3,1 |  | 2,1 | 3,55E-13 | 2,31E-12 |
| *VPS9D1* | 3,37E-11 | 1,81E-10 | 3,6 |  | 1,3 | 1,64E-07 | 6,28E-07 |
| *RAI14* | 3,44E-11 | 1,85E-10 | 1,8 |  | 1,1 | 3,52E-11 | 1,95E-10 |
| *SLC35F6* | 3,48E-11 | 1,87E-10 | 2,2 |  | 0,8 | 1,93E-04 | 4,78E-04 |
| *NDUFB2* | 3,58E-11 | 1,92E-10 | -2,1 |  | -2,3 | 2,25E-01 | 2,89E-01 |
| *UBC* | 3,58E-11 | 1,93E-10 | 1,8 |  | 2,1 | 1,36E-17 | 1,23E-16 |
| *CSRP1* | 3,71E-11 | 1,99E-10 | 1,9 |  | -2,1 | 7,79E-01 | 8,20E-01 |
| *FBXO22* | 3,72E-11 | 2,00E-10 | -1,9 |  | -2,8 | 8,67E-05 | 2,28E-04 |
| *TOM1L2* | 3,79E-11 | 2,03E-10 | -2,0 |  | -2,0 | 8,33E-01 | 8,66E-01 |
| *ETV3* | 3,82E-11 | 2,05E-10 | 2,5 |  | 0,5 | 2,16E-03 | 4,49E-03 |
| *PRELID1* | 3,90E-11 | 2,09E-10 | 1,8 |  | 1,0 | 1,65E-04 | 4,15E-04 |
| *FOXO3* | 4,06E-11 | 2,18E-10 | 2,4 |  | 0,2 | 3,11E-01 | 3,82E-01 |
| *RAB6A* | 4,11E-11 | 2,20E-10 | 1,7 |  | 0,3 | 1,17E-02 | 2,10E-02 |
| *TLR2* | 4,26E-11 | 2,28E-10 | 2394,2 |  | 4731,4 | 7,83E-19 | 7,75E-18 |
| *SNHG4* | 4,33E-11 | 2,32E-10 | -5,0 |  | -32,4 | 4,75E-05 | 1,31E-04 |
| *TRIAP1* | 4,36E-11 | 2,33E-10 | -3,3 |  | -3,5 | 5,56E-06 | 1,76E-05 |
| *PATL2* | 4,44E-11 | 2,38E-10 | 73,6 |  | 4,0 | 2,54E-03 | 5,21E-03 |
| *TNFSF13B* | 4,48E-11 | 2,39E-10 | 1005,6 |  | 575,4 | 2,50E-27 | 3,95E-26 |
| *LRP5* | 4,56E-11 | 2,43E-10 | -3,6 |  | -3,6 | 2,79E-06 | 9,12E-06 |
| *ELP1* | 4,65E-11 | 2,48E-10 | -1,9 |  | -2,7 | 2,02E-06 | 6,75E-06 |
| *STX4* | 4,66E-11 | 2,48E-10 | 2,5 |  | 0,8 | 3,89E-05 | 1,09E-04 |
| *ARL6IP6* | 4,68E-11 | 2,50E-10 | 2,8 |  | 3,6 | 1,88E-18 | 1,82E-17 |
| *SLC25A24* | 4,70E-11 | 2,50E-10 | 1,9 |  | 0,6 | 1,13E-02 | 2,03E-02 |
| *AFDN* | 4,72E-11 | 2,51E-10 | 2,2 |  | 1,3 | 7,78E-08 | 3,10E-07 |
| *PLPP2* | 4,74E-11 | 2,52E-10 | -5,1 |  | -2,3 | 3,45E-01 | 4,17E-01 |
| *DNAJC7* | 4,99E-11 | 2,65E-10 | 1,8 |  | 1,0 | 5,16E-12 | 3,10E-11 |
| *MAP7D1* | 4,99E-11 | 2,65E-10 | 1,9 |  | 1,7 | 1,88E-11 | 1,07E-10 |
| *BLVRB* | 5,01E-11 | 2,66E-10 | -2,5 |  | -3,4 | 8,09E-12 | 4,77E-11 |
| *NCDN* | 5,02E-11 | 2,67E-10 | -2,6 |  | -2,0 | 8,86E-01 | 9,10E-01 |
| *CCDC43* | 5,06E-11 | 2,69E-10 | -2,2 |  | -2,7 | 9,89E-05 | 2,58E-04 |
| *ZNF440* | 5,13E-11 | 2,72E-10 | -2,6 |  | -3,1 | 3,96E-04 | 9,35E-04 |
| *TSPYL4* | 5,21E-11 | 2,77E-10 | -3,9 |  | -2,6 | 3,34E-03 | 6,69E-03 |
| *CXCL12* | 5,23E-11 | 2,77E-10 | 2,7 |  | -9,8 | 7,20E-62 | 3,44E-60 |
| *RPL21* | 5,28E-11 | 2,80E-10 | -2,4 |  | -5,7 | 2,91E-39 | 7,63E-38 |
| *SGSH* | 5,37E-11 | 2,84E-10 | 4,4 |  | 1,4 | 7,62E-07 | 2,69E-06 |
| *TIGAR* | 5,57E-11 | 2,95E-10 | -3,0 |  | -4,2 | 5,24E-03 | 1,01E-02 |
| *DDI2* | 5,58E-11 | 2,95E-10 | 2,7 |  | -2,3 | 2,86E-01 | 3,56E-01 |
| *C11orf24* | 5,62E-11 | 2,97E-10 | -2,1 |  | -2,9 | 1,78E-02 | 3,08E-02 |
| *RALBP1* | 5,66E-11 | 2,99E-10 | 1,9 |  | 0,3 | 5,66E-03 | 1,08E-02 |
| *IER2* | 5,69E-11 | 3,01E-10 | 3,4 |  | 2,9 | 7,06E-09 | 3,13E-08 |
| *NCBP2* | 5,72E-11 | 3,02E-10 | -1,9 |  | -2,5 | 4,36E-07 | 1,59E-06 |
| *COQ10B* | 5,77E-11 | 3,05E-10 | 1,9 |  | 1,4 | 6,19E-10 | 3,07E-09 |
| *ENSG00000226380* | 5,78E-11 | 3,05E-10 | 3,3 |  | 0,3 | 1,96E-01 | 2,57E-01 |
| *TRIM32* | 5,86E-11 | 3,09E-10 | -2,1 |  | -2,3 | 1,58E-02 | 2,75E-02 |
| *PFN1* | 5,89E-11 | 3,11E-10 | 1,8 |  | 0,8 | 9,08E-05 | 2,38E-04 |
| *CEBPG* | 5,97E-11 | 3,14E-10 | 3,0 |  | 0,7 | 3,12E-04 | 7,48E-04 |
| *BCL7B* | 6,12E-11 | 3,22E-10 | 2,1 |  | 1,2 | 9,75E-08 | 3,84E-07 |
| *KPNA5* | 6,21E-11 | 3,27E-10 | 3,3 |  | 2,4 | 3,51E-09 | 1,61E-08 |
| *NR2F1-AS1* | 6,27E-11 | 3,30E-10 | -3,9 |  | -2,5 | 6,95E-02 | 1,04E-01 |
| *EFNB1* | 6,31E-11 | 3,32E-10 | 2,7 |  | -3,1 | 1,89E-02 | 3,25E-02 |
| *TGFB1* | 6,34E-11 | 3,33E-10 | 3,9 |  | 0,3 | 1,91E-01 | 2,51E-01 |
| *VPS37B* | 6,35E-11 | 3,34E-10 | -2,7 |  | -2,3 | 1,01E-01 | 1,44E-01 |
| *C6orf132* | 6,44E-11 | 3,38E-10 | 6,2 |  | 7,4 | 6,25E-11 | 3,38E-10 |
| *SLC35B2* | 6,44E-11 | 3,38E-10 | -2,5 |  | -2,9 | 7,73E-04 | 1,73E-03 |
| *DHRS7B* | 6,48E-11 | 3,40E-10 | 3,3 |  | 1,3 | 2,29E-04 | 5,60E-04 |
| *NSMF* | 6,53E-11 | 3,43E-10 | -2,2 |  | -4,0 | 3,08E-17 | 2,71E-16 |
| *C3orf14* | 6,67E-11 | 3,50E-10 | 2,9 |  | 2,0 | 5,20E-07 | 1,87E-06 |
| *SIX5* | 6,75E-11 | 3,54E-10 | 5,4 |  | 1,1 | 1,97E-04 | 4,87E-04 |
| *DCXR* | 6,89E-11 | 3,61E-10 | -4,4 |  | -2,9 | 2,42E-03 | 4,97E-03 |
| *MTCL1* | 6,90E-11 | 3,62E-10 | -2,4 |  | -7,8 | 5,99E-30 | 1,05E-28 |
| *SORD* | 6,91E-11 | 3,62E-10 | -2,3 |  | -2,7 | 1,02E-02 | 1,85E-02 |
| *PKIG* | 7,07E-11 | 3,70E-10 | 2,2 |  | -2,7 | 6,45E-03 | 1,22E-02 |
| *POLR2F* | 7,10E-11 | 3,72E-10 | -2,2 |  | -2,5 | 5,32E-02 | 8,17E-02 |
| *MCCC1* | 7,16E-11 | 3,74E-10 | -2,2 |  | -2,5 | 1,59E-02 | 2,78E-02 |
| *NDC1* | 7,24E-11 | 3,78E-10 | -2,1 |  | -2,2 | 4,92E-01 | 5,63E-01 |
| *VAT1* | 7,28E-11 | 3,80E-10 | -2,0 |  | -6,5 | 1,99E-62 | 9,64E-61 |
| *BOP1* | 7,41E-11 | 3,87E-10 | -2,4 |  | -3,6 | 1,69E-05 | 5,00E-05 |
| *CLIP1* | 7,53E-11 | 3,93E-10 | 1,9 |  | 0,7 | 1,77E-05 | 5,22E-05 |
| *RBM22* | 7,63E-11 | 3,98E-10 | 2,0 |  | 1,6 | 2,00E-13 | 1,33E-12 |
| *APBB2* | 7,88E-11 | 4,11E-10 | -1,9 |  | 0,1 | 4,85E-01 | 5,56E-01 |
| *B4GALT6* | 7,90E-11 | 4,12E-10 | -3,7 |  | -8,4 | 4,61E-05 | 1,27E-04 |
| *PCNA* | 7,93E-11 | 4,13E-10 | -2,2 |  | -2,8 | 4,19E-08 | 1,72E-07 |
| *MRPS27* | 8,03E-11 | 4,18E-10 | -2,1 |  | -3,1 | 1,41E-14 | 1,01E-13 |
| *TEX2* | 8,05E-11 | 4,19E-10 | -2,0 |  | -3,6 | 8,48E-21 | 9,40E-20 |
| *ODF2L* | 8,11E-11 | 4,22E-10 | 2,4 |  | 0,8 | 8,71E-05 | 2,29E-04 |
| *SIX1* | 8,48E-11 | 4,41E-10 | -1,8 |  | 9,9 | 2,42E-38 | 6,14E-37 |
| *C2orf27A* | 8,54E-11 | 4,44E-10 | 4,4 |  | 3,6 | 7,58E-10 | 3,73E-09 |
| *SLC2A13* | 9,06E-11 | 4,70E-10 | 3,7 |  | -2,1 | 8,01E-01 | 8,39E-01 |
| *LOXL4* | 9,20E-11 | 4,77E-10 | -3,2 |  | -3,2 | 9,71E-02 | 1,39E-01 |
| *CAMK1D* | 9,41E-11 | 4,88E-10 | 7,9 |  | 2,3 | 3,47E-14 | 2,42E-13 |
| *LDHA* | 9,53E-11 | 4,94E-10 | 1,8 |  | 1,4 | 9,62E-04 | 2,12E-03 |
| *USO1* | 9,57E-11 | 4,96E-10 | 1,8 |  | 0,1 | 3,90E-01 | 4,62E-01 |
| *PPP1R12A* | 9,60E-11 | 4,97E-10 | 1,8 |  | 0,9 | 8,95E-16 | 7,02E-15 |
| *NUP58* | 9,83E-11 | 5,09E-10 | 1,9 |  | 0,9 | 2,85E-05 | 8,16E-05 |
| *CARD8-AS1* | 1,01E-10 | 5,23E-10 | 14,7 |  | 7,1 | 5,02E-10 | 2,51E-09 |
| *MTA3* | 1,03E-10 | 5,30E-10 | -2,5 |  | -2,4 | 3,48E-02 | 5,58E-02 |
| *VSTM4* | 1,03E-10 | 5,31E-10 | -4,4 |  | -4,2 | 5,35E-07 | 1,92E-06 |
| *TPM3* | 1,03E-10 | 5,32E-10 | 1,7 |  | 1,3 | 4,99E-07 | 1,80E-06 |
| *ACACA* | 1,08E-10 | 5,55E-10 | -2,3 |  | -8,0 | 1,73E-44 | 5,42E-43 |
| *TRIM44* | 1,08E-10 | 5,57E-10 | 1,7 |  | -2,3 | 1,35E-02 | 2,39E-02 |
| *ANKZF1* | 1,09E-10 | 5,62E-10 | 2,4 |  | 2,4 | 1,05E-10 | 5,56E-10 |
| *CUTA* | 1,11E-10 | 5,71E-10 | -1,9 |  | -2,9 | 8,20E-08 | 3,26E-07 |
| *TRUB1* | 1,12E-10 | 5,77E-10 | -2,5 |  | -2,4 | 6,72E-02 | 1,01E-01 |
| *RPS7* | 1,17E-10 | 6,03E-10 | -2,1 |  | -4,6 | 5,69E-44 | 1,75E-42 |
| *NAALADL2* | 1,18E-10 | 6,06E-10 | -4,3 |  | -9,8 | 9,89E-15 | 7,19E-14 |
| *APLF* | 1,19E-10 | 6,13E-10 | 4,7 |  | -2,0 | 9,67E-01 | 9,75E-01 |
| *RPS4X* | 1,19E-10 | 6,15E-10 | -2,1 |  | -5,7 | 2,99E-46 | 9,98E-45 |
| *GPX4* | 1,19E-10 | 6,15E-10 | -2,2 |  | -4,2 | 1,50E-17 | 1,36E-16 |
| *NFS1* | 1,22E-10 | 6,27E-10 | -3,0 |  | -2,9 | 1,98E-04 | 4,90E-04 |
| *TRIP12* | 1,25E-10 | 6,42E-10 | 1,7 |  | 0,2 | 1,35E-02 | 2,38E-02 |
| *MFN1* | 1,26E-10 | 6,49E-10 | -2,0 |  | -2,7 | 5,05E-03 | 9,74E-03 |
| *JUND* | 1,29E-10 | 6,60E-10 | 2,1 |  | 1,8 | 8,20E-19 | 8,11E-18 |
| *TOP2A* | 1,33E-10 | 6,82E-10 | -2,8 |  | -2,7 | 1,05E-01 | 1,49E-01 |
| *MTFR1L* | 1,34E-10 | 6,88E-10 | -2,0 |  | -2,3 | 4,02E-03 | 7,91E-03 |
| *RSPRY1* | 1,36E-10 | 6,98E-10 | 1,7 |  | 0,5 | 4,70E-04 | 1,09E-03 |
| *TRAPPC10* | 1,37E-10 | 7,01E-10 | 1,9 |  | 1,2 | 1,27E-07 | 4,94E-07 |
| *LBHD1* | 1,37E-10 | 7,02E-10 | 7,0 |  | 0,4 | 4,43E-01 | 5,15E-01 |
| *RPA2* | 1,37E-10 | 7,02E-10 | 2,6 |  | 0,7 | 1,11E-03 | 2,43E-03 |
| *C1D* | 1,39E-10 | 7,11E-10 | -4,3 |  | -2,9 | 3,37E-02 | 5,43E-02 |
| *TCFL5* | 1,42E-10 | 7,28E-10 | -2,9 |  | -4,9 | 7,99E-14 | 5,45E-13 |
| *WASHC4* | 1,43E-10 | 7,32E-10 | 2,0 |  | 0,6 | 1,06E-02 | 1,92E-02 |
| *TBK1* | 1,44E-10 | 7,36E-10 | 2,4 |  | 0,6 | 2,13E-02 | 3,61E-02 |
| *ARF4* | 1,45E-10 | 7,40E-10 | 1,8 |  | 0,3 | 6,44E-02 | 9,69E-02 |
| *CCM2* | 1,49E-10 | 7,60E-10 | 1,9 |  | 1,6 | 7,58E-08 | 3,03E-07 |
| *PIGM* | 1,50E-10 | 7,65E-10 | -3,0 |  | -3,6 | 9,57E-11 | 5,10E-10 |
| *MTMR9LP* | 1,50E-10 | 7,67E-10 | 12,6 |  | 3,5 | 3,81E-07 | 1,40E-06 |
| *LUCAT1* | 1,53E-10 | 7,80E-10 | 8,9 |  | 6,6 | 3,03E-09 | 1,40E-08 |
| *ATP5F1A* | 1,54E-10 | 7,83E-10 | -1,8 |  | -2,6 | 5,81E-09 | 2,60E-08 |
| *KDM2A* | 1,54E-10 | 7,84E-10 | 2,3 |  | 1,7 | 1,58E-18 | 1,54E-17 |
| *MDM2* | 1,57E-10 | 8,01E-10 | -1,9 |  | -2,7 | 1,01E-03 | 2,22E-03 |
| *TMEM208* | 1,59E-10 | 8,09E-10 | 2,6 |  | 1,0 | 6,11E-06 | 1,92E-05 |
| *SAMD8* | 1,61E-10 | 8,20E-10 | 2,0 |  | 0,3 | 1,95E-01 | 2,56E-01 |
| *TTYH3* | 1,62E-10 | 8,22E-10 | -3,0 |  | -3,3 | 6,75E-05 | 1,81E-04 |
| *DAZAP2* | 1,63E-10 | 8,29E-10 | 1,8 |  | 0,8 | 1,31E-08 | 5,65E-08 |
| *ZFP36L1* | 1,64E-10 | 8,35E-10 | 3,0 |  | 3,1 | 9,59E-11 | 5,11E-10 |
| *PID1* | 1,65E-10 | 8,36E-10 | -3,4 |  | 12,8 | 3,35E-20 | 3,59E-19 |
| *PGM1* | 1,65E-10 | 8,40E-10 | 2,2 |  | 0,8 | 3,14E-03 | 6,33E-03 |
| *NOL6* | 1,66E-10 | 8,42E-10 | -2,2 |  | 0,0 | 8,44E-01 | 8,75E-01 |
| *MAP3K2* | 1,68E-10 | 8,52E-10 | 2,3 |  | -2,2 | 2,64E-01 | 3,32E-01 |
| *ABCB9* | 1,68E-10 | 8,52E-10 | -2,9 |  | -4,1 | 9,10E-09 | 3,99E-08 |
| *CACNA1C* | 1,69E-10 | 8,54E-10 | -6,1 |  | -44,9 | 3,55E-18 | 3,35E-17 |
| *PABPC4* | 1,71E-10 | 8,68E-10 | -2,3 |  | -7,5 | 3,13E-44 | 9,69E-43 |
| *ANKIB1* | 1,73E-10 | 8,74E-10 | 1,9 |  | 1,5 | 3,53E-15 | 2,64E-14 |
| *GATC* | 1,74E-10 | 8,82E-10 | -2,0 |  | -2,2 | 1,07E-01 | 1,51E-01 |
| *MIR23AHG* | 1,75E-10 | 8,86E-10 | 2,5 |  | -2,0 | 9,51E-01 | 9,62E-01 |
| *CYFIP2* | 1,76E-10 | 8,92E-10 | -2,6 |  | -3,1 | 9,36E-03 | 1,71E-02 |
| *GAK* | 1,78E-10 | 9,01E-10 | 2,0 |  | 1,0 | 1,28E-11 | 7,46E-11 |
| *PRRG4* | 1,79E-10 | 9,04E-10 | 14,2 |  | 27,9 | 1,05E-22 | 1,29E-21 |
| *RPS8* | 1,79E-10 | 9,05E-10 | -2,2 |  | -6,6 | 2,53E-100 | 2,71E-98 |
| *NALCN* | 1,81E-10 | 9,13E-10 | -75,5 |  | -49,5 | 2,46E-46 | 8,24E-45 |
| *GTF2B* | 1,81E-10 | 9,13E-10 | 2,1 |  | 1,7 | 2,37E-14 | 1,67E-13 |
| *MED20* | 1,81E-10 | 9,14E-10 | -2,8 |  | -3,4 | 4,61E-04 | 1,08E-03 |
| *GOLT1B* | 1,82E-10 | 9,18E-10 | 2,1 |  | 0,7 | 2,36E-02 | 3,96E-02 |
| *PDK2* | 1,84E-10 | 9,28E-10 | -3,0 |  | -3,1 | 4,21E-06 | 1,35E-05 |
| *JOSD1* | 1,98E-10 | 9,98E-10 | 2,0 |  | 0,3 | 1,98E-03 | 4,15E-03 |
| *ENTPD4* | 1,98E-10 | 9,99E-10 | -1,6 |  | -2,3 | 3,11E-02 | 5,05E-02 |
| *NACA* | 2,00E-10 | 1,01E-09 | -2,0 |  | -4,1 | 1,37E-21 | 1,59E-20 |
| *RPL31* | 2,03E-10 | 1,02E-09 | -2,1 |  | -4,9 | 9,98E-45 | 3,14E-43 |
| *DPY19L4* | 2,05E-10 | 1,03E-09 | -2,1 |  | -3,6 | 1,29E-06 | 4,43E-06 |
| *PRXL2C* | 2,07E-10 | 1,04E-09 | 2,4 |  | 1,4 | 2,29E-07 | 8,61E-07 |
| *ARL8A* | 2,11E-10 | 1,06E-09 | 2,4 |  | 1,7 | 1,43E-14 | 1,03E-13 |
| *MGME1* | 2,12E-10 | 1,06E-09 | 2,3 |  | 0,9 | 9,22E-06 | 2,84E-05 |
| *PHLDA1* | 2,17E-10 | 1,09E-09 | 2,2 |  | 0,4 | 2,83E-04 | 6,83E-04 |
| *TMEM98* | 2,19E-10 | 1,10E-09 | -2,0 |  | -4,1 | 2,68E-14 | 1,89E-13 |
| *LRIG1* | 2,25E-10 | 1,13E-09 | 2,8 |  | 0,1 | 5,94E-01 | 6,57E-01 |
| *KIF1B* | 2,27E-10 | 1,14E-09 | 2,3 |  | 0,3 | 2,03E-02 | 3,46E-02 |
| *NES* | 2,32E-10 | 1,16E-09 | 3,0 |  | -3,1 | 5,02E-04 | 1,16E-03 |
| *FH* | 2,35E-10 | 1,18E-09 | -2,1 |  | -4,0 | 1,65E-13 | 1,10E-12 |
| *IL13RA1* | 2,38E-10 | 1,19E-09 | 2,0 |  | -2,6 | 3,57E-03 | 7,10E-03 |
| *ADAMTS1* | 2,43E-10 | 1,22E-09 | 1,8 |  | 0,4 | 3,76E-01 | 4,49E-01 |
| *EFNA5* | 2,47E-10 | 1,24E-09 | -5,7 |  | -24,1 | 3,40E-20 | 3,63E-19 |
| *ANKH* | 2,50E-10 | 1,25E-09 | -3,4 |  | -17,0 | 2,08E-46 | 6,99E-45 |
| *RPS15A* | 2,52E-10 | 1,26E-09 | -2,2 |  | -5,1 | 1,35E-30 | 2,43E-29 |
| *TCF19* | 2,55E-10 | 1,28E-09 | 2,4 |  | 1,2 | 4,61E-04 | 1,07E-03 |
| *ABI1* | 2,56E-10 | 1,28E-09 | 1,8 |  | 1,7 | 9,90E-24 | 1,28E-22 |
| *COX7A2L* | 2,59E-10 | 1,29E-09 | -1,9 |  | -3,5 | 3,71E-12 | 2,25E-11 |
| *AK1* | 2,61E-10 | 1,30E-09 | -4,8 |  | -5,7 | 2,83E-18 | 2,70E-17 |
| *KRT34* | 2,62E-10 | 1,31E-09 | 15,4 |  | 8,7 | 1,06E-07 | 4,16E-07 |
| *AHNAK2* | 2,62E-10 | 1,31E-09 | -4,1 |  | -4,9 | 1,94E-08 | 8,24E-08 |
| *SMPD2* | 2,66E-10 | 1,33E-09 | 3,5 |  | 1,2 | 1,95E-05 | 5,72E-05 |
| *KIRREL1* | 2,67E-10 | 1,33E-09 | 1,9 |  | 0,1 | 5,55E-01 | 6,22E-01 |
| *SHC4* | 2,67E-10 | 1,33E-09 | -7,4 |  | 0,5 | 2,30E-01 | 2,95E-01 |
| *FBLN2* | 2,68E-10 | 1,33E-09 | -6,2 |  | -10,8 | 6,07E-11 | 3,28E-10 |
| *OMA1* | 2,70E-10 | 1,34E-09 | -4,0 |  | -7,0 | 1,08E-10 | 5,70E-10 |
| *ACADSB* | 2,79E-10 | 1,39E-09 | -3,2 |  | -3,1 | 8,77E-08 | 3,47E-07 |
| *UBALD2* | 2,79E-10 | 1,39E-09 | 4,0 |  | 4,1 | 1,94E-13 | 1,29E-12 |
| *SATB1* | 2,80E-10 | 1,39E-09 | -3,7 |  | -3,1 | 2,85E-06 | 9,33E-06 |
| *ARSL* | 2,88E-10 | 1,43E-09 | -8,2 |  | -11,9 | 3,81E-05 | 1,07E-04 |
| *GPR176* | 2,90E-10 | 1,44E-09 | 1,8 |  | 2,2 | 1,73E-10 | 9,05E-10 |
| *RYK* | 2,94E-10 | 1,46E-09 | -1,8 |  | -3,3 | 1,85E-13 | 1,23E-12 |
| *KLF3* | 3,01E-10 | 1,50E-09 | 2,1 |  | 0,7 | 3,46E-03 | 6,89E-03 |
| *GNA13* | 3,04E-10 | 1,51E-09 | 2,1 |  | 0,9 | 8,12E-05 | 2,14E-04 |
| *ITPRIP* | 3,06E-10 | 1,52E-09 | 3,8 |  | 4,8 | 2,57E-15 | 1,94E-14 |
| *CROT* | 3,07E-10 | 1,52E-09 | -2,6 |  | -2,6 | 5,56E-03 | 1,07E-02 |
| *MMP1* | 3,11E-10 | 1,54E-09 | 2,1 |  | 47,2 | 1,32E-05 | 3,95E-05 |
| *PARP1* | 3,18E-10 | 1,57E-09 | -2,0 |  | -2,9 | 3,66E-09 | 1,67E-08 |
| *CAVIN2* | 3,28E-10 | 1,62E-09 | -112,0 |  | -22,8 | 9,66E-09 | 4,24E-08 |
| *NDUFV1* | 3,42E-10 | 1,69E-09 | -1,8 |  | -2,7 | 3,09E-05 | 8,80E-05 |
| *INAFM2* | 3,50E-10 | 1,73E-09 | -1,9 |  | -2,6 | 7,58E-02 | 1,12E-01 |
| *ATG16L2* | 3,53E-10 | 1,74E-09 | 5,0 |  | 1,9 | 5,09E-05 | 1,39E-04 |
| *EXTL2* | 3,71E-10 | 1,83E-09 | 2,8 |  | -3,1 | 3,76E-04 | 8,89E-04 |
| *SLC39A6* | 3,78E-10 | 1,86E-09 | 2,0 |  | 0,5 | 3,43E-04 | 8,16E-04 |
| *SRPK2* | 3,82E-10 | 1,89E-09 | -2,3 |  | -2,9 | 5,89E-10 | 2,93E-09 |
| *TIMM8B* | 3,83E-10 | 1,89E-09 | -2,4 |  | -2,4 | 3,80E-02 | 6,03E-02 |
| *RANBP10* | 3,91E-10 | 1,93E-09 | 3,2 |  | 1,2 | 6,82E-06 | 2,13E-05 |
| *TEAD4* | 3,93E-10 | 1,94E-09 | 3,1 |  | 0,6 | 7,06E-02 | 1,05E-01 |
| *DMPK* | 4,02E-10 | 1,98E-09 | -2,9 |  | -4,9 | 1,29E-12 | 8,07E-12 |
| *STRBP* | 4,04E-10 | 1,99E-09 | -3,8 |  | -4,9 | 4,62E-06 | 1,47E-05 |
| *RAC3* | 4,09E-10 | 2,01E-09 | -4,4 |  | -2,9 | 2,78E-01 | 3,47E-01 |
| *CLDN23* | 4,12E-10 | 2,02E-09 | 55,5 |  | 7,5 | 1,25E-02 | 2,23E-02 |
| *FBXO44* | 4,17E-10 | 2,05E-09 | -2,8 |  | -2,7 | 6,32E-03 | 1,20E-02 |
| *FAM171A1* | 4,25E-10 | 2,09E-09 | -2,0 |  | -2,2 | 3,81E-01 | 4,53E-01 |
| *TMX1* | 4,45E-10 | 2,18E-09 | 2,5 |  | 4,7 | 2,17E-35 | 4,95E-34 |
| *GSTM3* | 4,50E-10 | 2,21E-09 | -2,5 |  | -4,8 | 1,38E-16 | 1,16E-15 |
| *CNOT9* | 4,54E-10 | 2,22E-09 | 2,1 |  | 0,6 | 1,55E-04 | 3,91E-04 |
| *RNF41* | 4,54E-10 | 2,22E-09 | -2,1 |  | -2,8 | 5,70E-09 | 2,56E-08 |
| *MAPK9* | 4,67E-10 | 2,29E-09 | -2,1 |  | -2,1 | 3,30E-01 | 4,02E-01 |
| *CREM* | 4,69E-10 | 2,30E-09 | 2,2 |  | 4,1 | 2,30E-27 | 3,64E-26 |
| *CANT1* | 4,74E-10 | 2,32E-09 | 2,0 |  | 2,7 | 6,00E-23 | 7,46E-22 |
| *FBXL4* | 4,86E-10 | 2,38E-09 | 2,1 |  | -2,1 | 2,48E-01 | 3,14E-01 |
| *FBXL6* | 4,88E-10 | 2,39E-09 | 4,3 |  | 0,9 | 2,38E-02 | 3,99E-02 |
| *ANXA5* | 4,89E-10 | 2,39E-09 | 1,8 |  | 0,7 | 2,13E-03 | 4,44E-03 |
| *BCAT1* | 4,93E-10 | 2,41E-09 | -1,8 |  | -3,2 | 2,59E-04 | 6,28E-04 |
| *UBE4B* | 5,01E-10 | 2,45E-09 | -2,1 |  | -6,1 | 3,57E-38 | 8,99E-37 |
| *CHCHD6* | 5,03E-10 | 2,46E-09 | -3,2 |  | -4,5 | 8,17E-08 | 3,25E-07 |
| *MTA1* | 5,15E-10 | 2,52E-09 | -2,4 |  | -3,0 | 1,37E-06 | 4,67E-06 |
| *PAPPA* | 5,24E-10 | 2,56E-09 | -2,5 |  | -8,3 | 3,30E-29 | 5,63E-28 |
| *CFAP251* | 5,25E-10 | 2,56E-09 | -2,5 |  | 22,7 | 1,20E-17 | 1,09E-16 |
| *PLAAT3* | 5,25E-10 | 2,56E-09 | 11,1 |  | 3,5 | 9,22E-07 | 3,22E-06 |
| *MSN* | 5,30E-10 | 2,58E-09 | 1,7 |  | 0,6 | 1,06E-03 | 2,32E-03 |
| *GASK1B* | 5,34E-10 | 2,60E-09 | -33,7 |  | -7,8 | 3,89E-04 | 9,18E-04 |
| *COL12A1* | 5,42E-10 | 2,64E-09 | -2,3 |  | -141,1 | 4,87E-31 | 9,04E-30 |
| *FLNA* | 5,48E-10 | 2,67E-09 | 2,1 |  | 0,2 | 4,06E-01 | 4,77E-01 |
| *FHL3* | 5,50E-10 | 2,68E-09 | -3,6 |  | 0,0 | 8,47E-01 | 8,78E-01 |
| *SLMAP* | 5,51E-10 | 2,68E-09 | 2,0 |  | 0,1 | 4,75E-01 | 5,46E-01 |
| *RPL27* | 5,56E-10 | 2,70E-09 | -2,0 |  | -4,4 | 5,58E-27 | 8,64E-26 |
| *RC3H2* | 5,63E-10 | 2,74E-09 | 2,0 |  | -2,2 | 3,33E-01 | 4,05E-01 |
| *PRICKLE3* | 5,78E-10 | 2,81E-09 | 4,9 |  | 1,1 | 3,26E-03 | 6,54E-03 |
| *SRPRB* | 6,58E-10 | 3,19E-09 | -2,3 |  | 0,1 | 7,02E-01 | 7,53E-01 |
| *ADAMTS12* | 6,79E-10 | 3,30E-09 | -2,0 |  | 0,4 | 1,04E-01 | 1,48E-01 |
| *MLEC* | 6,93E-10 | 3,36E-09 | -1,7 |  | -6,6 | 1,70E-24 | 2,28E-23 |
| *KCNK6* | 7,01E-10 | 3,40E-09 | 5,8 |  | -2,5 | 1,13E-01 | 1,59E-01 |
| *SHROOM3* | 7,07E-10 | 3,42E-09 | -3,5 |  | -2,8 | 7,49E-02 | 1,11E-01 |
| *SCARB1* | 7,22E-10 | 3,50E-09 | -3,5 |  | -4,0 | 1,37E-05 | 4,10E-05 |
| *SLC7A11* | 7,35E-10 | 3,56E-09 | -10,0 |  | -5,4 | 1,74E-12 | 1,08E-11 |
| *CNIH3* | 7,45E-10 | 3,60E-09 | -2,5 |  | -7,8 | 3,25E-36 | 7,61E-35 |
| *LRRN3* | 7,59E-10 | 3,67E-09 | 73,1 |  | 2,9 | 2,04E-04 | 5,03E-04 |
| *CPEB1* | 7,81E-10 | 3,78E-09 | -6,7 |  | -6,8 | 9,00E-06 | 2,77E-05 |
| *EPB41L4A-AS1* | 7,82E-10 | 3,78E-09 | -4,4 |  | -4,4 | 2,51E-10 | 1,29E-09 |
| *RASSF3* | 7,94E-10 | 3,84E-09 | 2,1 |  | 1,2 | 5,07E-09 | 2,29E-08 |
| *ADRM1* | 8,16E-10 | 3,94E-09 | 1,7 |  | 1,8 | 2,36E-08 | 9,94E-08 |
| *P4HA1* | 8,28E-10 | 4,00E-09 | 2,3 |  | 2,3 | 4,36E-06 | 1,39E-05 |
| *MTR* | 8,32E-10 | 4,01E-09 | 1,9 |  | -2,0 | 9,35E-01 | 9,50E-01 |
| *SIRT2* | 8,39E-10 | 4,05E-09 | -1,9 |  | -2,7 | 1,90E-05 | 5,57E-05 |
| *CADPS2* | 8,49E-10 | 4,10E-09 | 30,1 |  | -2,4 | 2,23E-01 | 2,86E-01 |
| *TNS1* | 8,54E-10 | 4,12E-09 | 7,2 |  | 2,6 | 4,21E-14 | 2,92E-13 |
| *BAIAP2-DT* | 8,57E-10 | 4,13E-09 | -4,6 |  | -3,7 | 8,61E-09 | 3,79E-08 |
| *HYOU1* | 8,66E-10 | 4,17E-09 | 1,9 |  | 0,2 | 3,11E-01 | 3,82E-01 |
| *SMCHD1* | 9,39E-10 | 4,52E-09 | 1,8 |  | 0,5 | 3,53E-02 | 5,66E-02 |
| *WLS* | 9,51E-10 | 4,58E-09 | -2,0 |  | -4,1 | 9,72E-12 | 5,70E-11 |
| *EIF1AY* | 9,58E-10 | 4,61E-09 | 2,9 |  | 2,5 | 4,73E-16 | 3,79E-15 |
| *RAP2A* | 9,62E-10 | 4,62E-09 | 2,5 |  | 0,1 | 5,31E-01 | 5,99E-01 |
| *GPR107* | 9,62E-10 | 4,62E-09 | 2,0 |  | 0,2 | 2,21E-01 | 2,84E-01 |
| *SMARCD2* | 9,70E-10 | 4,66E-09 | -1,9 |  | -2,9 | 5,08E-06 | 1,61E-05 |
| *TET3* | 9,86E-10 | 4,73E-09 | 5,3 |  | 1,4 | 1,64E-04 | 4,12E-04 |
| *FAM167B* | 1,02E-09 | 4,87E-09 | 28,2 |  | 6,7 | 6,30E-17 | 5,40E-16 |
| *FZD8* | 1,05E-09 | 5,05E-09 | -3,7 |  | -3,4 | 5,65E-03 | 1,08E-02 |
| *NRSN2* | 1,05E-09 | 5,05E-09 | 2,2 |  | 2,0 | 5,60E-17 | 4,82E-16 |
| *CCT7* | 1,06E-09 | 5,07E-09 | -1,9 |  | -2,4 | 4,81E-03 | 9,32E-03 |
| *EXOC6* | 1,08E-09 | 5,18E-09 | 3,2 |  | 6,9 | 1,26E-21 | 1,46E-20 |
| *METTL8* | 1,08E-09 | 5,19E-09 | -2,1 |  | -3,1 | 1,22E-06 | 4,20E-06 |
| *RSL1D1* | 1,11E-09 | 5,29E-09 | -1,7 |  | -2,3 | 1,26E-03 | 2,72E-03 |
| *MSRB2* | 1,11E-09 | 5,29E-09 | 3,0 |  | 0,9 | 1,01E-05 | 3,09E-05 |
| *RPL30* | 1,11E-09 | 5,30E-09 | -2,0 |  | -4,0 | 3,21E-18 | 3,06E-17 |
| *MMP15* | 1,11E-09 | 5,31E-09 | -10,0 |  | -5,3 | 7,01E-10 | 3,46E-09 |
| *RHEBL1* | 1,12E-09 | 5,35E-09 | 126,8 |  | 21,9 | 1,55E-15 | 1,19E-14 |
| *PRADC1* | 1,13E-09 | 5,39E-09 | -3,0 |  | -11,7 | 1,37E-16 | 1,15E-15 |
| *ZFP36* | 1,14E-09 | 5,46E-09 | 4,7 |  | 2,6 | 3,28E-08 | 1,36E-07 |
| *IFT57* | 1,15E-09 | 5,47E-09 | -2,2 |  | -4,5 | 2,16E-14 | 1,53E-13 |
| *FCF1* | 1,15E-09 | 5,48E-09 | -2,1 |  | -2,6 | 6,30E-04 | 1,43E-03 |
| *RPS28* | 1,15E-09 | 5,49E-09 | -2,0 |  | -3,8 | 1,19E-12 | 7,50E-12 |
| *FAT4* | 1,16E-09 | 5,52E-09 | -3,3 |  | -3,7 | 7,35E-12 | 4,35E-11 |
| *KIAA1191* | 1,16E-09 | 5,53E-09 | 1,8 |  | 1,0 | 5,31E-06 | 1,68E-05 |
| *TARBP1* | 1,17E-09 | 5,59E-09 | -2,6 |  | -4,1 | 8,97E-10 | 4,39E-09 |
| *PDZD8* | 1,19E-09 | 5,65E-09 | -2,2 |  | -5,6 | 1,13E-18 | 1,11E-17 |
| *USP9X* | 1,20E-09 | 5,72E-09 | 1,8 |  | 0,0 | 9,36E-01 | 9,51E-01 |
| *KIAA0513* | 1,21E-09 | 5,78E-09 | -3,3 |  | -2,4 | 5,31E-02 | 8,15E-02 |
| *WWC3* | 1,22E-09 | 5,80E-09 | 1,9 |  | 0,9 | 5,16E-03 | 9,95E-03 |
| *NUP62* | 1,24E-09 | 5,87E-09 | 1,8 |  | 4,6 | 2,86E-36 | 6,70E-35 |
| *GPR153* | 1,25E-09 | 5,94E-09 | -6,3 |  | -8,4 | 6,17E-05 | 1,66E-04 |
| *KIF26B* | 1,26E-09 | 5,97E-09 | -24,5 |  | -69,2 | 4,84E-05 | 1,33E-04 |
| *TRPC4* | 1,27E-09 | 6,02E-09 | 2,6 |  | 1,6 | 9,78E-08 | 3,85E-07 |
| *VCPIP1* | 1,27E-09 | 6,03E-09 | 2,6 |  | 2,2 | 1,25E-09 | 6,01E-09 |
| *THBS1* | 1,28E-09 | 6,07E-09 | -3,9 |  | -16,0 | 3,15E-84 | 2,46E-82 |
| *CITED4* | 1,29E-09 | 6,13E-09 | 5,5 |  | 0,4 | 2,45E-01 | 3,11E-01 |
| *CRYBG3* | 1,30E-09 | 6,16E-09 | 2,3 |  | -2,3 | 1,12E-01 | 1,58E-01 |
| *GLS* | 1,30E-09 | 6,17E-09 | -2,0 |  | -5,0 | 1,01E-12 | 6,34E-12 |
| *B3GALT4* | 1,33E-09 | 6,31E-09 | 13,2 |  | 12,0 | 2,20E-12 | 1,35E-11 |
| *CYBC1* | 1,34E-09 | 6,34E-09 | 2,0 |  | 1,4 | 3,13E-11 | 1,74E-10 |
| *DHDDS* | 1,35E-09 | 6,38E-09 | 1,9 |  | -2,1 | 6,79E-01 | 7,33E-01 |
| *GDI1* | 1,35E-09 | 6,41E-09 | 1,8 |  | 1,1 | 7,97E-08 | 3,18E-07 |
| *ZNF562* | 1,37E-09 | 6,50E-09 | 1,9 |  | 0,7 | 4,09E-07 | 1,49E-06 |
| *PPM1F* | 1,37E-09 | 6,50E-09 | -2,1 |  | -2,8 | 6,91E-04 | 1,56E-03 |
| *SECISBP2L* | 1,38E-09 | 6,53E-09 | 2,0 |  | 1,1 | 1,57E-12 | 9,80E-12 |
| *PRKAA2* | 1,38E-09 | 6,54E-09 | 16,1 |  | 2,3 | 5,28E-06 | 1,67E-05 |
| *ATG13* | 1,39E-09 | 6,55E-09 | 1,8 |  | 1,0 | 3,55E-22 | 4,21E-21 |
| *WFDC21P* | 1,39E-09 | 6,57E-09 | -49,0 |  | -2,0 | 9,53E-01 | 9,63E-01 |
| *HPS3* | 1,40E-09 | 6,58E-09 | 2,0 |  | 3,1 | 3,18E-33 | 6,61E-32 |
| *USP48* | 1,40E-09 | 6,61E-09 | 1,8 |  | 0,0 | 8,62E-01 | 8,90E-01 |
| *CSRNP2* | 1,42E-09 | 6,70E-09 | 2,2 |  | 1,5 | 1,10E-10 | 5,81E-10 |
| *SLC25A1* | 1,43E-09 | 6,72E-09 | -2,0 |  | 0,0 | 9,80E-01 | 9,84E-01 |
| *ZSWIM8* | 1,51E-09 | 7,08E-09 | 2,1 |  | 0,6 | 3,38E-04 | 8,06E-04 |
| *GNAI1* | 1,51E-09 | 7,10E-09 | 2,4 |  | 0,6 | 2,20E-03 | 4,56E-03 |
| *UQCRQ* | 1,52E-09 | 7,15E-09 | -2,1 |  | -3,9 | 8,18E-10 | 4,01E-09 |
| *COTL1* | 1,52E-09 | 7,15E-09 | -1,6 |  | -4,8 | 1,61E-15 | 1,24E-14 |
| *RPS12* | 1,52E-09 | 7,15E-09 | -2,1 |  | -5,3 | 1,12E-36 | 2,66E-35 |
| *GEMIN5* | 1,55E-09 | 7,30E-09 | -2,0 |  | -2,6 | 5,92E-04 | 1,35E-03 |
| *LMBR1L* | 1,57E-09 | 7,38E-09 | 3,0 |  | 1,8 | 4,70E-08 | 1,92E-07 |
| *DENND2D* | 1,60E-09 | 7,52E-09 | 10,0 |  | 9,9 | 1,89E-17 | 1,69E-16 |
| *RPS4Y1* | 1,63E-09 | 7,66E-09 | -2,2 |  | -5,6 | 1,53E-39 | 4,05E-38 |
| *UQCC2* | 1,64E-09 | 7,71E-09 | -2,5 |  | -3,4 | 9,73E-09 | 4,26E-08 |
| *HADH* | 1,66E-09 | 7,77E-09 | -4,2 |  | -8,7 | 1,41E-09 | 6,76E-09 |
| *SLC37A3* | 1,67E-09 | 7,84E-09 | 2,1 |  | 0,4 | 6,19E-02 | 9,34E-02 |
| *SECISBP2* | 1,73E-09 | 8,09E-09 | 2,2 |  | 0,4 | 6,36E-04 | 1,44E-03 |
| *FBXO7* | 1,76E-09 | 8,25E-09 | 1,8 |  | 1,9 | 1,17E-26 | 1,78E-25 |
| *ARL4A* | 1,76E-09 | 8,25E-09 | 3,0 |  | 4,0 | 3,34E-09 | 1,54E-08 |
| *LPIN3* | 1,81E-09 | 8,49E-09 | 7,0 |  | 1,0 | 1,68E-03 | 3,55E-03 |
| *ELOB* | 1,83E-09 | 8,58E-09 | -2,0 |  | -2,8 | 5,26E-05 | 1,44E-04 |
| *QSOX2* | 1,85E-09 | 8,65E-09 | 2,1 |  | 0,0 | 8,33E-01 | 8,66E-01 |
| *BID* | 1,88E-09 | 8,78E-09 | 2,2 |  | 5,1 | 1,99E-15 | 1,51E-14 |
| *UBQLN2* | 1,90E-09 | 8,86E-09 | 2,9 |  | 1,4 | 4,14E-11 | 2,27E-10 |
| *CEP135* | 1,90E-09 | 8,87E-09 | 3,0 |  | 1,3 | 2,67E-04 | 6,45E-04 |
| *PKNOX1* | 1,91E-09 | 8,93E-09 | 2,2 |  | 1,4 | 1,84E-09 | 8,70E-09 |
| *XPO6* | 1,92E-09 | 8,96E-09 | 1,7 |  | 2,5 | 1,06E-27 | 1,69E-26 |
| *RAB7B* | 1,92E-09 | 8,97E-09 | -29,8 |  | -65,9 | 1,83E-46 | 6,19E-45 |
| *CCNC* | 1,94E-09 | 9,03E-09 | -1,9 |  | -3,1 | 6,64E-08 | 2,67E-07 |
| *MTX3* | 1,94E-09 | 9,05E-09 | -3,1 |  | -4,6 | 1,45E-07 | 5,59E-07 |
| *PSMA3* | 1,97E-09 | 9,16E-09 | 1,9 |  | 0,7 | 2,59E-06 | 8,50E-06 |
| *ZNF92* | 1,98E-09 | 9,22E-09 | 3,5 |  | 8,6 | 1,59E-20 | 1,74E-19 |
| *SNX24* | 1,98E-09 | 9,23E-09 | 2,2 |  | 1,0 | 1,03E-04 | 2,68E-04 |
| *SLC9A7* | 1,99E-09 | 9,24E-09 | -2,0 |  | -2,5 | 3,54E-02 | 5,67E-02 |
| *CHML* | 2,11E-09 | 9,84E-09 | -2,5 |  | -7,4 | 4,97E-26 | 7,30E-25 |
| *AADAT* | 2,14E-09 | 9,94E-09 | -4,6 |  | -10,8 | 1,77E-15 | 1,36E-14 |
| *KLHL42* | 2,16E-09 | 1,01E-08 | 1,8 |  | -2,1 | 5,66E-01 | 6,32E-01 |
| *HTR7P1* | 2,18E-09 | 1,01E-08 | -3,1 |  | -4,6 | 3,37E-10 | 1,71E-09 |
| *OXA1L* | 2,18E-09 | 1,02E-08 | -1,8 |  | -2,8 | 3,22E-06 | 1,05E-05 |
| *SOCS7* | 2,20E-09 | 1,02E-08 | -2,8 |  | -6,1 | 1,00E-18 | 9,88E-18 |
| *NDUFS6* | 2,22E-09 | 1,03E-08 | -2,5 |  | -2,9 | 1,87E-04 | 4,65E-04 |
| *CDH2* | 2,24E-09 | 1,04E-08 | 1,8 |  | 1,1 | 6,01E-03 | 1,15E-02 |
| *ZNF532* | 2,25E-09 | 1,04E-08 | 1,9 |  | 0,7 | 2,24E-04 | 5,49E-04 |
| *TSHZ1* | 2,27E-09 | 1,05E-08 | -5,4 |  | -3,4 | 8,92E-07 | 3,12E-06 |
| *MPZL1* | 2,28E-09 | 1,06E-08 | 1,8 |  | 1,9 | 1,47E-08 | 6,35E-08 |
| *ARFIP2* | 2,34E-09 | 1,08E-08 | -1,9 |  | -2,4 | 2,31E-02 | 3,88E-02 |
| *SCO1* | 2,36E-09 | 1,09E-08 | 1,8 |  | 0,9 | 2,93E-07 | 1,09E-06 |
| *BAG1* | 2,38E-09 | 1,10E-08 | 2,7 |  | 0,9 | 2,02E-04 | 4,98E-04 |
| *TMOD2* | 2,42E-09 | 1,12E-08 | -1,8 |  | -5,4 | 8,03E-14 | 5,47E-13 |
| *RAB15* | 2,42E-09 | 1,12E-08 | -3,4 |  | -2,9 | 3,95E-02 | 6,26E-02 |
| *UQCR10* | 2,46E-09 | 1,13E-08 | -2,0 |  | -2,1 | 4,70E-01 | 5,41E-01 |
| *MMP24OS* | 2,47E-09 | 1,14E-08 | -2,4 |  | -3,7 | 6,75E-09 | 3,01E-08 |
| *GTF3A* | 2,52E-09 | 1,16E-08 | -2,1 |  | -3,2 | 1,13E-05 | 3,44E-05 |
| *RNF146* | 2,53E-09 | 1,17E-08 | 2,0 |  | 0,5 | 1,01E-02 | 1,84E-02 |
| *SPINT2* | 2,54E-09 | 1,17E-08 | 7,2 |  | 12,0 | 1,60E-12 | 9,93E-12 |
| *OPN3* | 2,55E-09 | 1,18E-08 | -2,4 |  | -3,4 | 1,56E-05 | 4,64E-05 |
| *C15orf39* | 2,56E-09 | 1,18E-08 | 5,9 |  | 2,8 | 2,44E-07 | 9,14E-07 |
| *MFAP4* | 2,62E-09 | 1,21E-08 | -8,3 |  | -5,2 | 1,73E-04 | 4,34E-04 |
| *ITM2C* | 2,65E-09 | 1,22E-08 | -1,6 |  | -2,5 | 3,79E-02 | 6,02E-02 |
| *PPP4R4* | 2,66E-09 | 1,23E-08 | 136,6 |  | 38,0 | 1,07E-14 | 7,73E-14 |
| *LYPLA1* | 2,71E-09 | 1,25E-08 | 2,0 |  | 0,2 | 3,64E-01 | 4,36E-01 |
| *SLC39A11* | 2,74E-09 | 1,26E-08 | 2,5 |  | -2,1 | 4,89E-01 | 5,60E-01 |
| *PRELID3B* | 2,81E-09 | 1,29E-08 | 1,9 |  | 0,5 | 1,01E-02 | 1,83E-02 |
| *DDA1* | 2,81E-09 | 1,29E-08 | 1,8 |  | 1,3 | 6,13E-05 | 1,65E-04 |
| *LOXL1-AS1* | 2,87E-09 | 1,32E-08 | -3,0 |  | 0,3 | 3,63E-01 | 4,36E-01 |
| *SRGAP1* | 2,88E-09 | 1,32E-08 | 3,5 |  | 1,7 | 7,10E-11 | 3,82E-10 |
| *DCLK1* | 2,91E-09 | 1,34E-08 | -26,2 |  | -37,1 | 3,34E-29 | 5,68E-28 |
| *GVINP1* | 2,97E-09 | 1,36E-08 | 872,2 |  | 138,4 | 5,59E-54 | 2,22E-52 |
| *SCG5* | 3,02E-09 | 1,39E-08 | 2,7 |  | -4,7 | 1,45E-08 | 6,27E-08 |
| *NRIP3* | 3,04E-09 | 1,39E-08 | 1,9 |  | 3,0 | 2,89E-07 | 1,08E-06 |
| *NUTF2* | 3,05E-09 | 1,40E-08 | -1,6 |  | -2,4 | 9,04E-03 | 1,66E-02 |
| *KIAA1217* | 3,06E-09 | 1,40E-08 | 2,7 |  | 11,1 | 8,09E-67 | 4,32E-65 |
| *DOCK11* | 3,07E-09 | 1,40E-08 | -2,1 |  | -3,2 | 3,27E-05 | 9,26E-05 |
| *FAM98A* | 3,14E-09 | 1,44E-08 | -1,8 |  | -3,0 | 1,19E-06 | 4,11E-06 |
| *LPIN1* | 3,17E-09 | 1,45E-08 | -2,0 |  | -2,5 | 3,11E-02 | 5,05E-02 |
| *CC2D2A* | 3,20E-09 | 1,46E-08 | -2,1 |  | -15,6 | 7,12E-38 | 1,75E-36 |
| *DDX41* | 3,24E-09 | 1,48E-08 | 1,9 |  | 2,0 | 3,80E-06 | 1,22E-05 |
| *PRMT3* | 3,26E-09 | 1,49E-08 | -3,0 |  | -4,9 | 1,44E-06 | 4,88E-06 |
| *ARPC2* | 3,32E-09 | 1,51E-08 | 1,7 |  | 0,6 | 3,62E-03 | 7,19E-03 |
| *GLRX* | 3,32E-09 | 1,52E-08 | 1,8 |  | 1,5 | 6,70E-05 | 1,80E-04 |
| *MFSD11* | 3,33E-09 | 1,52E-08 | 1,9 |  | 0,5 | 2,20E-03 | 4,57E-03 |
| *MSX1* | 3,34E-09 | 1,52E-08 | -2,0 |  | 7,8 | 4,69E-14 | 3,24E-13 |
| *DRAP1* | 3,44E-09 | 1,57E-08 | 1,9 |  | 2,2 | 7,85E-11 | 4,21E-10 |
| *FUT11* | 3,45E-09 | 1,57E-08 | 2,5 |  | 1,0 | 9,80E-05 | 2,56E-04 |
| *TAF13* | 3,46E-09 | 1,58E-08 | 1,9 |  | 0,2 | 3,88E-01 | 4,61E-01 |
| *KBTBD8* | 3,46E-09 | 1,58E-08 | 3,5 |  | 5,0 | 2,01E-15 | 1,52E-14 |
| *MRPL24* | 3,47E-09 | 1,58E-08 | -2,7 |  | -5,1 | 5,45E-10 | 2,72E-09 |
| *ENSG00000238045* | 3,48E-09 | 1,58E-08 | 38,0 |  | 16,0 | 5,22E-15 | 3,87E-14 |
| *AEN* | 3,52E-09 | 1,60E-08 | -1,8 |  | -2,9 | 4,10E-04 | 9,64E-04 |
| *ARSD* | 3,60E-09 | 1,64E-08 | 2,9 |  | 0,6 | 8,08E-04 | 1,80E-03 |
| *ARL2BP* | 3,61E-09 | 1,64E-08 | 1,9 |  | 0,7 | 5,23E-05 | 1,43E-04 |
| *C6orf120* | 3,64E-09 | 1,65E-08 | -1,8 |  | -2,6 | 1,71E-03 | 3,61E-03 |
| *ITGA5* | 3,64E-09 | 1,65E-08 | 1,8 |  | 2,3 | 4,93E-06 | 1,57E-05 |
| *TM9SF2* | 3,66E-09 | 1,66E-08 | 1,9 |  | 0,7 | 1,01E-06 | 3,52E-06 |
| *GALE* | 3,71E-09 | 1,68E-08 | 2,4 |  | 1,5 | 8,72E-06 | 2,69E-05 |
| *MUS81* | 3,80E-09 | 1,73E-08 | 2,0 |  | 0,4 | 3,53E-02 | 5,66E-02 |
| *MRPS26* | 3,81E-09 | 1,73E-08 | -3,0 |  | -2,8 | 5,88E-03 | 1,12E-02 |
| *CCDC167* | 3,83E-09 | 1,74E-08 | -5,2 |  | -5,2 | 6,10E-04 | 1,39E-03 |
| *CNIH1* | 3,87E-09 | 1,75E-08 | -1,8 |  | -3,0 | 4,29E-06 | 1,37E-05 |
| *DAGLB* | 3,88E-09 | 1,76E-08 | -2,7 |  | -2,6 | 7,05E-02 | 1,05E-01 |
| *HDAC9* | 3,96E-09 | 1,79E-08 | 1,8 |  | 15,5 | 1,09E-23 | 1,40E-22 |
| *SLC7A7* | 4,01E-09 | 1,82E-08 | 6,0 |  | 1,0 | 1,77E-01 | 2,36E-01 |
| *C8orf33* | 4,06E-09 | 1,84E-08 | -2,0 |  | -3,5 | 3,25E-16 | 2,64E-15 |
| *TPGS2* | 4,11E-09 | 1,86E-08 | 1,5 |  | 2,6 | 1,21E-22 | 1,47E-21 |
| *EML2* | 4,27E-09 | 1,93E-08 | 2,9 |  | 2,9 | 3,98E-22 | 4,72E-21 |
| *ZCCHC24* | 4,29E-09 | 1,94E-08 | -1,9 |  | -2,7 | 1,02E-08 | 4,48E-08 |
| *MYH9* | 4,44E-09 | 2,01E-08 | 2,3 |  | 0,3 | 8,77E-02 | 1,27E-01 |
| *HPSE* | 4,48E-09 | 2,02E-08 | 16,8 |  | 26,1 | 1,60E-24 | 2,16E-23 |
| *ZNF285* | 4,53E-09 | 2,05E-08 | -9,0 |  | -6,2 | 2,50E-04 | 6,07E-04 |
| *USP33* | 4,55E-09 | 2,05E-08 | 1,7 |  | 1,1 | 9,17E-10 | 4,48E-09 |
| *HSP90B1* | 4,56E-09 | 2,06E-08 | 1,6 |  | 0,7 | 5,68E-04 | 1,30E-03 |
| *MBOAT2* | 4,60E-09 | 2,07E-08 | -1,8 |  | 0,1 | 6,20E-01 | 6,80E-01 |
| *UQCRH* | 4,63E-09 | 2,09E-08 | -1,9 |  | -3,4 | 1,67E-11 | 9,63E-11 |
| *SGSM2* | 4,65E-09 | 2,10E-08 | -1,9 |  | -2,3 | 5,99E-03 | 1,14E-02 |
| *AMPD2* | 4,67E-09 | 2,10E-08 | -2,0 |  | -2,7 | 4,08E-03 | 8,02E-03 |
| *PRRG1* | 4,67E-09 | 2,10E-08 | 2,2 |  | 0,9 | 2,19E-09 | 1,03E-08 |
| *BRI3* | 4,73E-09 | 2,13E-08 | 1,7 |  | 0,7 | 2,60E-05 | 7,48E-05 |
| *BRI3BP* | 4,76E-09 | 2,14E-08 | -2,9 |  | -6,8 | 1,46E-04 | 3,69E-04 |
| *HNRNPA1* | 4,78E-09 | 2,15E-08 | -1,9 |  | -3,0 | 2,58E-17 | 2,28E-16 |
| *BAX* | 4,85E-09 | 2,18E-08 | -1,8 |  | -2,5 | 8,10E-03 | 1,50E-02 |
| *PMS2* | 4,86E-09 | 2,18E-08 | -2,5 |  | -3,1 | 4,08E-04 | 9,61E-04 |
| *MATN2* | 4,87E-09 | 2,19E-08 | -13,2 |  | -18,4 | 4,77E-20 | 5,04E-19 |
| *WDR61* | 4,93E-09 | 2,21E-08 | -2,1 |  | -3,0 | 1,12E-05 | 3,40E-05 |
| *SUOX* | 4,93E-09 | 2,21E-08 | -3,5 |  | 0,1 | 6,41E-01 | 6,99E-01 |
| *ARPC5L* | 4,99E-09 | 2,24E-08 | 1,9 |  | 0,5 | 1,54E-03 | 3,29E-03 |
| *BCL3* | 5,04E-09 | 2,26E-08 | 8,0 |  | 3,2 | 4,66E-09 | 2,11E-08 |
| *CDK5* | 5,05E-09 | 2,26E-08 | -2,4 |  | -2,2 | 1,77E-01 | 2,36E-01 |
| *MFSD4B* | 5,11E-09 | 2,29E-08 | -3,0 |  | -2,9 | 1,98E-03 | 4,14E-03 |
| *PTGR1* | 5,36E-09 | 2,40E-08 | 2,1 |  | -2,2 | 2,35E-01 | 3,00E-01 |
| *TTLL5* | 5,44E-09 | 2,43E-08 | -2,1 |  | -2,9 | 4,72E-04 | 1,10E-03 |
| *DENND5B* | 5,53E-09 | 2,47E-08 | -2,1 |  | 0,1 | 6,73E-01 | 7,28E-01 |
| *TRABD2A* | 5,55E-09 | 2,48E-08 | 4,7 |  | 3,2 | 1,30E-08 | 5,64E-08 |
| *SMAD5* | 5,56E-09 | 2,49E-08 | -1,8 |  | -3,3 | 1,90E-06 | 6,36E-06 |
| *NAPG* | 5,71E-09 | 2,55E-08 | 1,8 |  | 0,2 | 3,32E-01 | 4,04E-01 |
| *LRRC2* | 5,74E-09 | 2,56E-08 | -3,0 |  | -3,3 | 2,18E-02 | 3,69E-02 |
| *FAM102A* | 5,80E-09 | 2,59E-08 | -3,0 |  | -4,0 | 3,41E-10 | 1,73E-09 |
| *SEPTIN4* | 5,96E-09 | 2,66E-08 | 119,4 |  | 121,3 | 2,00E-12 | 1,23E-11 |
| *TOMM22* | 6,00E-09 | 2,68E-08 | -2,0 |  | -2,7 | 7,27E-04 | 1,63E-03 |
| *ERI1* | 6,02E-09 | 2,69E-08 | 1,9 |  | -2,5 | 1,33E-02 | 2,35E-02 |
| *GFOD2* | 6,04E-09 | 2,69E-08 | -2,0 |  | -2,7 | 5,25E-04 | 1,21E-03 |
| *DGCR2* | 6,20E-09 | 2,77E-08 | -1,7 |  | 0,3 | 6,72E-02 | 1,01E-01 |
| *H4C3* | 6,21E-09 | 2,77E-08 | -3,0 |  | -4,5 | 2,86E-06 | 9,34E-06 |
| *ANGPT1* | 6,27E-09 | 2,79E-08 | -3,4 |  | -2,1 | 4,87E-01 | 5,58E-01 |
| *BCL7C* | 6,32E-09 | 2,81E-08 | -2,1 |  | -2,7 | 1,11E-03 | 2,43E-03 |
| *FMNL1* | 6,32E-09 | 2,81E-08 | 99,7 |  | 247,9 | 1,98E-31 | 3,75E-30 |
| *ELP4* | 6,66E-09 | 2,96E-08 | -2,6 |  | -3,0 | 2,89E-03 | 5,86E-03 |
| *ODC1* | 6,72E-09 | 2,99E-08 | -2,1 |  | 0,1 | 6,19E-01 | 6,79E-01 |
| *CNTLN* | 6,77E-09 | 3,01E-08 | 2,8 |  | 0,5 | 3,62E-02 | 5,78E-02 |
| *SNHG18* | 6,77E-09 | 3,01E-08 | -2,7 |  | -7,4 | 4,90E-38 | 1,23E-36 |
| *SFT2D1* | 6,92E-09 | 3,08E-08 | 2,1 |  | 1,3 | 9,41E-12 | 5,52E-11 |
| *ACP7* | 6,95E-09 | 3,09E-08 | 4,6 |  | 1,8 | 6,18E-02 | 9,33E-02 |
| *MYO5A* | 6,96E-09 | 3,09E-08 | -1,8 |  | -5,1 | 1,80E-39 | 4,77E-38 |
| *DPH5* | 7,12E-09 | 3,16E-08 | -2,9 |  | -6,0 | 1,04E-14 | 7,55E-14 |
| *ETV1* | 7,20E-09 | 3,19E-08 | -2,3 |  | -3,6 | 4,40E-07 | 1,60E-06 |
| *GMPS* | 7,23E-09 | 3,21E-08 | -1,7 |  | -2,6 | 2,23E-06 | 7,38E-06 |
| *PLD1* | 7,28E-09 | 3,23E-08 | 2,4 |  | 0,7 | 3,33E-03 | 6,66E-03 |
| *ZNF771* | 7,30E-09 | 3,23E-08 | -5,8 |  | -6,5 | 3,09E-10 | 1,57E-09 |
| *PTPRG* | 7,40E-09 | 3,28E-08 | -1,9 |  | -4,6 | 4,64E-22 | 5,49E-21 |
| *FBXL14* | 7,50E-09 | 3,32E-08 | 3,6 |  | 2,9 | 4,12E-14 | 2,87E-13 |
| *DCBLD1* | 7,58E-09 | 3,35E-08 | 2,0 |  | 5,5 | 1,88E-15 | 1,44E-14 |
| *COPS6* | 7,61E-09 | 3,37E-08 | -2,0 |  | -2,4 | 1,42E-01 | 1,94E-01 |
| *SYNC* | 7,67E-09 | 3,39E-08 | 1,8 |  | 0,2 | 1,31E-01 | 1,81E-01 |
| *C20orf194* | 7,67E-09 | 3,39E-08 | 1,9 |  | 0,9 | 6,81E-09 | 3,03E-08 |
| *CYTH1* | 7,72E-09 | 3,41E-08 | 2,2 |  | 2,8 | 2,08E-12 | 1,28E-11 |
| *MEX3B* | 7,76E-09 | 3,43E-08 | -5,3 |  | -7,2 | 9,08E-10 | 4,44E-09 |
| *NOTCH2NLC* | 7,85E-09 | 3,47E-08 | 7,9 |  | 5,4 | 1,19E-05 | 3,59E-05 |
| *TMEM45A* | 7,96E-09 | 3,51E-08 | 2,2 |  | 3,3 | 5,35E-07 | 1,92E-06 |
| *RPS6KA6* | 8,20E-09 | 3,62E-08 | -4,2 |  | -9,8 | 1,12E-16 | 9,47E-16 |
| *STXBP5-AS1* | 8,29E-09 | 3,66E-08 | 2,8 |  | 2,0 | 1,11E-02 | 2,00E-02 |
| *LIPG* | 8,32E-09 | 3,67E-08 | 6,3 |  | 12,0 | 1,55E-03 | 3,31E-03 |
| *RRAGB* | 8,39E-09 | 3,70E-08 | -2,8 |  | -5,4 | 2,03E-11 | 1,15E-10 |
| *CLIP4* | 8,39E-09 | 3,70E-08 | -1,9 |  | -3,1 | 2,20E-07 | 8,32E-07 |
| *TFDP1* | 8,50E-09 | 3,74E-08 | -1,7 |  | -2,2 | 9,39E-02 | 1,35E-01 |
| *ZNF512B* | 8,57E-09 | 3,77E-08 | -2,5 |  | -2,1 | 4,50E-01 | 5,22E-01 |
| *FURIN* | 8,62E-09 | 3,80E-08 | 1,9 |  | 1,0 | 8,11E-04 | 1,81E-03 |
| *NMB* | 8,64E-09 | 3,80E-08 | -8,3 |  | -4,2 | 5,79E-08 | 2,35E-07 |
| *EFCAB2* | 8,75E-09 | 3,85E-08 | 4,2 |  | -2,2 | 5,09E-01 | 5,79E-01 |
| *STX10* | 8,78E-09 | 3,86E-08 | -2,7 |  | -3,4 | 1,95E-11 | 1,11E-10 |
| *HCLS1* | 8,88E-09 | 3,90E-08 | 7,1 |  | 4,4 | 7,05E-15 | 5,18E-14 |
| *CALM3* | 8,92E-09 | 3,92E-08 | 1,6 |  | 0,7 | 8,02E-05 | 2,12E-04 |
| *TMEM65* | 9,13E-09 | 4,01E-08 | -2,7 |  | -3,0 | 6,36E-05 | 1,71E-04 |
| *SHLD2* | 9,38E-09 | 4,12E-08 | 2,0 |  | -2,1 | 4,72E-01 | 5,43E-01 |
| *HERC4* | 9,61E-09 | 4,22E-08 | 1,7 |  | 1,0 | 1,18E-10 | 6,25E-10 |
| *BAHD1* | 9,96E-09 | 4,37E-08 | -2,5 |  | 0,1 | 6,54E-01 | 7,10E-01 |
| *EEF1D* | 1,01E-08 | 4,41E-08 | -1,8 |  | -4,1 | 2,22E-24 | 2,94E-23 |
| *RUVBL2* | 1,02E-08 | 4,45E-08 | -1,9 |  | -2,0 | 9,68E-01 | 9,75E-01 |
| *DBP* | 1,02E-08 | 4,47E-08 | -9,0 |  | -5,7 | 6,24E-06 | 1,96E-05 |
| *JAM3* | 1,02E-08 | 4,48E-08 | -1,8 |  | -4,2 | 2,16E-15 | 1,64E-14 |
| *TLR6* | 1,03E-08 | 4,49E-08 | -9,7 |  | -13,4 | 1,29E-12 | 8,09E-12 |
| *STUB1* | 1,06E-08 | 4,64E-08 | -1,8 |  | -2,1 | 4,57E-01 | 5,29E-01 |
| *MDFIC* | 1,06E-08 | 4,66E-08 | 2,1 |  | -2,2 | 1,28E-01 | 1,78E-01 |
| *TNFRSF1A* | 1,07E-08 | 4,67E-08 | 1,8 |  | 0,1 | 4,20E-01 | 4,92E-01 |
| *ALMS1* | 1,09E-08 | 4,78E-08 | 2,2 |  | 0,0 | 8,47E-01 | 8,78E-01 |
| *ASB1* | 1,10E-08 | 4,81E-08 | -2,2 |  | -4,1 | 4,59E-09 | 2,09E-08 |
| *TICAM1* | 1,11E-08 | 4,83E-08 | 4,2 |  | 5,1 | 9,51E-11 | 5,07E-10 |
| *SH2B3* | 1,12E-08 | 4,89E-08 | 2,0 |  | 1,2 | 5,68E-06 | 1,79E-05 |
| *ABCF1* | 1,13E-08 | 4,92E-08 | 1,7 |  | 0,7 | 6,52E-06 | 2,04E-05 |
| *FHOD3* | 1,13E-08 | 4,92E-08 | 2,1 |  | 0,5 | 7,69E-02 | 1,13E-01 |
| *GABARAPL1* | 1,19E-08 | 5,17E-08 | 1,8 |  | 0,2 | 4,29E-01 | 5,01E-01 |
| *ARG2* | 1,19E-08 | 5,19E-08 | -5,4 |  | 0,1 | 7,13E-01 | 7,63E-01 |
| *HLA-DMB* | 1,21E-08 | 5,25E-08 | 78,5 |  | 12,8 | 4,07E-06 | 1,31E-05 |
| *LHFPL2* | 1,22E-08 | 5,29E-08 | 2,2 |  | 1,4 | 6,24E-09 | 2,79E-08 |
| *TMEM154* | 1,23E-08 | 5,36E-08 | 2,2 |  | -2,5 | 6,25E-02 | 9,43E-02 |
| *PRMT6* | 1,24E-08 | 5,38E-08 | -2,7 |  | -2,4 | 7,71E-02 | 1,13E-01 |
| *DDB1* | 1,29E-08 | 5,61E-08 | -1,6 |  | -3,3 | 6,90E-17 | 5,90E-16 |
| *ANKFY1* | 1,32E-08 | 5,73E-08 | 2,5 |  | 2,8 | 3,12E-24 | 4,12E-23 |
| *GMPR2* | 1,32E-08 | 5,73E-08 | -2,2 |  | -2,8 | 4,32E-06 | 1,38E-05 |
| *LMBR1* | 1,33E-08 | 5,79E-08 | -1,9 |  | 0,2 | 1,70E-01 | 2,27E-01 |
| *CHSY1* | 1,36E-08 | 5,92E-08 | 2,1 |  | 1,0 | 1,04E-05 | 3,18E-05 |
| *HSBP1L1* | 1,40E-08 | 6,08E-08 | 6,3 |  | 1,1 | 3,95E-04 | 9,32E-04 |
| *RPS25* | 1,40E-08 | 6,09E-08 | -2,0 |  | -4,0 | 4,41E-20 | 4,68E-19 |
| *RPS6KB2* | 1,43E-08 | 6,20E-08 | -1,9 |  | -2,0 | 8,92E-01 | 9,14E-01 |
| *ZYG11B* | 1,44E-08 | 6,24E-08 | -1,6 |  | -3,4 | 6,52E-12 | 3,87E-11 |
| *COMMD7* | 1,44E-08 | 6,26E-08 | -1,9 |  | -2,1 | 5,42E-01 | 6,10E-01 |
| *POLDIP2* | 1,47E-08 | 6,35E-08 | -1,9 |  | -2,5 | 2,83E-03 | 5,74E-03 |
| *STX1B* | 1,47E-08 | 6,39E-08 | -9,5 |  | -2,7 | 1,96E-01 | 2,56E-01 |
| *ENSG00000263731* | 1,48E-08 | 6,40E-08 | -4,6 |  | -10,2 | 2,42E-09 | 1,14E-08 |
| *ESYT2* | 1,48E-08 | 6,40E-08 | -1,7 |  | -2,6 | 8,25E-04 | 1,84E-03 |
| *PSMG2* | 1,49E-08 | 6,43E-08 | 1,9 |  | 0,3 | 4,42E-02 | 6,91E-02 |
| *CPSF4* | 1,50E-08 | 6,48E-08 | -2,4 |  | -2,7 | 5,00E-04 | 1,16E-03 |
| *PFKL* | 1,52E-08 | 6,56E-08 | 1,7 |  | 2,8 | 6,70E-09 | 2,98E-08 |
| *RUBCN* | 1,53E-08 | 6,60E-08 | 2,2 |  | 6,5 | 1,86E-40 | 5,08E-39 |
| *C5orf15* | 1,55E-08 | 6,68E-08 | 1,8 |  | 2,8 | 1,81E-15 | 1,38E-14 |
| *TCEAL1* | 1,57E-08 | 6,77E-08 | -2,7 |  | -2,9 | 1,70E-04 | 4,26E-04 |
| *SEC14L2* | 1,61E-08 | 6,95E-08 | 2,9 |  | 0,4 | 1,70E-01 | 2,27E-01 |
| *NLGN1* | 1,62E-08 | 6,99E-08 | -14,3 |  | -38,5 | 4,99E-16 | 4,00E-15 |
| *DEXI* | 1,63E-08 | 7,03E-08 | -2,1 |  | -3,8 | 5,81E-17 | 5,00E-16 |
| *NAE1* | 1,69E-08 | 7,28E-08 | -1,9 |  | -2,6 | 1,48E-02 | 2,59E-02 |
| *TIMP2* | 1,71E-08 | 7,37E-08 | -2,1 |  | -4,4 | 4,34E-21 | 4,89E-20 |
| *CYGB* | 1,72E-08 | 7,41E-08 | 2,0 |  | -4,8 | 1,09E-24 | 1,48E-23 |
| *CHKA* | 1,73E-08 | 7,46E-08 | -2,6 |  | -4,1 | 3,10E-04 | 7,44E-04 |
| *HS1BP3* | 1,74E-08 | 7,50E-08 | -2,2 |  | -2,5 | 7,51E-03 | 1,40E-02 |
| *LARP4* | 1,79E-08 | 7,72E-08 | 2,0 |  | 1,0 | 1,55E-04 | 3,91E-04 |
| *PXYLP1* | 1,80E-08 | 7,74E-08 | -4,5 |  | -3,0 | 1,38E-02 | 2,44E-02 |
| *RPF2* | 1,81E-08 | 7,76E-08 | -1,7 |  | -4,3 | 2,33E-11 | 1,32E-10 |
| *HIBCH* | 1,83E-08 | 7,88E-08 | -2,8 |  | -6,2 | 1,64E-06 | 5,54E-06 |
| *BCS1L* | 1,89E-08 | 8,10E-08 | -2,9 |  | -3,7 | 2,34E-06 | 7,75E-06 |
| *CUL2* | 1,89E-08 | 8,10E-08 | 1,6 |  | 0,9 | 2,67E-13 | 1,76E-12 |
| *MYD88* | 1,93E-08 | 8,30E-08 | 9,8 |  | 7,9 | 9,50E-10 | 4,64E-09 |
| *VMA21* | 1,94E-08 | 8,33E-08 | -1,8 |  | -2,5 | 2,23E-02 | 3,76E-02 |
| *ACTR6* | 1,96E-08 | 8,38E-08 | 2,4 |  | 1,2 | 3,22E-04 | 7,71E-04 |
| *TPX2* | 1,96E-08 | 8,38E-08 | -2,4 |  | 0,1 | 8,47E-01 | 8,78E-01 |
| *ZCCHC8* | 1,96E-08 | 8,41E-08 | 2,0 |  | 0,4 | 1,15E-01 | 1,61E-01 |
| *MYO1D* | 2,01E-08 | 8,59E-08 | -2,5 |  | -37,4 | 1,63E-40 | 4,43E-39 |
| *GAB1* | 2,01E-08 | 8,62E-08 | 2,8 |  | -2,2 | 5,14E-01 | 5,84E-01 |
| *SERTAD2* | 2,02E-08 | 8,64E-08 | 2,6 |  | 0,1 | 4,62E-01 | 5,34E-01 |
| *NDUFS3* | 2,13E-08 | 9,11E-08 | -2,2 |  | -2,4 | 5,37E-02 | 8,23E-02 |
| *PSMG1* | 2,14E-08 | 9,14E-08 | -2,4 |  | -3,3 | 2,44E-04 | 5,93E-04 |
| *STMP1* | 2,18E-08 | 9,31E-08 | -2,6 |  | -4,2 | 2,78E-10 | 1,42E-09 |
| *KBTBD6* | 2,18E-08 | 9,32E-08 | -2,4 |  | -2,6 | 4,48E-02 | 7,00E-02 |
| *RPS26* | 2,18E-08 | 9,33E-08 | -1,8 |  | -3,4 | 4,17E-13 | 2,70E-12 |
| *NRBF2* | 2,20E-08 | 9,38E-08 | 2,0 |  | 0,2 | 1,18E-01 | 1,65E-01 |
| *YWHAG* | 2,20E-08 | 9,38E-08 | 1,7 |  | 0,5 | 1,44E-02 | 2,53E-02 |
| *THADA* | 2,21E-08 | 9,43E-08 | -2,1 |  | -5,0 | 2,85E-19 | 2,92E-18 |
| *CLIC1* | 2,23E-08 | 9,52E-08 | 1,7 |  | 1,7 | 3,98E-08 | 1,63E-07 |
| *MPP4* | 2,29E-08 | 9,77E-08 | -2,6 |  | -3,5 | 1,81E-02 | 3,11E-02 |
| *GOLGA8A* | 2,31E-08 | 9,84E-08 | 5,5 |  | -3,7 | 3,25E-02 | 5,25E-02 |
| *TSPYL1* | 2,32E-08 | 9,86E-08 | -1,7 |  | 0,5 | 4,42E-04 | 1,04E-03 |
| *DIS3L* | 2,34E-08 | 9,98E-08 | -2,5 |  | -3,4 | 1,97E-07 | 7,48E-07 |
| *FZD3* | 2,36E-08 | 1,00E-07 | -3,8 |  | -8,0 | 1,03E-13 | 6,97E-13 |
| *HOMER3* | 2,37E-08 | 1,01E-07 | -2,3 |  | -3,2 | 1,35E-06 | 4,60E-06 |
| *XKR8* | 2,38E-08 | 1,01E-07 | 2,4 |  | 0,4 | 6,74E-02 | 1,01E-01 |
| *CCNL1* | 2,40E-08 | 1,02E-07 | 2,7 |  | -3,3 | 8,33E-03 | 1,54E-02 |
| *ARL3* | 2,43E-08 | 1,04E-07 | -2,5 |  | -6,3 | 2,22E-23 | 2,81E-22 |
| *PTK2B* | 2,45E-08 | 1,04E-07 | 7,6 |  | 3,5 | 2,78E-05 | 7,95E-05 |
| *PPP2R5D* | 2,47E-08 | 1,05E-07 | -1,8 |  | -2,2 | 8,11E-02 | 1,19E-01 |
| *DSTN* | 2,49E-08 | 1,06E-07 | -1,7 |  | -5,1 | 1,59E-38 | 4,05E-37 |
| *RPS3AP6* | 2,49E-08 | 1,06E-07 | -4,3 |  | -7,6 | 1,71E-14 | 1,22E-13 |
| *CCNG2* | 2,57E-08 | 1,09E-07 | 2,5 |  | 0,7 | 1,09E-03 | 2,39E-03 |
| *TSPO* | 2,58E-08 | 1,09E-07 | 1,7 |  | 0,9 | 7,73E-14 | 5,28E-13 |
| *NOTCH3* | 2,59E-08 | 1,10E-07 | -19,8 |  | -3,3 | 4,26E-03 | 8,35E-03 |
| *SPTLC3* | 2,63E-08 | 1,12E-07 | -3,5 |  | -3,5 | 2,26E-06 | 7,48E-06 |
| *HBEGF* | 2,71E-08 | 1,15E-07 | 2,4 |  | 0,4 | 1,68E-01 | 2,26E-01 |
| *STARD4* | 2,79E-08 | 1,18E-07 | 2,0 |  | 1,7 | 2,89E-10 | 1,48E-09 |
| *TMEM51* | 2,81E-08 | 1,19E-07 | 3,6 |  | 33,9 | 5,55E-22 | 6,52E-21 |
| *TTC39B* | 2,82E-08 | 1,19E-07 | 2,3 |  | 0,1 | 4,85E-01 | 5,56E-01 |
| *PAQR4* | 2,87E-08 | 1,21E-07 | 3,0 |  | 3,0 | 1,21E-07 | 4,70E-07 |
| *CRAMP1* | 2,91E-08 | 1,23E-07 | 3,7 |  | 0,5 | 1,92E-01 | 2,53E-01 |
| *DTD2* | 2,93E-08 | 1,24E-07 | -2,8 |  | -3,9 | 1,51E-06 | 5,12E-06 |
| *ZNF346* | 2,96E-08 | 1,25E-07 | -2,2 |  | 0,0 | 8,81E-01 | 9,06E-01 |
| *SLC25A33* | 2,96E-08 | 1,25E-07 | -2,3 |  | -2,8 | 5,87E-03 | 1,12E-02 |
| *IPP* | 2,99E-08 | 1,26E-07 | -2,3 |  | -5,0 | 5,02E-13 | 3,24E-12 |
| *RHOU* | 3,03E-08 | 1,28E-07 | 3,5 |  | 41,8 | 8,06E-25 | 1,11E-23 |
| *SMURF2* | 3,06E-08 | 1,29E-07 | 1,5 |  | 4,0 | 2,53E-13 | 1,67E-12 |
| *IQGAP1* | 3,06E-08 | 1,29E-07 | 1,5 |  | 0,6 | 9,80E-08 | 3,86E-07 |
| *SIPA1L1* | 3,13E-08 | 1,32E-07 | 2,1 |  | 0,6 | 8,16E-03 | 1,51E-02 |
| *ZFP30* | 3,15E-08 | 1,33E-07 | 2,0 |  | -2,3 | 1,62E-01 | 2,18E-01 |
| *OSGIN2* | 3,16E-08 | 1,33E-07 | 2,0 |  | 0,5 | 6,22E-03 | 1,18E-02 |
| *PIK3CA* | 3,18E-08 | 1,34E-07 | 2,0 |  | -2,1 | 6,37E-01 | 6,95E-01 |
| *CNIH4* | 3,19E-08 | 1,34E-07 | 1,7 |  | 1,1 | 3,88E-09 | 1,77E-08 |
| *TMEM168* | 3,20E-08 | 1,35E-07 | -2,3 |  | -4,7 | 9,17E-11 | 4,90E-10 |
| *ZBTB41* | 3,21E-08 | 1,35E-07 | 2,5 |  | -2,2 | 5,05E-01 | 5,76E-01 |
| *SMARCA1* | 3,22E-08 | 1,36E-07 | -1,7 |  | -2,8 | 2,98E-06 | 9,71E-06 |
| *DYRK3* | 3,30E-08 | 1,39E-07 | -1,7 |  | -3,5 | 3,22E-10 | 1,64E-09 |
| *ISCU* | 3,32E-08 | 1,40E-07 | -1,8 |  | -2,8 | 7,65E-04 | 1,71E-03 |
| *AGRN* | 3,38E-08 | 1,42E-07 | 2,7 |  | 29,4 | 1,14E-62 | 5,58E-61 |
| *GRN* | 3,39E-08 | 1,42E-07 | 2,0 |  | 1,7 | 3,09E-08 | 1,29E-07 |
| *LINC02035* | 3,40E-08 | 1,43E-07 | -6,1 |  | -24,4 | 6,32E-15 | 4,66E-14 |
| *LMTK2* | 3,45E-08 | 1,45E-07 | 2,5 |  | 0,6 | 1,05E-04 | 2,72E-04 |
| *PA2G4* | 3,47E-08 | 1,46E-07 | -1,7 |  | -2,7 | 4,08E-05 | 1,14E-04 |
| *NOP53* | 3,49E-08 | 1,46E-07 | -1,9 |  | -3,2 | 2,59E-07 | 9,68E-07 |
| *TMEM25* | 3,49E-08 | 1,46E-07 | -2,6 |  | -2,6 | 3,24E-02 | 5,24E-02 |
| *OAT* | 3,57E-08 | 1,50E-07 | -1,6 |  | -3,1 | 4,66E-11 | 2,55E-10 |
| *UBE2A* | 3,62E-08 | 1,52E-07 | 1,7 |  | 0,3 | 3,13E-02 | 5,08E-02 |
| *ZBTB34* | 3,65E-08 | 1,53E-07 | 3,0 |  | 0,0 | 8,41E-01 | 8,73E-01 |
| *EAF1* | 3,67E-08 | 1,54E-07 | 1,9 |  | 2,1 | 6,65E-21 | 7,42E-20 |
| *PRLR* | 3,69E-08 | 1,55E-07 | 4,7 |  | 66,3 | 1,19E-06 | 4,11E-06 |
| *PEPD* | 3,77E-08 | 1,58E-07 | -1,8 |  | -3,7 | 1,62E-19 | 1,67E-18 |
| *MTHFD1L* | 3,80E-08 | 1,59E-07 | 2,0 |  | 0,3 | 1,77E-01 | 2,35E-01 |
| *MYO1E* | 3,80E-08 | 1,59E-07 | 1,7 |  | -2,4 | 1,13E-02 | 2,03E-02 |
| *MRPS25* | 3,80E-08 | 1,59E-07 | -2,0 |  | -4,7 | 1,30E-12 | 8,10E-12 |
| *TMBIM6* | 3,84E-08 | 1,60E-07 | 1,6 |  | 1,1 | 8,80E-09 | 3,87E-08 |
| *ZDHHC12* | 3,89E-08 | 1,62E-07 | 3,1 |  | 4,6 | 1,58E-18 | 1,54E-17 |
| *ATF6* | 3,89E-08 | 1,63E-07 | 1,6 |  | 0,6 | 8,70E-06 | 2,69E-05 |
| *CCT2* | 4,00E-08 | 1,67E-07 | -1,7 |  | -2,6 | 3,92E-05 | 1,10E-04 |
| *SLIRP* | 4,01E-08 | 1,67E-07 | -1,9 |  | -2,3 | 7,58E-02 | 1,12E-01 |
| *STOML2* | 4,02E-08 | 1,68E-07 | -1,8 |  | -2,5 | 1,16E-03 | 2,52E-03 |
| *LIMD1* | 4,04E-08 | 1,68E-07 | 2,0 |  | 0,9 | 1,87E-05 | 5,48E-05 |
| *EIF3H* | 4,06E-08 | 1,69E-07 | -1,6 |  | -3,0 | 1,06E-11 | 6,18E-11 |
| *ENSG00000225205* | 4,17E-08 | 1,74E-07 | 4,9 |  | 19,4 | 4,00E-09 | 1,82E-08 |
| *DHRS11* | 4,19E-08 | 1,75E-07 | -8,6 |  | -7,0 | 1,54E-02 | 2,69E-02 |
| *PPP1CC* | 4,28E-08 | 1,78E-07 | -1,5 |  | -2,1 | 5,95E-01 | 6,58E-01 |
| *AP1G2* | 4,30E-08 | 1,79E-07 | 4,8 |  | 2,9 | 9,69E-06 | 2,97E-05 |
| *VPS29* | 4,31E-08 | 1,79E-07 | 1,7 |  | 0,2 | 1,24E-01 | 1,72E-01 |
| *PGAM1* | 4,32E-08 | 1,79E-07 | 1,7 |  | 3,6 | 2,54E-13 | 1,68E-12 |
| *EEF1B2* | 4,32E-08 | 1,80E-07 | -2,0 |  | -9,1 | 3,34E-71 | 1,98E-69 |
| *SPINDOC* | 4,34E-08 | 1,80E-07 | 2,1 |  | 1,0 | 3,34E-04 | 7,96E-04 |
| *DGCR6L* | 4,39E-08 | 1,82E-07 | -2,1 |  | -2,2 | 2,89E-01 | 3,59E-01 |
| *GPAT4* | 4,39E-08 | 1,82E-07 | 1,6 |  | 0,0 | 8,84E-01 | 9,08E-01 |
| *ZNF616* | 4,40E-08 | 1,83E-07 | -3,2 |  | -3,4 | 5,32E-07 | 1,91E-06 |
| *SLC25A16* | 4,40E-08 | 1,83E-07 | -2,2 |  | -5,6 | 9,41E-13 | 5,95E-12 |
| *LIPA* | 4,42E-08 | 1,83E-07 | -1,8 |  | -2,1 | 7,37E-01 | 7,84E-01 |
| *EVC2* | 4,51E-08 | 1,87E-07 | 3,4 |  | 1,1 | 2,24E-07 | 8,45E-07 |
| *TNFRSF12A* | 4,59E-08 | 1,90E-07 | 1,8 |  | 3,5 | 5,16E-09 | 2,33E-08 |
| *CILK1* | 4,59E-08 | 1,90E-07 | -2,3 |  | -3,9 | 2,49E-12 | 1,53E-11 |
| *TVP23B* | 4,60E-08 | 1,91E-07 | 2,2 |  | 0,7 | 1,78E-04 | 4,45E-04 |
| *FLRT2* | 4,60E-08 | 1,91E-07 | 2,2 |  | -2,1 | 7,11E-01 | 7,62E-01 |
| *CHAC1* | 4,65E-08 | 1,93E-07 | 3,0 |  | 3,7 | 1,26E-03 | 2,72E-03 |
| *CDAN1* | 4,67E-08 | 1,93E-07 | -2,6 |  | -2,1 | 5,99E-01 | 6,61E-01 |
| *LNPEP* | 4,68E-08 | 1,93E-07 | 2,1 |  | 0,1 | 3,52E-01 | 4,25E-01 |
| *CLCN5* | 4,70E-08 | 1,94E-07 | -3,1 |  | -12,6 | 1,08E-45 | 3,53E-44 |
| *HYI* | 4,75E-08 | 1,96E-07 | -2,4 |  | -2,6 | 2,16E-02 | 3,66E-02 |
| *ANP32A* | 4,75E-08 | 1,96E-07 | -1,8 |  | 0,1 | 6,18E-01 | 6,78E-01 |
| *DESI2* | 4,75E-08 | 1,96E-07 | -1,8 |  | -3,3 | 2,74E-09 | 1,27E-08 |
| *ABLIM3* | 4,75E-08 | 1,96E-07 | 2,0 |  | -2,1 | 7,41E-01 | 7,88E-01 |
| *PAFAH2* | 4,81E-08 | 1,99E-07 | -2,4 |  | -2,2 | 3,74E-01 | 4,46E-01 |
| *H2BC8* | 4,85E-08 | 2,00E-07 | -2,7 |  | -2,4 | 1,65E-01 | 2,21E-01 |
| *TMEM9* | 4,86E-08 | 2,00E-07 | -2,0 |  | 0,0 | 9,94E-01 | 9,95E-01 |
| *SLC2A5* | 4,87E-08 | 2,01E-07 | 476,0 |  | 61,5 | 1,27E-201 | 4,33E-199 |
| *UQCC3* | 4,89E-08 | 2,02E-07 | -2,8 |  | -3,6 | 3,17E-03 | 6,37E-03 |
| *AMIGO1* | 4,93E-08 | 2,03E-07 | -3,2 |  | -3,0 | 2,82E-01 | 3,52E-01 |
| *RPS6KC1* | 4,96E-08 | 2,04E-07 | 1,8 |  | 1,5 | 1,64E-13 | 1,10E-12 |
| *FAM110A* | 4,97E-08 | 2,05E-07 | 5,5 |  | 2,1 | 1,00E-05 | 3,07E-05 |
| *TCF12* | 5,08E-08 | 2,09E-07 | -1,6 |  | -2,5 | 2,33E-04 | 5,69E-04 |
| *TRIM16L* | 5,10E-08 | 2,10E-07 | -3,6 |  | -4,5 | 3,85E-06 | 1,24E-05 |
| *KIDINS220* | 5,11E-08 | 2,10E-07 | -1,6 |  | -3,8 | 1,12E-07 | 4,37E-07 |
| *PDE4A* | 5,12E-08 | 2,11E-07 | 20,9 |  | 5,8 | 1,68E-04 | 4,22E-04 |
| *DMXL2* | 5,22E-08 | 2,15E-07 | 1,8 |  | -2,1 | 4,41E-01 | 5,12E-01 |
| *DMWD* | 5,23E-08 | 2,15E-07 | 2,4 |  | 0,6 | 2,91E-02 | 4,77E-02 |
| *ZNF821* | 5,24E-08 | 2,15E-07 | -3,6 |  | -2,3 | 2,25E-01 | 2,89E-01 |
| *NOP56* | 5,24E-08 | 2,15E-07 | -1,7 |  | -2,6 | 3,63E-04 | 8,62E-04 |
| *NDUFA8* | 5,24E-08 | 2,15E-07 | -2,0 |  | 0,1 | 5,33E-01 | 6,02E-01 |
| *MAPK13* | 5,25E-08 | 2,15E-07 | 3,3 |  | 1,6 | 2,35E-04 | 5,73E-04 |
| *MTF1* | 5,26E-08 | 2,16E-07 | 2,8 |  | 4,5 | 9,71E-36 | 2,25E-34 |
| *HOMER2* | 5,28E-08 | 2,17E-07 | -5,1 |  | -8,7 | 7,61E-15 | 5,57E-14 |
| *LAMA2* | 5,40E-08 | 2,21E-07 | -6,1 |  | -16,9 | 5,85E-15 | 4,32E-14 |
| *NOA1* | 5,48E-08 | 2,25E-07 | -2,4 |  | -3,6 | 1,79E-04 | 4,46E-04 |
| *RFK* | 5,55E-08 | 2,28E-07 | -2,1 |  | -2,4 | 1,62E-01 | 2,18E-01 |
| *BIVM* | 5,73E-08 | 2,35E-07 | -2,5 |  | -3,3 | 3,60E-07 | 1,32E-06 |
| *HYAL2* | 5,89E-08 | 2,41E-07 | -2,1 |  | -2,9 | 3,55E-04 | 8,44E-04 |
| *FAM32A* | 6,01E-08 | 2,46E-07 | 1,7 |  | 1,0 | 7,80E-09 | 3,44E-08 |
| *EEF2K* | 6,03E-08 | 2,47E-07 | -2,0 |  | -4,5 | 1,53E-16 | 1,27E-15 |
| *RPS19* | 6,09E-08 | 2,49E-07 | -1,7 |  | -3,7 | 1,29E-18 | 1,26E-17 |
| *GTF2H5* | 6,12E-08 | 2,50E-07 | -2,2 |  | -3,7 | 3,04E-25 | 4,32E-24 |
| *GAS2L3* | 6,26E-08 | 2,56E-07 | -4,3 |  | -9,1 | 2,31E-11 | 1,31E-10 |
| *ARRDC4* | 6,29E-08 | 2,57E-07 | -3,2 |  | -3,3 | 1,58E-03 | 3,35E-03 |
| *OBSL1* | 6,34E-08 | 2,59E-07 | -2,1 |  | -2,7 | 1,14E-04 | 2,95E-04 |
| *GGNBP2* | 6,37E-08 | 2,60E-07 | 1,8 |  | 0,1 | 7,72E-01 | 8,15E-01 |
| *C3orf52* | 6,38E-08 | 2,60E-07 | 2,8 |  | 0,3 | 4,84E-01 | 5,55E-01 |
| *ELP2* | 6,40E-08 | 2,61E-07 | -1,5 |  | -3,4 | 2,15E-23 | 2,71E-22 |
| *FBXL2* | 6,40E-08 | 2,61E-07 | -2,1 |  | -2,1 | 4,55E-01 | 5,27E-01 |
| *BRMS1* | 6,43E-08 | 2,62E-07 | -1,8 |  | 0,1 | 5,45E-01 | 6,12E-01 |
| *AGFG2* | 6,44E-08 | 2,62E-07 | -3,8 |  | -2,2 | 2,60E-01 | 3,28E-01 |
| *TMSB10* | 6,45E-08 | 2,63E-07 | 1,7 |  | 1,9 | 1,83E-10 | 9,53E-10 |
| *RPL13AP5* | 6,47E-08 | 2,64E-07 | -3,4 |  | -5,6 | 1,95E-09 | 9,20E-09 |
| *SCRN1* | 6,50E-08 | 2,65E-07 | 1,5 |  | 0,1 | 1,80E-01 | 2,39E-01 |
| *TP53TG1* | 6,59E-08 | 2,68E-07 | -4,0 |  | -3,6 | 8,52E-05 | 2,24E-04 |
| *REXO2* | 6,61E-08 | 2,69E-07 | -1,7 |  | -2,5 | 3,12E-03 | 6,29E-03 |
| *EGLN1* | 6,62E-08 | 2,69E-07 | 2,9 |  | 3,2 | 1,26E-18 | 1,24E-17 |
| *ZBTB17* | 6,68E-08 | 2,72E-07 | 2,1 |  | 1,5 | 3,86E-07 | 1,41E-06 |
| *TMEM184C* | 6,79E-08 | 2,76E-07 | -2,0 |  | -2,2 | 1,02E-01 | 1,45E-01 |
| *CPED1* | 6,92E-08 | 2,81E-07 | 1,7 |  | 0,3 | 2,35E-02 | 3,94E-02 |
| *RCBTB1* | 7,07E-08 | 2,87E-07 | -1,8 |  | -4,1 | 3,08E-08 | 1,29E-07 |
| *CHMP4B* | 7,08E-08 | 2,87E-07 | 1,8 |  | 0,8 | 2,55E-07 | 9,54E-07 |
| *MBD2* | 7,12E-08 | 2,89E-07 | 1,7 |  | 1,7 | 6,54E-23 | 8,10E-22 |
| *CDC6* | 7,18E-08 | 2,91E-07 | -2,7 |  | -2,6 | 2,11E-01 | 2,74E-01 |
| *ASTN2* | 7,26E-08 | 2,94E-07 | -10,8 |  | -11,5 | 2,78E-07 | 1,04E-06 |
| *ATP6V0A2* | 7,36E-08 | 2,98E-07 | 1,8 |  | 0,7 | 4,92E-05 | 1,35E-04 |
| *SYTL3* | 7,37E-08 | 2,98E-07 | 6,5 |  | 7,9 | 1,48E-18 | 1,45E-17 |
| *CSNK1G1* | 7,40E-08 | 3,00E-07 | 1,8 |  | 1,7 | 2,81E-16 | 2,30E-15 |
| *FBRS* | 7,54E-08 | 3,05E-07 | 2,8 |  | 1,8 | 1,08E-08 | 4,72E-08 |
| *CD302* | 7,57E-08 | 3,06E-07 | -9,5 |  | -33,4 | 4,97E-28 | 8,05E-27 |
| *NDUFB10* | 7,68E-08 | 3,11E-07 | -1,9 |  | -2,8 | 4,92E-05 | 1,35E-04 |
| *UNC119* | 7,75E-08 | 3,13E-07 | 2,1 |  | 0,9 | 1,90E-07 | 7,25E-07 |
| *PDK3* | 7,81E-08 | 3,15E-07 | -3,3 |  | -2,4 | 2,48E-01 | 3,15E-01 |
| *PPFIBP1* | 7,82E-08 | 3,16E-07 | 1,5 |  | 0,2 | 1,24E-01 | 1,72E-01 |
| *PER3* | 7,83E-08 | 3,16E-07 | -7,9 |  | -21,2 | 2,40E-22 | 2,88E-21 |
| *POLR3G* | 7,87E-08 | 3,18E-07 | -2,5 |  | -2,5 | 1,03E-01 | 1,47E-01 |
| *KIAA1549L* | 7,87E-08 | 3,18E-07 | -2,9 |  | -5,1 | 1,90E-10 | 9,85E-10 |
| *SOX11* | 7,93E-08 | 3,20E-07 | 2,4 |  | -4,3 | 4,56E-05 | 1,26E-04 |
| *EIF4E* | 7,98E-08 | 3,22E-07 | -1,8 |  | -2,2 | 1,55E-01 | 2,10E-01 |
| *USP9Y* | 8,03E-08 | 3,23E-07 | -1,9 |  | -3,4 | 3,18E-07 | 1,18E-06 |
| *TAF4B* | 8,03E-08 | 3,23E-07 | 3,4 |  | 2,4 | 2,83E-04 | 6,84E-04 |
| *RPL41* | 8,09E-08 | 3,26E-07 | -1,8 |  | -3,9 | 5,03E-18 | 4,70E-17 |
| *ERCC1* | 8,16E-08 | 3,28E-07 | -1,8 |  | -2,9 | 9,67E-03 | 1,76E-02 |
| *LRRC49* | 8,21E-08 | 3,30E-07 | 2,7 |  | -2,3 | 1,72E-01 | 2,30E-01 |
| *PIGG* | 8,41E-08 | 3,38E-07 | 1,6 |  | 1,6 | 1,36E-17 | 1,23E-16 |
| *BMP2K* | 8,44E-08 | 3,39E-07 | -1,9 |  | 0,1 | 4,49E-01 | 5,21E-01 |
| *ABAT* | 8,45E-08 | 3,40E-07 | -2,5 |  | -2,3 | 4,15E-01 | 4,87E-01 |
| *ZADH2* | 8,45E-08 | 3,40E-07 | -1,9 |  | 0,1 | 3,43E-01 | 4,15E-01 |
| *METAP1D* | 8,48E-08 | 3,41E-07 | -5,1 |  | -6,5 | 6,83E-09 | 3,04E-08 |
| *SLC25A25* | 8,51E-08 | 3,42E-07 | 2,1 |  | 0,6 | 7,98E-03 | 1,48E-02 |
| *RGS17* | 8,54E-08 | 3,43E-07 | 2,9 |  | -3,8 | 2,01E-04 | 4,96E-04 |
| *SLC27A1* | 8,56E-08 | 3,44E-07 | -2,9 |  | -3,6 | 2,38E-08 | 1,00E-07 |
| *RPL23AP82* | 8,57E-08 | 3,44E-07 | -2,1 |  | -3,9 | 1,76E-08 | 7,49E-08 |
| *CREB5* | 8,60E-08 | 3,45E-07 | -6,2 |  | -8,0 | 1,52E-04 | 3,85E-04 |
| *POLR1C* | 8,84E-08 | 3,55E-07 | -2,0 |  | -2,4 | 9,48E-02 | 1,36E-01 |
| *GRB2* | 9,05E-08 | 3,63E-07 | 1,7 |  | 0,8 | 5,06E-06 | 1,61E-05 |
| *RNF20* | 9,12E-08 | 3,66E-07 | -1,9 |  | -3,0 | 8,85E-12 | 5,20E-11 |
| *NONO* | 9,19E-08 | 3,68E-07 | -1,6 |  | -2,4 | 9,08E-03 | 1,67E-02 |
| *C19orf53* | 9,32E-08 | 3,73E-07 | -2,0 |  | -2,5 | 2,16E-02 | 3,66E-02 |
| *DBNL* | 9,40E-08 | 3,76E-07 | 1,6 |  | 0,4 | 3,91E-03 | 7,71E-03 |
| *UFM1* | 9,58E-08 | 3,83E-07 | 1,8 |  | -2,2 | 3,90E-01 | 4,62E-01 |
| *LONP2* | 9,60E-08 | 3,84E-07 | -1,6 |  | -2,2 | 7,38E-02 | 1,09E-01 |
| *MSH6* | 9,74E-08 | 3,89E-07 | -1,6 |  | -2,4 | 8,56E-03 | 1,58E-02 |
| *XIAP* | 9,95E-08 | 3,98E-07 | 1,8 |  | -2,2 | 1,46E-01 | 1,99E-01 |
| *CBX1* | 1,02E-07 | 4,06E-07 | -1,8 |  | -2,9 | 2,31E-05 | 6,69E-05 |
| *HDAC1* | 1,03E-07 | 4,09E-07 | 1,7 |  | 0,4 | 1,60E-03 | 3,39E-03 |
| *NUBP1* | 1,07E-07 | 4,25E-07 | 2,2 |  | 0,5 | 7,26E-04 | 1,63E-03 |
| *GTF2A2* | 1,07E-07 | 4,26E-07 | -1,9 |  | -2,7 | 4,75E-03 | 9,21E-03 |
| *CYTH2* | 1,07E-07 | 4,27E-07 | 2,0 |  | -2,1 | 6,88E-01 | 7,41E-01 |
| *DDIT4* | 1,08E-07 | 4,30E-07 | 2,4 |  | 12,0 | 7,64E-19 | 7,58E-18 |
| *SLC12A2* | 1,08E-07 | 4,31E-07 | -2,1 |  | -3,6 | 2,45E-05 | 7,07E-05 |
| *SNPH* | 1,08E-07 | 4,31E-07 | -2,5 |  | -2,1 | 5,36E-01 | 6,04E-01 |
| *SPRY2* | 1,11E-07 | 4,43E-07 | -3,0 |  | -3,7 | 2,66E-08 | 1,12E-07 |
| *PLPP5* | 1,11E-07 | 4,43E-07 | 2,1 |  | 0,3 | 7,79E-02 | 1,14E-01 |
| *NARF* | 1,12E-07 | 4,45E-07 | 1,9 |  | 0,3 | 4,75E-02 | 7,38E-02 |
| *SNX25* | 1,13E-07 | 4,49E-07 | 1,7 |  | 0,7 | 4,83E-06 | 1,54E-05 |
| *COPS8* | 1,13E-07 | 4,49E-07 | -1,6 |  | -2,8 | 2,70E-09 | 1,26E-08 |
| *ATP2C1* | 1,14E-07 | 4,53E-07 | 1,7 |  | 0,6 | 1,00E-02 | 1,82E-02 |
| *IST1* | 1,15E-07 | 4,57E-07 | 1,8 |  | 1,2 | 1,48E-15 | 1,14E-14 |
| *BTF3* | 1,15E-07 | 4,58E-07 | -1,6 |  | -3,1 | 1,13E-10 | 5,98E-10 |
| *MPP5* | 1,15E-07 | 4,58E-07 | -1,8 |  | -2,6 | 3,95E-05 | 1,11E-04 |
| *HEG1* | 1,16E-07 | 4,59E-07 | 4,0 |  | 0,9 | 1,05E-05 | 3,20E-05 |
| *CCDC113* | 1,16E-07 | 4,61E-07 | -3,5 |  | -9,0 | 8,56E-18 | 7,87E-17 |
| *PSMD12* | 1,18E-07 | 4,68E-07 | 1,8 |  | 1,2 | 1,20E-08 | 5,22E-08 |
| *EIF4EBP1* | 1,18E-07 | 4,69E-07 | -2,0 |  | 0,7 | 1,70E-01 | 2,27E-01 |
| *RDH10* | 1,18E-07 | 4,69E-07 | -2,8 |  | -3,4 | 6,62E-04 | 1,50E-03 |
| *PDLIM4* | 1,19E-07 | 4,73E-07 | 2,1 |  | 0,3 | 4,09E-01 | 4,81E-01 |
| *SLCO5A1* | 1,22E-07 | 4,83E-07 | 81,3 |  | 125,5 | 2,38E-16 | 1,96E-15 |
| *MFHAS1* | 1,23E-07 | 4,88E-07 | 2,4 |  | 0,7 | 3,09E-05 | 8,81E-05 |
| *STRAP* | 1,23E-07 | 4,88E-07 | -1,6 |  | -2,7 | 3,78E-06 | 1,22E-05 |
| *ARID4B* | 1,24E-07 | 4,92E-07 | 2,2 |  | 0,2 | 4,05E-01 | 4,77E-01 |
| *NOL4L* | 1,24E-07 | 4,92E-07 | 2,5 |  | 0,4 | 8,68E-02 | 1,26E-01 |
| *ZNF491* | 1,25E-07 | 4,95E-07 | -7,3 |  | -8,2 | 2,04E-05 | 5,96E-05 |
| *NIPAL3* | 1,26E-07 | 5,00E-07 | -1,7 |  | -2,7 | 1,81E-02 | 3,12E-02 |
| *PDE7B* | 1,27E-07 | 5,01E-07 | -20,0 |  | -3,6 | 1,37E-05 | 4,09E-05 |
| *BTF3L4* | 1,28E-07 | 5,05E-07 | -1,7 |  | 0,1 | 4,88E-01 | 5,59E-01 |
| *GNA11* | 1,29E-07 | 5,10E-07 | 1,5 |  | 1,1 | 2,43E-06 | 8,02E-06 |
| *LINC00205* | 1,29E-07 | 5,10E-07 | -2,2 |  | -3,0 | 1,80E-04 | 4,48E-04 |
| *MFAP3L* | 1,32E-07 | 5,20E-07 | -2,2 |  | -7,1 | 3,25E-09 | 1,50E-08 |
| *TMEM147* | 1,34E-07 | 5,30E-07 | -1,9 |  | -2,8 | 2,05E-03 | 4,29E-03 |
| *PTPRE* | 1,35E-07 | 5,34E-07 | 4,8 |  | 2,7 | 4,58E-17 | 3,96E-16 |
| *CDIP1* | 1,38E-07 | 5,42E-07 | -2,0 |  | 0,2 | 1,41E-01 | 1,93E-01 |
| *MOAP1* | 1,38E-07 | 5,44E-07 | 1,9 |  | 0,9 | 3,39E-06 | 1,10E-05 |
| *KEAP1* | 1,38E-07 | 5,45E-07 | 1,7 |  | 0,7 | 1,01E-06 | 3,51E-06 |
| *AFF1* | 1,40E-07 | 5,50E-07 | 2,4 |  | 1,9 | 1,11E-09 | 5,39E-09 |
| *CTPS1* | 1,40E-07 | 5,51E-07 | 2,1 |  | -2,8 | 1,62E-03 | 3,43E-03 |
| *MGAT1* | 1,40E-07 | 5,53E-07 | 1,7 |  | 1,4 | 2,28E-09 | 1,07E-08 |
| *PSMB2* | 1,42E-07 | 5,60E-07 | 1,6 |  | 1,3 | 2,28E-10 | 1,18E-09 |
| *NORAD* | 1,45E-07 | 5,72E-07 | -1,7 |  | -4,4 | 2,35E-19 | 2,42E-18 |
| *PIP4P2* | 1,46E-07 | 5,75E-07 | -2,0 |  | -2,1 | 4,81E-01 | 5,52E-01 |
| *GCC2* | 1,47E-07 | 5,77E-07 | 2,2 |  | 1,0 | 4,33E-04 | 1,02E-03 |
| *AKR7A2* | 1,48E-07 | 5,83E-07 | -2,4 |  | -4,1 | 4,20E-25 | 5,90E-24 |
| *CSE1L* | 1,52E-07 | 5,97E-07 | -1,6 |  | -2,1 | 4,54E-01 | 5,26E-01 |
| *APAF1* | 1,52E-07 | 5,98E-07 | 1,7 |  | 0,4 | 1,88E-02 | 3,23E-02 |
| *TRIP4* | 1,54E-07 | 6,06E-07 | 2,5 |  | 1,6 | 9,78E-06 | 3,00E-05 |
| *NT5C3B* | 1,55E-07 | 6,07E-07 | -1,8 |  | -2,5 | 1,70E-03 | 3,59E-03 |
| *CLGN* | 1,55E-07 | 6,07E-07 | -2,6 |  | -13,0 | 4,85E-25 | 6,77E-24 |
| *GLMP* | 1,55E-07 | 6,10E-07 | -1,8 |  | -2,5 | 2,17E-03 | 4,51E-03 |
| *HSF2* | 1,59E-07 | 6,22E-07 | -2,1 |  | -2,1 | 6,30E-01 | 6,89E-01 |
| *PLPP3* | 1,59E-07 | 6,24E-07 | 2,5 |  | 0,4 | 3,05E-02 | 4,97E-02 |
| *MYO9B* | 1,60E-07 | 6,25E-07 | 2,0 |  | 1,6 | 6,20E-12 | 3,69E-11 |
| *NDUFS5* | 1,60E-07 | 6,25E-07 | -1,8 |  | -3,5 | 9,10E-17 | 7,73E-16 |
| *TPST2* | 1,62E-07 | 6,35E-07 | -2,1 |  | -2,8 | 2,43E-03 | 5,00E-03 |
| *PI4K2A* | 1,63E-07 | 6,38E-07 | 2,0 |  | 2,0 | 5,28E-17 | 4,55E-16 |
| *PLPPR2* | 1,64E-07 | 6,41E-07 | -2,3 |  | -2,6 | 8,21E-03 | 1,52E-02 |
| *TOMM34* | 1,64E-07 | 6,43E-07 | -1,7 |  | -2,2 | 1,68E-01 | 2,25E-01 |
| *GORAB* | 1,65E-07 | 6,44E-07 | 2,5 |  | 0,2 | 3,25E-01 | 3,97E-01 |
| *NAV2* | 1,65E-07 | 6,44E-07 | 9,8 |  | 5,3 | 4,41E-22 | 5,22E-21 |
| *TRUB2* | 1,68E-07 | 6,56E-07 | -2,0 |  | -2,6 | 7,50E-04 | 1,68E-03 |
| *RMND5A* | 1,69E-07 | 6,60E-07 | -2,4 |  | -3,8 | 1,67E-05 | 4,95E-05 |
| *SLC35A5* | 1,69E-07 | 6,60E-07 | 1,8 |  | 1,1 | 1,19E-11 | 6,95E-11 |
| *SCFD2* | 1,69E-07 | 6,61E-07 | -2,2 |  | -6,3 | 8,12E-41 | 2,23E-39 |
| *MAFK* | 1,71E-07 | 6,66E-07 | 2,2 |  | 2,0 | 3,46E-15 | 2,59E-14 |
| *PLEKHA4* | 1,71E-07 | 6,69E-07 | 5,8 |  | 2,8 | 2,37E-03 | 4,88E-03 |
| *ANTXR1* | 1,72E-07 | 6,70E-07 | 2,1 |  | -4,0 | 1,71E-38 | 4,34E-37 |
| *CHMP1B* | 1,78E-07 | 6,95E-07 | 1,7 |  | 1,4 | 1,05E-16 | 8,87E-16 |
| *C3AR1* | 1,79E-07 | 6,97E-07 | 15,7 |  | 184,6 | 6,81E-23 | 8,42E-22 |
| *ERCC6* | 1,80E-07 | 7,00E-07 | -1,9 |  | -2,2 | 3,89E-01 | 4,61E-01 |
| *MED10* | 1,81E-07 | 7,06E-07 | 2,2 |  | 1,0 | 1,32E-05 | 3,95E-05 |
| *TSPAN31* | 1,82E-07 | 7,08E-07 | 2,0 |  | 0,3 | 1,07E-01 | 1,52E-01 |
| *SHOC2* | 1,82E-07 | 7,08E-07 | 1,8 |  | 0,5 | 5,78E-04 | 1,32E-03 |
| *RPL7L1* | 1,82E-07 | 7,08E-07 | -1,6 |  | -2,4 | 5,88E-03 | 1,12E-02 |
| *INSYN2B* | 1,83E-07 | 7,12E-07 | -1,8 |  | -45,9 | 6,58E-08 | 2,65E-07 |
| *C1orf109* | 1,83E-07 | 7,13E-07 | -2,3 |  | -3,4 | 2,28E-06 | 7,55E-06 |
| *STMN3* | 1,84E-07 | 7,16E-07 | 2,3 |  | 3,4 | 2,62E-06 | 8,59E-06 |
| *FKRP* | 1,86E-07 | 7,22E-07 | 2,1 |  | 1,0 | 2,28E-08 | 9,63E-08 |
| *SEPTIN8* | 1,88E-07 | 7,28E-07 | 1,9 |  | 0,9 | 5,12E-12 | 3,07E-11 |
| *LETM1* | 1,88E-07 | 7,29E-07 | -1,7 |  | -2,1 | 7,55E-01 | 8,00E-01 |
| *P2RX6* | 1,90E-07 | 7,37E-07 | -6,1 |  | -9,3 | 1,87E-08 | 7,96E-08 |
| *PPME1* | 1,91E-07 | 7,43E-07 | -2,1 |  | 1,2 | 4,19E-06 | 1,34E-05 |
| *C1QBP* | 1,96E-07 | 7,59E-07 | -1,7 |  | -2,2 | 1,90E-01 | 2,49E-01 |
| *NEDD1* | 1,96E-07 | 7,60E-07 | 2,4 |  | 2,0 | 3,32E-15 | 2,49E-14 |
| *USP38* | 1,97E-07 | 7,62E-07 | 1,8 |  | 0,2 | 2,76E-01 | 3,45E-01 |
| *GPBP1* | 1,97E-07 | 7,65E-07 | 1,9 |  | 1,4 | 2,00E-09 | 9,43E-09 |
| *LNCSRLR* | 1,98E-07 | 7,66E-07 | 11,4 |  | 1,6 | 1,01E-01 | 1,44E-01 |
| *CDKN2AIPNL* | 1,99E-07 | 7,70E-07 | -2,2 |  | -3,0 | 1,89E-02 | 3,24E-02 |
| *PIP4K2A* | 1,99E-07 | 7,70E-07 | 1,7 |  | -2,1 | 4,30E-01 | 5,02E-01 |
| *MDN1* | 2,00E-07 | 7,73E-07 | -2,0 |  | -6,1 | 3,28E-18 | 3,11E-17 |
| *MGAT4B* | 2,00E-07 | 7,73E-07 | 1,6 |  | -2,1 | 5,15E-01 | 5,85E-01 |
| *REEP2* | 2,02E-07 | 7,82E-07 | -2,8 |  | -2,6 | 4,01E-02 | 6,33E-02 |
| *ARNT* | 2,02E-07 | 7,82E-07 | 1,6 |  | 0,4 | 1,53E-04 | 3,86E-04 |
| *HSD17B4* | 2,02E-07 | 7,82E-07 | -1,7 |  | -3,4 | 1,79E-14 | 1,28E-13 |
| *NAB2* | 2,07E-07 | 8,01E-07 | 2,2 |  | 0,8 | 4,11E-13 | 2,67E-12 |
| *MAP4K5* | 2,10E-07 | 8,10E-07 | -1,9 |  | -4,2 | 4,27E-20 | 4,56E-19 |
| *KCNS3* | 2,10E-07 | 8,11E-07 | 184,9 |  | 73,8 | 4,12E-45 | 1,31E-43 |
| *MOGS* | 2,11E-07 | 8,14E-07 | 1,8 |  | 0,5 | 3,21E-02 | 5,20E-02 |
| *CCDC91* | 2,11E-07 | 8,14E-07 | 2,5 |  | 0,3 | 4,51E-02 | 7,04E-02 |
| *SLU7* | 2,15E-07 | 8,31E-07 | 1,6 |  | 0,4 | 6,75E-03 | 1,27E-02 |
| *RFXANK* | 2,18E-07 | 8,40E-07 | 1,8 |  | 1,2 | 3,22E-07 | 1,19E-06 |
| *ZNF281* | 2,21E-07 | 8,52E-07 | 1,7 |  | 3,5 | 9,00E-32 | 1,75E-30 |
| *WDCP* | 2,21E-07 | 8,53E-07 | -2,2 |  | -2,5 | 2,53E-02 | 4,21E-02 |
| *NAA38* | 2,23E-07 | 8,57E-07 | -2,1 |  | -3,1 | 4,50E-06 | 1,44E-05 |
| *PTPRF* | 2,23E-07 | 8,59E-07 | -2,4 |  | 1,4 | 1,91E-02 | 3,28E-02 |
| *TEF* | 2,23E-07 | 8,59E-07 | -3,0 |  | -4,3 | 1,55E-09 | 7,42E-09 |
| *RASSF1* | 2,26E-07 | 8,69E-07 | 2,3 |  | 1,0 | 9,56E-05 | 2,50E-04 |
| *TMED4* | 2,26E-07 | 8,69E-07 | 1,7 |  | 0,9 | 2,81E-07 | 1,05E-06 |
| *APBA3* | 2,29E-07 | 8,81E-07 | 2,6 |  | 3,2 | 1,84E-10 | 9,58E-10 |
| *USP11* | 2,29E-07 | 8,81E-07 | 1,8 |  | 2,0 | 2,70E-17 | 2,37E-16 |
| *ENSG00000266208* | 2,30E-07 | 8,84E-07 | 5,0 |  | -2,4 | 3,00E-01 | 3,71E-01 |
| *TBX2* | 2,30E-07 | 8,84E-07 | -3,4 |  | 0,7 | 1,11E-03 | 2,42E-03 |
| *KLHL28* | 2,31E-07 | 8,86E-07 | 3,0 |  | 0,5 | 2,89E-02 | 4,73E-02 |
| *PGM3* | 2,39E-07 | 9,18E-07 | 1,6 |  | -3,2 | 9,22E-11 | 4,92E-10 |
| *ZNF678* | 2,40E-07 | 9,20E-07 | 2,7 |  | 1,7 | 2,67E-05 | 7,67E-05 |
| *NME1* | 2,40E-07 | 9,20E-07 | -2,4 |  | -3,3 | 7,95E-04 | 1,77E-03 |
| *MAPK8IP1* | 2,41E-07 | 9,22E-07 | -2,5 |  | -2,9 | 9,30E-03 | 1,71E-02 |
| *TARBP2* | 2,41E-07 | 9,23E-07 | -1,8 |  | -2,2 | 2,25E-01 | 2,89E-01 |
| *ADCY6* | 2,42E-07 | 9,27E-07 | -2,2 |  | 0,3 | 9,51E-02 | 1,37E-01 |
| *NUBPL* | 2,42E-07 | 9,27E-07 | -2,6 |  | -7,1 | 1,77E-12 | 1,10E-11 |
| *IFNAR2* | 2,43E-07 | 9,29E-07 | 25,9 |  | 9,4 | 9,48E-23 | 1,17E-21 |
| *FAM117B* | 2,45E-07 | 9,40E-07 | 4,3 |  | 3,5 | 1,11E-12 | 6,97E-12 |
| *GBE1* | 2,46E-07 | 9,42E-07 | 1,6 |  | 0,3 | 1,65E-01 | 2,21E-01 |
| *SSR2* | 2,47E-07 | 9,44E-07 | -1,7 |  | -3,4 | 1,73E-08 | 7,38E-08 |
| *RAB22A* | 2,47E-07 | 9,45E-07 | -1,6 |  | -2,5 | 1,21E-02 | 2,16E-02 |
| *RRS1* | 2,50E-07 | 9,57E-07 | -3,3 |  | -4,0 | 2,71E-06 | 8,88E-06 |
| *NHP2* | 2,53E-07 | 9,67E-07 | -2,0 |  | -3,7 | 3,59E-09 | 1,64E-08 |
| *SNHG19* | 2,56E-07 | 9,79E-07 | -10,3 |  | -50,5 | 2,72E-19 | 2,79E-18 |
| *SELENOW* | 2,58E-07 | 9,84E-07 | -1,6 |  | 0,1 | 6,37E-01 | 6,96E-01 |
| *ATP13A2* | 2,59E-07 | 9,88E-07 | 1,8 |  | 0,4 | 8,97E-02 | 1,30E-01 |
| *PPP1R37* | 2,59E-07 | 9,88E-07 | -1,9 |  | -2,5 | 3,34E-04 | 7,98E-04 |
| *MT1X* | 2,60E-07 | 9,93E-07 | 7,8 |  | 8,7 | 5,02E-09 | 2,27E-08 |
| *ICE2* | 2,61E-07 | 9,94E-07 | -1,7 |  | -3,1 | 1,25E-05 | 3,77E-05 |
| *FAM241A* | 2,61E-07 | 9,94E-07 | 3,3 |  | 2,7 | 1,07E-05 | 3,27E-05 |
| *WAC* | 2,62E-07 | 9,98E-07 | 1,5 |  | 0,2 | 7,17E-02 | 1,06E-01 |
| *TAOK3* | 2,65E-07 | 1,01E-06 | 1,7 |  | 0,4 | 1,10E-02 | 1,98E-02 |
| *ABI2* | 2,65E-07 | 1,01E-06 | -1,9 |  | -2,4 | 1,11E-02 | 2,01E-02 |
| *STK4* | 2,70E-07 | 1,03E-06 | 1,6 |  | 0,6 | 1,19E-04 | 3,07E-04 |
| *SHANK1* | 2,76E-07 | 1,05E-06 | -14,7 |  | -5,6 | 2,24E-03 | 4,63E-03 |
| *RNASEH1* | 2,77E-07 | 1,05E-06 | -1,8 |  | -5,2 | 7,32E-27 | 1,12E-25 |
| *PPTC7* | 2,80E-07 | 1,07E-06 | 3,4 |  | 3,3 | 1,43E-09 | 6,84E-09 |
| *FKBP4* | 2,80E-07 | 1,07E-06 | -1,8 |  | 0,0 | 8,83E-01 | 9,07E-01 |
| *POLD3* | 2,83E-07 | 1,08E-06 | 2,6 |  | 1,2 | 2,01E-07 | 7,63E-07 |
| *ANGPTL2* | 2,85E-07 | 1,08E-06 | -2,4 |  | -12,4 | 7,33E-12 | 4,33E-11 |
| *HINT3* | 2,85E-07 | 1,08E-06 | 2,1 |  | -2,0 | 8,57E-01 | 8,86E-01 |
| *CNOT7* | 2,86E-07 | 1,09E-06 | -1,6 |  | -2,1 | 5,57E-01 | 6,23E-01 |
| *ZFYVE26* | 2,88E-07 | 1,09E-06 | 1,8 |  | 2,3 | 2,49E-17 | 2,20E-16 |
| *ZNF287* | 2,88E-07 | 1,09E-06 | -3,1 |  | -4,4 | 5,36E-06 | 1,69E-05 |
| *CHRNB1* | 2,89E-07 | 1,10E-06 | 5,9 |  | 2,1 | 5,12E-05 | 1,40E-04 |
| *NSUN5* | 2,93E-07 | 1,11E-06 | 2,8 |  | 1,3 | 1,17E-05 | 3,55E-05 |
| *MAFG* | 2,95E-07 | 1,12E-06 | 2,0 |  | 0,1 | 2,26E-01 | 2,90E-01 |
| *PTGES3* | 2,97E-07 | 1,13E-06 | 1,7 |  | 1,1 | 2,79E-07 | 1,04E-06 |
| *PDE5A* | 2,98E-07 | 1,13E-06 | -20,7 |  | -41,0 | 4,10E-94 | 3,73E-92 |
| *PIGA* | 3,03E-07 | 1,15E-06 | 3,3 |  | 2,1 | 2,04E-07 | 7,71E-07 |
| *KLHDC10* | 3,04E-07 | 1,15E-06 | -1,8 |  | -2,4 | 1,24E-02 | 2,21E-02 |
| *KLHL11* | 3,05E-07 | 1,15E-06 | -2,0 |  | -3,3 | 1,35E-08 | 5,82E-08 |
| *TMED3* | 3,05E-07 | 1,15E-06 | -1,7 |  | -2,7 | 2,28E-04 | 5,59E-04 |
| *STXBP3* | 3,07E-07 | 1,16E-06 | 2,1 |  | 0,5 | 4,27E-02 | 6,70E-02 |
| *PCOLCE* | 3,08E-07 | 1,16E-06 | -1,9 |  | -4,8 | 8,31E-33 | 1,69E-31 |
| *ZNF462* | 3,09E-07 | 1,17E-06 | 2,2 |  | 1,6 | 2,37E-12 | 1,45E-11 |
| *PCDHGC3* | 3,10E-07 | 1,17E-06 | -3,0 |  | -9,1 | 6,21E-24 | 8,07E-23 |
| *NBPF10* | 3,12E-07 | 1,18E-06 | 4,0 |  | 1,6 | 1,10E-04 | 2,84E-04 |
| *LIMK1* | 3,13E-07 | 1,18E-06 | 1,7 |  | 0,3 | 6,85E-02 | 1,02E-01 |
| *TXNDC9* | 3,19E-07 | 1,20E-06 | 1,6 |  | 0,2 | 4,42E-01 | 5,14E-01 |
| *ARL5B* | 3,22E-07 | 1,21E-06 | 2,0 |  | 1,5 | 4,05E-05 | 1,13E-04 |
| *OAZ2* | 3,22E-07 | 1,22E-06 | -1,7 |  | -2,2 | 2,07E-01 | 2,68E-01 |
| *MAP1S* | 3,25E-07 | 1,22E-06 | 3,1 |  | 5,8 | 4,05E-18 | 3,81E-17 |
| *NTN4* | 3,25E-07 | 1,22E-06 | -6,2 |  | -4,5 | 3,82E-03 | 7,56E-03 |
| *EFCAB14* | 3,25E-07 | 1,23E-06 | 1,6 |  | 0,9 | 2,62E-12 | 1,60E-11 |
| *HMCN1* | 3,25E-07 | 1,23E-06 | -3,1 |  | -105,1 | 9,67E-93 | 8,36E-91 |
| *ARHGAP32* | 3,28E-07 | 1,24E-06 | -4,2 |  | -3,1 | 2,93E-03 | 5,94E-03 |
| *FITM2* | 3,30E-07 | 1,24E-06 | -2,9 |  | -13,7 | 4,19E-19 | 4,23E-18 |
| *WDR44* | 3,32E-07 | 1,25E-06 | 1,8 |  | 0,7 | 1,19E-04 | 3,07E-04 |
| *GTPBP6* | 3,33E-07 | 1,25E-06 | -2,0 |  | -2,8 | 1,64E-02 | 2,86E-02 |
| *MRPL51* | 3,33E-07 | 1,25E-06 | -1,8 |  | -2,9 | 4,41E-05 | 1,22E-04 |
| *RPLP1* | 3,34E-07 | 1,25E-06 | -2,0 |  | -4,2 | 1,08E-20 | 1,18E-19 |
| *PDLIM5* | 3,35E-07 | 1,26E-06 | 1,7 |  | -2,6 | 9,05E-04 | 2,00E-03 |
| *ZNF518A* | 3,43E-07 | 1,29E-06 | 2,6 |  | -2,0 | 9,07E-01 | 9,28E-01 |
| *FAM107B* | 3,44E-07 | 1,29E-06 | 2,3 |  | 1,1 | 7,15E-07 | 2,54E-06 |
| *TMEM217* | 3,45E-07 | 1,29E-06 | 38,2 |  | 21,4 | 7,33E-25 | 1,01E-23 |
| *RPS13* | 3,45E-07 | 1,30E-06 | -1,7 |  | -4,8 | 7,68E-33 | 1,56E-31 |
| *TPI1* | 3,48E-07 | 1,30E-06 | 1,7 |  | 3,7 | 3,73E-08 | 1,53E-07 |
| *CEP68* | 3,49E-07 | 1,31E-06 | -3,1 |  | -4,3 | 2,37E-07 | 8,89E-07 |
| *ZNF75A* | 3,56E-07 | 1,33E-06 | 2,0 |  | -2,1 | 5,80E-01 | 6,44E-01 |
| *CEMIP* | 3,56E-07 | 1,34E-06 | -16,5 |  | -174,2 | 1,31E-22 | 1,60E-21 |
| *DCUN1D3* | 3,57E-07 | 1,34E-06 | 1,8 |  | 0,6 | 1,54E-03 | 3,28E-03 |
| *NOMO2* | 3,58E-07 | 1,34E-06 | 1,8 |  | 2,8 | 1,01E-15 | 7,85E-15 |
| *PDCD2* | 3,58E-07 | 1,34E-06 | -1,8 |  | -2,8 | 3,76E-06 | 1,21E-05 |
| *THY1* | 3,60E-07 | 1,35E-06 | -1,6 |  | -6,1 | 8,05E-30 | 1,41E-28 |
| *ZBTB25* | 3,62E-07 | 1,36E-06 | 2,2 |  | 0,3 | 1,08E-01 | 1,53E-01 |
| *NDUFB6* | 3,64E-07 | 1,36E-06 | -2,0 |  | -4,2 | 5,14E-16 | 4,11E-15 |
| *NDUFA2* | 3,65E-07 | 1,37E-06 | -1,9 |  | -2,3 | 1,67E-01 | 2,23E-01 |
| *COPS9* | 3,66E-07 | 1,37E-06 | -2,3 |  | -4,0 | 1,45E-13 | 9,75E-13 |
| *FOSL1* | 3,67E-07 | 1,37E-06 | 2,5 |  | 17,2 | 3,29E-30 | 5,82E-29 |
| *UQCRFS1* | 3,68E-07 | 1,38E-06 | -1,7 |  | 0,1 | 4,99E-01 | 5,69E-01 |
| *CUL1* | 3,72E-07 | 1,39E-06 | 1,6 |  | 1,0 | 3,30E-11 | 1,83E-10 |
| *HGS* | 3,73E-07 | 1,39E-06 | 1,7 |  | 1,9 | 3,20E-13 | 2,09E-12 |
| *ATAD1* | 3,76E-07 | 1,40E-06 | 1,8 |  | 0,4 | 8,47E-03 | 1,56E-02 |
| *CD276* | 3,78E-07 | 1,41E-06 | -1,8 |  | -2,3 | 1,58E-01 | 2,14E-01 |
| *RPA1* | 3,80E-07 | 1,42E-06 | -2,2 |  | -2,9 | 5,38E-08 | 2,18E-07 |
| *SETDB1* | 3,80E-07 | 1,42E-06 | 1,9 |  | 0,7 | 2,56E-05 | 7,37E-05 |
| *NELFCD* | 3,85E-07 | 1,44E-06 | -1,7 |  | -2,3 | 6,82E-03 | 1,28E-02 |
| *SEMA4B* | 3,87E-07 | 1,44E-06 | 3,0 |  | 8,8 | 1,19E-22 | 1,45E-21 |
| *AKAP1* | 3,89E-07 | 1,45E-06 | -2,6 |  | -3,1 | 1,76E-02 | 3,04E-02 |
| *PATL1* | 3,91E-07 | 1,45E-06 | 2,5 |  | 6,0 | 8,45E-35 | 1,89E-33 |
| *DHX29* | 3,93E-07 | 1,46E-06 | -1,6 |  | -2,9 | 5,71E-08 | 2,31E-07 |
| *MCRIP1* | 3,96E-07 | 1,47E-06 | -1,8 |  | -3,2 | 6,87E-06 | 2,15E-05 |
| *NBL1* | 3,99E-07 | 1,49E-06 | 2,2 |  | -3,5 | 2,54E-13 | 1,68E-12 |
| *EFNB2* | 4,00E-07 | 1,49E-06 | 2,6 |  | 3,0 | 1,77E-07 | 6,76E-07 |
| *CARD8* | 4,00E-07 | 1,49E-06 | 2,0 |  | 0,1 | 5,64E-01 | 6,30E-01 |
| *AGTPBP1* | 4,01E-07 | 1,49E-06 | 2,4 |  | 0,2 | 3,45E-01 | 4,17E-01 |
| *ABCA5* | 4,06E-07 | 1,51E-06 | 4,3 |  | -2,9 | 1,55E-02 | 2,71E-02 |
| *SPATC1L* | 4,07E-07 | 1,51E-06 | -4,3 |  | -6,5 | 2,87E-06 | 9,37E-06 |
| *CAMK1* | 4,10E-07 | 1,52E-06 | -4,9 |  | -2,5 | 7,60E-02 | 1,12E-01 |
| *TSEN2* | 4,12E-07 | 1,53E-06 | -3,1 |  | -8,7 | 3,30E-12 | 2,01E-11 |
| *KLF11* | 4,13E-07 | 1,53E-06 | 3,5 |  | 1,8 | 3,48E-04 | 8,29E-04 |
| *CRKL* | 4,14E-07 | 1,54E-06 | 1,8 |  | 2,2 | 3,13E-21 | 3,56E-20 |
| *IGFBP2* | 4,19E-07 | 1,55E-06 | -3,0 |  | -2,7 | 6,55E-03 | 1,24E-02 |
| *FOCAD* | 4,21E-07 | 1,56E-06 | -1,7 |  | -4,4 | 1,43E-14 | 1,03E-13 |
| *ATP5MJ* | 4,22E-07 | 1,56E-06 | -1,9 |  | -2,7 | 6,24E-05 | 1,68E-04 |
| *RAVER2* | 4,22E-07 | 1,56E-06 | -3,2 |  | -4,4 | 4,64E-06 | 1,48E-05 |
| *SMAP2* | 4,24E-07 | 1,57E-06 | -2,0 |  | 0,6 | 2,54E-02 | 4,22E-02 |
| *SNX21* | 4,24E-07 | 1,57E-06 | -2,1 |  | -2,2 | 2,14E-01 | 2,77E-01 |
| *MAT2B* | 4,25E-07 | 1,58E-06 | 1,6 |  | 3,5 | 8,96E-52 | 3,40E-50 |
| *RAD50* | 4,27E-07 | 1,58E-06 | 3,7 |  | -3,3 | 1,46E-01 | 1,99E-01 |
| *GMIP* | 4,35E-07 | 1,61E-06 | 2,6 |  | 3,7 | 1,03E-12 | 6,48E-12 |
| *WDR6* | 4,41E-07 | 1,63E-06 | -1,7 |  | -2,1 | 2,16E-01 | 2,78E-01 |
| *CBLN3* | 4,41E-07 | 1,63E-06 | 11,4 |  | 4,3 | 4,98E-17 | 4,30E-16 |
| *ARHGAP18* | 4,45E-07 | 1,65E-06 | 1,8 |  | 1,6 | 1,01E-05 | 3,08E-05 |
| *RBM19* | 4,50E-07 | 1,66E-06 | 1,7 |  | 0,5 | 5,62E-03 | 1,08E-02 |
| *PHGDH* | 4,54E-07 | 1,68E-06 | -3,0 |  | -6,1 | 1,23E-08 | 5,32E-08 |
| *MPDZ* | 4,61E-07 | 1,70E-06 | -1,8 |  | -3,8 | 1,93E-17 | 1,72E-16 |
| *PLEKHF1* | 4,66E-07 | 1,72E-06 | 2,9 |  | 0,8 | 1,73E-02 | 3,00E-02 |
| *TCF4* | 4,67E-07 | 1,72E-06 | 2,1 |  | -2,2 | 2,50E-01 | 3,17E-01 |
| *CDKN2D* | 4,69E-07 | 1,73E-06 | 3,3 |  | 3,9 | 1,37E-18 | 1,34E-17 |
| *ANXA7* | 4,70E-07 | 1,73E-06 | 1,6 |  | 0,6 | 5,75E-06 | 1,82E-05 |
| *GEMIN7* | 4,70E-07 | 1,73E-06 | 2,2 |  | 3,1 | 4,44E-07 | 1,61E-06 |
| *CERK* | 4,71E-07 | 1,74E-06 | -1,6 |  | -6,2 | 7,86E-27 | 1,20E-25 |
| *TCEAL4* | 4,74E-07 | 1,75E-06 | -2,0 |  | -4,2 | 4,51E-17 | 3,91E-16 |
| *SEC61G* | 4,74E-07 | 1,75E-06 | 1,7 |  | 1,0 | 8,75E-03 | 1,61E-02 |
| *VCL* | 4,77E-07 | 1,76E-06 | -1,7 |  | -4,3 | 2,01E-53 | 7,85E-52 |
| *ARID3A* | 4,80E-07 | 1,77E-06 | 1,9 |  | 2,1 | 5,22E-17 | 4,50E-16 |
| *MAPK7* | 4,80E-07 | 1,77E-06 | 2,3 |  | 3,4 | 1,97E-15 | 1,50E-14 |
| *FIBIN* | 4,82E-07 | 1,77E-06 | -3,8 |  | -2,9 | 1,90E-02 | 3,26E-02 |
| *SHOX* | 4,86E-07 | 1,79E-06 | -2,6 |  | -2,1 | 8,65E-01 | 8,93E-01 |
| *SYAP1* | 4,87E-07 | 1,79E-06 | -1,5 |  | -3,1 | 1,29E-09 | 6,19E-09 |
| *EIF5A* | 4,87E-07 | 1,79E-06 | 1,5 |  | 0,7 | 8,36E-03 | 1,55E-02 |
| *GRB10* | 4,89E-07 | 1,80E-06 | 2,1 |  | 1,3 | 1,71E-11 | 9,85E-11 |
| *TTC27* | 4,95E-07 | 1,82E-06 | 1,8 |  | 0,0 | 7,97E-01 | 8,36E-01 |
| *ERBB2* | 4,97E-07 | 1,83E-06 | -1,7 |  | 0,2 | 1,34E-01 | 1,84E-01 |
| *AASDHPPT* | 4,98E-07 | 1,83E-06 | -1,8 |  | 0,2 | 2,16E-01 | 2,79E-01 |
| *ZBED1* | 4,99E-07 | 1,83E-06 | 1,8 |  | 0,7 | 4,52E-07 | 1,64E-06 |
| *MMD* | 5,06E-07 | 1,86E-06 | 2,5 |  | 0,5 | 4,53E-03 | 8,82E-03 |
| *OGA* | 5,07E-07 | 1,86E-06 | 1,5 |  | -2,6 | 1,34E-02 | 2,37E-02 |
| *FABP3* | 5,11E-07 | 1,87E-06 | -4,3 |  | -47,6 | 6,47E-07 | 2,30E-06 |
| *ZDHHC21* | 5,12E-07 | 1,88E-06 | 2,5 |  | 0,3 | 1,50E-01 | 2,04E-01 |
| *KDELR2* | 5,18E-07 | 1,90E-06 | -1,6 |  | -2,9 | 5,33E-05 | 1,45E-04 |
| *HRAS* | 5,19E-07 | 1,90E-06 | -1,9 |  | -2,4 | 1,52E-01 | 2,06E-01 |
| *FKBP15* | 5,20E-07 | 1,90E-06 | 1,6 |  | 0,9 | 3,35E-06 | 1,09E-05 |
| *EFR3A* | 5,20E-07 | 1,90E-06 | 1,6 |  | 0,0 | 8,58E-01 | 8,87E-01 |
| *POMGNT1* | 5,31E-07 | 1,94E-06 | 1,7 |  | 0,5 | 8,69E-02 | 1,26E-01 |
| *FOXP1* | 5,35E-07 | 1,96E-06 | 2,0 |  | -2,3 | 1,41E-01 | 1,93E-01 |
| *TTPAL* | 5,40E-07 | 1,97E-06 | -1,8 |  | -2,9 | 3,96E-07 | 1,45E-06 |
| *TMEM99* | 5,41E-07 | 1,98E-06 | -2,6 |  | -3,2 | 4,00E-03 | 7,88E-03 |
| *SPATS2* | 5,44E-07 | 1,99E-06 | -1,8 |  | -3,0 | 2,27E-05 | 6,59E-05 |
| *CCNYL1* | 5,53E-07 | 2,02E-06 | 2,7 |  | 0,3 | 1,69E-01 | 2,26E-01 |
| *UBE3D* | 5,56E-07 | 2,03E-06 | -3,9 |  | -6,0 | 1,75E-08 | 7,47E-08 |
| *STK17A* | 5,58E-07 | 2,04E-06 | -1,7 |  | 0,1 | 7,59E-01 | 8,03E-01 |
| *TGDS* | 5,62E-07 | 2,05E-06 | 2,4 |  | 0,5 | 5,80E-02 | 8,82E-02 |
| *RASSF7* | 5,66E-07 | 2,07E-06 | 3,1 |  | 3,9 | 6,26E-07 | 2,23E-06 |
| *EFHD2* | 5,67E-07 | 2,07E-06 | 2,0 |  | 1,8 | 3,31E-12 | 2,01E-11 |
| *MED13* | 5,73E-07 | 2,09E-06 | 1,7 |  | 0,5 | 1,87E-02 | 3,22E-02 |
| *ENOX1* | 5,74E-07 | 2,09E-06 | 2,9 |  | -2,1 | 7,94E-01 | 8,33E-01 |
| *NDUFAF2* | 5,78E-07 | 2,11E-06 | -2,2 |  | -3,4 | 1,63E-05 | 4,83E-05 |
| *POP5* | 5,80E-07 | 2,11E-06 | -4,0 |  | -5,4 | 1,97E-13 | 1,31E-12 |
| *RPAP2* | 5,80E-07 | 2,11E-06 | -1,9 |  | -2,7 | 4,30E-04 | 1,01E-03 |
| *POC1B* | 5,82E-07 | 2,12E-06 | 2,0 |  | 0,1 | 4,05E-01 | 4,77E-01 |
| *GRAMD1C* | 5,85E-07 | 2,13E-06 | 2,6 |  | 2,0 | 3,38E-06 | 1,09E-05 |
| *PYGO1* | 5,94E-07 | 2,16E-06 | -3,1 |  | -12,0 | 4,23E-19 | 4,26E-18 |
| *CTSA* | 5,99E-07 | 2,18E-06 | 1,5 |  | -2,4 | 5,03E-02 | 7,76E-02 |
| *GPR157* | 6,03E-07 | 2,19E-06 | 3,3 |  | 3,2 | 1,33E-11 | 7,71E-11 |
| *JADE2* | 6,11E-07 | 2,22E-06 | 1,9 |  | 5,4 | 4,12E-44 | 1,27E-42 |
| *STON1* | 6,12E-07 | 2,23E-06 | -7,1 |  | -12,4 | 6,94E-27 | 1,07E-25 |
| *RPL13* | 6,23E-07 | 2,26E-06 | -1,7 |  | -5,0 | 1,14E-39 | 3,03E-38 |
| *TPBG* | 6,24E-07 | 2,27E-06 | 1,6 |  | 1,4 | 6,93E-08 | 2,78E-07 |
| *KHDRBS3* | 6,25E-07 | 2,27E-06 | -3,3 |  | -4,1 | 8,12E-03 | 1,51E-02 |
| *TUBB2B* | 6,25E-07 | 2,27E-06 | -2,8 |  | 0,4 | 6,16E-01 | 6,77E-01 |
| *MECR* | 6,29E-07 | 2,28E-06 | -2,6 |  | -3,4 | 8,29E-07 | 2,91E-06 |
| *TRAF7* | 6,30E-07 | 2,29E-06 | -1,7 |  | -2,2 | 1,32E-01 | 1,82E-01 |
| *PKIA* | 6,33E-07 | 2,30E-06 | 2,5 |  | -2,5 | 2,86E-02 | 4,70E-02 |
| *GLE1* | 6,54E-07 | 2,37E-06 | -2,0 |  | 0,1 | 7,30E-01 | 7,78E-01 |
| *PDIA4* | 6,55E-07 | 2,37E-06 | 1,5 |  | 0,9 | 6,77E-04 | 1,53E-03 |
| *TMEM267* | 6,55E-07 | 2,37E-06 | -2,3 |  | -3,7 | 1,56E-06 | 5,27E-06 |
| *KLHL15* | 6,57E-07 | 2,38E-06 | -2,3 |  | -2,5 | 1,19E-01 | 1,66E-01 |
| *STIM2* | 6,65E-07 | 2,41E-06 | 1,8 |  | 1,5 | 1,58E-09 | 7,53E-09 |
| *TCP1* | 6,70E-07 | 2,43E-06 | -1,5 |  | -3,6 | 5,09E-25 | 7,09E-24 |
| *SOCS2* | 6,75E-07 | 2,44E-06 | -3,2 |  | -2,2 | 5,45E-01 | 6,12E-01 |
| *DLST* | 6,81E-07 | 2,46E-06 | 1,8 |  | 0,2 | 8,29E-02 | 1,21E-01 |
| *RXRB* | 6,83E-07 | 2,47E-06 | -1,8 |  | -2,1 | 3,31E-01 | 4,03E-01 |
| *STX6* | 6,87E-07 | 2,48E-06 | 1,7 |  | 0,1 | 5,75E-01 | 6,40E-01 |
| *RGMB* | 6,96E-07 | 2,52E-06 | -1,8 |  | 4,4 | 9,53E-29 | 1,59E-27 |
| *CLEC2D* | 7,03E-07 | 2,54E-06 | 9,2 |  | 1,6 | 3,76E-02 | 5,99E-02 |
| *WAKMAR2* | 7,04E-07 | 2,54E-06 | 72,5 |  | 4,0 | 6,02E-06 | 1,89E-05 |
| *ZZZ3* | 7,05E-07 | 2,54E-06 | -1,6 |  | -3,1 | 6,45E-07 | 2,29E-06 |
| *U2SURP* | 7,16E-07 | 2,58E-06 | 1,7 |  | 0,5 | 8,40E-03 | 1,55E-02 |
| *RCSD1* | 7,18E-07 | 2,59E-06 | 82,0 |  | 364,9 | 1,24E-13 | 8,37E-13 |
| *SLC35A4* | 7,19E-07 | 2,59E-06 | 1,9 |  | 1,7 | 5,91E-08 | 2,39E-07 |
| *SERPINB6* | 7,24E-07 | 2,61E-06 | 1,7 |  | 0,4 | 5,20E-02 | 8,00E-02 |
| *SAT2* | 7,33E-07 | 2,64E-06 | -2,2 |  | -6,7 | 3,73E-17 | 3,25E-16 |
| *LMNB2* | 7,37E-07 | 2,65E-06 | -1,5 |  | 0,3 | 2,63E-01 | 3,31E-01 |
| *FAM86DP* | 7,40E-07 | 2,66E-06 | -2,2 |  | -3,3 | 5,25E-03 | 1,01E-02 |
| *ACOT9* | 7,43E-07 | 2,68E-06 | 1,7 |  | 1,0 | 1,15E-09 | 5,53E-09 |
| *ACTN1* | 7,46E-07 | 2,68E-06 | 1,8 |  | 0,3 | 6,58E-02 | 9,87E-02 |
| *MIR29B2CHG* | 7,52E-07 | 2,71E-06 | 13,2 |  | 1,9 | 7,07E-02 | 1,05E-01 |
| *B4GALT1* | 7,54E-07 | 2,71E-06 | 4,1 |  | 2,3 | 1,01E-21 | 1,18E-20 |
| *CDCA7L* | 7,60E-07 | 2,73E-06 | 1,8 |  | -3,9 | 2,37E-05 | 6,86E-05 |
| *GGCX* | 7,67E-07 | 2,76E-06 | 1,7 |  | 1,0 | 1,79E-10 | 9,36E-10 |
| *UBTF* | 7,71E-07 | 2,77E-06 | -1,7 |  | -2,6 | 2,77E-06 | 9,06E-06 |
| *ZPR1* | 7,73E-07 | 2,78E-06 | 1,7 |  | 0,9 | 5,14E-06 | 1,63E-05 |
| *SCRIB* | 7,75E-07 | 2,78E-06 | -1,9 |  | -2,1 | 5,70E-01 | 6,35E-01 |
| *IRF2BPL* | 7,85E-07 | 2,82E-06 | 2,0 |  | 3,0 | 6,90E-25 | 9,53E-24 |
| *EVL* | 7,91E-07 | 2,84E-06 | -3,4 |  | -6,8 | 6,64E-11 | 3,57E-10 |
| *AFF4* | 7,95E-07 | 2,85E-06 | 1,9 |  | 0,8 | 9,78E-07 | 3,40E-06 |
| *HMGN2* | 8,05E-07 | 2,89E-06 | -1,8 |  | -2,9 | 7,17E-07 | 2,54E-06 |
| *SPDL1* | 8,08E-07 | 2,90E-06 | -2,1 |  | -2,7 | 1,01E-01 | 1,44E-01 |
| *ZFP91* | 8,14E-07 | 2,92E-06 | 1,7 |  | 0,9 | 2,68E-05 | 7,69E-05 |
| *ABCC10* | 8,24E-07 | 2,95E-06 | 1,9 |  | 1,1 | 1,09E-04 | 2,82E-04 |
| *NIM1K* | 8,24E-07 | 2,95E-06 | 9,9 |  | 14,9 | 2,64E-25 | 3,77E-24 |
| *TNKS1BP1* | 8,27E-07 | 2,96E-06 | 2,1 |  | 0,9 | 3,73E-06 | 1,20E-05 |
| *NPM1P27* | 8,29E-07 | 2,97E-06 | -2,1 |  | -6,2 | 2,56E-18 | 2,46E-17 |
| *SLC7A8* | 8,30E-07 | 2,97E-06 | -18,3 |  | -36,1 | 4,80E-24 | 6,30E-23 |
| *CDK13* | 8,33E-07 | 2,98E-06 | 1,6 |  | 0,1 | 6,99E-01 | 7,51E-01 |
| *RPS9* | 8,35E-07 | 2,99E-06 | -1,8 |  | -4,0 | 3,46E-18 | 3,27E-17 |
| *TGFB3* | 8,36E-07 | 2,99E-06 | -6,5 |  | -4,5 | 5,71E-07 | 2,04E-06 |
| *DNAJC6* | 8,38E-07 | 3,00E-06 | -2,6 |  | -3,2 | 2,97E-06 | 9,67E-06 |
| *SNHG1* | 8,39E-07 | 3,00E-06 | -2,2 |  | -3,8 | 2,33E-11 | 1,32E-10 |
| *RPL41P1* | 8,51E-07 | 3,04E-06 | -2,6 |  | -3,0 | 4,39E-03 | 8,57E-03 |
| *NBPF14* | 8,60E-07 | 3,07E-06 | 2,8 |  | 2,1 | 3,37E-10 | 1,71E-09 |
| *RRAGD* | 8,71E-07 | 3,11E-06 | 6,0 |  | 0,2 | 7,27E-01 | 7,75E-01 |
| *H3-3B* | 8,71E-07 | 3,11E-06 | 1,6 |  | 1,1 | 1,04E-09 | 5,07E-09 |
| *BCOR* | 8,79E-07 | 3,14E-06 | 2,4 |  | 3,5 | 4,44E-20 | 4,72E-19 |
| *CYP2U1* | 8,85E-07 | 3,16E-06 | -1,8 |  | -2,6 | 2,21E-04 | 5,43E-04 |
| *PRKCI* | 8,86E-07 | 3,16E-06 | -1,7 |  | -3,5 | 4,70E-12 | 2,82E-11 |
| *PRDX2* | 8,89E-07 | 3,17E-06 | -1,6 |  | -3,0 | 4,54E-07 | 1,64E-06 |
| *HEXA* | 8,89E-07 | 3,17E-06 | 1,7 |  | 0,1 | 6,67E-01 | 7,23E-01 |
| *ZNF774* | 8,95E-07 | 3,19E-06 | -9,3 |  | 0,2 | 7,46E-01 | 7,92E-01 |
| *MLLT3* | 9,07E-07 | 3,23E-06 | 1,9 |  | 1,2 | 9,96E-08 | 3,92E-07 |
| *NSMCE3* | 9,16E-07 | 3,26E-06 | -1,8 |  | 0,3 | 7,66E-02 | 1,13E-01 |
| *CDC20* | 9,18E-07 | 3,27E-06 | -2,8 |  | -3,2 | 9,53E-02 | 1,37E-01 |
| *WFS1* | 9,20E-07 | 3,27E-06 | -2,9 |  | -2,1 | 7,19E-01 | 7,69E-01 |
| *RICTOR* | 9,22E-07 | 3,28E-06 | 1,9 |  | 0,6 | 2,18E-02 | 3,69E-02 |
| *CDC14A* | 9,39E-07 | 3,33E-06 | 3,9 |  | 1,1 | 2,08E-02 | 3,54E-02 |
| *ETFDH* | 9,41E-07 | 3,34E-06 | -2,1 |  | -2,5 | 3,37E-03 | 6,73E-03 |
| *SRP54* | 9,43E-07 | 3,35E-06 | 1,6 |  | 0,6 | 6,88E-06 | 2,15E-05 |
| *FAM229B* | 9,49E-07 | 3,37E-06 | -2,6 |  | -4,1 | 1,84E-07 | 7,01E-07 |
| *MOB3A* | 9,53E-07 | 3,38E-06 | 2,1 |  | 0,8 | 2,55E-04 | 6,20E-04 |
| *MARCHF5* | 9,65E-07 | 3,42E-06 | 1,8 |  | 0,7 | 5,77E-04 | 1,32E-03 |
| *CDKL1* | 9,76E-07 | 3,46E-06 | 4,7 |  | 1,8 | 6,92E-04 | 1,56E-03 |
| *LBR* | 9,88E-07 | 3,50E-06 | -2,0 |  | -2,3 | 1,01E-01 | 1,44E-01 |
| *PPP1R11* | 9,95E-07 | 3,53E-06 | 1,9 |  | 0,6 | 5,65E-06 | 1,78E-05 |
| *EDNRA* | 9,98E-07 | 3,53E-06 | 2,0 |  | -5,2 | 6,92E-20 | 7,25E-19 |
| *UQCRB* | 9,98E-07 | 3,53E-06 | -1,9 |  | -5,2 | 2,00E-47 | 6,99E-46 |
| *ERMAP* | 1,01E-06 | 3,58E-06 | 2,2 |  | 0,6 | 5,71E-05 | 1,55E-04 |
| *APP* | 1,02E-06 | 3,61E-06 | 1,6 |  | 0,9 | 2,07E-06 | 6,90E-06 |
| *RPL19* | 1,02E-06 | 3,62E-06 | -1,7 |  | -3,6 | 4,21E-16 | 3,39E-15 |
| *MORF4L2* | 1,03E-06 | 3,65E-06 | -1,4 |  | -2,2 | 3,14E-01 | 3,85E-01 |
| *PGAP4* | 1,03E-06 | 3,65E-06 | -1,8 |  | 0,1 | 6,27E-01 | 6,86E-01 |
| *BAIAP2L1* | 1,04E-06 | 3,67E-06 | -17,4 |  | 0,4 | 9,76E-02 | 1,40E-01 |
| *GRAMD4* | 1,04E-06 | 3,68E-06 | -2,3 |  | -2,8 | 3,29E-03 | 6,59E-03 |
| *SLC25A13* | 1,04E-06 | 3,68E-06 | 1,9 |  | -2,1 | 7,56E-01 | 8,00E-01 |
| *TMEM106C* | 1,05E-06 | 3,70E-06 | -2,1 |  | -2,5 | 6,14E-03 | 1,17E-02 |
| *RNF111* | 1,06E-06 | 3,73E-06 | 1,8 |  | 1,4 | 6,04E-12 | 3,59E-11 |
| *PECR* | 1,06E-06 | 3,73E-06 | -4,2 |  | -4,7 | 4,12E-07 | 1,50E-06 |
| *ETFB* | 1,06E-06 | 3,74E-06 | -2,3 |  | -2,6 | 1,95E-02 | 3,34E-02 |
| *ERLEC1* | 1,07E-06 | 3,77E-06 | 1,6 |  | 0,2 | 1,78E-01 | 2,37E-01 |
| *RTCA* | 1,07E-06 | 3,78E-06 | 1,4 |  | 0,2 | 1,13E-01 | 1,60E-01 |
| *FBXO21* | 1,08E-06 | 3,79E-06 | -1,8 |  | -2,7 | 7,55E-06 | 2,35E-05 |
| *ZNF804A* | 1,08E-06 | 3,80E-06 | 2,3 |  | 3,5 | 9,36E-05 | 2,44E-04 |
| *APCDD1L* | 1,08E-06 | 3,81E-06 | 2,5 |  | 1,4 | 2,36E-03 | 4,87E-03 |
| *GLIS3* | 1,08E-06 | 3,81E-06 | 4,9 |  | 0,4 | 1,84E-02 | 3,17E-02 |
| *FAM117A* | 1,08E-06 | 3,81E-06 | -5,3 |  | -3,0 | 4,85E-02 | 7,51E-02 |
| *NCS1* | 1,09E-06 | 3,84E-06 | -1,5 |  | -4,6 | 1,34E-13 | 8,99E-13 |
| *SPC24* | 1,09E-06 | 3,84E-06 | -3,5 |  | -4,8 | 1,57E-05 | 4,68E-05 |
| *MEGF8* | 1,10E-06 | 3,86E-06 | -2,4 |  | -4,8 | 7,66E-15 | 5,60E-14 |
| *SKA2* | 1,10E-06 | 3,86E-06 | 1,7 |  | 0,0 | 8,29E-01 | 8,62E-01 |
| *KCTD5* | 1,10E-06 | 3,87E-06 | 2,1 |  | 1,3 | 1,95E-07 | 7,41E-07 |
| *SNAI3-AS1* | 1,10E-06 | 3,88E-06 | -3,3 |  | -6,6 | 1,16E-08 | 5,05E-08 |
| *PRDX3* | 1,11E-06 | 3,90E-06 | -1,6 |  | 0,3 | 6,93E-02 | 1,03E-01 |
| *DYRK1A* | 1,12E-06 | 3,92E-06 | 1,6 |  | 0,3 | 4,08E-02 | 6,44E-02 |
| *CYB561D2* | 1,12E-06 | 3,93E-06 | 2,5 |  | 2,0 | 9,80E-08 | 3,86E-07 |
| *ANAPC7* | 1,13E-06 | 3,96E-06 | -1,7 |  | -2,2 | 2,41E-01 | 3,07E-01 |
| *GM2A* | 1,14E-06 | 4,00E-06 | 1,7 |  | -2,7 | 2,91E-08 | 1,22E-07 |
| *TRIM26* | 1,15E-06 | 4,04E-06 | 1,9 |  | 3,9 | 5,54E-38 | 1,38E-36 |
| *NEK2* | 1,16E-06 | 4,06E-06 | -3,6 |  | -2,5 | 5,62E-01 | 6,28E-01 |
| *PAWR* | 1,17E-06 | 4,10E-06 | -1,7 |  | -3,0 | 1,03E-05 | 3,15E-05 |
| *ATP2A2* | 1,17E-06 | 4,10E-06 | 1,7 |  | 0,7 | 1,59E-05 | 4,72E-05 |
| *TIMM44* | 1,18E-06 | 4,13E-06 | -2,1 |  | -2,6 | 3,30E-03 | 6,61E-03 |
| *EGLN2* | 1,19E-06 | 4,17E-06 | 2,6 |  | -2,6 | 1,24E-01 | 1,72E-01 |
| *OPA1* | 1,20E-06 | 4,18E-06 | -1,5 |  | -3,1 | 1,10E-08 | 4,79E-08 |
| *SF3B5* | 1,20E-06 | 4,18E-06 | -1,8 |  | -2,8 | 3,34E-09 | 1,54E-08 |
| *ICE1* | 1,20E-06 | 4,19E-06 | 1,5 |  | 0,0 | 8,77E-01 | 9,03E-01 |
| *ARFRP1* | 1,21E-06 | 4,21E-06 | 1,9 |  | 1,6 | 2,50E-16 | 2,05E-15 |
| *SMARCAD1* | 1,21E-06 | 4,22E-06 | 1,7 |  | 0,1 | 5,55E-01 | 6,22E-01 |
| *GPAA1* | 1,21E-06 | 4,24E-06 | -1,8 |  | -2,7 | 1,83E-04 | 4,56E-04 |
| *GPRASP1* | 1,22E-06 | 4,25E-06 | -7,6 |  | -3,6 | 6,26E-03 | 1,19E-02 |
| *SHQ1* | 1,22E-06 | 4,27E-06 | -1,9 |  | -2,3 | 1,11E-01 | 1,56E-01 |
| *DAPK2* | 1,23E-06 | 4,31E-06 | 10,5 |  | -2,8 | 3,83E-01 | 4,56E-01 |
| *ELK4* | 1,25E-06 | 4,37E-06 | 1,9 |  | -2,4 | 2,06E-01 | 2,68E-01 |
| *ZNF415* | 1,25E-06 | 4,37E-06 | -3,9 |  | -4,3 | 2,55E-09 | 1,19E-08 |
| *MOCS2* | 1,26E-06 | 4,38E-06 | -2,0 |  | -2,9 | 3,12E-07 | 1,16E-06 |
| *TCEAL8* | 1,27E-06 | 4,41E-06 | -1,6 |  | -3,6 | 8,80E-16 | 6,92E-15 |
| *VPS37A* | 1,27E-06 | 4,42E-06 | 1,5 |  | 0,4 | 1,49E-02 | 2,62E-02 |
| *FLOT1* | 1,29E-06 | 4,50E-06 | -1,8 |  | 0,1 | 2,65E-01 | 3,33E-01 |
| *HDHD5* | 1,31E-06 | 4,55E-06 | 2,1 |  | 2,6 | 1,34E-07 | 5,20E-07 |
| *LMAN2* | 1,31E-06 | 4,58E-06 | 1,5 |  | 1,9 | 2,70E-11 | 1,51E-10 |
| *SIGMAR1* | 1,33E-06 | 4,63E-06 | -1,8 |  | -4,1 | 1,85E-21 | 2,13E-20 |
| *ACADVL* | 1,33E-06 | 4,63E-06 | 1,6 |  | 0,3 | 2,81E-02 | 4,62E-02 |
| *EEF1AKMT3* | 1,34E-06 | 4,65E-06 | 2,5 |  | 2,9 | 1,56E-08 | 6,71E-08 |
| *ENKD1* | 1,34E-06 | 4,67E-06 | 4,5 |  | 0,1 | 8,16E-01 | 8,51E-01 |
| *NOTCH1* | 1,34E-06 | 4,67E-06 | 2,8 |  | 1,8 | 6,03E-06 | 1,90E-05 |
| *DDB2* | 1,37E-06 | 4,76E-06 | -2,4 |  | -6,8 | 6,74E-29 | 1,13E-27 |
| *PSMD14* | 1,37E-06 | 4,77E-06 | 1,5 |  | 0,8 | 3,70E-05 | 1,04E-04 |
| *RESF1* | 1,38E-06 | 4,80E-06 | 1,8 |  | 0,1 | 8,08E-01 | 8,45E-01 |
| *ABCD1* | 1,39E-06 | 4,81E-06 | 2,8 |  | 4,1 | 2,14E-21 | 2,45E-20 |
| *VPS37D* | 1,39E-06 | 4,84E-06 | -6,8 |  | -2,4 | 3,07E-01 | 3,78E-01 |
| *LSM7* | 1,40E-06 | 4,85E-06 | -2,0 |  | -4,1 | 6,34E-11 | 3,42E-10 |
| *MAP4K2* | 1,40E-06 | 4,87E-06 | 1,8 |  | 0,9 | 1,01E-04 | 2,61E-04 |
| *DUSP16* | 1,42E-06 | 4,93E-06 | 2,0 |  | 5,2 | 3,42E-16 | 2,77E-15 |
| *FAM114A2* | 1,43E-06 | 4,94E-06 | -1,9 |  | -2,1 | 7,14E-01 | 7,64E-01 |
| *PRDX1* | 1,43E-06 | 4,96E-06 | -1,6 |  | -2,4 | 1,17E-02 | 2,09E-02 |
| *IGHMBP2* | 1,44E-06 | 4,97E-06 | 2,1 |  | 0,8 | 4,06E-03 | 7,99E-03 |
| *ENSG00000257354* | 1,45E-06 | 5,01E-06 | 4,6 |  | -10,4 | 1,84E-09 | 8,71E-09 |
| *FTO* | 1,45E-06 | 5,01E-06 | -1,8 |  | -3,1 | 1,99E-07 | 7,57E-07 |
| *DERL1* | 1,45E-06 | 5,01E-06 | 1,5 |  | 1,4 | 1,19E-09 | 5,72E-09 |
| *DPH1* | 1,45E-06 | 5,02E-06 | -2,1 |  | -2,7 | 5,93E-03 | 1,13E-02 |
| *ARF5* | 1,46E-06 | 5,04E-06 | -2,0 |  | -3,0 | 1,59E-07 | 6,10E-07 |
| *DYRK2* | 1,48E-06 | 5,10E-06 | -1,8 |  | -4,2 | 2,53E-12 | 1,55E-11 |
| *IRX5* | 1,48E-06 | 5,11E-06 | -5,1 |  | -2,8 | 2,09E-02 | 3,55E-02 |
| *H3C2* | 1,49E-06 | 5,14E-06 | -2,5 |  | -3,2 | 5,82E-02 | 8,86E-02 |
| *SVEP1* | 1,49E-06 | 5,16E-06 | -13,6 |  | -6,7 | 2,92E-12 | 1,78E-11 |
| *UBQLN1* | 1,50E-06 | 5,17E-06 | 1,6 |  | 0,9 | 3,20E-14 | 2,25E-13 |
| *MPP6* | 1,51E-06 | 5,20E-06 | -2,5 |  | -5,2 | 1,67E-28 | 2,75E-27 |
| *ZXDA* | 1,51E-06 | 5,21E-06 | 3,7 |  | 0,4 | 2,19E-01 | 2,82E-01 |
| *MAPRE2* | 1,51E-06 | 5,22E-06 | -2,0 |  | -3,0 | 4,51E-07 | 1,64E-06 |
| *R3HCC1L* | 1,52E-06 | 5,25E-06 | 2,0 |  | 0,7 | 1,64E-02 | 2,86E-02 |
| *UFD1* | 1,52E-06 | 5,25E-06 | 1,5 |  | 0,4 | 2,36E-03 | 4,87E-03 |
| *NXT2* | 1,54E-06 | 5,32E-06 | 3,0 |  | 1,8 | 1,39E-06 | 4,74E-06 |
| *UBL7* | 1,54E-06 | 5,32E-06 | -1,8 |  | 0,2 | 2,83E-01 | 3,53E-01 |
| *ARMC10* | 1,55E-06 | 5,34E-06 | -1,9 |  | -2,9 | 3,30E-06 | 1,07E-05 |
| *DLGAP5* | 1,56E-06 | 5,38E-06 | -2,8 |  | -6,3 | 1,07E-05 | 3,26E-05 |
| *TRIM65* | 1,56E-06 | 5,38E-06 | 2,0 |  | 0,2 | 8,75E-02 | 1,27E-01 |
| *CLU* | 1,56E-06 | 5,38E-06 | 2,4 |  | -2,6 | 9,79E-05 | 2,55E-04 |
| *PCCA* | 1,57E-06 | 5,40E-06 | -2,6 |  | -3,7 | 4,21E-05 | 1,17E-04 |
| *ZNF140* | 1,57E-06 | 5,41E-06 | 2,1 |  | 0,4 | 3,74E-02 | 5,96E-02 |
| *KDM5A* | 1,57E-06 | 5,41E-06 | 1,7 |  | 0,3 | 4,00E-03 | 7,88E-03 |
| *TAB3* | 1,59E-06 | 5,47E-06 | 1,9 |  | 1,4 | 1,00E-06 | 3,48E-06 |
| *KLHL3* | 1,60E-06 | 5,49E-06 | -3,3 |  | -2,2 | 3,68E-01 | 4,40E-01 |
| *DBNDD1* | 1,60E-06 | 5,50E-06 | -3,2 |  | 0,3 | 5,44E-01 | 6,12E-01 |
| *ZNF654* | 1,60E-06 | 5,50E-06 | 2,0 |  | 1,2 | 2,65E-06 | 8,68E-06 |
| *TPRA1* | 1,60E-06 | 5,51E-06 | 2,0 |  | 1,4 | 3,91E-13 | 2,54E-12 |
| *COG6* | 1,61E-06 | 5,52E-06 | -1,5 |  | -3,6 | 5,99E-17 | 5,14E-16 |
| *NTAN1* | 1,62E-06 | 5,55E-06 | 1,6 |  | 0,3 | 1,40E-02 | 2,46E-02 |
| *BRD3OS* | 1,62E-06 | 5,57E-06 | -2,0 |  | -2,9 | 5,05E-05 | 1,38E-04 |
| *MAPKAPK2* | 1,66E-06 | 5,68E-06 | 1,7 |  | 0,5 | 4,23E-05 | 1,18E-04 |
| *GPR173* | 1,66E-06 | 5,70E-06 | 5,6 |  | 2,7 | 1,41E-04 | 3,59E-04 |
| *UBE2H* | 1,68E-06 | 5,74E-06 | -1,5 |  | -2,7 | 1,59E-04 | 4,00E-04 |
| *ASB8* | 1,68E-06 | 5,74E-06 | -1,8 |  | -2,0 | 8,89E-01 | 9,12E-01 |
| *PTHLH* | 1,69E-06 | 5,78E-06 | 9,3 |  | 0,3 | 7,81E-01 | 8,22E-01 |
| *DNER* | 1,69E-06 | 5,78E-06 | 1,9 |  | 26,2 | 3,17E-09 | 1,46E-08 |
| *FAM174A* | 1,69E-06 | 5,79E-06 | 2,6 |  | 0,7 | 1,04E-02 | 1,88E-02 |
| *FNDC3A* | 1,69E-06 | 5,80E-06 | 1,6 |  | 0,7 | 4,03E-05 | 1,13E-04 |
| *BOD1L1* | 1,74E-06 | 5,97E-06 | 2,2 |  | -2,7 | 2,20E-02 | 3,72E-02 |
| *NDE1* | 1,75E-06 | 5,97E-06 | 2,0 |  | 0,3 | 1,57E-01 | 2,12E-01 |
| *MAD2L1BP* | 1,75E-06 | 5,98E-06 | 1,9 |  | 1,3 | 8,52E-09 | 3,75E-08 |
| *NTM* | 1,76E-06 | 6,01E-06 | -1,5 |  | -6,6 | 2,51E-13 | 1,66E-12 |
| *GPR137* | 1,76E-06 | 6,02E-06 | 2,3 |  | 1,7 | 2,44E-09 | 1,14E-08 |
| *AKIRIN2* | 1,80E-06 | 6,15E-06 | 2,8 |  | 1,4 | 7,33E-06 | 2,28E-05 |
| *EME2* | 1,81E-06 | 6,17E-06 | 2,1 |  | 0,3 | 3,14E-01 | 3,85E-01 |
| *HELB* | 1,81E-06 | 6,18E-06 | 7,5 |  | 8,0 | 1,89E-14 | 1,35E-13 |
| *TBL1X* | 1,82E-06 | 6,20E-06 | -2,0 |  | 0,3 | 1,64E-01 | 2,21E-01 |
| *TXLNA* | 1,82E-06 | 6,22E-06 | 1,6 |  | 0,1 | 2,15E-01 | 2,78E-01 |
| *NSFL1C* | 1,83E-06 | 6,25E-06 | 1,8 |  | 1,6 | 5,60E-10 | 2,79E-09 |
| *BCL2* | 1,83E-06 | 6,25E-06 | -4,9 |  | -6,0 | 1,08E-03 | 2,36E-03 |
| *PTPN22* | 1,84E-06 | 6,27E-06 | -4,0 |  | -2,7 | 9,87E-03 | 1,80E-02 |
| *LCOR* | 1,86E-06 | 6,34E-06 | 2,2 |  | 0,5 | 1,63E-04 | 4,09E-04 |
| *NDUFS8* | 1,87E-06 | 6,36E-06 | -1,7 |  | -2,1 | 5,11E-01 | 5,81E-01 |
| *TFG* | 1,89E-06 | 6,46E-06 | 1,5 |  | 0,7 | 9,41E-05 | 2,46E-04 |
| *LINC01128* | 1,90E-06 | 6,46E-06 | 2,4 |  | -2,3 | 1,47E-02 | 2,59E-02 |
| *RAB35* | 1,92E-06 | 6,53E-06 | 1,7 |  | 1,1 | 2,23E-14 | 1,58E-13 |
| *PNRC1* | 1,93E-06 | 6,58E-06 | 1,8 |  | 4,2 | 7,56E-62 | 3,58E-60 |
| *ENSG00000278948* | 1,94E-06 | 6,61E-06 | -2,4 |  | -3,4 | 1,89E-04 | 4,68E-04 |
| *CCDC57* | 1,94E-06 | 6,61E-06 | 2,6 |  | -2,9 | 2,53E-02 | 4,21E-02 |
| *UBTD1* | 1,95E-06 | 6,63E-06 | -1,9 |  | -3,1 | 9,90E-06 | 3,03E-05 |
| *VEPH1* | 1,96E-06 | 6,65E-06 | 1,5 |  | -2,7 | 2,90E-02 | 4,75E-02 |
| *COPG1* | 1,98E-06 | 6,71E-06 | 1,5 |  | 0,4 | 3,86E-02 | 6,13E-02 |
| *RASSF4* | 1,99E-06 | 6,75E-06 | 4,2 |  | 1,2 | 9,59E-04 | 2,11E-03 |
| *CIDECP1* | 1,99E-06 | 6,75E-06 | 2,9 |  | 1,2 | 1,41E-04 | 3,59E-04 |
| *SLC2A3* | 2,01E-06 | 6,81E-06 | 1,6 |  | 1,0 | 7,58E-05 | 2,01E-04 |
| *RFX3* | 2,01E-06 | 6,81E-06 | 5,6 |  | 0,3 | 3,53E-01 | 4,25E-01 |
| *CYCS* | 2,01E-06 | 6,82E-06 | -1,7 |  | -2,6 | 8,46E-03 | 1,56E-02 |
| *PPP1R7* | 2,02E-06 | 6,86E-06 | -2,0 |  | -2,8 | 4,83E-05 | 1,33E-04 |
| *RABGGTA* | 2,03E-06 | 6,88E-06 | 2,0 |  | 2,1 | 2,54E-08 | 1,07E-07 |
| *ARHGEF28* | 2,03E-06 | 6,88E-06 | -1,6 |  | 0,2 | 2,09E-01 | 2,71E-01 |
| *ATP5F1C* | 2,06E-06 | 6,99E-06 | -1,7 |  | -2,2 | 1,71E-01 | 2,28E-01 |
| *LAMTOR4* | 2,07E-06 | 7,00E-06 | -2,1 |  | -3,2 | 8,39E-08 | 3,34E-07 |
| *ZNF432* | 2,07E-06 | 7,00E-06 | 2,0 |  | 0,5 | 6,93E-02 | 1,03E-01 |
| *RENBP* | 2,07E-06 | 7,00E-06 | 10,9 |  | 4,6 | 3,87E-04 | 9,14E-04 |
| *HEATR5A* | 2,09E-06 | 7,07E-06 | -1,7 |  | -6,3 | 3,28E-20 | 3,52E-19 |
| *NEIL2* | 2,09E-06 | 7,07E-06 | -2,0 |  | -3,1 | 3,99E-04 | 9,41E-04 |
| *ZFP36L2* | 2,12E-06 | 7,17E-06 | -2,8 |  | -2,1 | 5,65E-01 | 6,31E-01 |
| *TMEM170B* | 2,17E-06 | 7,34E-06 | -9,8 |  | -6,8 | 3,21E-05 | 9,10E-05 |
| *COMMD6* | 2,17E-06 | 7,35E-06 | -1,7 |  | -3,2 | 6,61E-07 | 2,35E-06 |
| *SLC16A2* | 2,17E-06 | 7,35E-06 | -1,8 |  | 0,3 | 7,14E-02 | 1,06E-01 |
| *POLR2H* | 2,18E-06 | 7,37E-06 | 1,7 |  | 0,9 | 1,75E-05 | 5,16E-05 |
| *PCOLCE2* | 2,19E-06 | 7,40E-06 | -3,8 |  | -3,6 | 8,86E-09 | 3,90E-08 |
| *COPS4* | 2,21E-06 | 7,46E-06 | -1,7 |  | -2,5 | 1,00E-02 | 1,82E-02 |
| *SEMA4C* | 2,22E-06 | 7,50E-06 | 2,1 |  | 0,6 | 9,81E-04 | 2,16E-03 |
| *NUP43* | 2,22E-06 | 7,50E-06 | -1,7 |  | -2,5 | 2,53E-03 | 5,20E-03 |
| *PELI3* | 2,22E-06 | 7,50E-06 | -3,0 |  | -2,4 | 4,23E-02 | 6,66E-02 |
| *DARS1* | 2,23E-06 | 7,52E-06 | 1,6 |  | 1,1 | 1,30E-09 | 6,26E-09 |
| *TTLL11* | 2,23E-06 | 7,53E-06 | 3,7 |  | 1,2 | 3,74E-03 | 7,42E-03 |
| *ADIPOR2* | 2,24E-06 | 7,56E-06 | -1,6 |  | -2,1 | 4,17E-01 | 4,89E-01 |
| *CHROMR* | 2,25E-06 | 7,58E-06 | 2,7 |  | 2,1 | 5,40E-09 | 2,43E-08 |
| *THEM4* | 2,26E-06 | 7,62E-06 | -2,4 |  | -5,1 | 3,02E-11 | 1,69E-10 |
| *NDUFA12* | 2,27E-06 | 7,64E-06 | -1,9 |  | -3,0 | 1,38E-06 | 4,70E-06 |
| *GAPLINC* | 2,31E-06 | 7,79E-06 | -11,4 |  | -145,8 | 2,24E-18 | 2,16E-17 |
| *ZNF841* | 2,32E-06 | 7,80E-06 | 2,0 |  | 0,2 | 1,23E-01 | 1,71E-01 |
| *OSBPL10* | 2,33E-06 | 7,83E-06 | -2,0 |  | -2,6 | 1,12E-01 | 1,58E-01 |
| *NUP153* | 2,35E-06 | 7,92E-06 | 1,5 |  | 1,3 | 1,67E-15 | 1,28E-14 |
| *QDPR* | 2,38E-06 | 8,00E-06 | -3,2 |  | -3,5 | 7,52E-05 | 2,00E-04 |
| *ZDHHC5* | 2,39E-06 | 8,05E-06 | 1,6 |  | 1,0 | 3,38E-14 | 2,37E-13 |
| *CRTC2* | 2,40E-06 | 8,06E-06 | 2,6 |  | 2,1 | 3,37E-10 | 1,71E-09 |
| *ACOT4* | 2,42E-06 | 8,14E-06 | 14,8 |  | 40,1 | 6,75E-27 | 1,04E-25 |
| *AP1B1* | 2,42E-06 | 8,14E-06 | 1,7 |  | 1,1 | 4,39E-05 | 1,22E-04 |
| *LYST* | 2,47E-06 | 8,28E-06 | -2,2 |  | -4,9 | 1,46E-06 | 4,94E-06 |
| *PTK2* | 2,47E-06 | 8,30E-06 | 1,5 |  | 0,5 | 5,49E-05 | 1,49E-04 |
| *DNAJC21* | 2,50E-06 | 8,38E-06 | -1,7 |  | -2,9 | 9,76E-09 | 4,28E-08 |
| *SUCLG1* | 2,51E-06 | 8,43E-06 | -1,6 |  | -2,8 | 1,52E-05 | 4,53E-05 |
| *HOXB2* | 2,52E-06 | 8,46E-06 | -2,2 |  | -2,1 | 4,05E-01 | 4,77E-01 |
| *TUT7* | 2,53E-06 | 8,49E-06 | 1,6 |  | 0,4 | 2,04E-03 | 4,27E-03 |
| *FASTK* | 2,55E-06 | 8,55E-06 | -1,6 |  | -3,1 | 2,24E-05 | 6,50E-05 |
| *PAFAH1B2* | 2,55E-06 | 8,56E-06 | 1,6 |  | 1,1 | 3,51E-07 | 1,29E-06 |
| *WDFY1* | 2,56E-06 | 8,58E-06 | 1,7 |  | 2,3 | 8,19E-13 | 5,21E-12 |
| *URGCP* | 2,56E-06 | 8,59E-06 | 1,7 |  | 2,7 | 4,63E-30 | 8,15E-29 |
| *DYNC1LI2* | 2,57E-06 | 8,60E-06 | 1,5 |  | 0,2 | 1,16E-01 | 1,62E-01 |
| *GNAI2* | 2,57E-06 | 8,62E-06 | 1,5 |  | 0,9 | 1,42E-04 | 3,61E-04 |
| *PYCARD* | 2,58E-06 | 8,62E-06 | 4,4 |  | 2,4 | 1,27E-07 | 4,93E-07 |
| *POLR1B* | 2,58E-06 | 8,64E-06 | -1,8 |  | -2,3 | 2,02E-01 | 2,63E-01 |
| *DPYD* | 2,60E-06 | 8,69E-06 | 1,6 |  | 1,8 | 1,95E-36 | 4,61E-35 |
| *GABPB2* | 2,62E-06 | 8,77E-06 | -2,3 |  | -4,4 | 1,33E-14 | 9,58E-14 |
| *ABHD18* | 2,63E-06 | 8,81E-06 | 2,5 |  | 0,6 | 3,61E-02 | 5,77E-02 |
| *CARM1* | 2,67E-06 | 8,94E-06 | -1,7 |  | 0,2 | 3,39E-01 | 4,11E-01 |
| *PDLIM1* | 2,71E-06 | 9,07E-06 | 2,0 |  | 2,8 | 4,16E-10 | 2,09E-09 |
| *LRRC8A* | 2,72E-06 | 9,10E-06 | 1,6 |  | 0,3 | 1,66E-01 | 2,23E-01 |
| *URB1* | 2,73E-06 | 9,12E-06 | -2,0 |  | -3,6 | 3,31E-18 | 3,14E-17 |
| *UQCRC2* | 2,73E-06 | 9,12E-06 | -1,6 |  | -2,4 | 9,33E-03 | 1,71E-02 |
| *IL17D* | 2,73E-06 | 9,12E-06 | -7,8 |  | -38,8 | 8,50E-13 | 5,40E-12 |
| *ENPP2* | 2,73E-06 | 9,13E-06 | -2,9 |  | -5,3 | 4,25E-33 | 8,73E-32 |
| *BROX* | 2,74E-06 | 9,14E-06 | 1,6 |  | 0,5 | 3,26E-02 | 5,27E-02 |
| *CDC42BPB* | 2,76E-06 | 9,20E-06 | 1,6 |  | 0,4 | 1,87E-03 | 3,93E-03 |
| *CCNB2* | 2,78E-06 | 9,26E-06 | -3,4 |  | -3,3 | 1,07E-02 | 1,94E-02 |
| *UVSSA* | 2,79E-06 | 9,28E-06 | 3,5 |  | 0,1 | 8,57E-01 | 8,86E-01 |
| *TBC1D2B* | 2,80E-06 | 9,33E-06 | 1,6 |  | -2,4 | 1,47E-02 | 2,58E-02 |
| *AKT3* | 2,80E-06 | 9,34E-06 | 1,7 |  | 0,2 | 5,39E-02 | 8,26E-02 |
| *SPTY2D1* | 2,81E-06 | 9,37E-06 | 1,6 |  | 0,1 | 4,61E-01 | 5,32E-01 |
| *FAM43A* | 2,82E-06 | 9,39E-06 | 2,8 |  | -2,5 | 9,97E-02 | 1,43E-01 |
| *NPLOC4* | 2,84E-06 | 9,45E-06 | 1,5 |  | 0,8 | 6,49E-07 | 2,31E-06 |
| *UTP20* | 2,85E-06 | 9,48E-06 | -1,7 |  | -4,4 | 9,20E-16 | 7,20E-15 |
| *ARSJ* | 2,85E-06 | 9,49E-06 | -1,7 |  | 2,1 | 1,48E-02 | 2,61E-02 |
| *MED28* | 2,87E-06 | 9,53E-06 | -1,6 |  | -3,1 | 1,26E-05 | 3,79E-05 |
| *ANAPC11* | 2,90E-06 | 9,63E-06 | -1,8 |  | -2,5 | 1,86E-02 | 3,20E-02 |
| *FAM155A* | 2,92E-06 | 9,70E-06 | 2,4 |  | -2,8 | 3,36E-02 | 5,42E-02 |
| *PSPH* | 2,93E-06 | 9,75E-06 | -2,3 |  | -3,2 | 4,31E-06 | 1,38E-05 |
| *GNS* | 2,94E-06 | 9,75E-06 | 1,6 |  | 0,6 | 1,38E-06 | 4,70E-06 |
| *TRMU* | 2,96E-06 | 9,82E-06 | -1,8 |  | -2,3 | 2,13E-01 | 2,76E-01 |
| *DCLRE1B* | 2,96E-06 | 9,83E-06 | 2,4 |  | 0,9 | 1,07E-03 | 2,34E-03 |
| *SERTAD4* | 2,97E-06 | 9,85E-06 | -4,7 |  | -7,0 | 2,02E-15 | 1,53E-14 |
| *RPLP0P6* | 2,98E-06 | 9,87E-06 | -3,5 |  | -9,2 | 4,92E-11 | 2,68E-10 |
| *ACSF2* | 2,98E-06 | 9,88E-06 | -2,0 |  | -2,5 | 1,38E-04 | 3,52E-04 |
| *EBNA1BP2* | 3,00E-06 | 9,93E-06 | -1,8 |  | -2,6 | 2,96E-02 | 4,83E-02 |
| *RPAIN* | 3,01E-06 | 9,96E-06 | -1,7 |  | -2,9 | 3,67E-04 | 8,71E-04 |
